# Synthesis of 2-oxindoles via 'transition-metal-free' intramolecular dehydrogenative coupling (IDC) of sp^2^ C–H and sp^3^ C–H bonds

**DOI:** 10.3762/bjoc.12.111

**Published:** 2016-06-08

**Authors:** Nivesh Kumar, Santanu Ghosh, Subhajit Bhunia, Alakesh Bisai

**Affiliations:** 1 Department of Chemistry, Indian Institute of Science Education and Research Bhopal, Bhopal Bypass Road, Bhauri, Bhopal – 462 066, Madhya Pradesh, India

**Keywords:** C−H functionalization, intramolecular dehydrogenative coupling (IDC), iodine, *N*-iodosuccinimide, oxidants, 2-oxindoles

## Abstract

The synthesis of a variety of 2-oxindoles bearing an all-carbon quaternary center at the pseudo benzylic position has been achieved via a ‘transition-metal-free’ intramolecular dehydrogenative coupling (IDC). The construction of 2-oxindole moieties was carried out through formation of carbon–carbon bonds using KO*t-*Bu-catalyzed one pot C-alkylation of β-*N*-arylamido esters with alkyl halides followed by a dehydrogenative coupling. Experimental evidences indicated toward a radical-mediated path for this reaction.

## Introduction

The C−H functionalization is an attractive synthetic strategy used in organic synthesis for the development of atom- and step-economical routes [1–10]. In recent years it was witnessed a mushrooming growth in the number of reports in the literature owing to the efficiency of the oxidative coupling of two C–H bonds [also termed as cross-dehydrogenative-coupling (CDC)] in the formation of C–C bonds [11–16]. This was facilitated by the introduction of transition metals in organic synthesis providing an amazing tool to explore these oxidative coupling reactions in an efficient manner. However, despite the associated advantages, these methodologies require one or two metal catalysts for efficient reactions, which are sometimes undesirable [17–21]. Therefore, an alternate strategy to carry out these transformations under 'transition-metal-free' conditions has recently gained immense importance.

**Scheme 1:**
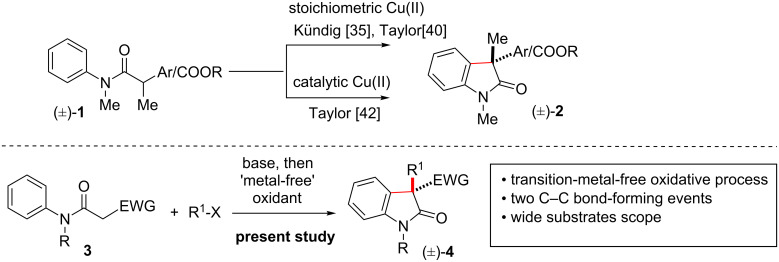
Synthesis of 2-oxindoles via oxidative processes.

2-Oxindoles having all carbon quaternary centres at the pseudobenzylic position are common structural scaffolds in many naturally occurring alkaloids of biological relevance [22–25]. These heterocyclic motifs especially exist in indole alkaloids with a wide spectrum of biological and pharmacological properties and hence are very attractive as well as challenging synthetic targets [26]. Selected examples for the synthesis of 2-oxindole include an intramolecular homolytic aromatic substitution on the aryl ring by an amidyl radical formed by homolysis of a C–X bond [27–30], single electron transfer (SET) to a α-halo anilides followed by halide elimination [31–32], and the formation of an aryl radical followed by a 1,5-hydrogen atom translocation [33–34]. Out of these strategies, the initial two require specifically functionalized precursors such as the presence of an *o*-halogen, an *o*-selenium, or an *o*-xanthate, respectively. One of the direct approaches to 2-oxindoles could be a one-electron oxidation of an amide enolate as shown in Scheme 1. Toward this end, in 2009, Kündig and co-workers have developed a novel route to 3,3-disubstituted-2-oxindoles while working on asymmetric synthesis of 3,3-disubstituted-2-oxindoles via a Pd-catalyzed (chiral *N*-heterocyclic carbene as ligands) intramolecular α-arylation of an amide [35–37]. For this 'intramolecular dehydrogenative coupling' (IDC) of Csp^2^-H and Csp^3^-H they used 2.2 equiv of CuCl_2_ and 5 equiv of NaO*t*-Bu [38–39].

In the same year, Taylor and co-workers independently reported synthesis of 2-oxindoles in the presence of Cu(OAc)_2_·H_2_O as oxidant (Scheme 1) [40–44]. Experimental evidence suggests involvement of a free-radical process in the addition of α-carbonylalkyl radicals to the phenyl ring. The α-carbonylalkyl radicals were formed by Cu(II)-mediated oxidation of the respective enolate precursors. In 2010, Yu and co-workers have reported the synthesis of 3-acetyloxindoles via Ag_2_O-mediated intramolecular oxidative coupling [45]. For the past few years, our group is engaged in the development of efficient methodologies for the synthesis of 2-oxindoles with intriguing ring systems. To this end, recently, we have reported a transition-metal-free ‘intramolecular-dehydrogenative-coupling' (IDC) strategy to access such 2-oxindole moieties through a C-alkylation followed by an oxidative construction of the C–C bond (Scheme 1) [46]. Applying the aforementioned strategy, we were able to synthesize several 3-alkyl-2-oxindoles bearing ester functionalities at the pseudobenzylic position from β-*N*-arylamido allyl, methallyl, dimethylallyl, and geranyl esters. Here, in this article, we disclose the scope and limitations of 'transition-metal-free' IDC of Csp^2^-H and Csp^3^-H using iodine and *N*-iodosuccinimide (NIS) as oxidants. In addition, we have also demonstrated the synthetic utility of oxidative coupling products in the syntheses of 3-substituted-2-oxindoles, via a decarboxylative protonation on 2-oxindoles bearing an benzylester or *para*-methoxybenzyl ester at the 3-position in presence of a catalytic amount of Pd on activated charcoal. We have also shown the direct installation of allyl, prenyl, *reverse*-prenyl, or geranyl groups at the 3-position of 2-oxindole using Pd-catalyzed decarboxylative strategies [47].

## Results and Discussion

We decided to use iodine as an oxidant for the synthesis of 2-oxindoles [48–53], starting from β-*N*-arylamido ester **3a** and methyl iodide as the substrates (Table 1). An elaborate optimization study suggested that the methylation can be done in the presence of 1.2 equivalents of KO*t-*Bu and 1.1 equivalents of methyl iodide. This was accompanied with an oxidative coupling using 1.2 equivalents of KO*t*-Bu and iodine to afford the desired product in 65% yield (Table 1, entries 1 and 2). Optimization studies in search of suitable solvent, potential bases, oxidants etc. yielded the desired product in good yields i.e. 85%, 88%, and 90% in THF, dioxane, and DMSO, respectively (Table 1, entries 3, 5, and 8). However, in non-polar aromatic solvents like xylene, benzene, and toluene, poor yields (43–49%, Table 1, entries 4, 6, and 7) of products were observed with reactions being unclean (mixture of products) [54]. KO*t*-Bu was superior over other bases used in this reaction like NaH, NaOMe, K_2_CO_3_, Cs_2_CO_3_, and NaO*t-*Bu (Table 1, entries 9–13). Among other metal-free oxidants, iodosobenzenediacetate (PIDA), DBDMH (1,3-dibromo-5,5-dimethylhydantoin), and ICl afforded 2-oxindole **4a** in 82%, 16%, and 69%, respectively (Table 1, entries 17–19). Later, we turned our attention to *N*-halo succinimides as potential oxidants in our methodology [53]. Interestingly, *N*-iodosuccinimide (NIS), *N*-bromosuccinimide (NBS), and *N*-chlorosuccinimide (NCS) afforded **4a** in 84%, 75%, and 58% yields, respectively (Table 1, entries 20–22). However, trichloroisocyanuric acid (TCICA) is found to be inefficient in oxidative coupling and afforded 56–62% yields of **4a** (Table 1, entries 23 and 24). In the absence of iodine or NIS, no product was formed. Eventually, the combination of 1.2 equivalents of iodine (conditions A) or NIS (conditions B) were found to be the best and chosen for further studies (Table 1, entries 14 and 20).

**Table 1:** Optimization of intramolecular-dehydrogenative-coupling (IDC)^a^.

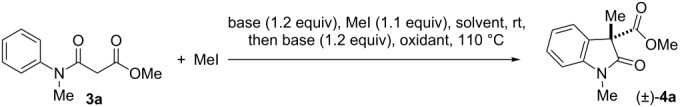						

entry	solvent	base	Alkylations at 25 °C	oxidants	time	% **4a**^b,c^

1.	DMF	*t*-BuOK	20 min	1.5 equiv I_2_	6 h	65%
2.	DMF	*t*-BuOK	20 min	1.2 equiv I_2_	3 h	62%
3.	THF	*t*-BuOK	30 min	1.2 equiv I_2_	3 h	85%
4.	xylene	*t*-BuOK	45 min	1.2 equiv I_2_	1 h	49%^d^
5.	dioxane	*t*-BuOK	20 min	1.2 equiv I_2_	2 h	88%
6.	benzene	*t*-BuOK	50 min	1.2 equiv I_2_	2 h	43%^d^
7.	toluene	*t*-BuOK	45 min	1.2 equiv I_2_	1 h	45%^d^
8.	DMSO	*t*-BuOK	20 min	1.2 equiv I_2_	30 min	90%
9.	DMSO	NaH	20 min	1.2 equiv I_2_	30 min	33%
10.	DMSO	NaOMe	2 h	1.2 equiv I_2_	30 min	–^e^
11.	DMSO	K_2_CO_3_	1 h^f^	–	–	–
12.	DMSO	CS_2_CO_3_	2 h	1.5 equiv I_2_	30 min	26%^g^
13.	DMSO	*t*-BuONa	30 min	1.5 equiv I_2_	30 min	^–g^
**14.**	**DMSO**	***t*-BuOK**	**15 min**	**1.2 equiv I_2_**	**30 min**	**90%**
15.	DMSO	*t*-BuOK	15 min	0.6 equiv I_2_	1 h	54%
16.	DMSO	*t*-BuOK	15 min	0.3 equiv I_2_	1 h	29%
17.	DMSO	*t*-BuOK	15 min	1.2 equiv PIDA	30 min	82%
18.	DMSO	*t*-BuOK	15 min	1.2 equiv DBDMH^h^	30 min	16%^g^
19.	DMSO	*t*-BuOK	15 min	1.2 equivICl	30 min	69%
**20.**	**DMSO**	***t*-BuOK**	**15 min**	**1.2 equiv NIS**	**30 min**	**84%**
21.	DMSO	*t*-BuOK	15 min	1.2 equiv NBS	30 min	75%
22.	DMSO	*t*-BuOK	15 min	1.2 equiv NCS	30 min	58%
23.	DMSO	*t*-BuOK	15 min	1.0 equivTCICA^i^	30 min	62%
24.	DMSO	*t*-BuOK	15 min	0.5 equivTCICA^i^	30 min	56%

^a^ Entries 1–18 have been reproduced from our preliminary communication (reference [46]. ^b^Reactions were carried out on a 0.25 mmol of **3a** with 0.275 mmol of methyl iodide in presence of 0.30 mmol of base in 1 mL of solvent at 25 °C for specified time for alkylations and 0.275 mmol of oxidant in presence of 0.30 mmol of base under heating at 110 °C for oxidative coupling steps, unless noted otherwise. ^c^Isolated yields of **4a** after column chromatography. ^d^Mixture of products were observed for rest of the mass balance. ^e^C-methylation as major product. ^f^Starting material was recovered (92%). ^g^Decomposition of starting materials. ^h^DBDMH (1,3-dibromo-5,5-dimethylhydantoin) as oxidant. ^i^TCICA (trichloroisocyanuric acid).

**Figure 1:**
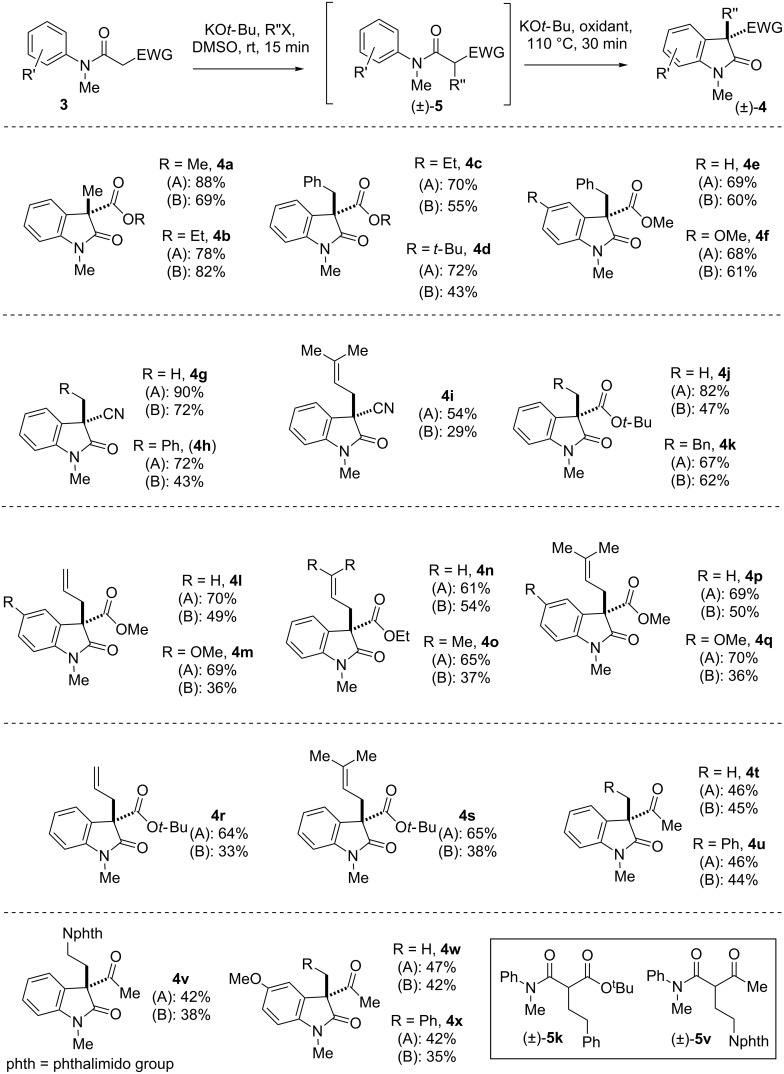
Substrates scope of one-pot ‘transition-metal-free’ IDC. The syntheses of compounds **4a**–**s** according to method A have been reproduced from reference [46]. Conditions A: KO*t*-Bu, iodine; conditions B: KO*t-*Bu, NIS.

Next, the substrate scope of the reaction was explored as shown in Figure 1. A variety of substrates were prepared by a coupling reaction of *N*-methyl arylamines and monoalkyl malonates/cyanoacetic acids. Under optimized conditions A and B, various β-*N*-arylamido esters and nitriles (**3**) were subjected to a one-pot alkylations using 1.2 equivalents of KO*t*-Bu to produce C-alkylated intermediate **5** followed by oxidative coupling using 1.2 equivalents iodine or NIS. Gratifyingly, it was found that a range of β-*N*-arylamido esters (**3a–s**) and β-*N*-arylamido ketones (**3t–x**) underwent intramolecular dehydrogenative coupling (IDC) under both conditions A and B to afford a wide range of 2-oxindoles (**4a–x**) having an all-carbon quaternary center in high yields. However, we observed that in case of 2-oxindoles **4v** and **4k**, a two-step protocol is necessary, where in first step C-alkylation of β-*N*-arylamido ketone was carried out using 1.2 equivalents of KO*t*-Bu to afford products **5v** and **5k**, respectively (Figure 1), followed by a second oxidative coupling reaction in the presence of iodine or NIS.

**Figure 2:**
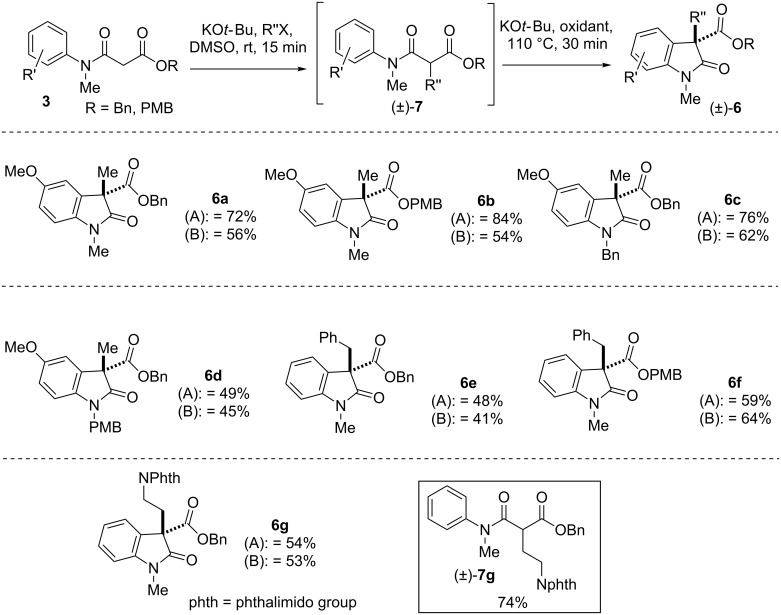
Further substrates scope of one-pot ‘transition metal-free’ IDC. Conditions A: KO*t-*Bu, iodine; conditions B: KO*t*-Bu, NIS.

We envisioned that the oxidative coupling products containing benzyl or *p*-methoxybenzyl ester could be effective intermediates for the synthesis of 3-monosubstituted 2-oxindoles via deprotection of the benzyl group followed by decarboxylative protonation in presence of a catalytic amount of Pd on activated charcoal under hydrogenolysis. Thus, we explored the substrate scope using β-*N*-arylamido benzyl ester or β-*N*-arylamido *p*-methoxybenzyl ester as starting materials for the oxidative coupling reaction shown in Figure 2. Towards this end, β-*N*-aryl amido benzylester or β-*N*-arylamido p-methoxybenzyl ester **3** were subjected to an one pot alkylation to generate the intermediate **7** followed by oxidative coupling reaction using our optimized conditions A and B to furnish products of type (±)-**6** in good yields (Figure 2). For the synthesis of compound (±)-**6g**, we followed a two-step protocol: In first step a C-alkylation of β-*N*-arylamido benzylester in presence of 1.2 equivalents of NaH and alkylating agent afford compound (±)-**7g** in good yields (74%), followed by an oxidative coupling in presence of 1.2 equivalents of KO*t*-Bu and iodine or NIS as oxidant.

**Figure 3:**
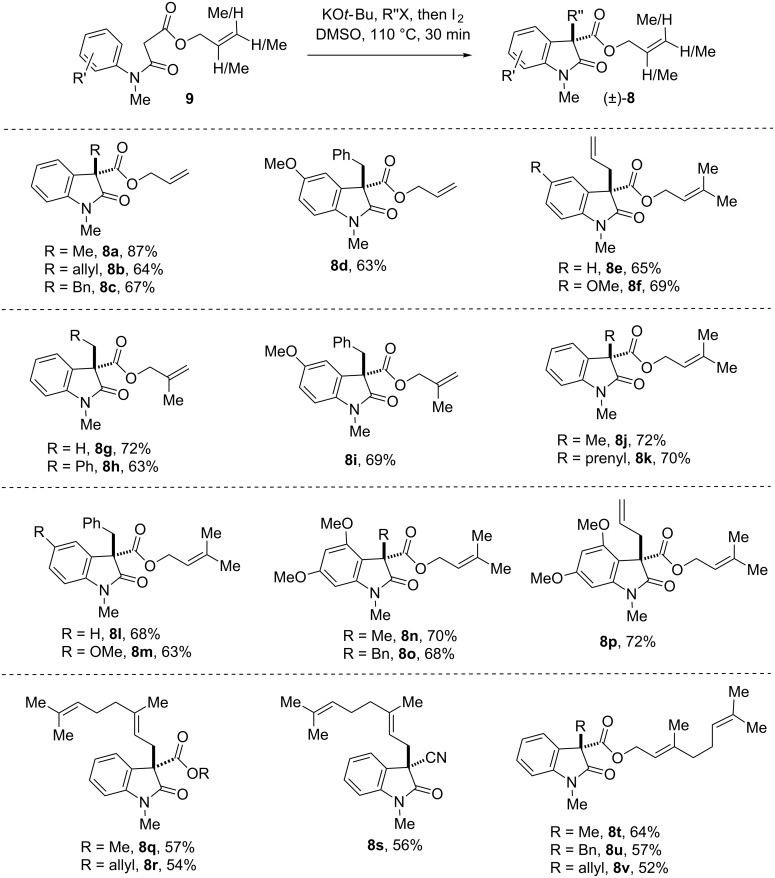
Substrates scope of ‘transition-metal-free’ IDC using KO*t*-Bu/I_2_. Reproduced from [46].

Next, we focussed our attention to prenylated, *reverse*-prenylated, and geranylated hexahydropyrrolo[2,3-*b*]indole alkaloids showing broad biological activities [55–61]. For the synthesis of these compounds, we thought of utilizing the Pd-catalyzed decarboxylative strategy to install the prenyl, *reverse*-prenyl, or geranyl group at the 3-position of 2-oxindole starting from the corresponding β-amido esters such as **8** [47]. This further extended the methodology to a variety of β-*N*-arylamido esters containing allyl, methallyl, dimethylallyl, and geranyl ester groups (**9**). It is noteworthy that, the substrate of type **9** could undergo smooth IDC in the presence of iodine (conditions A) to provide an access to compounds **8** in synthetically useful yields (Figure 3).

Noticeably, we could directly construct the 2-oxindoles with a geranyl group at the 3-position using geranyl bromide as an alkylating agent. Upon a subsequent oxidative coupling step, products in good yields (**8q–s**, Figure 3) were formed using conditions A. Later, the IDC was extended to substrates having β-*N*-arylamido geranyl esters to afford compounds **8t–v** (Figure 3). These compounds could be excellent substrates for carrying out Tsuji–Trost decarboxylative geranylations/*reverse*-geranylations [62–63]. However, conditions B (NIS) were found unsuccessful in case of β-*N*-arylamidoallyl, methallyl, dimethylallyl, and geranyl esters **9**. We speculate that the olefin functionality of substrates might be reacting with NIS (conditions B) faster than iodine (conditions A). Although our iodine-mediated IDC is successful in most of the cases, however, in few cases we have seen moderate yields of products. Thus, we decided to carry out IDC in the presence of organic bases as well.

Thus, for an alternative approach to 2-oxindoles bearing allyl, methallyl, dimethylallyl, and geranyl esters, we were interested for IDC using simple organic bases such as triethylamine, pyridine, and DABCO (Table 2) [64]. It was found that IDC can operate in the presence of organic bases to afford products only in 25–34% yields of 2-oxindoles (Table 2, entries 1, 2 and 4). These reactions were always associated with unreacted starting material (28–51%) and decomposition of the rest of the mass balance. Interestingly, when the base was changed to DBU (using 1.5 equiv DBU and 1.2 equiv of iodine) the desired 2-oxindole was isolated in 82% (conditions C).

**Table 2:** IDC in the presence of organic bases. Reproduced from [46].

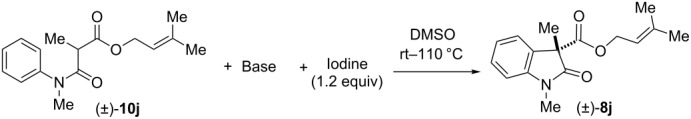				

entry	base	time	% of **8j**	% of **10j**

1.	pyridine	12 h	29	30
2.	Et_3_N	12 h	25	28
**3.**	**DBU**	**40 min**	**82**	**–**
4.	DABCO	12 h	34	51

^a^Reactions were carried out on a 0.25 mmol of **10j** using 0.50 mmol of base and 0.275 mmol of iodine in 1 mL of solvent for specified time.

**Figure 4:**
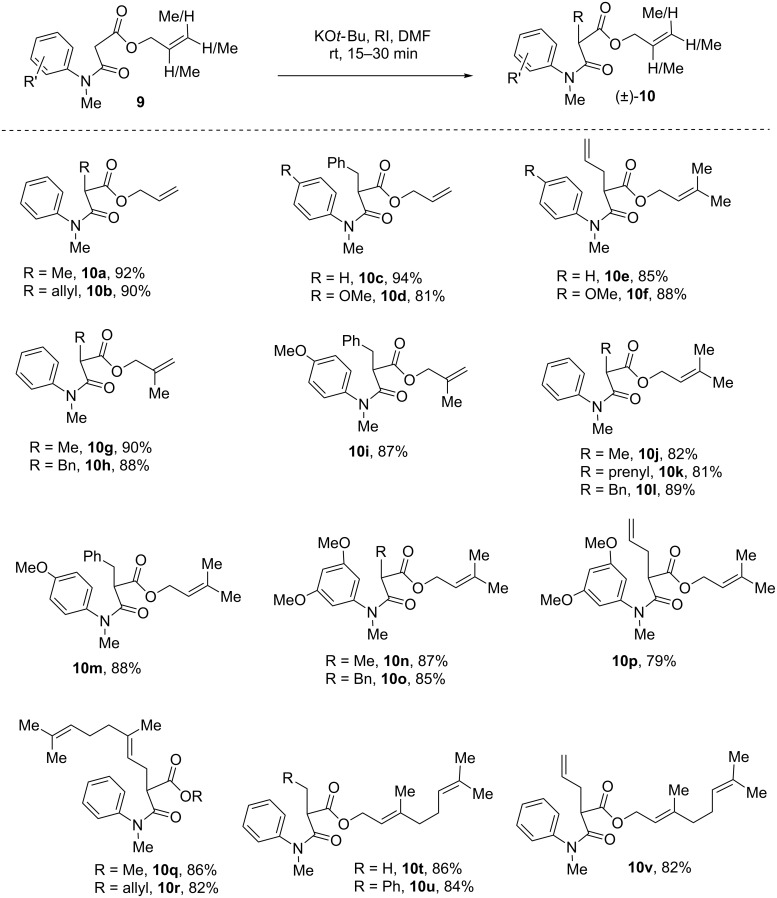
C-Alkylation of anilides using KO*t*-Bu.

**Figure 5:**
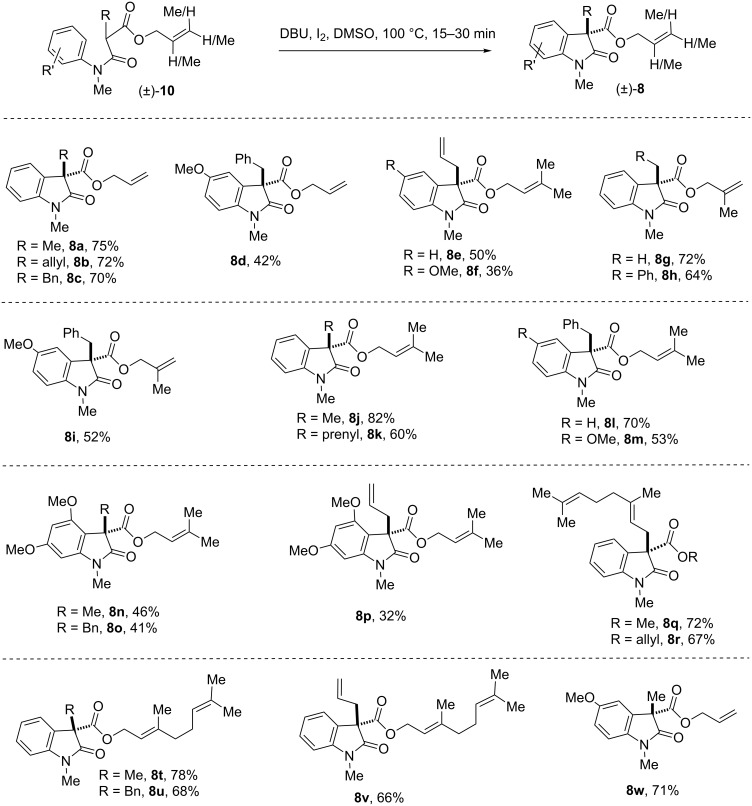
Substrates scope of *‘*transition-metal-free’ IDC of C-alkylated anilides using DBU/I_2_.

With this result in hand, we thought of exploring IDC using C-alkylated substrates **10**. For this purpose, a variety of C-alkylated β-*N*-arylamidoallyl, methallyl, dimethylallyl, and geranyl esters **10** were synthesized in good yields as per Figure 4. These substrates were then utilized in IDC-promoted by DBU/I_2_ and the results are summarized in Figure 5. Interestingly, under this conditions, we can synthesize a variety of 2-oxindoles **8** in moderate to good yields.

**Scheme 2:**
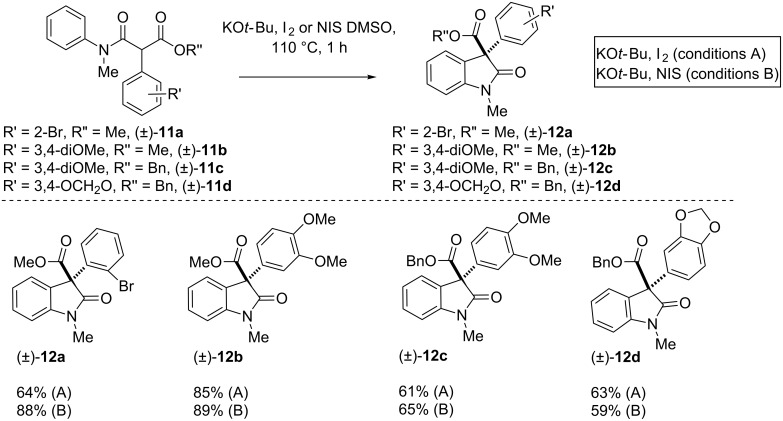
Oxidative coupling of C-arylated anilides (±)-**11a–d**. The synthesis of **12b** as per method A has been reproduced from reference [46].

There are a large number of indole alkaloids bearing a 3-arylated-2-oxindole moiety that are known for their various biological activities [65–67]. In a quest for such structural scaffolds, C-arylated substrates (±)-**11a–d** were subjected to standard reaction conditions to afford compound **12a–d** (Scheme 2). To our pleasure, C-arylated β-*N*-arylamidoesters (±)-**11a–d** afforded products (±)-**12a–d** in 59–89% yield after 1 h under conditions A and B.

**Scheme 3:**
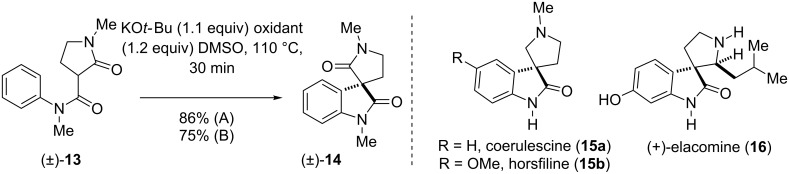
Synthesis of spirocyclic product through IDC The synthesis of **14** as per method A has been reproduced from reference [46]. Conditions A: KO*t*-Bu, iodine; conditions B: KO*t*-Bu, NIS.

Our synthetic methodology was further explored in the construction of spiro-fused oxindole ring systems (Scheme 3). The spiro-fused oxindoles such as coerulescine (**15a**) [68–72], horsfiline (**15b**) [73], and elacomine (**16**), are prevalently found in a huge number of indole-based alkaloids having analgesic properties. Our oxidative methodology offered us a direct access to the core structures of these alkaloids under the optimized IDC conditions in high yields (Scheme 3).

**Scheme 4:**
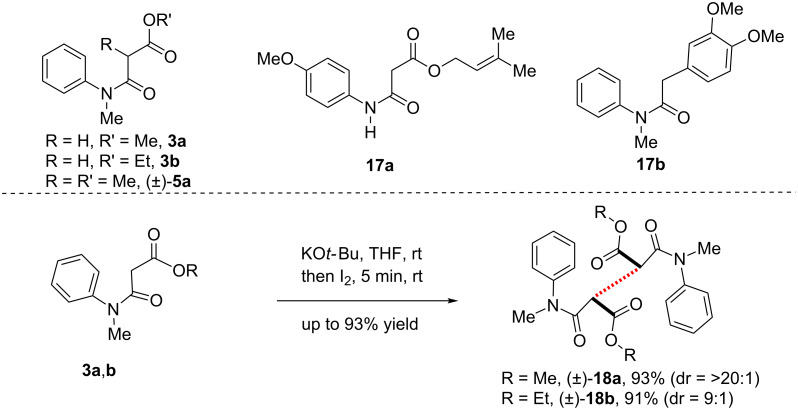
Dimerization of β-*N*-aryl-amidoesters **3a** and **b**. Reproduced from [46].

Next, we thought of carrying out the IDC without alkylations of compounds **3a** and **b** and **17a** and **b** (Scheme 4). Unfortunately, we could not isolate products due to decomposition under optimized IDC conditions. It was noticed that changing the solvent to THF effected very fast (within 5 minutes) dimerization of **3a** and **b** at room temperature to afford **18a** and **b** as sole products in 91–93% yield and in up to >20:1 dr (Scheme 4). This shows that formation of a stabilized tertiary radical probably facilitates the IDC process for the syntheses of 2-oxindoles.

**Scheme 5:**
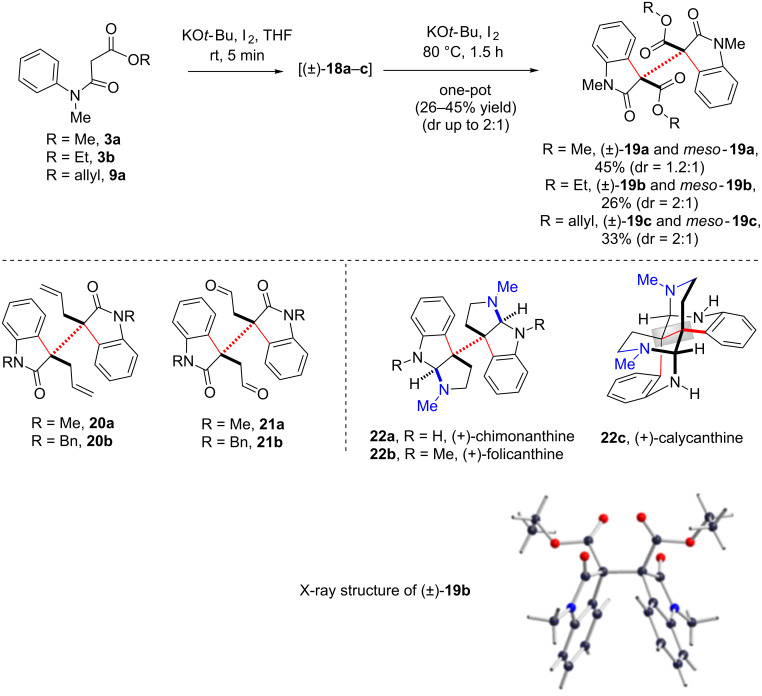
Synthesis of dimeric 2-oxindoles utilizing IDC. The syntheses of **19a** and **b** have been reproduced from [46].

However, if a tertiary radical is responsible for the oxidative process, then one would realize the formation of dimeric 2-oxindoles sharing vicinal all-carbon quaternary centers from dimeric β-*N*-arylamidoesters **18a** and **b** (Scheme 4). The reason behind our interest towards this direction was due to the prevalence of various dimeric cyclotryptamine alkaloids containing 3a,3a'-bis-pyrrolo[2,3-*b*]indole subunits (core structure of alkaloids **22a** and **b**, see, Scheme 5) [74–77], sharing a vicinal all-carbon quaternary stereogenic centers with extreme steric congestion at the C3a–C3a' σ-bond as well as the attendant lability of this linkage. Under the optimized conditions, one-pot dimerization of β-*N*-arylamido ester **3a** and **b** and **9a** took place in the presence of 1.2 equivalents of KO*t*-Bu and I_2_ followed by a double IDC on treatment with 1.2 equivalents of KO*t*-Bu and I_2_ affording the dimeric 2-oxindoles (±)-**19a–c** in poor to moderate yields (26–45% yield and 2:1 dr) along with 15–18% isolation of dimeric β-*N*-arylamidoesters (±)-**18a–c** (Scheme 5). This transformation is an efficient one-pot formation of three consecutive carbon–carbon bonds. X-ray crystal structure determination of (±)-**19b** proved the outcome of the reaction unambiguously. It was noteworthy to observe Pd-catalyzed highly enantio-, chemo-, and diastereoselective double decarboxylative allylations on dimeric β-*N*-arylamido allyl ester **19c** to yield the enantiopure compounds of type **20a** and **b** in good yields [78–81]. Especially, enantioenriched **20b** is the advanced intermediate for the total syntheses of 3a,3a'-bispyrrolo[2,3-*b*]indole alkaloids, chimonanthine (**22a**), folicanthine (**22b**), and their rearranged skeleton such as calycanthine (**22c**) (Scheme 5) [78].

**Scheme 6:**
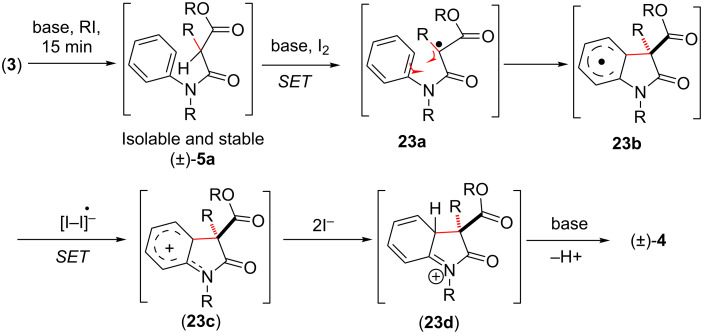
Plausible mechanism of ‘transition-metal-free’ IDC The mechanistic consideration in Scheme 6 has been reproduced from [46].

In all the cases, IDC was feasible with substrates having substituents at the carbon atom α- to the amides. This gave a clue for a radical-mediated process where a single electron transfer (SET) mechanism might be operating. A tentative mechanism has been proposed in Scheme 6, the reaction can adopt a SET mechanism leading to the intermediate **23a**, after C-alkylation. Compound **23a** in turn gets converted into intermediate aryl radical **23b**. From this intermediate another intermediate aryl carbocation **23c** is formed by transferring a single electron to the oxidant. Carbocation **23c** is stabilized by the amide nitrogen as shown in **23d**. Eventually, in the presence of base, rearomatization of **23d** takes place to afford the final product of the oxidative coupling reaction.

**Scheme 7:**
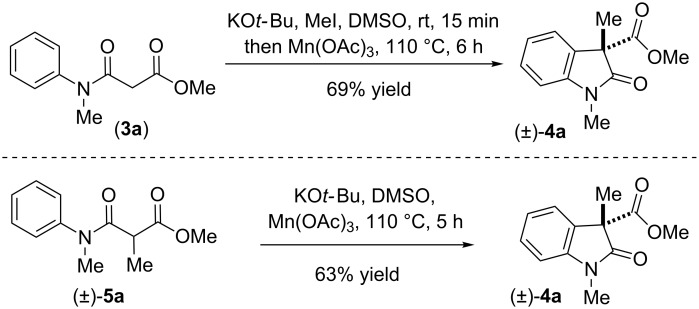
Intramolecular-dehydrogenative-coupling (IDC) of **3a** and **5a**. Reproduced from [46].

Kündig et al. in their oxidative coupling process using 2.2 equivalent of CuCl_2_ showed that it is important to have a tertiary carbon α- to the amide for the process to be radical mediated [38–39]. Also, it is well evident from literature that the oxidation processes using Mn(OAc)_3_ as oxidant follow a radical pathway [82–86]. In fact, the reaction of **3a** also afforded 2-oxindole **4a** in 69% yield when the oxidative coupling was carried out in presence of 1.2 equiv of Mn(OAc)_3_ (Scheme 7). A similar result was also observed when reaction was carried out using C-methyl β-*N*-arylamido ester **5a** (Scheme 7) [82–86].

**Scheme 8:**
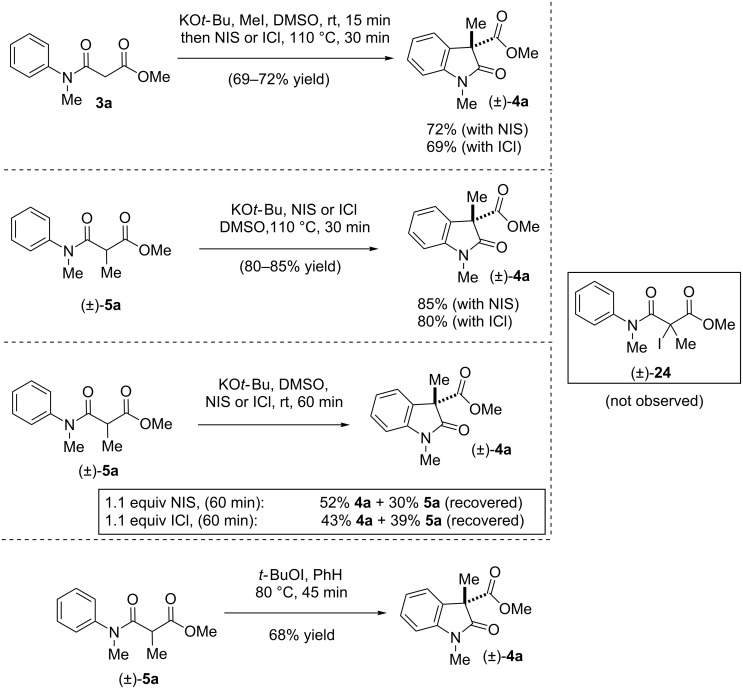
IDC of **3a** and **5a** using different oxidants. Reproduced from [46].

However, one can’t rule out the possibility of a substitution reaction on C-iodo product **24** from the adjacent aryl group (Scheme 8). Thus, the possibilty of the addition at the 2-position of electron-rich *N*-acylated aniline **3a** to the tertiary iodide intermediate was also investigated. Towards this, we thought of synthesizing the C-iodo intermediate using *N*-iodosuccinimide (NIS) or ICl in the presence of a base. Surprisingly, our all effort to prepare C-iodo compound **24** in the presence of KO*t-*Bu as a base only led to formation of 2-oxindole **4a** in 72% and 69% yields, respectively (Scheme 8). Along the same line, C-methyl β-*N*-arylamido ester **5a** also afforded product **4a** in 80–85% yield when the reaction was carried out at elevated temperature (Scheme 8). We thought there could be the possibility of a substitution reaction of iodide compound **24** prepared in-situ to form directly 2-oxindole **4a** under elevated temperature. Thus, it was decided to carry out the C-iodination at room temperature, where substitution reactions would be unlikely, considering the fact that the substitution has to occur at the sterically congested tertiary iodide **24**. However, to our surprise, when C-iodination of **5a** was carried out at rt, we found that it also afforded 2-oxindole **4a** in 30–39% yield along with 43–52% of recovered starting material (Scheme 8) and no trace of C-iodide **24** was observed. These results suggest that, NIS and ICl also acts as oxidants and helping in a single electron transfer (SET) in the oxidative coupling reaction [87–88]. It is also well evident in the literature that, these can also be used as oxidants in variety of oxidative coupling reactions [87–88]. Further, oxidative coupling of **5a** was carried out in presence of well-known *t*-BuOI, which generally goes through a radical-mediated pathway [89–90]. Towards this, when the oxidative coupling was carried out in presence of in situ generated *t*-BuOI [91], the reaction afforded oxidative coupling product (*±*)**-4a** in 68% yield, which is also probably indicating a radical pathway of the reaction (Scheme 8).

**Scheme 9:**
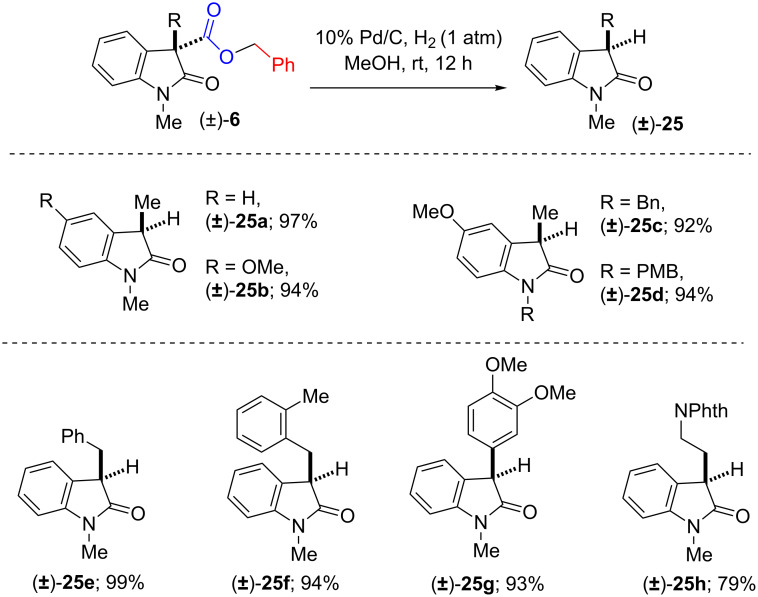
Synthesis of 3-substituted-2-oxindoles from benzyl esters.

Shifting our attention towards the synthetic application of our IDC methodology, we put forward our effort towards the synthesis of 3-alkylated or arylated 2-oxindoles. Towards this, we subjected to react, the oxidative coupling products (±)-**6,** (±)-**12c** and **d** having benzyl (Bn) or *p*-methoxybenzyl (PMB) esters with a catalytic amount of Pd on activated charcoal (10% Pd on charcoal) under atmospheric pressure of hydrogen gas in MeOH/EtOH (Scheme 9).

**Scheme 10:**
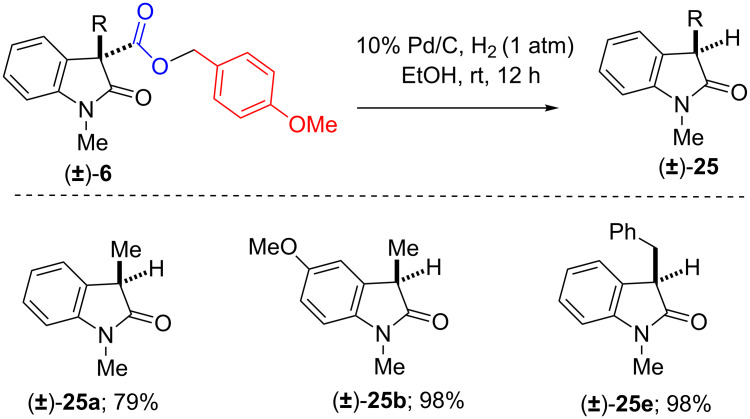
3-Substituted-2-oxindoles from p-methoxybenzyl esters.

Interestingly, we observed that the oxidative coupling products undergo deprotection of benzyl or p-methoxybenzyl group and provided the intermediate carboxylic acid, followed by decarboxylative protonation in the same pot gave us the desired products (±)-**25a**–**h** in excellent yields (Scheme 9 and Scheme 10).

**Scheme 11:**
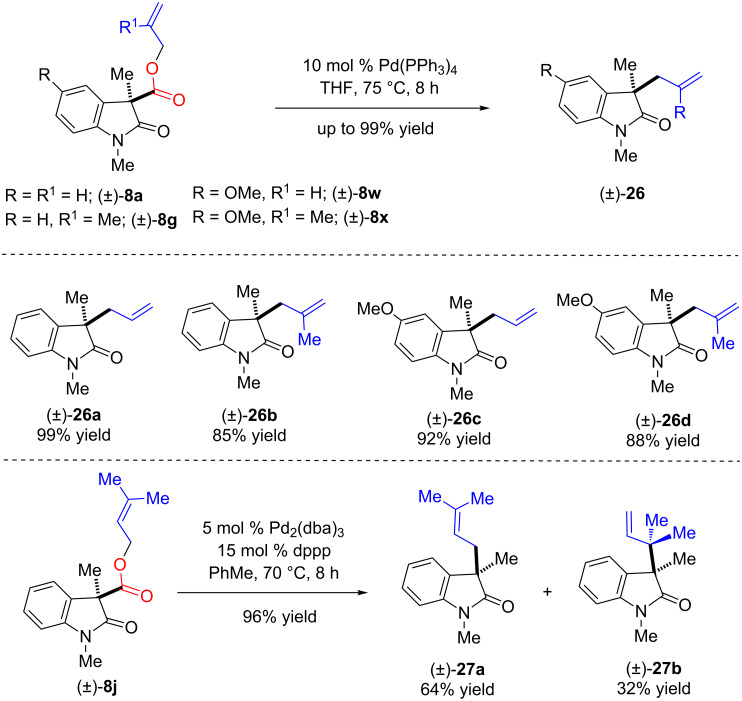
Synthetic elaboration using Tsuji–Trost reactions. Reproduced from [46].

Later, we envisioned that the oxidative coupling products having allyl, methallyl, dimethylallyl esters after Trost–Tsuji decarboxylative allylations could serve as an interesting platform for complex natural product synthesis after further synthetic elaboration and functionalization. A few substrates were treated under decarboxylative allylation (DcA) conditions in the presence of 10 mol % of Pd(PPh_3_)_4_ in refluxing tetrahydrofuran (7–8 h), which afforded products **26a**–**d** in up to 99% yield (Scheme 11). Interestingly, oxidative coupling products with dimethylallyl esters **8j** underwent smooth decarboxylative prenylation and *reverse*-prenylation in the presence of 5 mol % of Pd_2_(dba)_3_ and 15 mol % dppp in refluxing toluene (7–8 h, 96% yields) to afford prenylated (**27a**) and *reverse*-prenylated (**27b**) structures in 64% and 32% yield, respectively (Scheme 11) [92–93]. These structures commonly occur in many hexahydropyrrolo[2,3-*b*]indole-based alkaloids.

## Conclusion

In summary, we have successfully demonstrated the synthesis of 2-oxindoles bearing an all-carbon quaternary center applying a ‘transition-metal-free’ intramolecular dehydrogenative coupling (IDC) strategy. The methodology has been broadly applied to a wide range of substrates affording 2-oxindoles in good yields in a facile one-pot C-alkylation concomitant with oxidative coupling strategy. These products serve as a great synthetic platform for several indole-based natural products. The methodology demonstrated here has several advantages: (i) C-alkylations can be carried in same pot; (ii) simple oxidants like iodine and *N*-iodosuccinimide (NIS) could be used in the absence of any transition metal which may be toxic and (iii) substrates with a scope of further functionalization work equally well. The easy handling and the low cost of the reagents involved in this synthetic methodology offers profound opportunities to expand and explore the use of IDC in organic synthesis. Further applications of this strategy are under active investigation in our laboratory.

## Supporting Information

File 1 Copies of ^1^H, and^13^C NMR spectra for all new compounds.
