# Supplementary material for: Synthesis of 2-oxindoles via 'transition-metal-free' intramolecular dehydrogenative coupling (IDC) of sp2 C–H and sp3 C–H bonds
Source: Beilstein J Org Chem. 2016 Jun 8;12:1153–69. doi: 10.3762/bjoc.12.111 (PMC4979638; doi:10.3762/bjoc.12.111)
Supplement: File 1 — Copies of 1H, and13C NMR spectra for all new compounds. [file Beilstein_J_Org_Chem-12-1153-s001.pdf]

Supporting Information

for

Synthesis of 2-oxindoles via 'transition-metal-free' intramolecular dehydrogenative coupling (IDC) of sp^2 C–H and sp^3 C–H bonds

Nivesh Kumar[‡], Santanu Ghosh[‡], Subhajit Bhunia, and Alakesh Bisai*

Address: Department of Chemistry, Indian Institute of Science Education and Research Bhopal, Bhopal Bypass Road, Bhauri, Bhopal – 462 066, Madhya Pradesh, India

Email: Alakesh Bisai* - alakesh@iiserb.ac.in

*Corresponding author

[§]Both authors contributed equally.

Copies of ^{1}H, and ^{13}C NMR spectra for all new compounds

Table of Contents

General procedure for the synthesis of **3**	S3–S6
General procedure for the synthesis of **29**	S7–S8
General Procedure for alkylations of **9**	S8–S18
Procedure for the synthesis of compound (±)-**5k**	S18–S19
Procedure for one-pot alkylations followed by oxidative coupling (IDC)	S20–S27
General procedure for the synthesis of the β-*N*-arylamide	S27–S28
Synthesis of compound **11a**	S29
General procedure for the synthesis of the β-*N*-arylamido esters **11c**,**d**	S30–S32
General Procedure for Iodine and DBU mediated (IDC)	S33–S34
Experimental procedure for the synthesis of compound **25**	S34–S37
Procedure for the synthesis of **27**	S38–S39
References and notes	S39–S40
Spectral graphics	S41–S126

Materials and methods

Unless otherwise stated, reactions were performed in oven-dried glassware fitted with rubber septa under a nitrogen atmosphere and were stirred with Teflon-coated magnetic stirring bars. Liquid reagents and solvents were transferred via syringe using standard Schlenk techniques. Tetrahydrofuran (THF) and diethyl ether (Et_2O) were distilled over sodium/benzophenone ketyl. Dichloromethane (CH_2Cl_2), toluene, and benzene were distilled over calcium hydride. All other solvents such as DMSO, DMF, dioxane and reagents such as alkyl halides, *N*-methylaniline, *p*-anisidine etc. were used as received, unless otherwise noted. Thin layer chromatography was performed using Merck Silicagel 60 F-254 precoated plates (0.25 mm) and visualized by UV irradiation, anisaldehyde stain and other stains. Silicagel from Merck (particle size 100–200 mesh) was used for flash chromatography. Melting points were recorded on a digital melting point apparatus from Jyoti Scientific (AN ISO 9001:2000) and are uncorrected. ^{1}H and ^{13}C NMR spectra were recorded on Bruker 400, 500 MHz spectrometers with ^{13}C operating frequencies of 100, 125 MHz, respectively. Chemical shifts (δ) are reported in ppm relative to the residual solvent signal (δ = 7.24 for ^{1}H NMR and δ = 77.0 for ^{13}C NMR). Data for ^{1}H NMR spectra are reported as follows: chemical shift (multiplicity, coupling constants, number of hydrogens). Abbreviations are as follows: s (singlet), d (doublet), t (triplet), q (quartet), m (multiplet), br (broad). IR spectra were recorded on a FTIR system (Spectrum BX) from PerkinElmer spectrometer and are reported in frequency of absorption (cm^{-1}). Only selected IR absorbencies are reported.

General procedure for the synthesis of the β-*N*-arylamido esters 3:

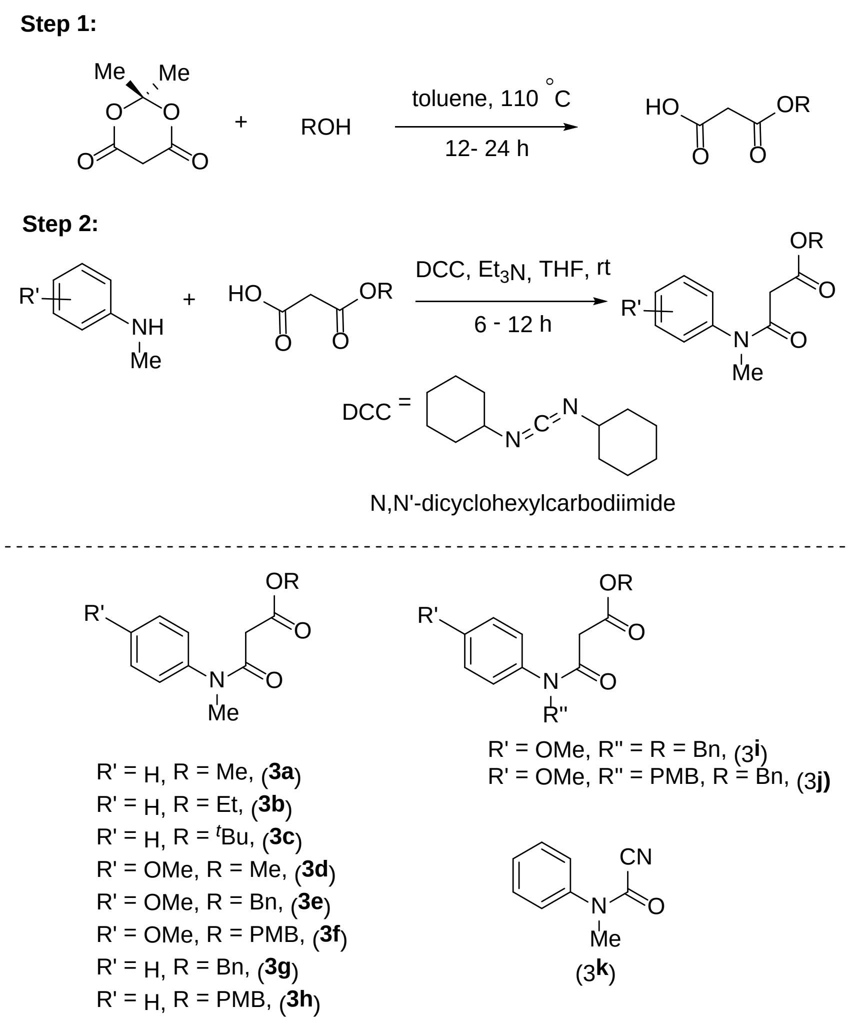

Scheme: Synthesis of β-*N*-arylamido esters.

Step 1:

As described in reference [1], a flame-dried round-bottom flask was charged with Meldrum's acid [1.0 equiv (generally in 10 g scale)] and required alcohol (10.0 equiv). The reaction mixture was heated under refluxed at 110 °C for 6–12 h. Upon completion of the reaction (judged by TLC analysis), the reaction mixture was cooled to room temperature. Then most of the volatile components were evaporated under reduced pressure. The crude malonic acid monoesters were directly used for coupling reaction without purification.

Step 2:

In a flame-dried round-bottom flask, crude malonic acid monoester (1.0 mmol) was taken in THF (5 mL/mmol) and cooled to 0 °C on an ice-bath. To this reaction mixture was added triethylamine (3 mmol). DCC (1.2 mmol) was then added to the mixture at same temperature. After 5 minutes of stirring at same temperature, a solution of *N*-methylaniline derivatives (1.0 mmol) in THF was added drop wise to the reaction mixture and slowly allowed to warm to rt. Then the stirring was continued untill TLC showed complete consumption of starting materials. The reaction mixture was then diluted with dichloromethane (approx. 40 mL) and then successively washed with water (20 mL), 2 N HCl (20 mL), saturated $NaHCO_3$ (20 mL) and finally with brine (20 mL). The organic extracts were dried over anhydrous Na_2SO_4 and concentrated under reduced pressure. The crude mixture was purified through flash chromatography (hexanes/EtOAc mixture as eluent) to afford β-*N*-arylamido esters.

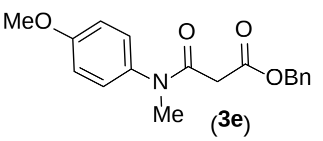

(**3e**)

Benzyl 3-((4-methoxyphenyl) (methyl)amino)-3-oxopropanoate (**3e**): The compound was obtained as brown gel (8.9 g, 49% yield), R_f = 0.42 (40% EtOAc in hexane). **^{1}H NMR** (400 MHz, $CDCl_3$) δ 7.32-7.24 (m, 5H), 7.06-7.01 (m, 2H), 6.82-6.78 (m, 2H), 5.05 (s, 2H), 3.74 (s, 3H), 3.20 (s, 5H); **^{13}C NMR** (100 MHz, $CDCl_3$) δ 167.6, 166.2, 159.2, 136.2, 135.5, 128.5, 128.4, 128.3, 128.3, 115.0, 66.9, 55.5, 41.5, 37.6; **IR** (film) υ_{max} 3026, 2926, 1737, 1652, 1494, 1452, 1380, 1300, 1247, 1156, 1113, 1028, 907, 840, 754, 703 cm^{-1}.

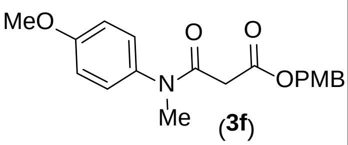

(**3f**)

4-Methoxybenzyl 3-((4-methoxyphenyl)(methyl)amino)-3-oxopropanoate (**3f**): The compound was obtained as brown gel (2.18 g, 52% yield), R_f = 0.40 (50% EtOAc in

hexane). **^{1}H NMR** (400 MHz, $CDCl_3$) δ 7.23 (d, *J* =8.7 Hz, 2H), 7.05-7.03 (m, 2H), 6.87-6.85 (m, 4H), 5.01 (s, 2H), 3.78 (s, 3H), 3.77 (s, 3H), 3.22 (s, 3H), 3.19 (s, 2H); **^{13}C NMR** (100 MHz, $CDCl_3$) δ 167.7, 166.2, 159.7, 159.2, 136.2, 130.3, 128.4, 127.6, 115.0, 113.9, 66.8, 55.5, 55.3, 41.6, 37.6; **IR** (film) υ_{max} 3020, 2924, 2841, 1733, 1650, 1602, 1490, 1452, 1373, 1305, 1245, 1179, 1156, 1115, 1029, 964, 908, 840, 757, 703 cm^{-1}.

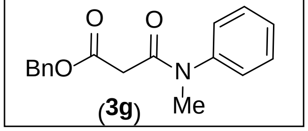

(**3g**)

Benzyl 3-(methyl(phenyl)amino)-3-oxopropanoate (**3g**): The compound was obtained as brown oil (2.2 g, 47% yield), R_f = 0.40 (30% EtOAc in hexane). **^{1}H NMR** (400 MHz, $CDCl_3$) δ 7.39-7.30 (m, 8H), 7.19-7.16 (m, 2H), 5.10 (s, 2H), 3.29 (s, 3H), 3.26 (s, 2H); **^{13}C NMR** (100 MHz, $CDCl_3$) δ 167.6, 165.8, 143.4, 135.5, 129.9, 128.5, 128.4, 128.3, 128.3, 127.2, 67.0, 41.5, 37.5; **IR** (film) υ_{max} 3025, 2923, 2851, 1737, 1652, 1597, 1492, 1451, 1378, 1311, 1238, 1151, 1120, 1027, 907, 838, 756, 703 cm^{-1}. Characterization data of compound **3g** are compatible with the literature data see: reference [3].

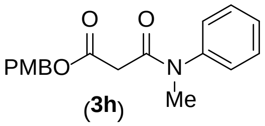

(**3h**)

4-Methoxybenzyl 3-(methyl(phenyl)amino)-3-oxopropanoate (**3h**): The compound was obtained as yellow gel (4.43 g, 50% yield), R_f = 0.40 (40% EtOAc in hexane). **^{1}H NMR** (400 MHz, $CDCl_3$) δ 7.34-7.28 (m, 3H), 7.21 (d, *J* =8.7 Hz, 2H), 7.13-7.11 (m, 2H), 6.83 (d, *J* = 8.7 Hz, 2H), 4.99 (s, 2H), 3.75 (s, 3H), 3.24 (s, 3H), 3.18 (s, 2H); **^{13}C NMR** (100 MHz, $CDCl_3$) δ 167.6, 165.9, 159.7, 143.4, 130.2, 129.9, 128.2, 127.6, 127.2, 113.9, 66.8, 55.3, 41.6, 37.5; **IR** (film) υ_{max} 3025, 2926, 1729, 1600, 1492, 1451, 1373, 1326, 1311, 1177, 1149, 1117, 1028, 968, 904, 824, 757, 703 cm^{-1}. Characterization data of compound **3h** are compatible with the literature data see: reference [3].

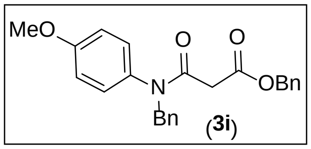

Benzyl 3-(benzyl(4-methoxyphenyl)amino)-3-oxopropanoate (**3i**): The compound was obtained as brown gel (3.12 g, 55% yield), R_f = 0.41 (40% EtOAc in hexane). **^{1}H NMR** (400 MHz, $CDCl_3$) δ 7.36-7.28 (m, 5H), 7.24-7.17 (m, 5H), 6.84-6.81 (m, 2H), 6.73-6.71 (m, 2H), 5.09 (s, 2H), 4.85 (s, 2H), 3.72 (s, 3H), 3.26 (s, 2H); **^{13}C NMR** (100 MHz, $CDCl_3$) δ 167.6, 166.2, 159.3, 137.0, 135.4, 134.3, 129.5 , 128.9, 128.6, 128.4, 128.4, 128.3, 127.4, 114.7, 67.0, 55.4, 53.2, 41.8; **IR** (film) υ_{max} 3026, 2925, 1733, 1651, 1601, 1492, 1452, 1374, 1326, 1179, 1151, 1067, 1028, 907, 836, 757, 703 cm^{-1}.

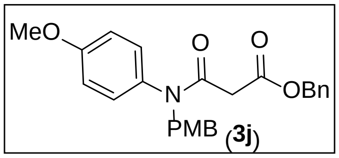

Benzyl 3-((4-methoxybenzyl)(4-methoxyphenyl)amino)-3-oxopropanoate (**3j**): The compound was obtained as brown gel (9.3 g, 51% yield), R_f = 0.30 (30% EtOAc in hexane). **^{1}H NMR** (400 MHz, $CDCl_3$) δ 7.34-7.28 (m, 5H), 7.08 (d, *J* = 8.5 Hz, 2H), 6.81-6.78 (m, 2H), 6.75-6.70 (m, 4H), 5.08 (s, 2H), 4.77 (s, 2H), 3.74 (s, 3H), 3.72 (s, 3H), 3.22 (s, 2H); **^{13}C NMR** (100 MHz, $CDCl_3$) δ 167.6, 166.1, 159.2, 159.0, 135.5, 134.2, 130.2, 129.5, 128.6, 128.4, 128.3, 121.9, 114.7, 113.7, 67.0, 55.4, 55.2, 52.5, 41.8; **IR** (film) υ_{max} 3025, 2924, 1731, 1652, 1598, 1493, 1451, 1397, 1323, 1248, 1176, 1153, 1109, 1028, 1002, 906, 840, 755, 703 cm^{-1}.

For characterization data of compounds **3**(**a–d**), **3k**, **9** see: reference [1].

General procedure for the synthesis of the *N*-methyl-3-oxo-*N*-arylbutanamides 29:

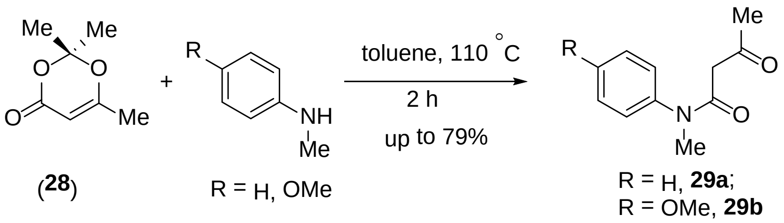

A flame-dried round-bottom flask was charged with 2,2,6-trimethyl-4*H*-1,3-dioxin-4-one (**28**) [1.0 equiv (in 5 g scale)] and *N*-methyl aromatic amine (1.0 equiv) in toluene (100 mL) at room temperature. The reaction mixture was then placed over a 110 °C oil bath for 2 h. Upon completion of the reaction (judged by TLC analysis), the reaction mixture was cooled to room temperature. Most of the volatile components were evaporated under reduced pressure. The crude product was purified by silicagel column chromatography by using EtOAc/hexane as eluents to afford desired products (**29**).

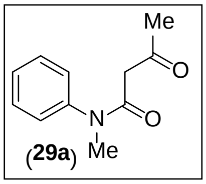

***N*-methyl-3-oxo-*N*-phenylbutanamide** (**29a**): The compound was obtained as reddish oil (5.3 g; 79% yield), R_f = 0.43 (20% EtOAc in hexane). **^{1}H NMR** (400 MHz, $CDCl_3$) δ 7.34-7.39 (m, 2H), 7.29-7.32 (m, 1H),7.14 (m, 2H), 3.23-3.25 [m, 5H (*N*-Me, -CH_2)], 2.04 (s, 3H); **^{13}C NMR** (100 MHz, $CDCl_3$) δ: 202.4, 166.7, 143.6, 129.9, 128.3, 127.2, 49.9, 37.3, 30.3; **IR** (film) υ_{max} 3500, 2926, 1722, 1644, 1595, 1496, 1379, 1124, 1026, 924 cm^{-1}. Experimental data of compound **29a** are compatible with the literature report, see reference [15].

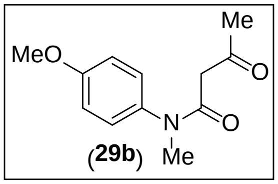

N-(4-methoxyphenyl)-N-methyl-3-oxobutanamide (**29b**): The compound was obtained as yellowish oil (5.6 g; 72% yield), R_f = 0.39 (20% EtOAc in hexane). **^{1}H NMR** (400 MHz, $CDCl_3$) δ 7.02-7.05 (m, 2H), 6.83-6.87 (m, 2H), 3.76 (s, 3H), 3.23 (s, 2H), 3.19 (s, 3H), 2.03 (s, 3H); **^{13}C NMR** (100 MHz, $CDCl_3$) δ 202.3, 167.0, 159.1, 136.2, 128.3, 114.9, 55.4, 49.8, 37.3, 30.2; **IR** (film) υ_{max} 3500, 2936, 1721, 1652, 1513, 1249, 1125, 1030, 924, 840 cm^{-1}. Experimental data of compound **29b** are compatible with the literature report, see reference [16].

General procedure for alkylations of β-amidoester using alkyl halides:

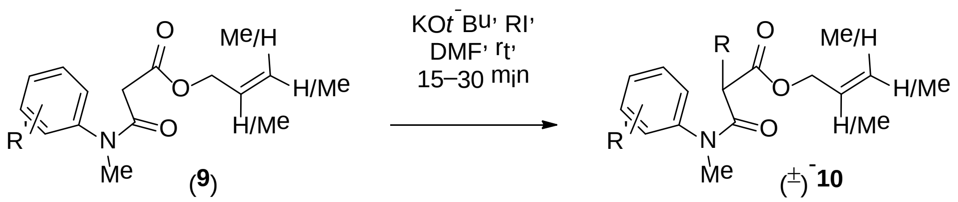

In a flame-dried round-bottom flask, *β*-amidoester (**9**) (1 mmol; 1 equiv) was taken in DMF (5 mL) at room temperature. To this reaction mixture KO*t*-Bu (1.2 mmol; 1.2 equiv) was added. After 5 minutes of stirring at same temperature alkyl halide (1.05 mmol; 1.05 equiv) was added and stirring was continued for 15 minutes. Upon completion of the alkylation (judged by TLC), it was diluted with 10 mL of EtOAc and quenched with water. The organic extracts were dried over anhydrous sodium sulphate and concentrated under vacuum. The crude product was purified by flash chromatography (hexane and EtOAc as eluents) to afford compounds **10**.

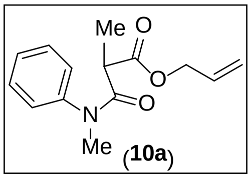

(±)-Allyl 2-methyl-3-(methyl (phenyl)amino)-3-oxopropanoate (**10a**)**:** The compound was obtained as colorless oil (310 mg; 92% yield), R_f = 0.42 (20% EtOAc in hexane). **^{1}H NMR** (500 MHz, $CDCl_3$) δ 7.45 (m, 2H), 7.39 (m, 1H), 7.28 (m, 2H), 5.85-5.93 (m, 1H), 5.32 (dd, J = 15.3, 1.8 Hz, 1H), 5.24 (d, J = 10.5 Hz, 1H), 4.53-4.62 (m, 2H), 3.46 (q, J =

7.1 Hz,1H), 3.33 (s, 3H), 1.33 (d, *J* =7.0 Hz, 3H); **^{13}C NMR** (125 MHz, $CDCl_3$) δ 170.4, 170.0, 143.6, 131.8, 130.0, 128.3, 127.5, 118.2, 65.7, 43.5, 37.7, 14.2; **IR** (film) υ_{max} 2941, 1747, 1661, 1596, 1496, 1386, 1317, 1190, 776 cm^{-1}; **HRMS** (ESI) m/z 248.1299 [(M+H)$^+$; calculated for $[C_{14}H_{18}NO_3]^+$: 248.1281]. Characterization data of compound **10a** are compatible with the literature report, see: reference [5].

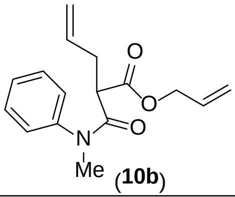

(**10b**)

(±)-Allyl 2-(methyl(phenyl)carbamoyl)pent-4-enoate (**10b**): The compound was obtained as colorless oil (323 mg; 90% yield), R_f = 0.51 (20% EtOAc in hexane). **^{1}H NMR** (400 MHz, $CDCl_3$) δ 7.40 (m, 2H), 7.33 (m, 1H), 7.20 (m, 2H), 5.80-5.89 (m, 1H), 5.57-5.67 (m, 1H), 5.19-5.29 (m, 2H), 4.96-5.03 (m, 2H), 4.49-4.58 (m, 2H), 3.41 (dd, *J* = 8.6, 6.2 Hz, 1H), 3.27 (s, 3H), 2.50-2.66 (m, 2H); **^{13}C NMR** (100 MHz, $CDCl_3$) δ 169.2, 168.4, 143.4, 134.6, 131.8, 129.8, 128.2, 127.8, 118.4, 117.4, 65.7, 48.9, 37.7, 33.5; **IR** (film) υ_{max} 2934, 1745, 1662, 1596, 1496, 1384, 1178, 921, 701 cm^{-1}; **HRMS** (ESI) m/z 274.1455 [(M+H)$^+$; calculated for $[C_{16}H_{20}NO_3]^+$: 274.1438].

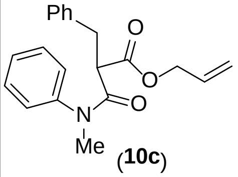

(**10c**)

(±)-Allyl 2-benzyl-3-(methyl(phenyl)amino)-3-oxopropanoate (**10c**): The compound was obtained as colorless oil (420 mg; 94% yield), R_f = 0.55 (20% EtOAc in hexane). **^{1}H NMR** (400 MHz, $CDCl_3$) δ 7.21-7.24 (m, 6H), 7.0-7.02 (m, 2H), 6.06 (br, 1H), 5.82-5.92 (m, 1H), 5.30 (dd, *J* = 17.2, 1.4 Hz, 1H), 5.22 (dd, *J* = 10.5, 1.1 Hz, 1H), 4.53-4.62 (m, 2H), 3.56 (dd, *J* = 10.2, 4.9 Hz, 1H), 3.22 (m, 1H), 3.15 (s, 3H), 3.06 (dd, *J* = 13.4, 4.9 Hz, 1H); **^{13}C NMR** (100 MHz, $CDCl_3$) δ 169.1, 168.4, 143.2, 138.4, 131.8, 129.5, 129.3, 128.4, 128.3, 128.0, 127.6, 126.6, 118.4, 65.8, 51.0, 37.4, 35.3; **IR** (film) υ_{max} 2938,

1747, 1661, 1596, 1496, 1386, 1263, 1153, 991, 701 cm^{-1}; **HRMS** (ESI) m/z 324.1610 $[(M+H)^+$; calculated for $[C_{20}H_{22}NO_3]^+$: 324.1594].

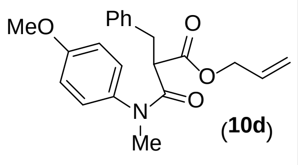

(±)-Allyl 2-benzyl-3-((4-methoxyphenyl)(methyl)amino)-3-oxopropanoate ((±)-10d): The compound was obtained as yellowish oil (109 mg; 81% yield), R_f = 0.29 (20% EtOAc in hexane). **^{1}H NMR** (400 MHz, $CDCl_3$) δ 7.23 (m, 4H), 7.02-7.05 (m, 2H), 6.71 (d, *J* = 7.5 Hz, 2H), 6.51 (br, 1H), 5.81-5.91 (m, 1H), 5.2-5.3 (m, 2H), 4.53-4.62 (m, 2H), 3.77 (s, 3H), 3.58 (dd, *J* = 10.3, 4.8 Hz, 1H), 3.22 (dd, *J* = 13.3, 10.5 Hz, 1H), 3.22 (s, 3H), 3.06 (dd, *J* = 13.4, 4.9 Hz, 1H); **^{13}C NMR** (100 MHz, $CDCl_3$) δ: 169.1, 168.7, 159.0, 138.4, 135.9, 131.8, 129.3, 128.6, 128.3, 126.6, 118.3, 114.6, 65.8, 55.4, 50.8, 37.6, 35.3; **IR** (film) υ_{max} 2937, 1747, 1659, 1513, 1385, 1249, 1169, 701cm^{-1}; **HRMS** (ESI) m/z 354.1720 $[(M+H)^+$; calculated for $[C_{21}H_{24}NO_4]^+$: 354.1700].

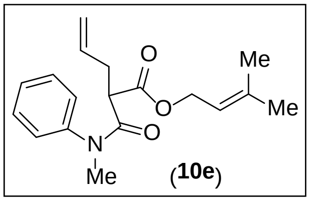

(±)-3-Methylbut-2-en-1-yl 2-(methyl(phenyl)carbamoyl)pent-4-enoate ((±)-10e): The compound was obtained as colorless oil (196 mg; 85% yield), R_f = 0.42 (20% EtOAc in hexane). **^{1}H NMR** (400 MHz, $CDCl_3$) δ 7.38 (m, 2H), 7.33 (m, 1H), 7.20 (m, 2H), 5.57-5.67 (m, 1H), 5.26 (m, 1H), 4.98 (m, 2H), 4.54-4.59 (m, 1H), 4.45-4.5 (m, 1H), 3.36 (dd, *J* = 8.9, 5.9 Hz, 1H), 3.27 (s, 3H), 2.59-2.66 (m, 1H), 2.48-2.55 (m, 1H), 1.74 (s, 3H), 1.66 (s, 3H); **^{13}C NMR** (100 MHz, $CDCl_3$) δ 169.6, 168.4, 143.4, 139.1, 134.7, 129.7, 128.1, 127.9, 118.3, 117.2, 62.1, 49.0, 37.7, 33.6, 20.7, 18.0; **IR** (film) υ_{max} 2923, 1740, 1664, 1381, 1266, 1171, 701cm^{-1}; **HRMS** (ESI) m/z 302.1755 $[(M+H)^+$; calculated for $[C_{18}H_{24}NO_3]^+$: 302.1751].

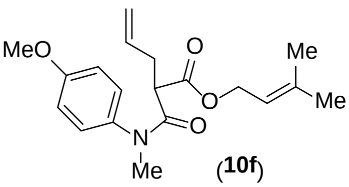

(10f)

(±)-3-Methylbut-2-en-1-yl 2-((4-methoxyphenyl)(methyl)carbamoyl)pent-4-enoate ((±)-10f): The compound was obtained as yellowish oil (220 mg; 88% yield), R_f = 0.34 (20% EtOAc in hexane). **^{1}H NMR** (400 MHz, $CDCl_3$) δ 7.09 (d, *J* = 8.9 Hz, 2H), 6.86 (d, *J* = 9.0 Hz, 2H), 5.57-5.67 (m, 1H), 5.25 (m, 1H), 4.94-5.02 (m, 2H), 4.52-4.57 (m, 1H), 4.44-4.49 (m, 1H), 3.79 (s, 3H), 3.37 (dd, *J* = 8.9, 6.0 Hz, 1H), 3.22 (s, 3H), 2.57-2.65 (m, 1H), 2.46-2.53 (m, 1H), 1.72 (s, 3H), 1.65 (s, 3H); **^{13}C NMR** (100 MHz, $CDCl_3$) δ 169.6, 168.7, 159.1, 139.1, 136.1, 134.8, 128.9, 118.3, 117.2, 114.8, 62.0, 55.5, 48.9, 37.8, 33.5, 25.7, 18.0; **IR** (film) υ_{max} 2935, 1739, 1661, 1532, 1382, 1249, 1172, 839cm^{-1}; **HRMS** (ESI) m/z 332.1875 $[(M+H)]^+$; calculated for $[C_{19}H_{26}NO_4]^+$: 332.1856].

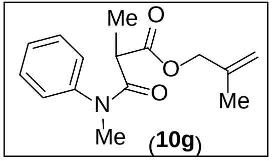

(10g)

(±)-2-Methylallyl 2-methyl-3-(methyl(phenyl)amino)-3-oxopropanoate ((±)-10g): The compound was obtained as colorless oil (190 mg; 90% yield), R_f = 0.33 (20% EtOAc in hexane). **^{1}H NMR** (400 MHz, $CDCl_3$) δ 7.39 (m, 2H), 7.32 (m, 1H), 7.21 (m, 2H), 4.88 (d, *J* = 10.1 Hz, 2H), 4.43 (m, 2H), 3.42 (q, *J* = 7.0 Hz, 1H), 3.26 (s, 3H), 1.68 (s, 3H), 1.28 (d, *J* = 7.0 Hz, 3H); **^{13}C NMR** (100 MHz, $CDCl_3$) δ 170.3, 170.0, 143.6, 139.6, 129.9, 128.2, 127.5, 113.0, 68.2, 43.4, 37.6, 19.4, 14.1; **IR** (film) υ_{max} 2981, 1748, 1662, 1596, 1497, 1458, 1387, 1181, 908cm^{-1}; **HRMS** (ESI) m/z 262.1442 $[(M+H)]^+$; calculated for $[C_{15}H_{20}NO_3]^+$: 262.1438]. Characterization data of compound **10g** are compatible with the literature report, see: reference [5].

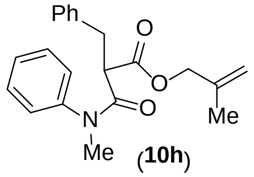

(10h)

(±)-2-Methylallyl 2-benzyl-3-(methyl(phenyl)amino)-3-oxopropanoate ((±)-10h): The compound was obtained as colorless oil (114 mg, 88% yield), R_f = 0.43 (20% EtOAc in hexane). **^{1}H NMR** (400 MHz, $CDCl_3$) δ 7.23 (m, 6H), 7.02 (m, 2H), 6.58 (br, 2H), 4.92 (d, *J* = 9.8 Hz, 2H), 4.50 (s, 2H), 3.57 (dd, *J* = 10.4, 4.9 Hz, 1H), 3.23 (dd, *J* = 13.3, 10.5 Hz, 1H), 3.15 (s, 3H), 3.07 (dd, *J* =13.3, 4.8 Hz, 1H), 1.71 (s, 3H); **^{13}C NMR** (100 MHz, $CDCl_3$) δ: 169.1, 168.4, 143.2, 139.6, 138.4, 129.5, 129.3, 128.4, 128.0, 127.6, 126.6, 113.1, 68.4, 50.9, 37.4, 35.3, 19.5; **IR** (film) υ_{max} 2938, 1747, 1660, 1496, 1385, 1193, 1152, 701cm^{-1}; **HRMS** (ESI) m/z 338.1765 [(M+H)$^+$; calculated for $[C_{21}H_{24}NO_3]^+$: 338.1751].

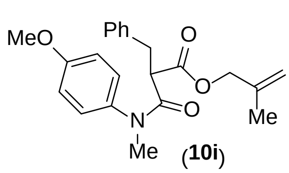

(**10i**)

(±)-2-Methylallyl 2-benzyl-3-((4-methoxyphenyl)(methyl)amino)-3-oxopropanoate ((±)-10i): The compound was isolated as yellowish oil (122 mg; 87% yield), R_f = 0.40 (20% EtOAc in hexane). **^{1}H NMR** (400 MHz, $CDCl_3$) δ 7.20-7.26 (m, 3H), 7.04 (m, 2H), 6.7 (d, *J* =7.4 Hz, 2H), 6.50 (br, 2H), 4.92 (d, *J* = 9.7 Hz, 2H), 4.49 (s, 2H), 3.76 (s, 3H), 3.59 (dd, *J* = 10.5, 4.8 Hz, 1H), 3.23 (dd, *J* = 13.3, 10.5 Hz, 1H), 3.11 (s, 3H), 3.06 (dd, *J* = 13.4, 4.8 Hz, 1H), 1.71 (s, 3H); **^{13}C NMR** (100 MHz, $CDCl_3$) δ 169.2, 168.7, 159.0, 139.6, 138.5, 136.0, 129.3, 128.7, 128.4, 126.6, 114.5, 113.2, 68.4, 55.4, 50.7, 37.6, 35.5, 19.5; **IR** (film) υ_{max} 2938, 1747, 1660, 1514, 1249, 1032, 838, 701 cm^{-1}; **HRMS** (ESI) m/z 368.1876 [M+H]$^+$; calculated for $[C_{22}H_{26}NO_4]^+$: 368.1856].

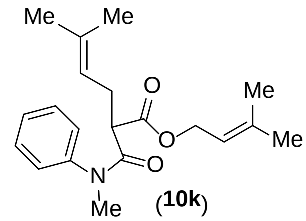

(**10k**)

(±)-3-Methylbut-2-en-1-yl 5-methyl-2-(methyl(phenyl)carbamoyl)hex-4-enoate ((±)-10k): The compound was obtained as colorless oil (205 mg; 81% yield), R_f = 0.41 (20% EtOAc in hexane). **^{1}H NMR** (400 MHz, $CDCl_3$) δ 7.38 (m, 2H), 7.32 (m, 1H), 7.17 (d, *J*

= 7.3 Hz, 2H), 5.27 (t, *J* = 7.1 Hz, 1H), 4.93 (t, *J* = 7.3 Hz, 1H), 4.45-4.59 (m, 2H), 3.31 (dd, *J* = 8.7, 6.3 Hz, 1H), 3.26 (s, 3H), 2.41-2.6 (m, 2H), 1.74 (s, 3H), 1.67 (s, 3H), 1.63 (s, 3H), 1.48 (s, 3H); **^{13}C NMR** (100 MHz, $CDCl_3$) δ 169.1, 168.9, 143.6, 139.0, 134.3, 129.7, 128.0, 127.8, 120.3, 118.4, 62.0, 49.1, 37.7, 28.1, 25.71, 25.72, 18.1, 17.6; **IR** (film) υ_{max} 2930, 1743, 1662, 1596, 1497, 1453, 1380, 1267, 1190, 1153, 961 cm^{-1}; **HRMS** (ESI) m/z 330.2073 $[(M+H)^+]$; calculated for $[C_{20}H_{28}NO_3]^+$: 330.2064].

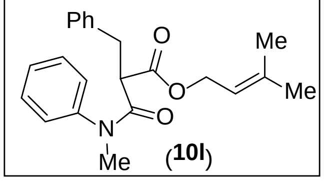

(10l)

(±)-3-Methylbut-2-en-1-yl 2-benzyl-3-(methyl(phenyl)amino)-3-oxopropanoate ((±)-10l): The compound was obtained as colorless oil (120 mg; 89% yield), R_f = 0.45 (20% EtOAc in hexane). **^{1}H NMR** (400 MHz, $CDCl_3$) δ 7.21-7.24 (m, 6H), 7.01 (m, 2H), 6.62 (br, 2H), 5.29 (t, *J* = 7.2 Hz, 1H), 4.50-4.64 (m, 2H), 3.52 (dd, *J* = 10.3, 4.9 Hz, 1H), 3.21 (dd, *J* = 13.3, 10.5 Hz, 1H), 3.15 (s, 3H), 3.05 (dd, *J* = 13.4, 4.9 Hz, 1H), 1.75 (s, 3H), 1.68 (s, 3H); **^{13}C NMR** (100 MHz, $CDCl_3$) δ 169.4, 168.5, 143.2, 139.2, 138.5, 129.5, 129.2, 128.3, 127.9, 127.6, 126.5, 118.3, 62.2, 51.1, 37.4, 35.3, 25.7, 18.1; **IR** (film) υ_{max} 2934, 1742, 1662, 1596, 1496, 1456, 1382, 1264, 1162, 756 cm^{-1}; **HRMS** (ESI) m/z 374.1753 $[(M+Na)^+]$; calculated for $[C_{22}H_{25}NO_3 + Na]^+$: 374.1727].

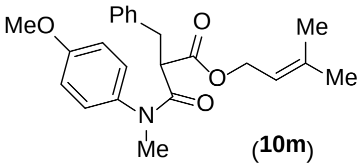

(10m)

(±)-3-Methylbut-2-en-1-yl 2-benzyl-3-((4-methoxyphenyl) (methyl)amino)-3-oxopropanoate ((±)-10m): The compound was isolated as yellowish oil (129 mg; 88% yield), R_f = 0.37 (20% EtOAc in hexane). **^{1}H NMR** (400 MHz, $CDCl_3$) δ 7.21-7.25 (m, 4H), 7.03-7.05 (m, 2H), 6.70 (d, *J* = 7.9 Hz, 2H), 6.51 (br, 1H), 5.26-5.30 (tt, *J* = 7.2, 1.2 Hz, 1H), 4.49-4.62 (m, 2H), 3.77 (s, 3H), 3.53 (dd, *J* = 10.4, 4.8 Hz, 1H), 3.20 (dd, *J* = 13.3, 10.5 Hz, 1H), 3.11 (s, 3H), 3.04 (dd, *J* = 13.4, 4.8 Hz, 1H), 1.74 (s, 3H), 1.68 (s,

3H); ^{13}**C NMR** (100 MHz, $CDCl_3$) δ 169.5, 168.7, 158.9, 139.1, 138.6, 136.0, 129.3, 128.7, 128.3, 126.5, 118.4, 114.5, 62.2, 55.4, 50.9, 37.6, 35.3, 25.8, 18.1; **IR** (film) υ_{max} 2940, 1741, 1660, 1513, 1456, 1382, 1248, 838, 756 cm^{-1}; **HRMS** (ESI) m/z 404.1839 $[(M+Na)^+$; calculated for $[C_{23}H_{27}NO_4 + Na]^+$: 404.1832].

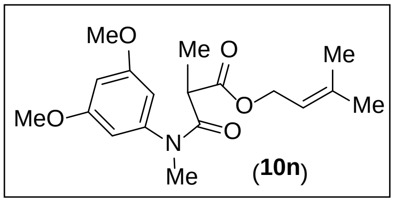

(±)-3-Methylbut-2-en-1-yl 3-((3,5-dimethoxyphenyl)(methyl)amino)-2-methyl-3-oxopropanoate ((±)-10n): Isolated compound was yellowish in color (110 mg; 87% yield), R_f = 0.37 (30% EtOAc in hexane). ^{1}**H NMR** (400 MHz, $CDCl_3$) δ 6.41 (m, 1H), 6.37 (m, 2H), 5.27 (t, *J* = 7.1 Hz, 1H), 4.46-4.63 (m, 2H), 3.76 (s, 6H), 3.48 (q, *J* = 7.0 Hz, 1H), 3.25 (s, 3H), 1.72 (s, 3H), 1.66 (s, 3H), 1.29 (d, *J* = 7.0 Hz, 3H); ^{13}**C NMR** (100 MHz, $CDCl_3$) δ 170.9, 170.0, 161.5, 145.3, 139.0, 118.4, 105.7, 110.0, 62.1, 55.5, 43.5, 37.4, 25.7, 18.0, 14.4; **IR** (film) υ_{max} 2940, 1743, 1662, 1606, 1456, 1350, 1206, 1158, 1061, 928 cm^{-1}; **HRMS** (ESI) m/z 336.1825 $[(M+H)^+$; calculated for $[C_{18}H_{26}NO_5]^+$: 336.1805].

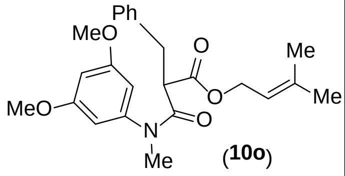

(±)-3-Methylbut-2-en-1-yl 2-benzyl-3-((3,5-dimethoxyphenyl)(methyl)amino)-3-oxopropanoate ((±)-10o): The compound was isolated as yellowish oil (134 mg; 85% yield), R_f = 0.40 (30% EtOAc in hexane). ^{1}**H NMR** (400 MHz, $CDCl_3$) δ: 7.19-7.24 (m, 4H), 7.06 (m, 2H), 6.32 (t, *J* = 2.2 Hz, 1H), 5.83 (br, 1H), 5.30 (tt, *J* = 7.2, 1.3 Hz, 1H), 4.66 (dd, *J* = 12.4, 7.5 Hz, 1H), 4.53 (dd, *J* = 12.2, 7.3 Hz, 1H), 3.66 (m, 1H), 3.65 (s, 6H), 3.2-3.27 (dd, *J* = 13.3, 10.8 Hz, 1H), 3.12 (s , 3H), 3.04-3.09 (dd, *J* = 13.6, 4.6 Hz, 1H), 1.73 (s, 3H), 1.68 (s, 3H); ^{13}**C NMR** (100 MHz, $CDCl_3$) δ 169.5, 168.3, 161.1, 144.9, 139.3, 138.6, 129.3, 128.3, 126.5, 118.3, 105.5, 100.4, 62.2, 55.4, 51.0, 37.2, 35.4,

25.7, 18.0; **IR** (film) υ_{max} 2963, 1741, 1661, 1392, 1206, 1157, 1065, 701 cm^{-1}; **HRMS** (ESI) m/z 412.2125 [(M+H)$^+$; calculated for [$C_{24}H_{30}NO_5$]$^+$: 412.2118].

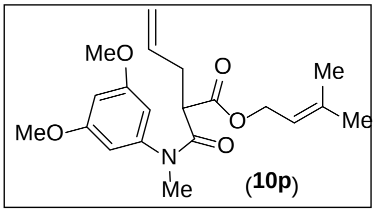

(±)-3-Methylbut-2-en-1-yl 2-((3,5-dimethoxyphenyl)(methyl)carbamoyl)pent-4-enoate ((±)-10p): The compound was isolated as yellowish oil (106 mg; 79% yield), R_f = 0.38 (30% EtOAc in hexane). **^{1}H NMR** (400 MHz, $CDCl_3$) δ 6.41 (t, *J* = 2.0 Hz, 1H), 6.34 (d, *J* = 1.9 Hz, 2H), 5.59-5.69 (m, 1H), 5.27 (t, *J* = 7.2 Hz, 1H), 5.05 (dd, *J* = 17.1, 1.0 Hz, 1H), 4.97 (d, *J* = 10.1 Hz, 1H), 4.46-4.62 (m, 2H), 3.75 (s, 6H), 3.48 (dd, *J* = 9.2, 5.7 Hz, 1H), 3.24 (s, 3H), 2.49- 2.68 (m, 2H), 1.72 (s, 3H), 1.66 (s, 3H); **^{13}C NMR** (100 MHz, $CDCl_3$) δ 169.7, 168.3, 161.4, 145.1, 139.2, 134.8, 118.3, 117.3, 106.0, 100.1, 62.2, 55.4, 48.9, 37.4, 33.7, 25.7, 18.0; **IR** (film) υ_{max} 2967, 1741, 1663, 1603, 1594, 1207, 1157, 1068, 701cm^{-1}; **HRMS** (ESI) m/z 362.1978 [(M+H)$^+$; calculated for [$C_{20}H_{28}NO_5$]$^+$: 362.1962].

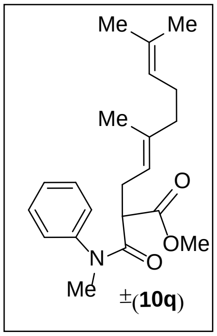

(±)-Methyl 5,9-dimethyl-2-(methyl(phenyl)carbamoyl)deca-4,8-dienoate ((±)-10q): The compound was obtained as colorless oil (286 mg; 86% yield), R_f = 0.6 (30% EtOAc in hexane). **^{1}H NMR** (400 MHz, $CDCl_3$) δ 7.39 (m, 2H), 7.32 (m, 1H), 7.19 (m, 2H), 5.04 (t, *J* = 6.6 Hz, 1H), 4.94 (t, *J* = 7.2 Hz, 1H), 3.64 (s, 3H), 3.34 (dd, *J* = 8.6, 6.5 Hz, 1H), 3.27 (s, 3H), 2.57 (m, 1H), 2.48 (m, 1H), 1.92 (m, 4H), 1.64 (s, 3H), 1.56 (s, 3H), 1.48 (s, 3H); **^{13}C NMR** (100 MHz, $CDCl_3$) δ 170.3, 168.9, 143.6, 138.1, 131.5, 129.7,

128.1, 127.7, 124.1, 119.9, 52.2, 48.9, 39.7, 37.6, 28.0, 26.6, 25.7, 17.7, 15.9; **IR** (film) υ_{max} 2924, 1748, 1662, 1436, 1383, 1266, 1199, 1116, 701cm^{-1}; **HRMS** (ESI) m/z 344.2232 [(M+H)$^+$; calculated for [$C_{21}H_{30}NO_3$]$^+$: 344.2220].

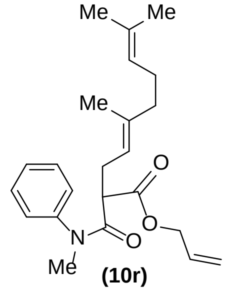

(10r)

(±)-Allyl 5,9-dimethyl-2-(methyl(phenyl)carbamoyl)deca-4,8-dienoate ((±)-10r): The compound was obtained as colorless oil (260 mg; 82% yield), R$_f$ = 0.71 (30% EtOAc in hexane). **^{1}H NMR** (400 MHz, $CDCl_3$) δ 7.39 (m, 2H), 7.32 (m, 1H), 7.19 (m, 2H), 5.85 (m, 1H), 5.25 (dd, *J* = 17.2, 1.2 Hz, 1H), 5.21 (dd, *J* = 10.5, 1.0 Hz, 1H), 5.04 (t, *J* = 6.6 Hz, 1H), 4.95 (t, *J* = 7.0 Hz, 1H), 4.54 (m, 2H), 3.37 (dd, *J* = 8.7, 6.3 Hz, 1H), 3.27 (s, 3H), 2.6 (m, 1H), 2.49 (m, 1H), 1.95 (m, 4H), 1.64 (s, 3H), 1.57 (s, 3H), 1.49 (s, 3H); **^{13}C NMR** (100 MHz, $CDCl_3$) δ 169.5, 168.8, 143.6, 138.1, 131.9, 131.5, 129.7, 128.1, 127.8, 124.1, 120.0, 118.2, 65.6, 49.0, 39.7, 37.7, 28.0, 26.6, 25.7, 17.7, 16.0; **IR** (film) υ_{max} 2927, 1747, 1662, 1497, 1382, 1268, 1155, 701cm^{-1}; **HRMS** (ESI) m/z 370.2395 [(M+H)$^+$; calculated for [$C_{23}H_{32}NO_3$]$^+$: 370.2377].

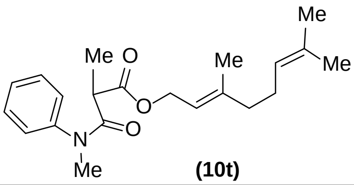

(10t)

(±)-3,7-Dimethylocta-2,6-dien-1-yl 2-methyl-3-(methyl(phenyl)amino)-3-oxopropanoate ((±)-10t): The compound was obtained as yellowish oil (179 mg; 86% yield), R$_f$ = 0.37 (20% EtOAc in hexane). **^{1}H NMR** (400 MHz, $CDCl_3$) δ 7.37 (m, 2H), 7.31 (m, 1H), 7.2 (m, 2H), 5.24 (t, *J* = 6.8 Hz, 1H), 5.04 (t, *J* = 5.5 Hz, 1H), 4.55-4.59 (m, 1H), 4.44-4.49 (m, 1H), 3.36 (q, *J* = 7.1 Hz, 1H), 3.25 (s, 3H), 1.98-2.06 (m, 4H), 1.63 (s, 6H), 1.56 (s, 3H), 1.26 (d, *J* = 7.0 Hz, 3H); **^{13}C NMR** (100 MHz, $CDCl_3$) δ 170.7, 170.0,

143.6, 142.3, 131.8, 129.8, 128.2, 127.5, 123.7, 118.1, 62.0, 43.6, 39.5, 37.6, 26.3, 25.7, 17.7, 16.4, 14.2; **IR** (film) υ_{max} 2933, 1744, 1668, 1497, 1456, 1385, 1189, 701cm^{-1}; **HRMS** (ESI) m/z 344.2214 [(M+H)$^+$; calculated for [$C_{21}H_{30}NO_3$]$^+$: 344.2220].

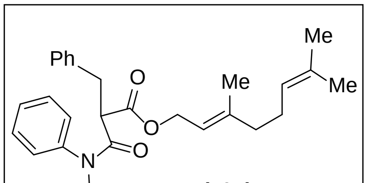

(10u)

(±)-3,7-Dimethylocta-2,6-dien-1-yl 2-benzyl-3-(methyl(phenyl)amino)-3-oxopropanoate ((±)-10u): The compound was isolated as yellowish oil (214 mg; 84% yield), R_f = 0.57 (20% EtOAc in hexane). **^{1}H NMR** (400 MHz, $CDCl_3$) δ 7.19-7.22 (m, 6H), 6.99-7.01 (m, 2H), 6.59 (br, 2H), 5.28 (t, J = 7.0 Hz, 1H), 5.06 (t, J = 5.4 Hz, 1H), 4.59-4.64 (m, 1H), 4.5-4.55 (m, 1H), 3.49-3.53 (m,1H), 3.17-3.23 (m, 1H), 3.12 (s, 3H), 3.03 (dd, J = 13.3, 4.8 Hz, 1H), 2.02-2.08 (m, 4H), 1.66 (s, 3H), 1.64 (s, 3H), 1.57 (s, 3H); **^{13}C NMR** (100 MHz, $CDCl_3$) δ 169.5, 168.4, 143.2, 142.5, 138.5, 131.8, 129.5, 129.2, 128.3, 127.9, 127.6, 126.5, 123.7, 118.0, 62.1, 51.0, 39.5, 37.4, 35.3, 26.3, 25.7, 17.7, 16.5; **IR** (film) υ_{max} 2934, 1744, 1662, 1496, 1384, 1194, 701cm^{-1}; **HRMS** (ESI) m/z 420.2534 [(M+H)$^+$; calculated for [$C_{27}H_{34}NO_3$]$^+$: 420.2533].

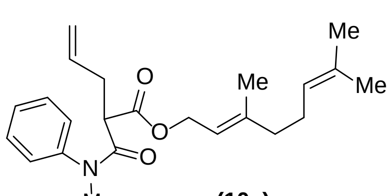

(10v)

(±)-3,7-Dimethylocta-2,6-dien-1-yl 2-(methyl(phenyl)carbamoyl)pent-4-enoate ((±)-10v): The isolated compound was yellow oil (184 mg; 82% yield), R_f = 0.51 (20% EtOAc in hexane). **^{1}H NMR** (400 MHz, $CDCl_3$) δ 7.40 (m, 2H), 7.34 (m, 1H), 7.22 (m, 2H), 5.59-5.7 (m, 1H), 5.29 (t, J = 7.0 Hz, 1H), 5.08 (m, 1H), 4.97-5.05 (m, 2H), 4.59-4.64 (m, 1H), 4.49-4.54 (m, 1H), 3.40 (dd, J = 8.9, 5.9 Hz, 1H), 3.29 (s, 3H), 2.61-2.69 (m, 1H), 2.5-2.57 (m, 1H), 2.04-2.11 (m, 4H), 1.68 (s, 3H), 1.67 (s, 3H), 1.6 (s, 3H); **^{13}C NMR** (100 MHz, $CDCl_3$) δ 169.5, 168.4, 143.4, 142.5, 134.7, 131.8, 129.7, 128.1, 127.9, 123.7,

118.0, 117.3, 62.1, 49.0, 39.5, 37.7, 33.5, 26.3, 25.7, 17.7, 16.5; **IR** (film) υ_{max} 2924, 1740, 1667, 1496, 1383, 1177, 1117, 701cm^{-1}; **HRMS** (ESI) m/z 370.2379 $[(M+H)^+$; calculated for $[C_{23}H_{32}NO_3]^+$: 370.2377].

For characterization data of compound **10j** see: reference [1].

Procedure for the synthesis of compound (±)-**5k**:

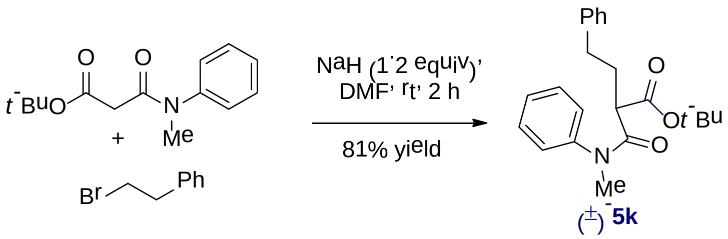

In a flame-dried round-bottom flask, β-amido-*tert*-butylester (1 mmol; 1 equiv) was taken in DMF (5 mL) at room temperature. To this reaction mixture NaH (1.2 mmol; 1.2 equiv) was added. After 5 minutes of stirring at same temperature (1.05 mmol; 1.05 equiv) of phenethyl bromide was added to the reaction mixture. Then the reaction mixture was stirred for 2 h. Upon completion of the alkylation (judged by TLC), it was diluted with 10 mL of EtOAc and quenched with water. The organic extracts were dried over $MgSO_4$ and concentrated under vacuum. The crude product was purified by flash chromatography (hexane and EtOAc as eluents) to afford compounds (±)-**5k**.

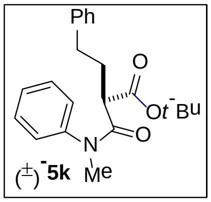

(±)-*tert*-Butyl 2-(methyl(phenyl)carbamoyl)-4-phenylbutanoate ((±)-5k): 82% yield, yellowish oil, R_f = 0.51 (20% EtOAc in hexane). **^{1}H NMR** (400 MHz, $CDCl_3$) δ: 7.33 (m, 3H), 7.20 (m, 2H), 7.12 (m, 3H), 7.05 (m, 2H), 3.28 (s, 3H), 3.25 (t, J = 7.32 Hz, 1H), 2.51 (m, 2H), 2.12 (m, 2H), 1.41 (s, 9H); **^{13}C NMR** (100 MHz, $CDCl_3$) δ: 169.2, 169.1, 143.5, 141.2, 129.7, 128.4, 128.3, 128.0, 127.6, 125.9, 81.4, 49.4, 37.6, 33.5, 30.9, 28.0; **IR** (film) υ_{max} 2977, 1738, 1662, 1496, 1369, 1254, 1148, 700 cm^{-1}.

Characterization data of compound (**5k**) are compatible with the literature data see: reference [17].

Procedure for the synthesis of compound **5v** and **7g** are similar as for **5k**.

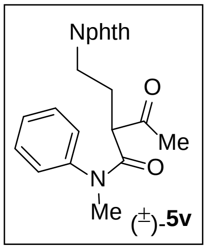

(±)-2-Acetyl-4-(1,3-dioxoisoindolin-2-yl)-*N*-methyl-*N*-phenylbutanamide (±)-**5v**: The compound was obtained as yellowish oil (236 mg; 67% yield), $R_f = 0.35$ (30% EtOAc in hexane). **^{1}H NMR** (400 MHz, $CDCl_3$) δ 7.69-7.75 (m, 4H), 7.07 (m, 4H), 6.78-6.82 (m, 1H), 3.48-3.63 (m, 2H), 3.45 (dd, *J* = 8.6, 5.0 Hz, 1H), 3.19 (s, 3H), 2.18-2.28 (m, 1H), 2.03 (s, 3H), 1.85-1.93 (m, 1H); **^{13}C NMR** (100 MHz, $CDCl_3$) δ 202.5, 168.9, 168.2, 143.1, 134.0, 132.0, 129.8, 127.9, 127.3, 123.2, 54.1, 37.5, 36.1, 28.7, 27.7; **IR** (film) υ_{max} 3443, 2929, 1770, 1713, 1652, 1595, 1496, 1394, 1168, 1118, 1025, 903 cm^{-1}.

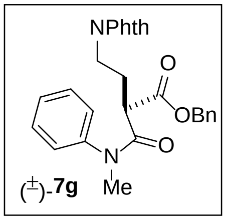

(±)-Benzyl-4-(1,3-dioxoisoindolin-2-yl)-2-(methyl(phenyl)carbamoyl)butanoate ((±)-7g): The compound was obtained as colorless gel (530 mg, 44% yield), R_f = 0.35 (40% EtOAc in hexane). **^{1}H NMR** (400 MHz, $CDCl_3$) δ 7.77-7.69 (m, 4H), 7.35-7.30 (m, 5H), 7.05-7.02 (m, 4H), 6.92-6.88 (m, 1H), 5.16-5.09 (m, 2H), 3.70-3.64 (m, 2H), 3.35 (dd, *J* = 5.9, 2.2 Hz, 1H), 3.19 (s, 3H), 2.33-2.24 (m, 1H), 2.15-2.06 (m, 1H); **^{13}C NMR** (100 MHz, $CDCl_3$) δ 169.2, 168.2, 168.1, 143.1, 135.7, 133.8, 132.0, 129.6, 128.5, 128.2, 128.1, 127.8, 127.4, 123.2, 67.0, 46.7, 37.5, 36.0, 28.3; **IR** (film) υ_{max} 3025, 2922, 1731, 1716, 1650, 1601, 1492, 1452, 1374, 1180, 1028, 907, 756, 703 cm^{-1}.

Procedure for the one-step alkylations followed by oxidative coupling (IDC):

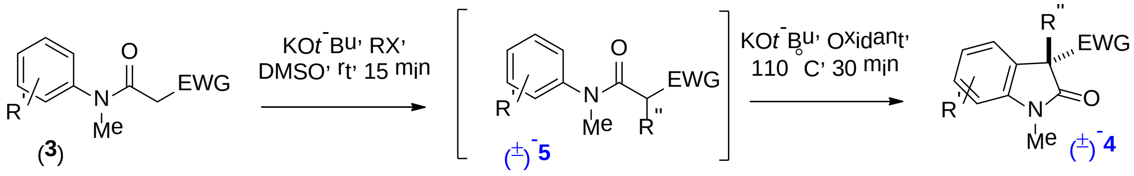

As described in reference [1]; in a flame-dried round-bottom flask was charged with β-amidoester **3** (0.25 mmol; 1 equiv) in DMSO (0.75 mL) at room temperature. KO*t*-Bu (0.30 mmol; 1.2 equiv) in one portion was added to the reaction mixture. After 1–2 minutes, alkyl halide (0.275 mmol, 1.1 equiv) was added and stirring was continued for 5–10 minutes (upon completion of the starting material judged by TLC analysis), KO*t*-Bu (0.30 mmol; 1.2 equiv) and 1.2 equiv (0.30 mmol) of oxidant were added at room temperature. Immediately afterwards, the reaction mixture was placed over an oil bath maintaining temperature 110 °C for 30–40 minutes. Upon completion of the oxidative coupling, the reaction mixture was cooled to room temperature and diluted with 5 mL of EtOAc. The reaction mixture was quenched with 5 mL saturated sodium thiosulfate. The organic layer was then successively washed with water (5 mL), and brine (5 mL). The organic extracts were dried over anhydrous Na_2SO_4 and concentrated under reduce pressure. The crude product was purified by flash chromatography using hexane and EtOAc mixture as eluents to afford the 2-oxindole derivatives.

General procedure for KO^{t}Bu/I_2- or NIS-mediated IDC of C-alkylated compounds: In a flame-dried round-bottom flask, β-amidoester (1.0 mmol; 1 equiv) was taken in DMSO (8 mL) at room temperature. To this reaction mixture KO*t*-Bu (1.2 mmol; 1.2 equiv) was added in one portion. After 1-2 minutes of stirring at same temperature, (1.2 equiv., 1.2 mmol) of I_2 was added to the reaction mixture at same temperature. Immediately afterwards, the reaction mixture was placed over oil bath maintaining temperature 80 °C for 15–30 minutes. Upon completion of the reaction (judged by TLC analysis), reaction mixture was cooled to room temperature and diluted with 10 mL of EtOAcand quenched with 10 mL saturated sodium thiosulfate solution. The organic layer was separated and successively washed with water (10 mL), and brine (10 mL). The

organic extracts were dried over anhydrous $MgSO_4$ and concentrated under vacuum. The crude product was purified by flash chromatography (hexane and EtOAc as eluents) to afford 2-oxindoles derivatives.

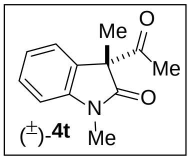

(±)-3-Acetyl-1,3-dimethylindolin-2-one (**4t**): The compound was obtained as yellowish oil (164 mg; 46% yield using conditions A), R_f = 0.44 (20% EtOAc in hexane). **^{1}H NMR** (400 MHz, $CDCl_3$) δ 7.33 (td, *J* = 7.7, 1.3 Hz, 1H), 7.13 (d, *J* = 6.5 Hz, 1H), 7.07 (td, *J* = 7.4, 0.5 Hz, 1H), 6.89 (d, *J* = 7.8 Hz, 1H), 3.28 (s, 3H), 1.94 (s, 3H), 1.55 (s, 3H); **^{13}C NMR** (100 MHz, $CDCl_3$) δ 201.0, 175.9, 143.7, 129.4, 129.1, 123.6, 123.3, 108.6, 62.0, 26.6, 25.9, 19.0; **IR** (film) υ_{max} 2932, 1730, 1714, 1610, 1494, 1471, 1374, 1347, 1304, 1262, 1198, 1120, 1104, 1030, 900 cm^{-1}. Characterization data of compound (**4t**) are compatible with the literature data see: **reference** [**2**].

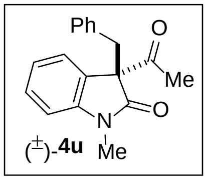

(±)-3-Acetyl-3-benzyl-1-methylindolin-2-one (**4u**): The product was obtained as a yellowish gel (92 mg; 46% yield using conditions A), R_f = 0.49 (20% EtOAc in hexane). **^{1}H NMR** (400 MHz, $CDCl_3$) δ 7.22-7.25 (m, 2H), 7.07 (t, *J* = 7.5 Hz, 1H), 6.95-7.01 (m, 3H), 6.77 (d, *J* = 6.5 Hz, 2H), 6.60 (d, *J* = 8.3 Hz, 1H), 3.44 (s, 2H), 2.97 (s, 3H), 2.0 (s, 3H); **^{13}C NMR** (100 MHz, $CDCl_3$) δ 201.0, 174.4, 144.2, 135.0, 129.7, 129.2, 127.5, 126.7, 126.6, 124.2, 122.9, 108.3, 68.0, 39.0, 26.9, 26.2; **IR** (film) υ_{max} 2932, 1730, 1714, 1610, 1494, 1471, 1374, 1347, 1262, 1198, 1119, 1030, 964, 901 cm^{-1}; **HRMS** (ESI) m/z 280.1360 $[(M+H)^+$; calculated for $[C_{18}H_{17}NO_2]^+$: 280.1332]. Characterization data of compound **4u** are compatible with the literature data see: reference [2].

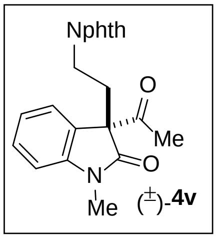

(±)-2-(2-(3-Acetyl-1-methyl-2-oxoindolin-3-yl)ethyl)isoindoline-1,3-dione (**4v**): The compound was obtained as yellowish oil (50 mg, 42% yield using conditions A), R_f = 0.52 (50% EtOAc in hexane). **^{1}H NMR** (400 MHz, $CDCl_3$) δ 7.59-7.65 (m, 4H), 7.01-7.05 (m, 2H), 6.74-6.78 (m, 2H), 3.57-3.64 (m, 1H), 3.46-3.52 (m, 1H), 3.25 (s, 3H), 2.78-2.85 (m, 1H), 2.57-2.63 (m, 1H), 1.90 (s, 3H); **^{13}C NMR** (100 MHz, $CDCl_3$) δ 199.8, 174.2, 167.7, 144.3, 133.6, 131.8, 139.0, 126.3, 123.4, 123.1, 122.9, 108.9, 65.1, 33.9, 29.6, 26.7, 25.6; **IR** (film) υ_{max} 2928, 1771, 1714, 1609, 1492, 1470, 1397, 1373, 1188, 1124 cm^{-1}; **HRMS** (ESI) m/z 363. 1368 $[(M+H)^+$; calculated for $[C_{21}H_{318}N_2O_4]^+$: 363.1339].

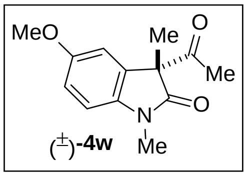

(±)-3-Acetyl-5-methoxy-1,3-dimethylindolin-2-one (**4w**): The compound was obtained as yellowish oil (47 mg; 47% yield using conditions A), R_f = 0.41 (20% EtOAc in hexane). **^{1}H NMR** (400 MHz, $CDCl_3$) δ 6.80 (dd, *J* = 8.5, 2.5 Hz, 1H), 6.74 (d, *J* = 8.4 Hz, 1H), 6.68 (d, *J* = 2.4 Hz, 1H), 3.71 (s, 3H), 3.20 (s, 3H), 1.90 (s, 3H), 1.49 (s, 3H); **^{13}C NMR** (100 MHz, $CDCl_3$) δ 201.1, 175.6, 156.5, 137.1, 130.6, 113.7, 110.6, 109.0, 62.4, 55.8, 26.7, 25.9, 19.0; **IR** (film) υ_{max} 2934, 1727, 1704, 1600, 1499, 1354, 1290, 1208, 1112, 1038 cm^{-1}; **HRMS** (ESI) m/z 234.1106 $[(M+H)^+$; calculated for $[C_{13}H_{15}NO_3]^+$: 234.1125].

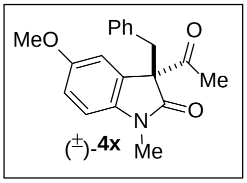

(±)-3-Acetyl-3-benzyl-5-methoxy-1-methylindolin-2-one (**4x**): The compound was obtained as yellow gel (58 mg; 42%yield), R_f = 0.37 (20% EtOAc in hexane). **^{1}H NMR** (400 MHz, $CDCl_3$) δ 6.96-7.02 (m, 3H), 6.79-6.83 (m, 3H), 6.74-6.77 (dd, *J* = 8.5, 2.5 Hz, 1H), 6.51 (d, *J* = 8.5 Hz, 1H), 3.78 (s, 3H), 3.42 (s, 2H), 2.95 (s, 3H), 2.01 (s, 3H); **^{13}C NMR** (100 MHz, $CDCl_3$) δ 201.1, 174.1, 156.1, 137.7, 135.0, 129.8, 127.9, 127.6, 126.6, 113.9, 111.2, 108.7, 68.3, 55.9, 39.0, 26.9, 26.3; **IR** (film) υ_{max} 3423, 1723, 1704, 1648, 1498, 1358, 1289, 1248, 1032, 808, 752 cm^{-1}; **HRMS** (ESI) m/z 310.1432 $[(M+H)^+]$; calculated for $[C_{19}H_{19}NO_3]^+$: 310.1438].

For the characterization of compounds **4a–s**, and **8a–v** see: reference [1].

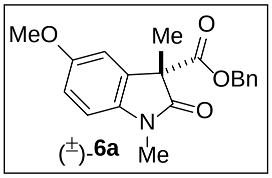

(±)-Benzyl 5-methoxy-1,3-dimethyl-2-oxoindoline-3-carboxylate (**6a**) [**4**]:
The compound was obtained as brown gel (499 mg, 72% yield in conditions A), R_f = 0.34 (30% EtOAc in hexane). **^{1}H NMR** (400 MHz, $CDCl_3$) δ 7.26-7.24 (m, 2H), 7.13-7.11 (m, 2H), 6.85-6.77 (m, 3H), 6.74 (d, *J* = 8.4 Hz, 1H), 5.09 (s, 2H), 3.72 (s, 3H), 3.20 (s, 3H), 1.65 (s, 3H); **^{13}C NMR** (100 MHz, $CDCl_3$) δ 174.7, 169.5, 156.2, 137.1, 135.5, 131.1, 128.4, 128.0, 127.4, 113.7, 110.2, 108.9, 67.1, 55.8, 55.5, 26.6, 20.0; **IR** (film) υ_{max} 2922, 1715, 1649, 1601, 1492, 1448, 1374, 1286, 1246, 1177, 1111, 1030, 964, 907, 814, 754, 703 cm^{-1}. Characterization data of compound **6a** are compatible with the literature data see: reference [4].

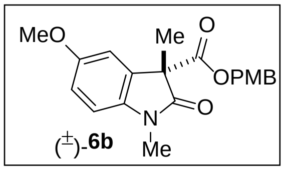

(±)-4-Methoxybenzyl 5-methoxy-1,3-dimethyl-2-oxoindoline-3-carboxylate (**6b**): The compound was obtained as brown gel (290 mg, 84% yield in conditions A), R_f = 0.30 (30% EtOAc in hexane). **^{1}H NMR** (400 MHz, $CDCl_3$) δ 7.08 (d, *J* = 8.6 Hz, 2H), 6.82-6.79 (m, 2H), 6.77-6.75 (m, 2H), 6.73 (d, *J* = 8.5 Hz, 1H), 5.02 (s, 2H), 3.76 (s, 3H), 3.72

(s, 3H), 3.19 (s, 3H), 1.63 (s, 3H); **^{13}C NMR** (100 MHz, $CDCl_3$) δ 174.7, 169.5, 159.5, 156.2, 137.1, 131.1, 129.4, 127.6, 113.8, 113.6, 110.2, 108.8, 67.1, 55.8, 55.6, 55.2, 26.6, 20.1; **IR** (film) υ_{max} 3025, 2926, 1731, 1716, 1601, 1493, 1452, 1374, 1286, 1247, 1177, 1111, 1066, 1029, 965, 907, 822, 757, 703 cm^{-1}.

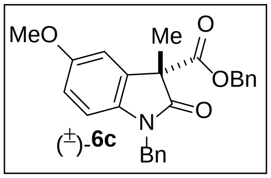

(±)-Benzyl 1-benzyl-5-methoxy-3-methyl-2-oxoindoline-3-carboxylate (**6c**): The compound was obtained as brown gel (277 mg, 62% yield in conditions B), R_f **=** 0.41 (30% EtOAc in hexane). **^{1}H NMR** (400 MHz, $CDCl_3$) δ 7.30-7.26 (m, 3H), 7.20-7.10 (m, 7H), 6.82 (d, *J* = 2.5 Hz, 1H), 6.69 (dd, *J* = 8.6, 2.6 Hz, 1H), 6.55 (d, *J* = 8.6 Hz, 1H), 5.18-5.07 (m, 3H), 4.66 (d, *J* = 16.1 Hz, 1H), 3.70 (s, 3H), 1.72 (s, 3H); **^{13}C NMR** (100 MHz, $CDCl_3$) δ 175.0, 169.6, 156.2, 136.0, 135.4, 135.2, 131.1, 128.7, 128.5, 128.2, 127.7, 127.5, 127.0, 115.4, 113.7, 110.1, 67.5, 55.8, 55.6, 43.9, 20.0; **IR** (film) υ_{max} 3020, 2930, 2845, 1730, 1716, 1600, 1492, 1448, 1375, 1345, 1247, 1179, 1112, 1030, 962, 907, 820, 703 cm^{-1}.

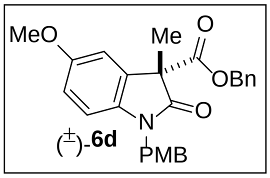

(±)-Benzyl-5-methoxy-1-(4-methoxybenzyl)-3-methyl-2-oxoindoline-3-carboxylate (**6d**): The compound was obtained as brown gel (292 mg, 49% yield in conditions A), R_f **=** 0.21 (20% EtOAc in hexane). **^{1}H NMR** (400 MHz, $CDCl_3$) δ 7.28-7.26 (m, 3H), 7.15-7.13 (m, 2H), 7.10 (d, *J* = 8.6 Hz, 2H), 6.81(d, *J* = 2.5 Hz, 1H), 6.69 (dd, *J* = 8.6, 2.5 Hz, 1H), 6.62 (d, *J* = 8.7 Hz, 2H), 6.57 (d, *J* = 8.6 Hz, 1H), 5.16 (d, *J* = 12.6 Hz, 1H), 5.07 (dd, *J* = 15.6, 7.8 Hz, 2H), 4.57 (d, *J* = 12.6 Hz, 1H), 3.70 (m, 6H), 1.72 (s, 3H); **^{13}C NMR** (100 MHz, $CDCl_3$) δ 174.9, 169.6, 158.9, 156.1, 136.1, 135.3, 131.1, 128.5, 128.3, 128.1, 127.8, 127.4, 114.1, 113.7, 110.1, 110.0, 67.5, 55.8, 55.6, 55.2, 43.3, 19.9; **IR**

(film) υ_{max} 3025, 2928, 2845, 1733, 1716, 1602, 1491, 1448, 1375, 1247, 1179, 1030, 962, 907, 820, 756, 703 cm^{-1}.

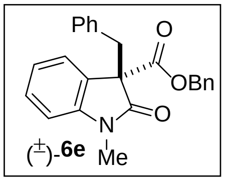

(±)-Benzyl 3-benzyl-1-methyl-2-oxoindoline-3-carboxylate (**6e**): The compound was obtained as yellow gel (212 mg, 48% yield in conditions A), R_f **=** 0.35 (20% EtOAc in hexane). **^{1}H NMR** (400 MHz, $CDCl_3$) δ 7.29-7.24 (m, 4H), 7.22-7.20 (m, 1H), 7.18-7.15 (m, 2H), 7.04-6.96 (m, 4H), 6.83 (d, *J* = 6.7 Hz, 2H), 6.56 (d, *J* = 7.8 Hz, 1H), 5.16 (dd, *J* = 12.6, 4.7 Hz, 2H), 3.59-3.51 (m, 2H), 2.93 (s, 3H); **^{13}C NMR** (100 MHz, $CDCl_3$) δ 173.3, 169.0, 144.1, 135.5, 134.4, 130.0, 129.1, 128.5, 128.1, 127.6, 127.5, 127.2, 126.8, 124.0, 122.5, 108.2, 67.3, 61.0, 39.9, 26.2; **IR** (film) υ_{max} 3025, 2923, 2850 1732, 1714, 1652, 1601, 1491, 1452, 1373, 1352, 1220, 1066, 1028, 1000, 907, 755, 703 cm^{-1}. Characterization data of compound **6e** are compatible with the literature data see; **reference** [**3**].

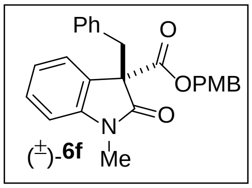

(±)-4-Methoxybenzyl 3-benzyl-1-methyl-2-oxoindoline-3-carboxylate (**6f**): The compound was obtained as yellow gel (350 mg, 59% yield in conditions A), R_f **=** 0.25 (20% EtOAc in hexane). **^{1}H NMR** (400 MHz, $CDCl_3$) δ 7.24-7.20 (m, 2H), 7.18 (dd, *J* = 7.75, 1.07 Hz, 1H), 7.12 (d, *J* = 8.6 Hz, 2H), 7.03 (d, *J* = 3.4 Hz, 1H), 7.01-6.99 (m, 2H), 6.98-6.96 (m, 1H), 6.82-6.80 (m, 3H), 6.54 (d, *J* = 7.8 Hz, 1H), 5.09 (dd, *J* = 12.2, 3.7 Hz, 2H), 3.77 (s, 3H), 3.57-3.49 (m, 2H), 2.92 (s, 3H); **^{13}C NMR** (100 MHz, $CDCl_3$) δ 173.3, 169.1, 159.5, 144.1, 134.4, 130.0, 129.5, 129.0, 127.57, 127.55, 127.2, 126.7, 124.0, 122.4, 113.9, 108.2, 67.2, 61.0, 55.3, 40.0, 26.1; **IR** (film) υ_{max} 3025, 2921, 2846 1732, 1710, 1602, 1515, 1492, 1453, 1373, 1353, 1302, 1246, 1176, 1086, 1029, 1000, 907, 825, 754, 704 cm^{-1}.

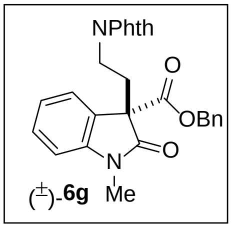

(±)-Benzyl-3-(2-(1,3-dioxoisoindolin-2-yl)ethyl)-1-methyl-2-oxoindoline-3-carboxylate (**6g**): The compound was obtained as yellow gel (268 mg, 54% yield in conditions A), R_f = 0.30 (40% EtOAc in hexane). **^{1}H NMR** (400 MHz, $CDCl_3$) δ 7.64-7.58 (m, 4H), 7.36-7.30 (m, 1H), 7.23-7.21 (m, 2H), 7.07-7.04 (m, 3H), 7.00 (t, *J* = 7.8 Hz, 1H), 6.71 (t, *J* = 7.0 Hz, 2H), 5.09-5.02 (m, 2H), 3.70-3.64 (m, 1H), 3.56-3.49 (m, 1H), 3.20 (s, 3H), 2.93-2.86 (m, 1H), 2.72-2.66 (m, 1H); **^{13}C NMR** (100 MHz, $CDCl_3$) δ 173.2, 168.6, 167.7, 144.2, 135.3, 133.9, 133.6, 131.8, 128.9, 128.4, 128.1, 127.3, 123.2, 122.9, 122.6, 108.8, 62.8, 58.1, 33.7, 30.4, 26.6; **IR** (film) υ_{max} 3025, 2928, 2847, 1716, 1602, 1492, 1452, 1395, 1374, 1215, 1153, 1070, 1028, 908, 756, 700 cm^{-1}.

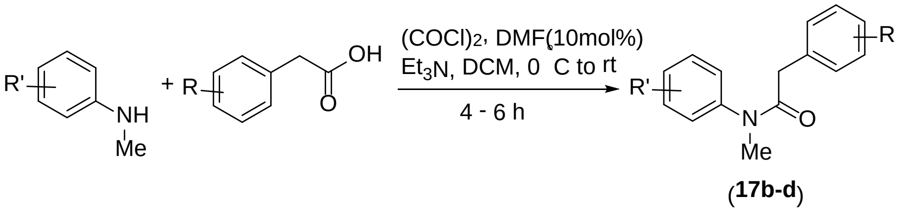

Scheme: Synthesis of β-*N*-arylamide.

General procedure for the synthesis of the β-*N*-arylamide: As described in reference [1], in a flame-dried round-bottom flask was charged with aromatic acid (1.0 mmol; 1.0 equiv) in dichloromethane (5 mL) at 0 °C. To this solution was added oxalyl chloride (1.2 mmol; 1.2 equiv) via a syringe in drop wise. After 5 minutes of stirring at same temperature, DMF (10 mmol; 10 equiv) added drop wise to the reaction mixture and allowed to stir at same temperature for 3 h. Then all the volatile components were evaporated under reduced pressure. Next, *N*-methylaniline derivatives (0.95 mmol; 0.95 equiv) was taken in another flame-dried round-bottom flask, with dichloromethane (4 mL) and cooled to 0 °C, to above reaction mixture triethylamine (3.0 mmol; 3.0 equiv) was added. The earlier reaction mixture was taken in dicholomethane, and added to

above *N*-methylaniline solution in drop wise via a syringe over 5 minutes at 0 ºC. The reaction mixture was allowed to warm to rt (over 10 minutes) and stirring was continued for additional 3 h (TLC showed complete consumption of starting material). The reaction mixture was diluted with dichloromethane (40 mL) and then successively washed with water (20 mL), 2 N HCl (20 mL), saturated $NaHCO_3$ (20 mL) and finally with brine (20 mL). The organic extracts were dried over anhydrous Na_2SO_4 and concentrated under vacuum. The crude product was purified by flash chromatography (4:1 hexanes/EtOAc) to afford β-*N*-arylamido esters.

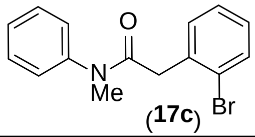

(**17c**)

2-(2-Bromophenyl)-*N*-methyl-*N*-phenylacetamide (17c): The compound was obtained as yellow gel (1.74 g; 69% yield), R_f **=** 0.39 (20% EtOAc in hexane). **^{1}H NMR** (500 MHz, $CDCl_3$) δ 7.49 (d, *J* = 7.9 Hz, 1H), 7.43-7.46 (m, 2H), 7.33-7.39 (m, 2H), 7.26 (m, 4H), 3.58 (s, 2H), 3.34 (s, 3H); **^{13}C NMR** (100 MHz, $CDCl_3$) δ 169.9, 143.8, 135.8, 132.5, 131.5, 129.9, 128.8, 128.4, 128.0, 127.2, 124.8, 41.7, 37.6; **IR** (film) υ_{max} 3059, 2936, 1660, 1596, 1495, 1379, 1264, 1121, 1026, 749 cm^{-1}.

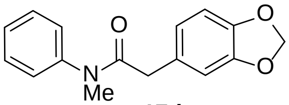

(**17d**)

2-(Benzo[*d*][1,3]dioxol-5-yl)-*N*-methyl-*N*-phenylacetamide (**17d**): The compound was obtained as yellow gel, (2.32 g; 60% yield), R_f = 0.32 (30% EtOAc in hexane). **^{1}H NMR** (400 MHz, $CDCl_3$) δ 7.40-7.30 (m, 3H), 7.11(d, *J* = 7.3 Hz, 2H), 6.64-6.60 (m, 2H), 6.41 (d, *J* = 7.8 Hz, 1H), 5.87 (s, 2H), 3.33 (s, 2H), 3.24 (s, 3H); **^{13}C NMR** (100 MHz, $CDCl_3$) δ 170.8, 159.5, 144.0, 136.9, 129.7, 129.2, 127.3, 127.6, 121.4, 114.5, 112.4, 55.1, 40.9, 37.6; **IR** (film) υ_{max} 3025, 2923, 1600, 1486, 1451, 1371, 1313, 1258, 1153, 1119, 1049, 906, 876, 761, 703 cm^{-1}.

For characterization data of compound **17b** see reference [1].

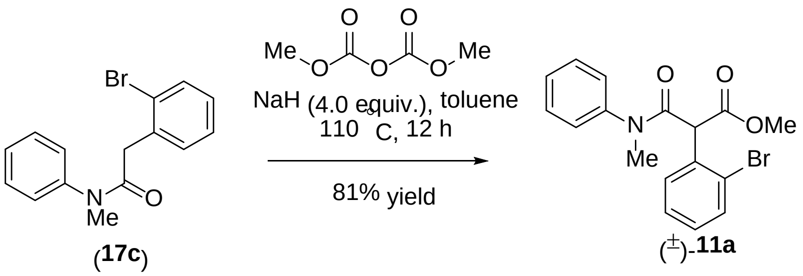

Synthesis of compound (11a): As described in reference [1], in an oven-dried round-bottom flask was charged with (524 mg, 13.14 mmol; 4.0 equiv.) of NaH (60% suspension in mineral oil) in toluene (10 mL) and cooled to 0 ºC in an ice-bath. After 15 minutes of stirring, a solution of amide **17c** (1.0 g; 3.28 mmol; 1.0 equiv) and dimethylcarbonate (1.1 mL, 13.14 mmol; 4.0 equiv) in 10 mL of toluene was added to the reaction mixture dropwise at same temperature and slowly warm the reaction mixture to rt. The reaction mixture was then placed over an oil-bath maintaining temperature at 110 ºC and stirring was continued for 12 h. Upon completion of the reaction (judged by TLC analysis) the reaction was cooled to 0 °C and 1.5 mL glacial acetic acid was added drop wise to the reaction mixture and stirring was continued for 1 h. Then, the reaction was filtered through celite-bed and the filtrate was evaporated under vacuum to dryness. The crude product was purified by flash chromatography (2:1 hexanes/EtOAc) to affordcompound (±)-**11a** in 81% yields.

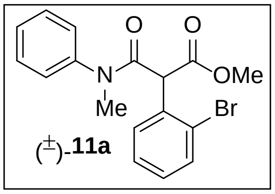

(±)-Methyl 2-(2-bromophenyl)-3-(methyl(phenyl)amino)-3-oxopropanoate ((±)-11a): The compound was isolated as yellowish gel (960 mg; 82% yield), R_f = 0.35 (20% EtOAc in hexane). **^{1}H NMR** (400 MHz, $CDCl_3$) δ 7.62 (dd, *J* = 7.8, 1.5 Hz, 1H), 7.37 (dd, *J* = 8.0, 0.9 Hz, 1H), 7.28-7.33 (m, 4H), 7.11 (td, *J* = 7.9, 1.5 Hz, 1H), 7.0 (brs, 2H), 5.11 (s, 1H), 3.68 (s, 3H), 3.27 (s, 3H); **^{13}C NMR** (100 MHz, $CDCl_3$) δ 168.8, 167.4, 142.7, 133.6, 132.4, 130.8, 130.0, 129.4, 128.4, 127.6, 127.5, 125.0, 55.6, 52.8, 37.9; **IR** (film) υ_{max} 3059, 2962, 1750, 1661, 1597, 1382, 1169, 1119, 1025, 749 cm^{-1}.

For the characterization data of compound **11b** see: **reference** [**1**].

General procedure for the synthesis of the β-*N*-arylamido esters 11c–d:

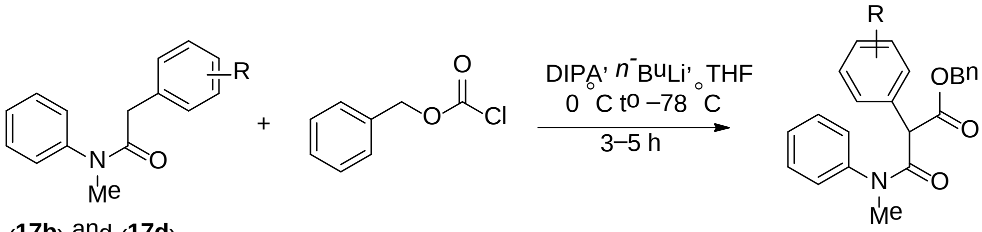

(**17b**) and (**17d**) **11c–d**

In a flame-dried round bottom flask was charged with diisopropylamine (15.6 mmol; 1.2 equiv) in dry THF (4 mL/mmol) and cooled to 0 ºC on an ice-bath. To this solution was added *n*-BuLi (18.2 mmol; 1.4 equiv) via a syringe. After 20 minutes of stirring at same temperature (pale yellowish solution was observed.). Then the reaction mixture was brought to −78 ºC and stirred for 20 minutes. After that a solution of the β-*N*-arylamide (13.0 mmol; 1.0 equiv) in THF was added drop wise to the reaction mixture. After 1.0 h stirring at same temperature benzyl chloroformate (19.5 mmol; 1.5 equiv) was slowly added by syringe to the reaction mixture at −78 ºC and stirring was continued for 3.0 h. Upon completion of the reaction (judged by TLC analysis) the reaction mixture was diluted with ethyl acetate (20 mL) and then successively washed the organic layer with saturated NH_4Cl (20 mL), water (20 mL), and finally with brine (20 mL). The organic extracts were dried over anhydrous $MgSO_4$ and concentrated under vacuum. The crude product was purified by flash chromatography (40% EtOAc/hexanes) to afford β-*N*-arylamido esters.

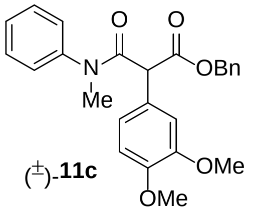

(±)-**11c**

(±)-Benzyl 2-(3,4-dimethoxyphenyl)-3-(methyl(phenyl)amino)-3-oxopropanoate (11c): The compound was obtained as yellow gel (1.76 g; 74% yield), R_f = 0.30 (40%

EtOAc in hexane). ^{1}**H NMR** (400 MHz, $CDCl_3$) δ 7.34-7.27 (m, 8H), 7.04 (br, 2H), 6.76 (d, *J* = 1.8 Hz, 1H), 6.72 (d, *J* = 8.31 Hz, 1H), 6.57 (dd, *J* = 8.3 Hz, 1.8 Hz, 1H), 5.19 (d, *J* = 12.4 Hz, 1H), 5.09 (d, *J* = 12.4 Hz, 1H), 4.56 (s, 1H), 3.83 (s, 3H), 3.76 (s, 3H), 3.24 (s, 3H); ^{13}**C NMR** (100 MHz, $CDCl_3$) δ 168.9, 167.9, 148.8, 148.7, 143.3, 135.74, 129.8, 128.5, 128.3, 128.2, 128.1, 127.9, 125.9, 121.9, 112.6, 110.8, 67.1, 55.9, 56.0, 55.2, 37.8; **IR** (film) υ_{max} 3020, 2924, 1748, 1652, 1599, 1514, 1490, 1452, 1373, 1301, 1264, 1154, 1117, 1027, 1002, 908, 756, 705 cm^{-1}.

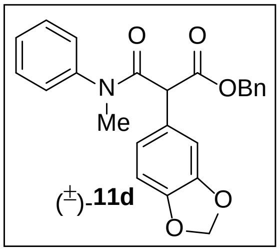

(±)-Benzyl 2-(benzo[*d*][1,3]dioxol-5-yl)-3-(methyl(phenyl)amino)-3-oxopropanoate (**11d**): The compound was obtained as yellow gel (2.61 g; 59% yield), R_f = 0.33 (30% EtOAc in hexane). ^{1}**H NMR** (400 MHz, $CDCl_3$) δ 7.29-7.21 (m, 8H), 6.99 (br, 2H), 6.77 (d, *J* = 1.1 Hz, 1H), 6.58 (d, *J* = 8.0 Hz, 1H), 6.35 (dd, *J* = 8.1 Hz, 1.1 Hz, 1H), 5.83 (s, 2H), 5.07 (q, *J* = 12.93 Hz, 2H), 4.47 (s, 1H), 3.18 (s, 3H); ^{13}**C NMR** (100 MHz, $CDCl_3$) δ 168.8, 167.7, 147.6, 147.4, 143.3, 135.7, 129.8, 128.5, 128.4, 128.2, 128.1, 127.8, 127.1, 123.0, 110.0, 107.9, 101.1, 67.2, 55.1, 37.8; **IR** (film) υ_{max} 3026, 2922, 1748, 1652, 1598, 1490, 1451, 1376, 1299, 1248, 1153, 1115, 1038, 934, 908, 804, 755, 704 cm^{-1}.

Procedure for the synthesis of compound **12** from **11** same as the general procedure for the oxidative coupling

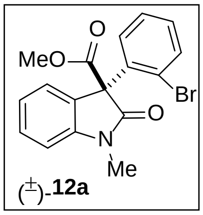

(±)-Methyl 3-(2-bromophenyl)-1-methyl-2-oxoindoline-3-carboxylate ((±)-12a): The compound was obtained as yellow gel (506 mg; 64% yield in case of conditions **A**), R_f = 0.35 (20% EtOAc in hexane). **^{1}H NMR** (400 MHz, $CDCl_3$) δ 7.16 (dd, *J* = 7.4, 1.6 Hz, 1H), 7.36-7.42 (m, 2H), 7.16 (td, *J* = 7.0, 1.7 Hz, 2H), 7.10 (td, *J* = 7.5, 0.7 Hz, 1H), 6.93 (dd, *J* = 7.2, 2.0 Hz, 1H), 6.88 (d, *J* = 7.8 Hz, 1H), 3.78 (s, 3H), 3.24 (s, 3H); **^{13}C NMR** (100 MHz, $CDCl_3$) δ 171.7, 168.7, 144.2, 136.2, 135.2, 130.2, 129.9, 129.7, 127.6, 127.4, 127.3, 126.0, 124.1, 123.2, 108.7, 53.6, 26.8; **IR** (film) υ_{max} 3064, 2963, 1724, 1654, 1471, 1371, 1227, 1024 cm^{-1}.

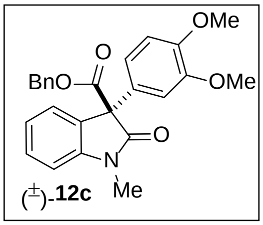

(±)-Benzyl 3-(3,4-dimethoxyphenyl)-1-methyl-2-oxoindoline-3-carboxylate (**12c**): The compound was obtained as yellow gel (319 mg; 61% yield in case of conditions **A**), R_f = 0.27 (30% EtOAc in hexane). **^{1}H NMR** (400 MHz, $CDCl_3$) δ 7.41-7.37 (m, 2H), 7.26-7.24 (m, 3H), 7.18-7.15 (m, 2H), 7.12 (t, *J* = 7.9 Hz, 1H), 6.97 (s, 1H), 6.90 (d, *J* = 7.8 Hz, 1H), 6.73 (d, *J* = 0.9 Hz, 2H), 5.17 (q, *J* = 12.5 Hz, 2H), 3.81 (s, 3H), 3.73 (s, 3H), 3.20 (s, 3H); **^{13}C NMR** (100 MHz, $CDCl_3$) δ 172.9, 169.0, 149.1, 149.0, 144.4, 135.4, 129.6, 128.4, 128.2, 127.9, 127.8, 126.7, 126.1, 122.8, 120.0, 111.6, 110.7, 108.7, 67.5, 63.4, 55.9 (2C), 26.7; **IR** (film) υ_{max} 3026, 2924, 1735, 1603, 1513, 1492, 1453, 1371, 1345, 1259, 1218, 1146, 1088, 1027, 977, 907, 805, 756, 703 cm^{-1}.

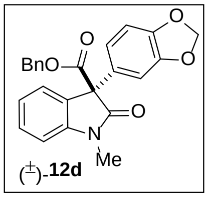

(±)-Benzyl 3-(benzo[*d*][1,3]dioxol-5-yl)-1-methyl-2-oxoindoline-3-carboxylate (**12d**): The compound was obtained as brown gel (110 mg; 63% yield in case of conditions **A**), R_f = 0.45 (30% EtOAc in hexane). **^{1}H NMR** (400 MHz, $CDCl_3$) δ 7.38-7.34 (m, 3H), 7.27-7.24 (m, 4H), 7.15-713 (m, 2H), 6.89-6.87 (m, 2H), 6.70 (s, 1H), 5.90-5.88 (m, 2H),

5.16 (s, 2H), 3.19 (s, 3H); **^{13}C NMR** (100 MHz, $CDCl_3$) δ 172.8, 169.5, 169.0, 147.8, 147.7, 144.3, 135.3, 129.7, 128.5, 128.4, 128.2, 127.6, 126.8, 126.0, 122.9, 121.4, 108.9, 108.7, 108.1, 101.3, 68.5, 67.6, 26.7; **IR** (film) υ_{max} 3025, 2850, 1733, 1716, 1652, 1601, 1491, 1451, 1372, 1246, 1029, 1029, 932, 907, 810, 756, 703 cm^{-1}.

For the characterization data of compound **12b** see reference [1].

General procedure for iodine and DBU mediated intramolecular oxidative coupling (IDC) of compounds (±)-**8**:

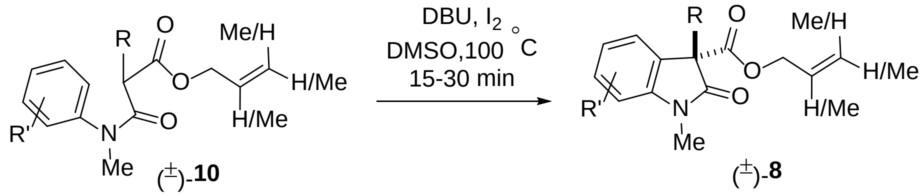

(±)-**10** (±)-**8**

As described in reference [1], in a flame-dried round-bottom flask, β-amidoester (±)**-10** (0.25 mmol; 1 equiv) was taken in DMSO (5 mL) at room temperature. To this reaction mixture DBU (0.38 mmol; 1.5 equiv) was added in one portion. After 1–2 minutes of stirring at same temperature, (1.2 equiv., 0.30 mmol) of I_2 was added to the reaction mixture at same temperature. Immediately afterwards, the reaction mixture was heated at 80 °C for 15–30 minutes. Upon completion of the oxidative coupling (judged by TLC analysis), it was cooled to room temperature and diluted with 5 mL of EtOAc. The reaction mixture was quenched with 5 mL of saturated sodium thiosulphate solution and then the separated organic layer was successively washed with water (5 mL), and brine (5 mL). The organic extracts were dried over anhydrous $MgSO_4$ and concentrated under vacuum. The crude product was purified by flash chromatography (hexane and EtOAc as eluents) to afford 2-oxindoles (±)**-8**.

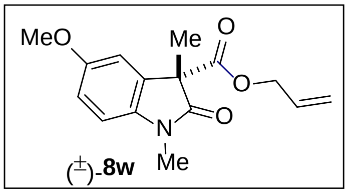

(±)-Allyl 5-methoxy-1,3-dimethyl-2-oxoindoline-3-carboxylate ((±)-8w): Compound was obtained as yellow gel (1.85 g; 71% yield), R_f = 0.44 (20% EtOAc in hexane). **^{1}H NMR** (400 MHz, $CDCl_3$) δ 6.83 (m, 1H), 6.80 (dd, *J* = 8.5, 2.2 Hz, 1H), 6.72 (d, *J* = 8.4 Hz, 1H), 5.70-5.79 (m, 1H), 5.12 (d, *J* = 1.7 Hz, 1H), 5.09 (br, 1H), 4.49-4.58 (m, 2H), 3.75 (s, 3H), 3.19 (s, 3H), 1.63 (s, 3H); **^{13}C NMR** (100 MHz, $CDCl_3$) δ 174.7, 169.4, 156.2, 137.1, 131.4, 131.2, 118.0, 113.4, 110.4, 108.8, 66.0, 55.8, 55.4, 26.6, 20.2; **IR** (film) υ_{max} 2919, 2347, 1744, 1701, 1498, 1432, 1358, 1231, 1110, 1040, 810 cm^{-1}.

For the characterization data of compounds **8a–v** see reference [1].

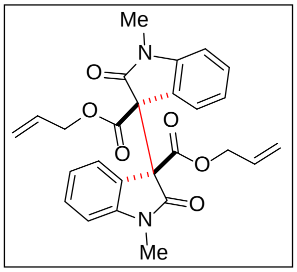

trans ±(**19c**)
Major diastereomer

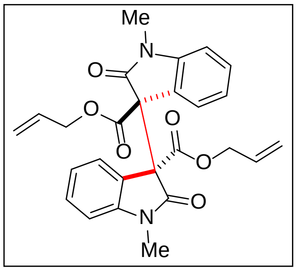

meso-(**19c**)
Minor diastereomer

(±)-Diallyl 1,1'-dimethyl-2,2'-dioxo-[3,3'-biindoline]-3,3'-dicarboxylate ((±)-19c): 40% yield, dr = (1.5:1.0), R_f = 0.41 (60% EtOAc in hexane), colorless solid. **^{1}H NMR** (400 MHz, $CDCl_3$) δ (major diastereomer) 7.33 (m, 2H), 7.20 (t, *J* =7.7 Hz, 2H), 6.89 (m, 2H), 6.56 (d, *J* = 7.7 Hz, 2H), 5.77-5.85 (m, 2H), 5.08-5.19 (m, 4H), 4.54-4.72 (m, 4H), 3.11 (s, 6H); **^{1}H NMR** (400 MHz, $CDCl_3$) δ (minor diastereomer) 7.33 (m, 4H), 6.90 (m, 2H), 6.78 (d, *J* = 7.8 Hz, 2H), 5.77-5.85 (m, 2H), 5.08-5.19 (m, 4H), 4.54-4.72 (m, 4H), 3.05 (s, 6H); **^{13}C NMR** (100 MHz, $CDCl_3$) δ 170.2, 166.8, 166.1, 144.7, 144.1, 131.5, 131.4, 130.1, 129.7, 126.2, 126.1, 123.6, 123.1, 122.1, 122.1, 118.3, 118.1, 108.1, 107.8, 66.7, 66.6, 61.8, 26.5, 26.4; **IR** (film) υ_{max} 2936, 1744, 1719, 1609, 1492, 1472, 1371, 1350, 1227, 1088, 755 cm^{-1}; **HRMS** (ESI) m/z 461.1716 $[(M + H)]^+$; calculated for $[C_{24}H_{28}N_2O_6+H]^+$: 461.1707; **MP** 121-123 ºC.

The characterization data of compound **19c** are compatible with the literature data see: reference [6].

Experimental procedure for the synthesis of compound 25:

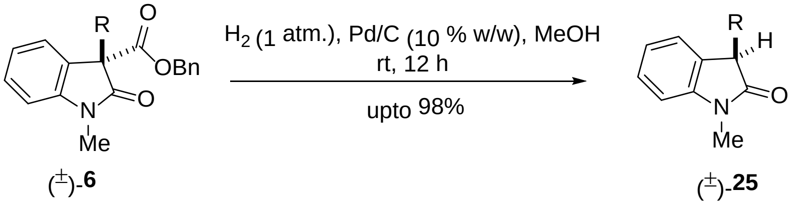

Experimental procedure for the synthesis of compound (**25**): In a flame-dried round-bottom flask was charged with 2-oxindole having a benzylester unit at C3 position (**6**) (1.0 mmol; 1.0 equiv) in MeOH (15 mL) at room temperature. The above solution was purged with N_2 gas over 15 minutes. Later, Pd/C (10% w/w) was added and H_2 gas was purged through the solution and stirring was continued for 12 h at 1 atmospheric pressure of hydrogen. Upon completation of reaction, judge by TLC, the crude product was purified by flash chromatography (hexane and EtOAc mixture as eluents) to afford 3-substiuted 2-oxindoles **25**.

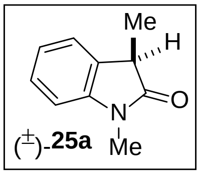

(±)-1,3-Dimethylindolin-2-one (**25a**): The product obtained as colorless solid (21 mg, 97% yield), R_f = 0.55 (30% EtOAc in hexane). **^{1}H NMR** (400 MHz, $CDCl_3$) δ 7.27-7.20 (m, 2H), 7.05-7.01 (m, 1H), 6.80 (d, *J* = 7.7 Hz, 1H), 3.41 (q, *J* = 7.7 Hz, 1H), 3.18 (s, 3H), 1.46 (d, *J* = 7.7 Hz, 3H); **^{13}C NMR** (100 MHz, $CDCl_3$) δ 178.7, 130.7, 130.0, 127.9, 123.4, 122.4, 107.9, 40.5, 26.1, 15.3; **IR** (film) υ_{max} 3023, 2851, 1941, 1865, 1697, 1601, 1489, 1452, 1370, 1179, 1153, 1067, 1027, 964, 906, 756, 703 cm^{-1}; MP = 119-124 °C. Characterization data of compound **25a** are compatible with the literature data see; reference [8].

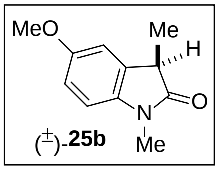

(±)-5-Methoxy-1,3-dimethylindolin-2-one (**25b**): The product obtained as light yellow solid (36 mg, 94% yield), R_f = 0.50 (40% EtOAc in hexane). **^{1}H NMR** (400 MHz, $CDCl_3$) δ 6.84-6.83 (m, 1H), 6.77 (dd, *J* = 8.5, 2.4 Hz, 1H), 6.69 (d, *J* = 8.4 Hz, 1H), 3.77 (s, 3H), 3.38 (q, *J* = 7.6 Hz, 1H), 3.15 (s, 3H), 1.44 (d, *J* = 7.7 Hz, 3H); **^{13}C NMR** (100 MHz, $CDCl_3$) δ 178.3, 156.0, 137.5, 132.0, 111.9, 111.2, 108.1, 55.8, 40.9, 26.2, 15.4; **IR** (film) υ_{max} 3023, 2920, 2842, 1698, 1601, 1492, 1453, 1373, 1291, 1233, 1153, 1059, 1030, 983, 807, 757, 703 cm^{-1}; MP = 115-117 °C. Characterization data of compound **25b** are compatible with the literature data see; reference [9].

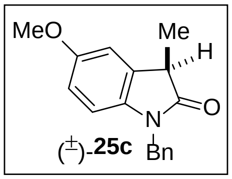

(±)-1-Benzyl-5-methoxy-3-methylindolin-2-one (**25c**): The product obtained as colorless gel (54 mg, 92% Yield), R_f = 0.41 (40% EtOAc in hexane). **^{1}H NMR** (400 MHz, $CDCl_3$) δ 7.31-7.20 (m, 5H), 6.85 (s, 1H), 6.66 (dd, *J* = 8.5, 2.4 Hz, 1H), 6.58 (d, *J* = 8.5 Hz, 1H), 4.87 (s, 2H), 3.74 (s, 3H), 3.51 (q, *J* = 7.6 Hz, 1H), 1.52 (d, *J* = 7.7 Hz, 3H); **^{13}C NMR** (100 MHz, $CDCl_3$) δ 178.5, 156.0, 136.5, 136.0, 132.0, 128.7, 127.5, 127.2, 112.0, 111.2, 109.3, 55.8, 43.8, 41.0, 15.7; **IR** (film) υ_{max} 3025, 2923, 2842, 1700, 1601, 1491, 1452, 1348, 1296, 1180, 1151, 1067, 1029, 973, 906, 805, 756, 703 cm^{-1}. Characterization data of compound **25c** are compatible with the literature data see: reference [10].

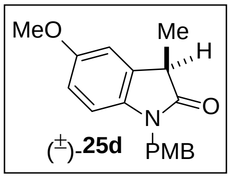

(±)-5-Methoxy-1-(4-methoxybenzyl)-3-methylindolin-2-one (**25d**): The product obtained as colorless solid (70 mg, 94% Yield), R_f = 0.48 (50% EtOAc in hexane). **^{1}H NMR** (400 MHz, $CDCl_3$) δ 7.21-7.17 (m, 2H), 6.85-6.80 (m, 3H), 6.67 (dd, *J* = 8.5, 2.3 Hz, 1H), 6.61 (d, *J* = 8.4 Hz, 1H), 4.80 (s, 2H), 3.74 (s, 3H), 3.73 (s, 3H), 3.48 (q, *J* = 7.7 Hz, 1H), 1.50 (d, *J* = 7.7 Hz, 3H); **^{13}C NMR** (100 MHz, $CDCl_3$) δ 178.5, 159.0, 156.0, 136.5, 132.0, 128.7, 128.1, 114.1, 111.9, 111.2, 109.3, 55.8, 55.2, 43.2, 41.0, 15.7; **IR** (film) υ_{max} 3025, 2928, 2845, 1698, 1601, 1489, 1450, 1371, 1339, 1288, 1248, 1179, 1147, 1113, 1032, 977, 900, 870, 811, 756, 703 cm^{-1}. mp = 77-79 °C; MP = 94-96 °C. Characterization data of compound **25d** are compatible with the literature data see: reference [11].

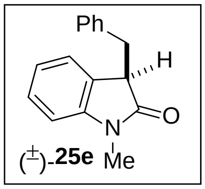

(±)-3-Benzyl-1-methylindolin-2-one (**25e**): The product obtained as yellow gel (39 mg, 99% Yield), R_f = 0.50 (30% EtOAc in hexane). **^{1}H NMR** (400 MHz, $CDCl_3$) δ 7.24-7.18 (m, 4H), 7.16-7.14 (m, 2H), 6.90 (t, *J* = 7.5 Hz, 1H), 6.73 (dd, *J* = 3.1, 7.8 Hz, 2H), 3.70 (dd, *J* = 4.4, 9.3 Hz, 1H), 3.49 (dd, *J* = 4.4, 13.6 Hz, 1H), 3.13 (s, 3H), 2.87 (dd, *J* = 9.3, 4.3 Hz, 1H); **^{13}C NMR** (100 MHz, $CDCl_3$) δ 177.0, 144.2, 138.0, 129.4, 128.4, 128.3, 127.9, 126.6, 124.5, 122.0, 107.9, 47.0, 36.8, 26.1; **IR** (film) υ_{max} 3025, 2923, 1715, 1602, 1492, 1452, 1375, 1154, 1089, 1028, 907, 756, 703 cm^{-1}. Characterization data of compound **25e** are compatible with the literature data see; reference [12].

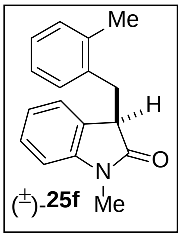

(±)-1-Methyl-3-(2-methylbenzyl)indolin-2-one (**25f**): The product obtained as light yellow solid (62 mg, 94% yield), R_f = 0.45 (40% EtOAc in hexane). **^{1}H NMR** (400 MHz, $CDCl_3$) δ 7.24-7.20 (m, 1H), 7.18-7.16 (m, 4H), 6.85 (t, *J* = 7.4 Hz, 1H), 6.81 (d, *J*

= 7.7 Hz, 1H), 6.54 (d, J = 7.4 Hz, 1H), 3.66 (dd, J = 11.0, 4.5 Hz, 1H), 3.52 (dd, J = 14.0, 4.7 Hz, 1H), 3.21 (s, 3H), 2.73 (dd, J = 10.9, 2.9 Hz, 1H), 2.27 (s, 3H); **^{13}C NMR** (100 MHz, $CDCl_3$) δ 177.3, 144.1, 136.8, 136.7, 130.5, 130.0, 128.6, 127.9, 126.8, 125.9, 124.7, 122.0, 107.9, 45.7, 34.4, 26.2, 19.6; **IR** (film) υ_{max} 3026, 2924, 1715, 1601, 1489, 1452, 1374, 1347, 1124, 1088, 1028, 907, 754, 703 cm^{-1}; MP = 70-72 °C. Characterization data of compound **25f** are compatible with the literature data see; reference [13].

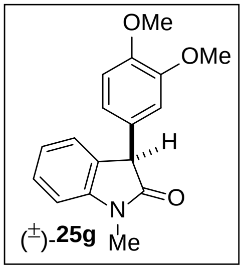

(±)-3-(3,4-dimethoxyphenyl)-1-methylindolin-2-one (**25g**): The product obtained as light yellow solid (172 mg, 93% Yield), R_f **=** 0.40 (50% EtOAc in hexane). **^{1}H NMR** (400 MHz, $CDCl_3$) δ 7.32 (t, J = 7.7 Hz, 2H), 7.17 (d, J = 7.4 Hz, 1H), 7.06 (t, J = 7.3 Hz, 1H), 6.88 (d, J = 7.8 Hz, 1H), 6.80 (d, J = 8.1 Hz, 1H), 6.73-6.72 (m, 1H), 4.53 (s, 1H), 3.83 (s, 3H), 3.81 (s, 3H), 3.23 (s, 3H); **^{13}C NMR** (100 MHz, $CDCl_3$) δ 176.2, 149.2, 148.6, 144.4, 128.9, 128.8, 128.4, 125.0, 122.7, 120.5, 111.7, 111.4, 108.1, 55.9, 51.5, 29.7, 26.4; **IR** (film) υ_{max} 3025, 2922, 1716, 1700, 1601, 1492, 1452, 1373, 1258, 1181, 1154, 1067, 1028, 962, 907, 757, 703 cm^{-1}; MP = 77-79 °C.

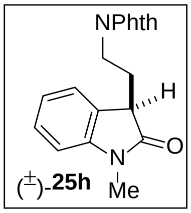

(±)-2-(2-(1-Methyl-2-oxoindolin-3-yl)ethyl)isoindoline-1,3-dione (**25h**) [6]: The product obtained as colorless solid (22 mg, 79% Yield), R_f **=** 0.40 (50% EtOAc in hexane). **^{1}H NMR** (400 MHz, $CDCl_3$) δ 7.71-7.61 (m, 4H), 7.24 (d, J = 5.9 Hz, 1H), 7.06 (t, J = 7.4 Hz, 1H), 6.82 (t, J = 7.4 Hz, 1H), 6.71 (d, J = 7.8 Hz, 1H), 3.93-2.86 (m, 1H), 3.74-3.68 (m, 1H), 3.50 (t, J = 5.7 Hz, 1H), 3.15 (s, 3H), 2.54-2.45 (m, 1H), 2.36-2.28

(m, 1H); **^{13}C NMR** (100 MHz, $CDCl_3$) δ 176.8, 168.0, 144.3, 133.7, 132.0, 127.9, 123.4, 123.7, 122.3, 108.1, 43.5, 35.0, 28.0, 26.2, 19.6; **IR** (film) υ_{max} 3025, 2924, 1714, 1699, 1601, 1492, 1451, 1374, 1182, 1067, 1028, 964, 907, 839, 757, 703 cm^{-1}. Characterization data of compound **25h** are compatible with the literature data see; reference [14].

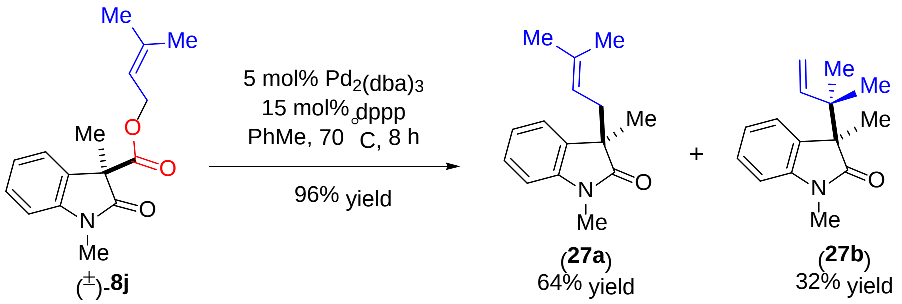

Procedure for decarboxylative prenylation and *reverse* prenylation to the synthesis of 27: As described in reference 1, in an oven-dried sealed tube, toluene (2 mL) was degassed by using nitrogen balloon over a period of 10 minutes at room temperature. 7.5 mol % of $Pd_2(dba)_3$ and 15 mol % 1,3-bis(diphenylphosphino)propane (dppp) were added to it and stirring was continued for 20 minutes (greenish color persisted indicating the complex formation). Then ester (±)-**8j** (30mg, 0.1 mmol; 1.0 equiv) was dissolved in dry degassed toluene (2 mL) and the solution was added drop wise to the complex solution. The reaction mixture was then placed over 70 °C oil bath and stirring was continued for 8 h. After full consumption of starting material (judged by TLC analysis) the reaction mixture was concentrated and purified by column chromatography using hexanes/EtOAc as eluent to the desired product.

Synthetic procedure and spectral data of **13**, **14**, 1**8a,b**, **19a,b**, **26**, **27** see: reference [1] and for compounds **20a,b**, **21a,b** see: references [6,7].

References:

1. For synthetic procedure and characterization data of compounds **3a-d**, **3k**, **4a-s**, **9**, **8a-v**, **10j**, **11b**, **12b, 13, 14, 17a, 17b, 18a, 18b, 19a, 19b, 26**, **27** see: Ghosh, S.;

De, S.; Kakde, B. N.; Bhunia, S.; Adhikary, A.; Bisai, A. *Org. Lett.* **2012**, *14*, 5864.

2. For the characterization of compound (**4t-u**) see: Ju ,X.; Liang,Y.; Jia, P.; Li*a*, W.; Yu, W. *Org. Biomol. Chem*. **2012**, *10*, 498.
3. For the characterization of compound (**3g-h**), **6e** see: Bhunia, S.; Ghosh, S.; Dey, D.; Bisai, A. *Org. Lett.* **2013**, *15*, 2426.
4. For the characterization of compound (**6a**) see: Ismail, M.; Nguyen, H. V.; Ilyashenko, G.; Motevalli, M.; Richards, C. J. *Tetrahedron. Lett.* **2009**, *50*, 6332.
5. For the characterization of compound **10a**, **10g** see: Franckevičius, V.; Cuthbertson, J. D.; Pickworth, M.; Pugh, D. S.; and Taylor, R. J. K. *Org. Lett.* **2011**, *13*, 4264.
6. For the characterization of compound **19c, 20a-b**, **21a-b** see: Ghosh, S.; Bhunia, S.; Kakde, B. N.; De, S.; Bisai, A. *Chem. Commun.* **2014**, *50*, 2434.
7. Ghosh, Chaudhuri, Bisai. *Chem. –Eur. J.* **2015**, *21*, 17479.
8. For the characterization of compound **25a** see: Ghosh, S.; Chaudhuri, S.; Bisai, A. *Org. Lett.* **2015**, *17*, 1373.
9. For the characterization of compound **25b** see: Liu, C.; Liu, D.; Zhang, W.; Zhou, L.; Lei, A. *Org. Lett.* **2013**, *15*, 6166.
10. For the characterization of compound **25c** see: Silva, G. P. da.; Ali, A.; Silva, R. Da.; Jiang, H.; Paixão, M. W. *Chem. Commun.* **2015**, *51*, 15110.
11. For the characterization of compound **25d** see: Yang, Y.; Moinodeen, F.; Chin, W.; Ma, T.; Jiang, Z.; Tan, C.-H. *Org. Lett.* **2012**, *14*, 4762.
12. For the characterization of compound **25e** see: Huang, L.; Li, J.; Zhao, Y.; Ye, X.; Liu, Y.; Yan, L.; Tan, C.-H.; Liu, H.; Jiang, Z.; *J. Org. Chem.* **2015**, *80*, 8933.
13. For the characterization of compound **25f** see: Liu, X. –L.; Pan, B. –W.; Zhang, W. –H.; Yang, C.; Yang, J.; Shi, Y.; Feng, T. –T.; Zhou, Y.; Yuan, W. –C. *Org. Biomol. Chem*. **2015**, *13*, 601.
14. For the characterization of compound **25h** see: De, S.; Das, M. K.; Bhunia, S.; Bisai, A. *Org. Lett.* **2015**, *17*, 5922.
15. For the characterization of compound **29a** see: Yuan, Y.; Yang, R.; Zhang-Negrerie, D.; Wang, J.; Du, Y.; Zhao, K. *J. Org. Chem.* **2013**, *78*, 5385.

16. For the characterization of compound **29b** see: Lv, J.; Zhang-Negrerie, D.; Deng, J.; Du, Y.; Zhao, K. *J. Org. Chem.* **2014**, *79*, 1111.
17. For the characterization of compound **5k** see: Pugh, D. S.; Klein, J. E. M. N.; Perry, A.; Taylor, R. J. K. *Synlett* **2010**, 934.

Spectral Graphics

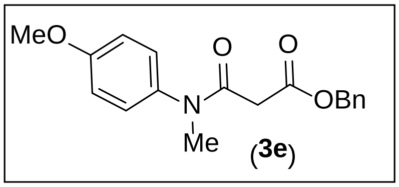

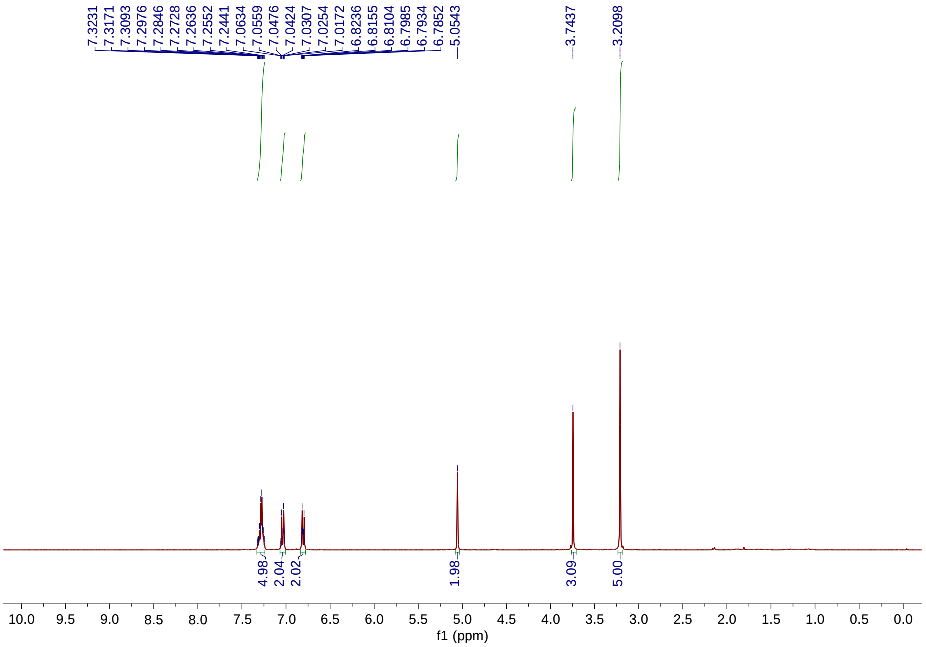

^{1}H NMR (400 MHz, $CDCl_3$) of compound (**3e**)

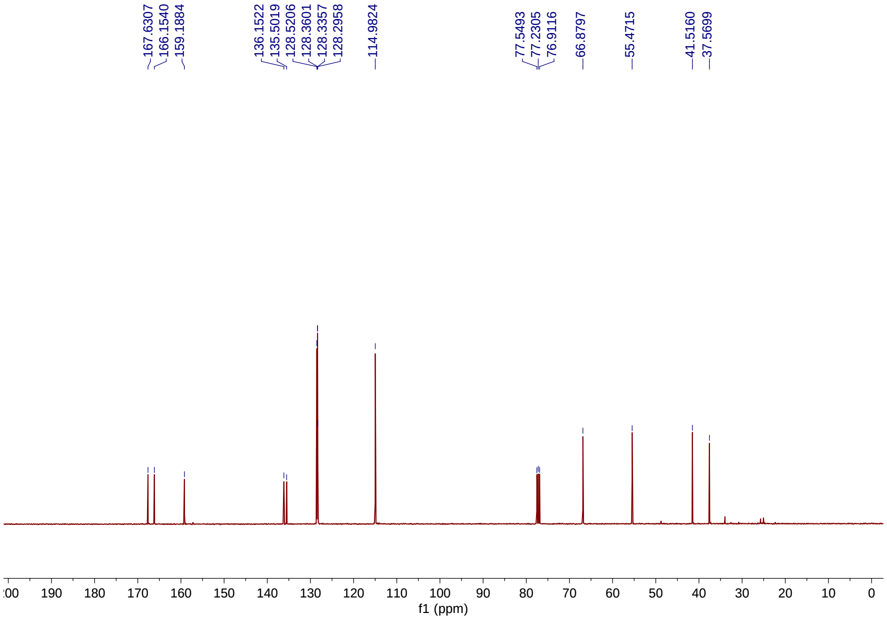

^{13}C NMR (100 MHz, $CDCl_3$) of compound (**3e**)

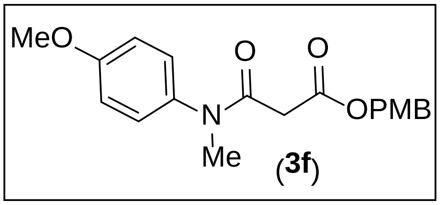

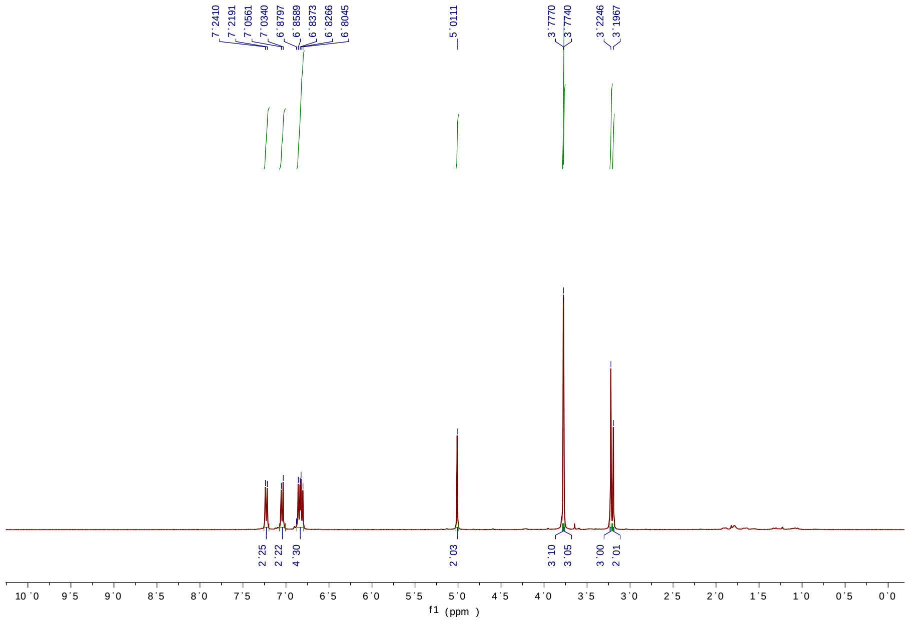

^{1}H NMR (400 MHz, $CDCl_3$) of compound (**3f**)

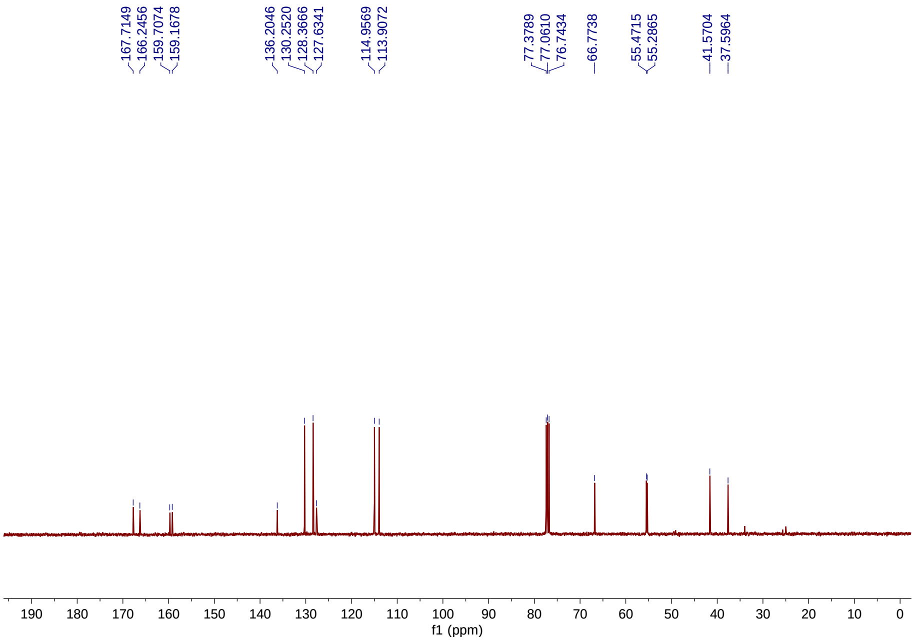

^{13}C NMR (100 MHz, $CDCl_3$) of compound (**3f**)

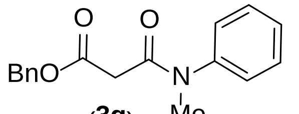

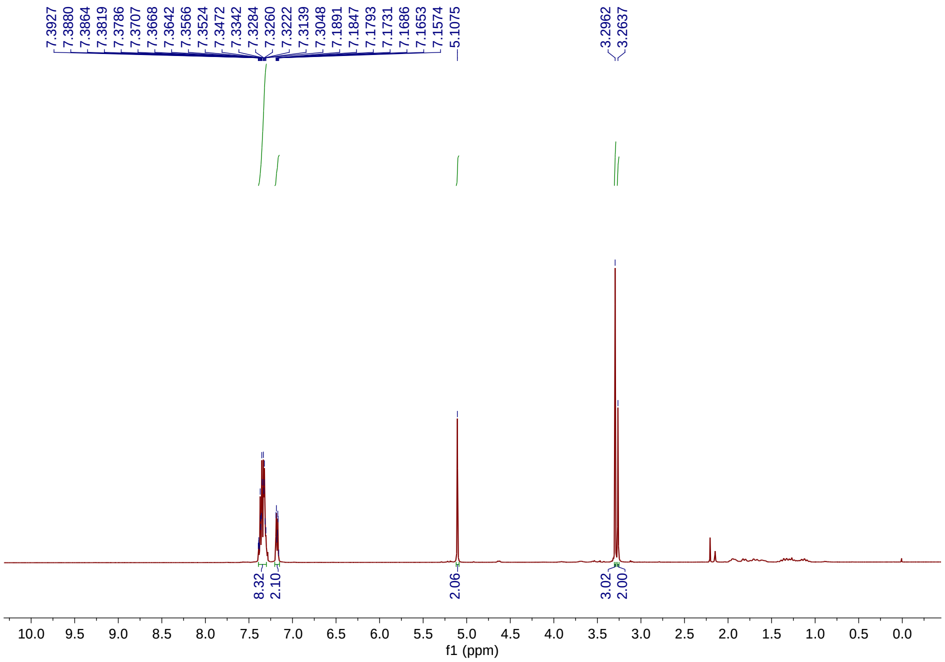

^{1}H NMR (400 MHz, $CDCl_3$) of compound (**3g**)

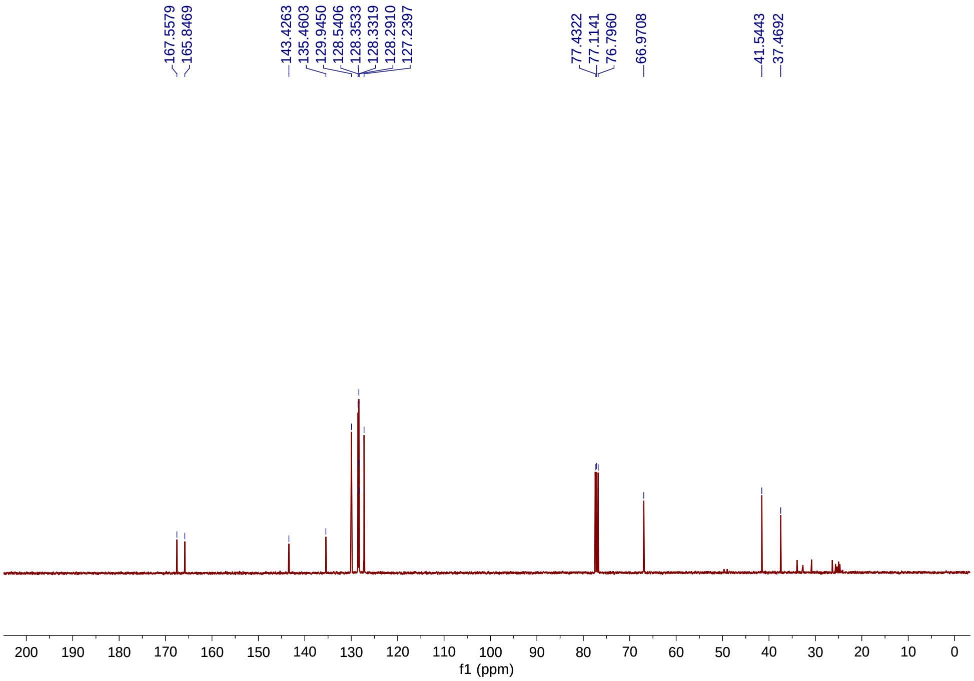

^{13}C NMR (100 MHz, $CDCl_3$) of compound (**3g**)

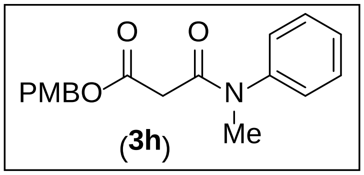

(**3h**)

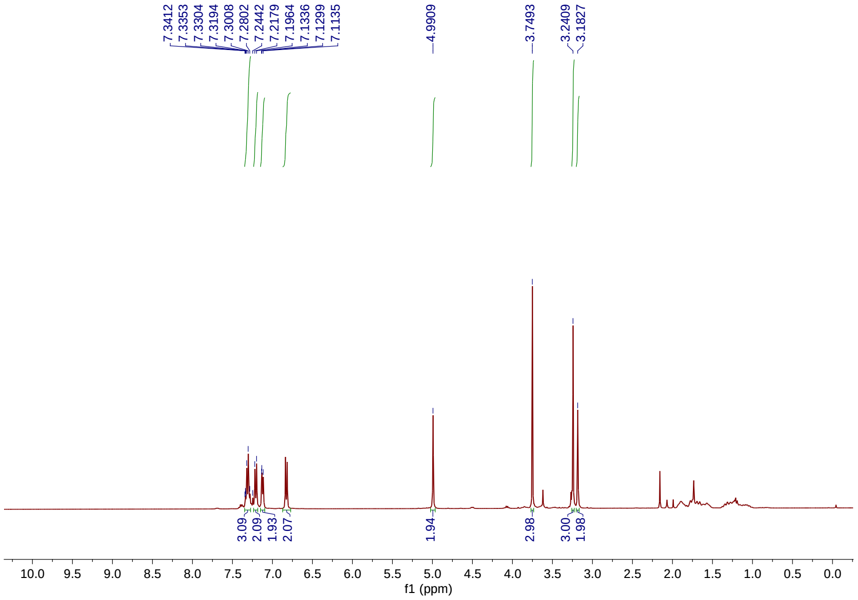

^{1}H NMR (400 MHz, $CDCl_3$) of compound (**3h**)

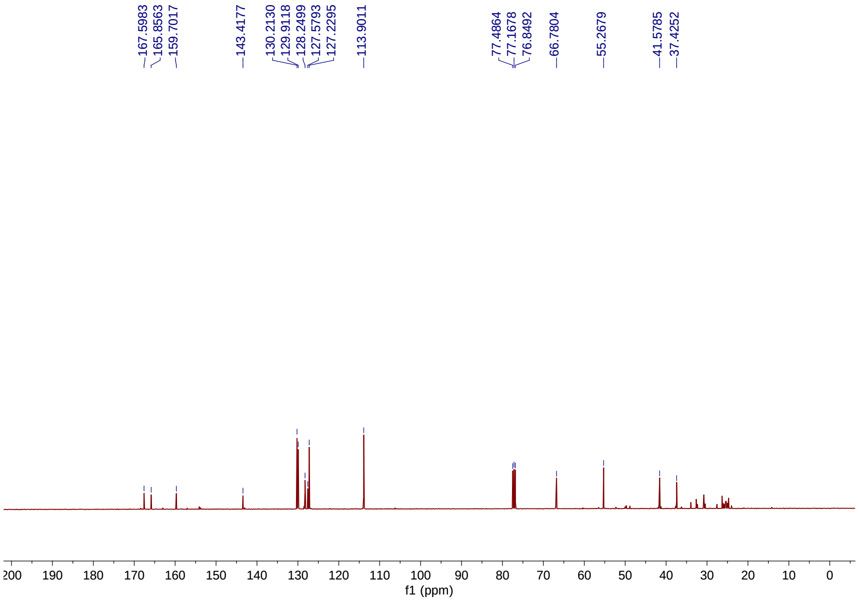

^{13}C NMR (100 MHz, $CDCl_3$) of compound (**3h**)

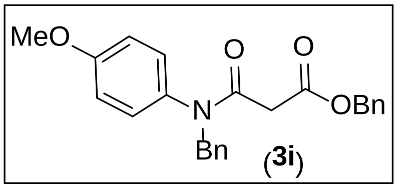

(**3i**)

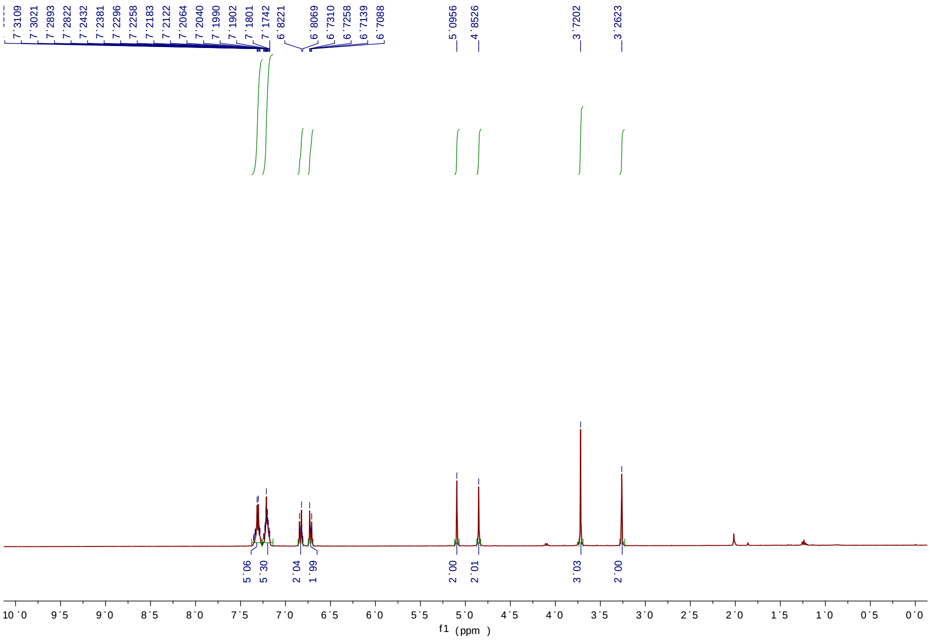

^{1}H NMR (400 MHz, $CDCl_3$) of compound (**3i**)

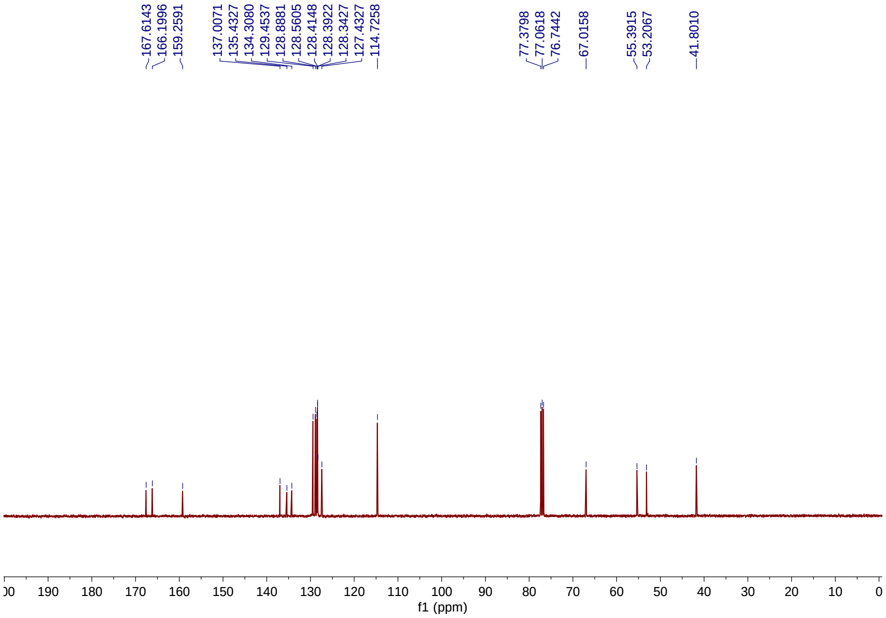

^{13}C NMR (100 MHz, $CDCl_3$) of compound (**3i**)

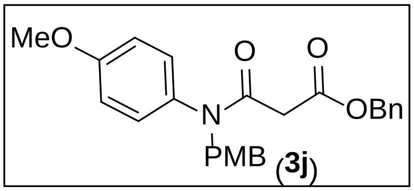

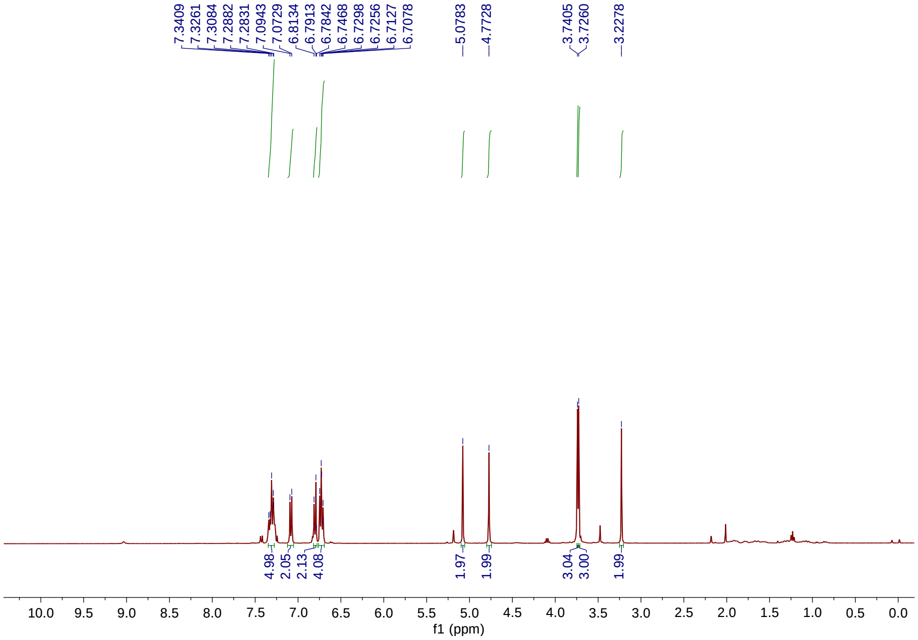

^{1}H NMR (400 MHz, $CDCl_3$) of compound (**3j**)

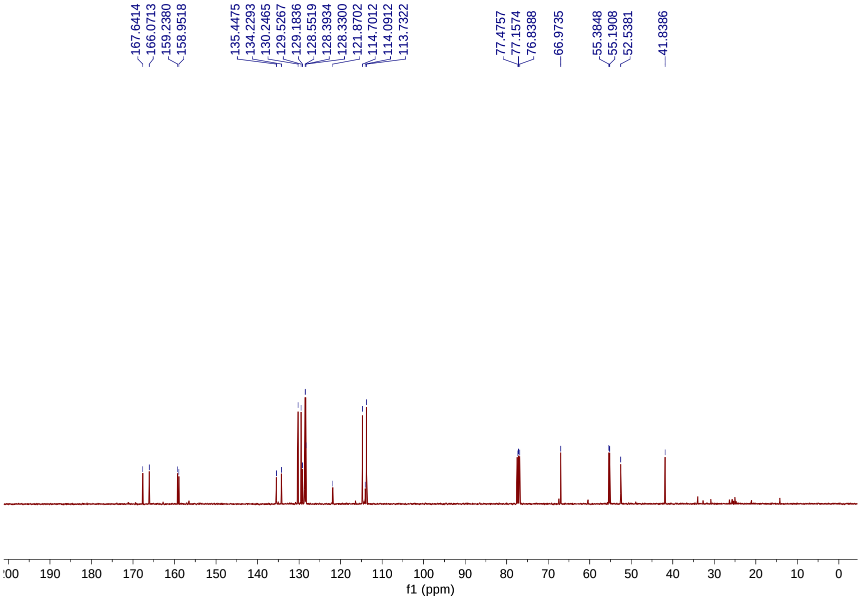

^{13}C NMR (100 MHz, $CDCl_3$) of compound (**3j**)

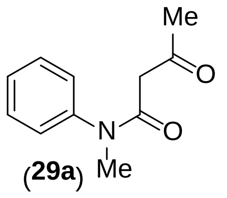

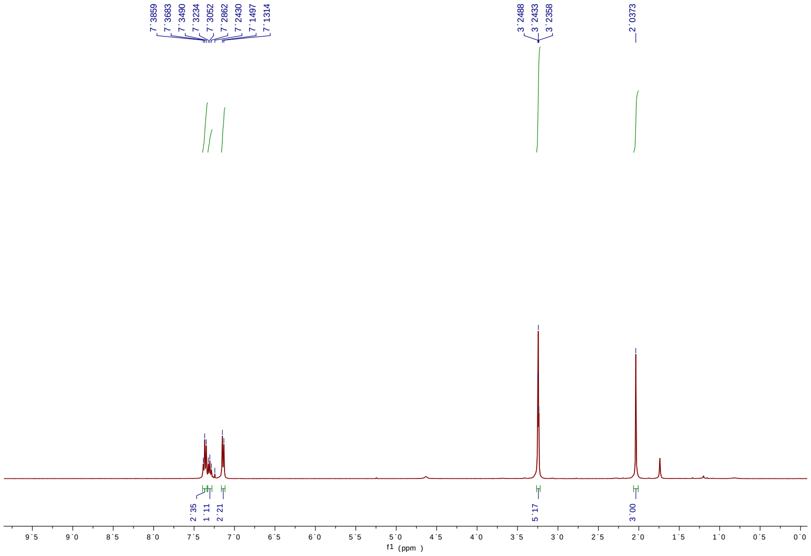

^{1}H NMR (400 MHz, $CDCl_3$) of compound (**29a**)

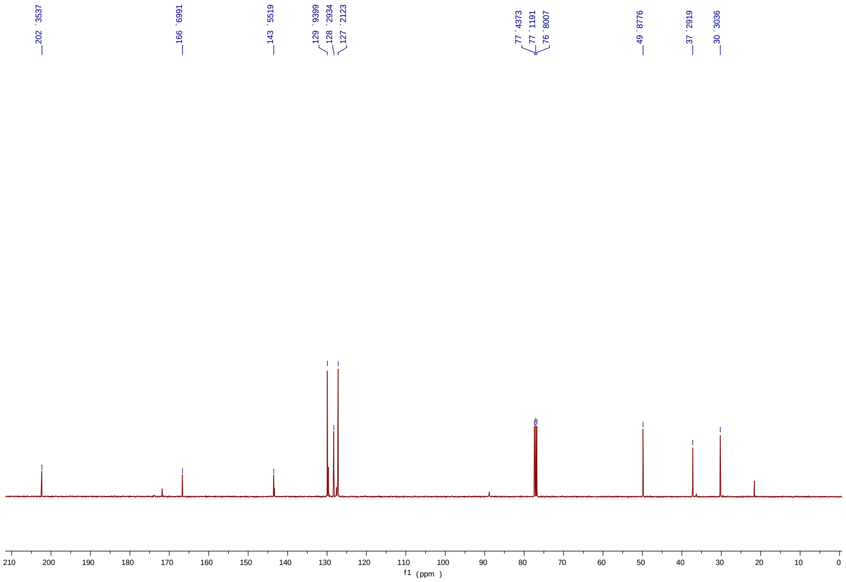

^{13}C NMR (100 MHz, $CDCl_3$) of compound (**29a**)

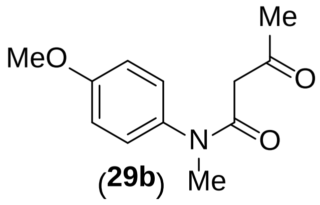

(**29b**)

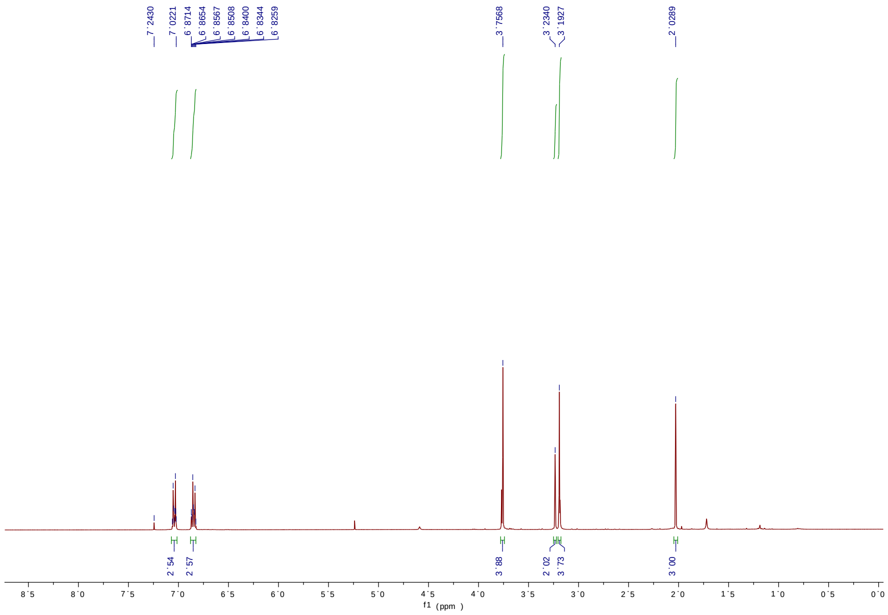

^{1}H NMR (400 MHz, $CDCl_3$) of compound (**29b**)

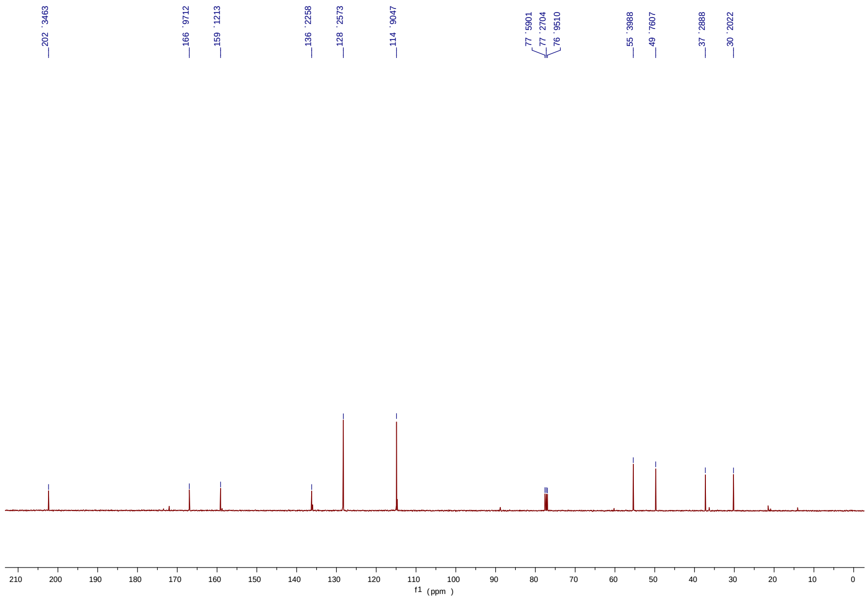

^{13}C NMR (100 MHz, $CDCl_3$) of compound (**29b**)

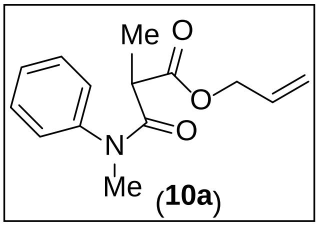

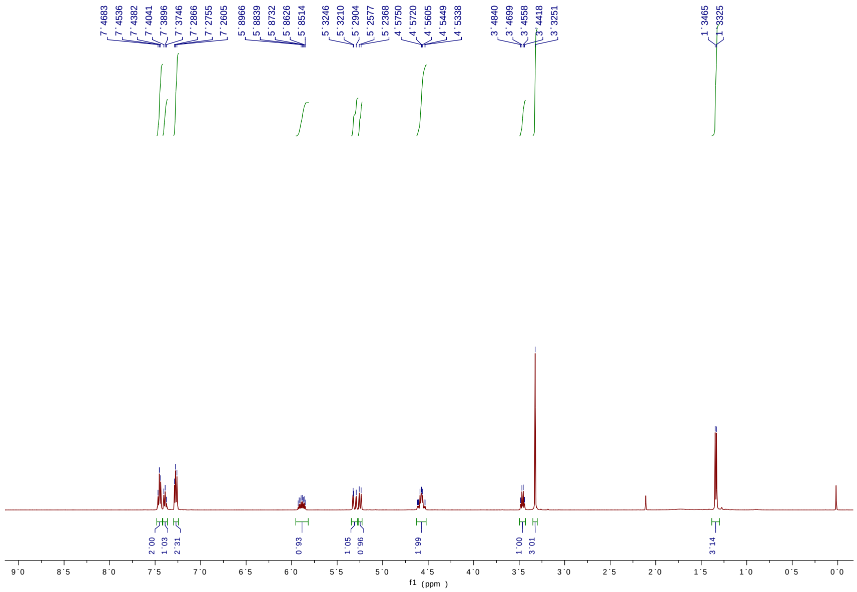

^{1}H NMR (500 MHz, $CDCl_3$) of compound (±)-**10a**

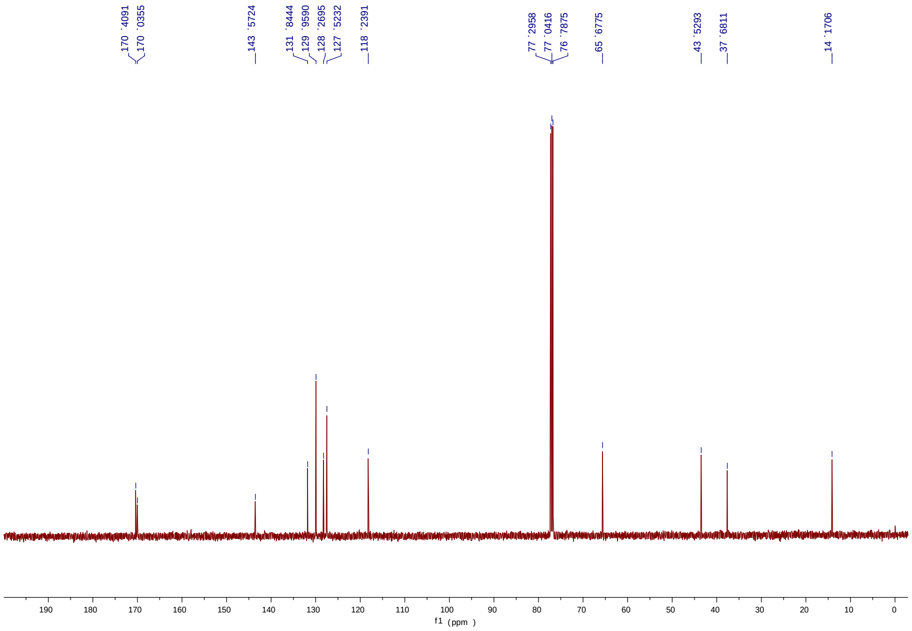

^{13}C NMR (125 MHz, $CDCl_3$) of compound (±)-**10a**

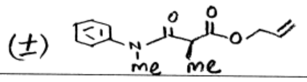

Display Report

Analysis Info

Analysis Name	D:\Data\user data\MARCH 2012\05 mar\Dr. A. Bisai- SG4-184_1-A,3_01_1431.d	Acquisition Date	3/6/2012 3:51:15 PM
Method	HRLCMS-20 Sept.m	Operator	Meena Sharma
Sample Name	Dr. A. Bisai- SG4-184	Instrument	micrOTOF-Q II 10330
Comment			

Acquisition Parameter

Source Type	ESI	Ion Polarity	Positive	Set Nebulizer	1.2 Bar
Focus	Active	Set Capillary	4500 V	Set Dry Heater	200 °C
Scan Begin	50 m/z	Set End Plate Offset	-500 V	Set Dry Gas	7.0 l/min
Scan End	3000 m/z	Set Collision Cell RF	130.0 Vpp	Set Divert Valve	Waste

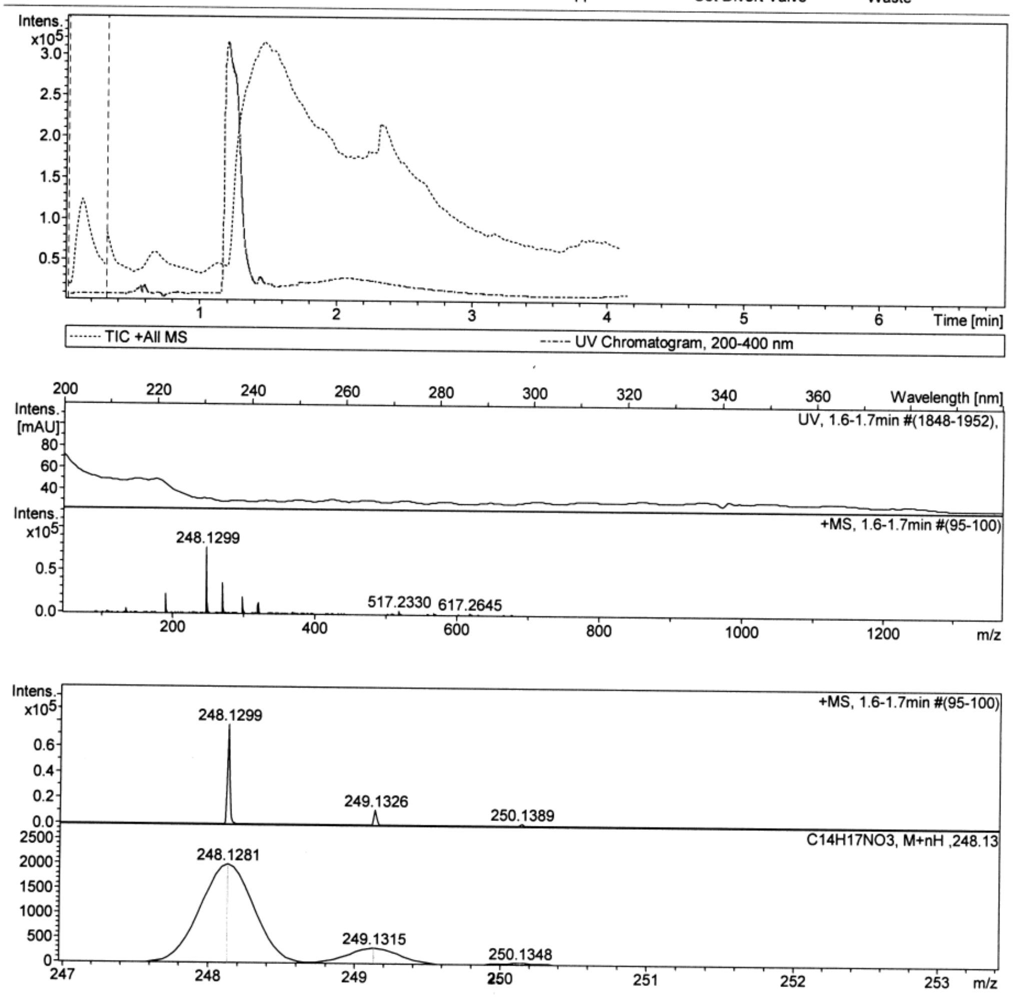

Bruker Compass DataAnalysis 4.0 printed: 3/6/2012 4:00:29 PM Page 1 of 1

Scanned copy of mass spectrum (HRMS) of (±)-**10a**

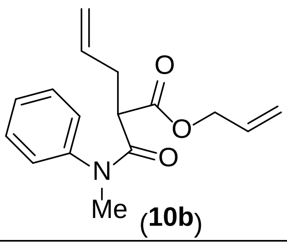

(**10b**)

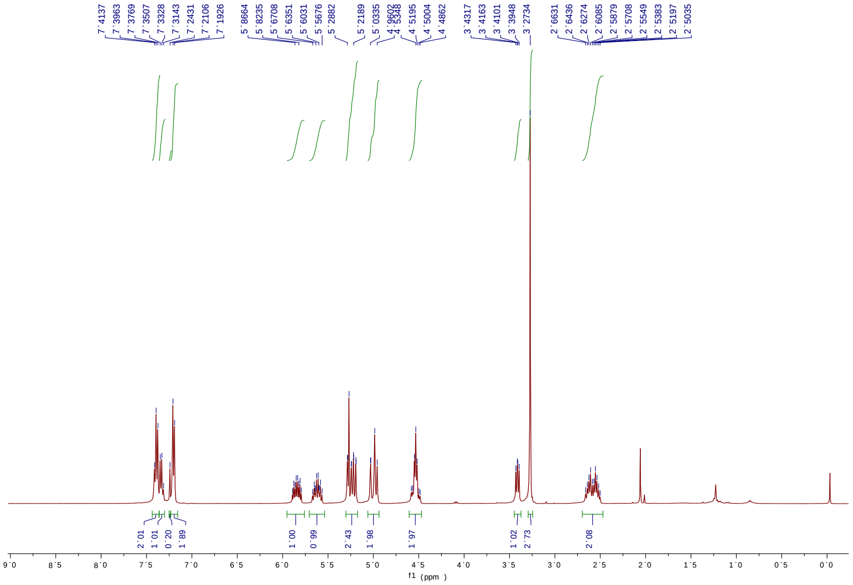

^{1}H NMR (400 MHz, CDCl$_3$) of compound (±)-**10b**

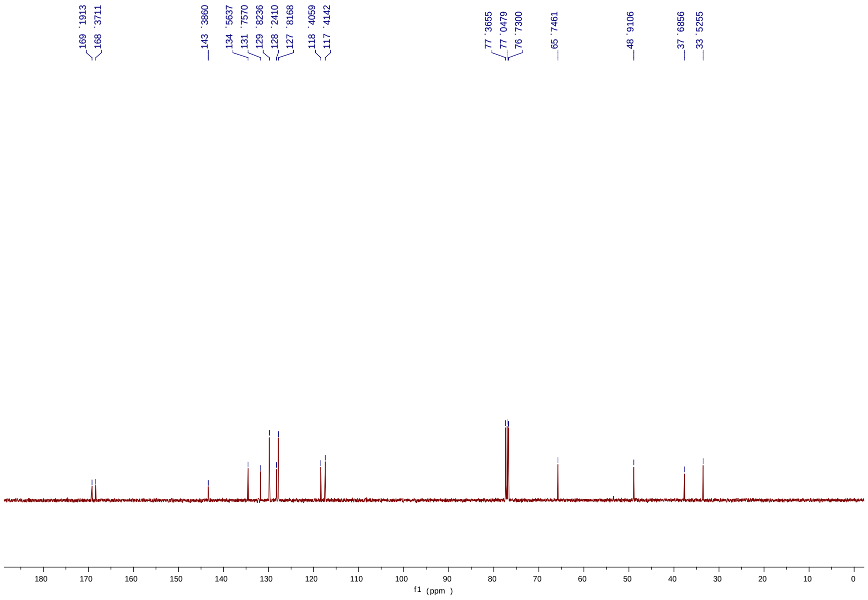

^{13}C NMR (100 MHz, CDCl$_3$) of compound (±)-**10b**

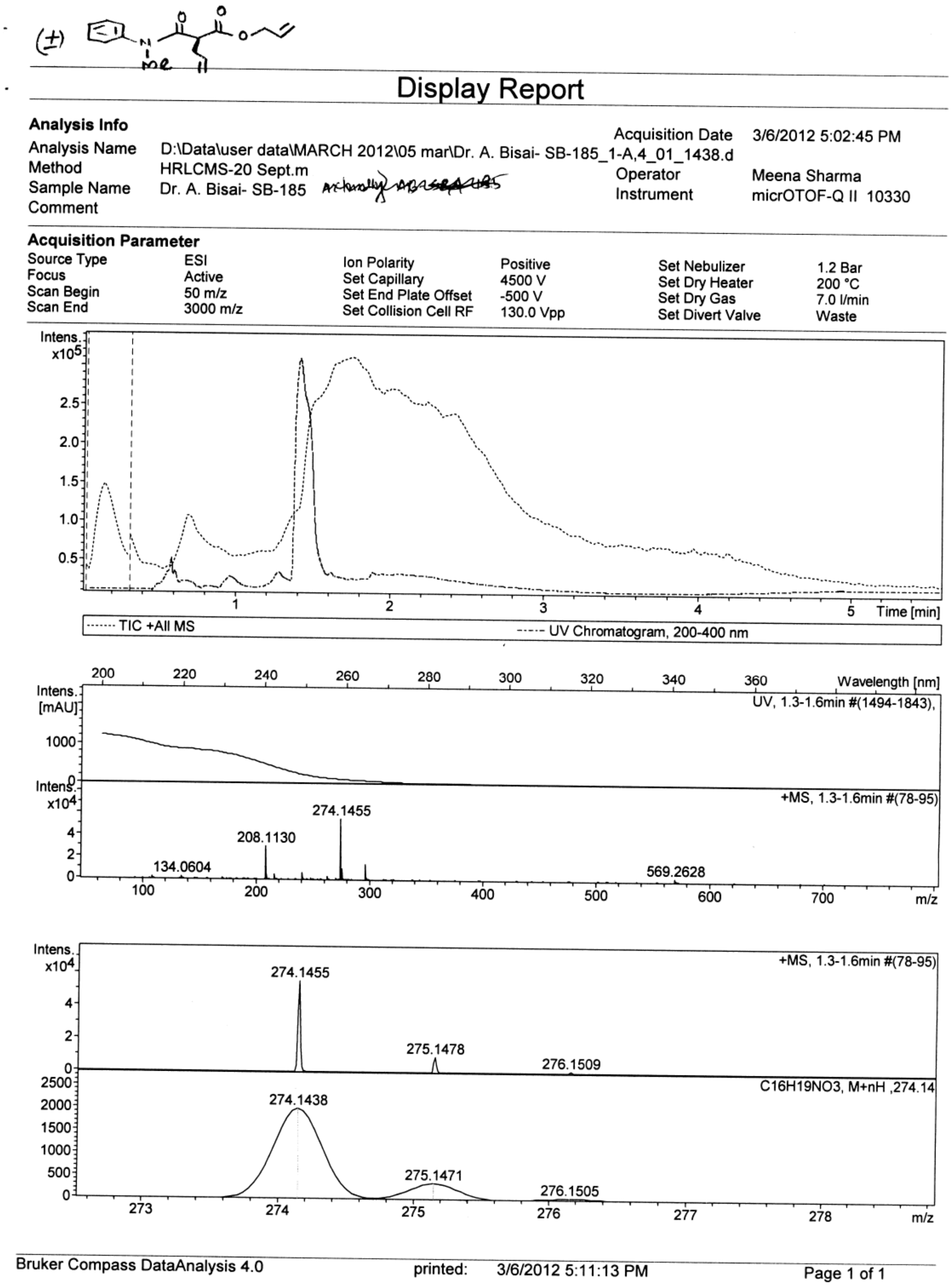

Scanned copy of mass spectrum (HRMS) of (±)-**10b**

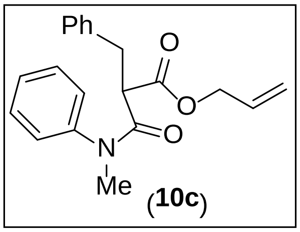

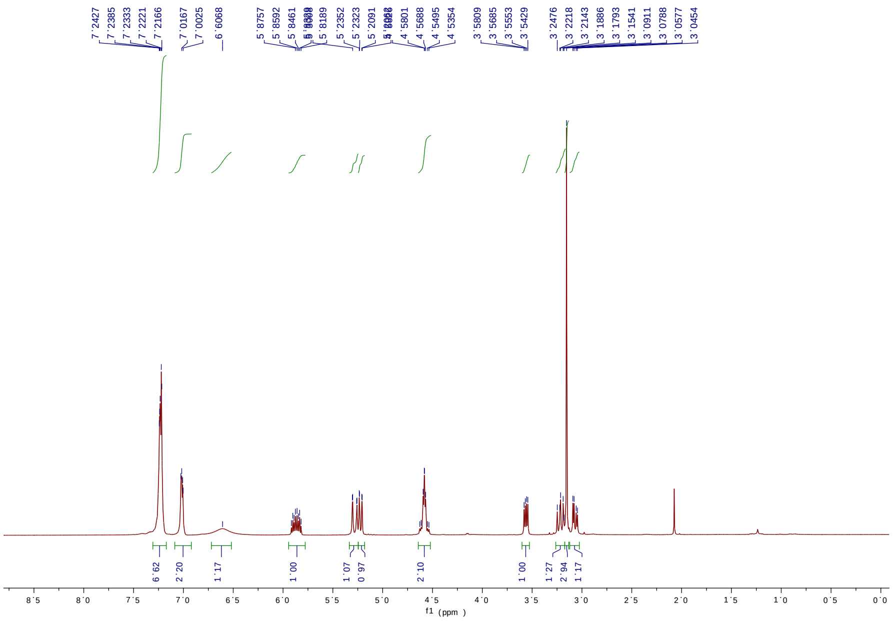

^{1}H NMR (400 MHz, CDCl$_3$) of compound (±)-**10c**

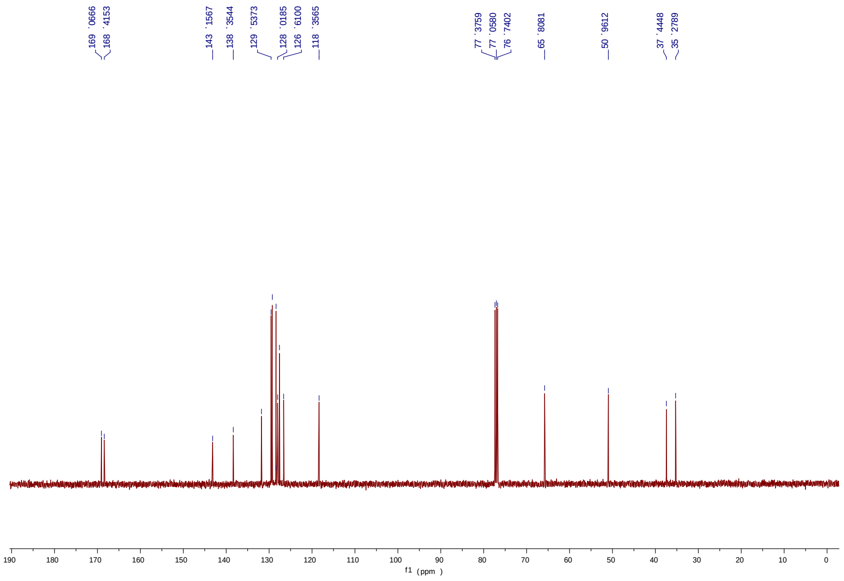

^{13}C NMR (100 MHz, CDCl$_3$) of compound (±)-**10c**

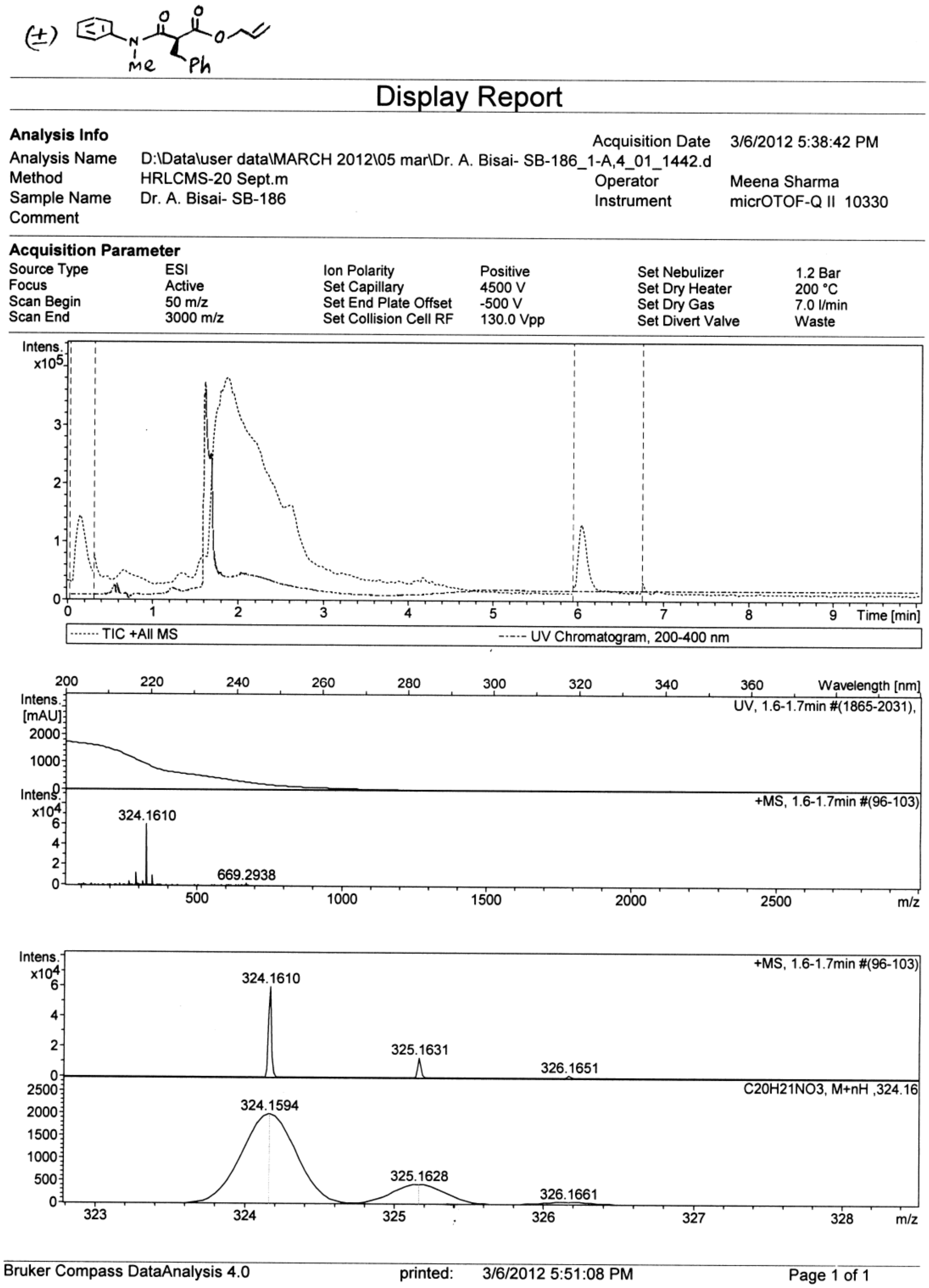

Scanned copy of mass spectrum (HRMS) of (±)-**10c**

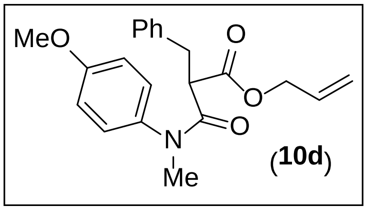

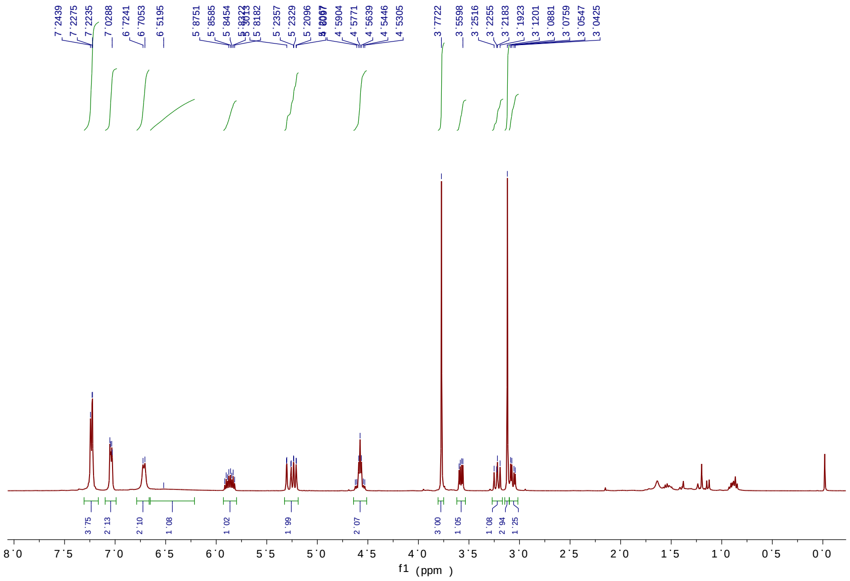

^{1}H NMR (400 MHz, $CDCl_3$) of compound (±)-**10d**

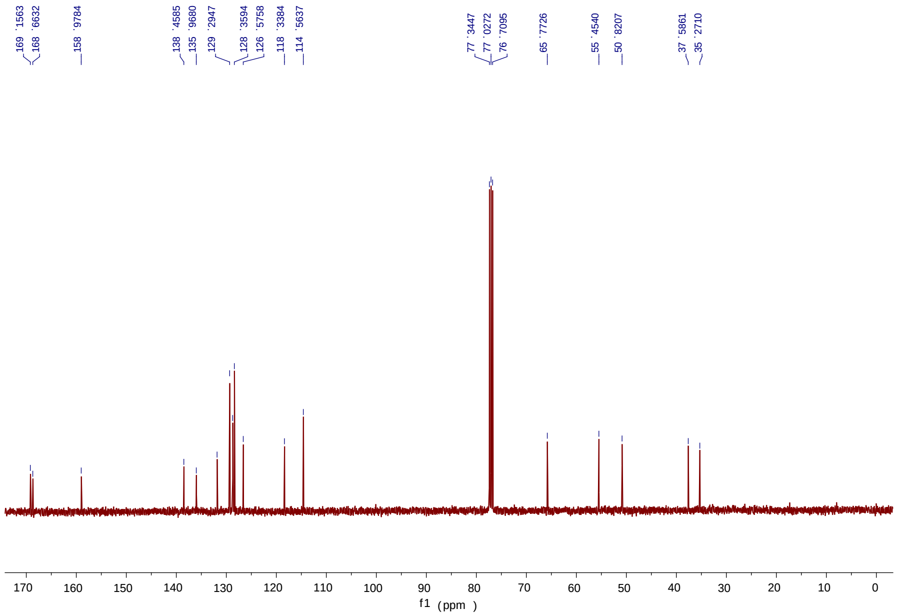

^{13}C NMR (100 MHz, $CDCl_3$) of compound (±)-**10d**

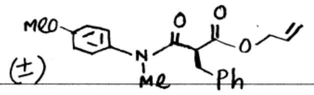

Display Report

Analysis Info

		Acquisition Date	3/20/2013 12:07:52 PM
Analysis Name	D:\Data\user data\2013\MARCH\20 MAR\Dr.A.Bisai-SB3-55_1-A,7_01_687.d		
Method	HRLCMS-20 Sept.m	Operator	Amit
Sample Name	Dr.A.Bisai-SB3-55	Instrument	micrOTOF-Q II 10330
Comment			

Acquisition Parameter

Source Type	ESI	Ion Polarity	Positive	Set Nebulizer	1.2 Bar
Focus	Active	Set Capillary	4500 V	Set Dry Heater	200 °C
Scan Begin	50 m/z	Set End Plate Offset	-500 V	Set Dry Gas	7.0 l/min
Scan End	3000 m/z	Set Collision Cell RF	130.0 Vpp	Set Divert Valve	Waste

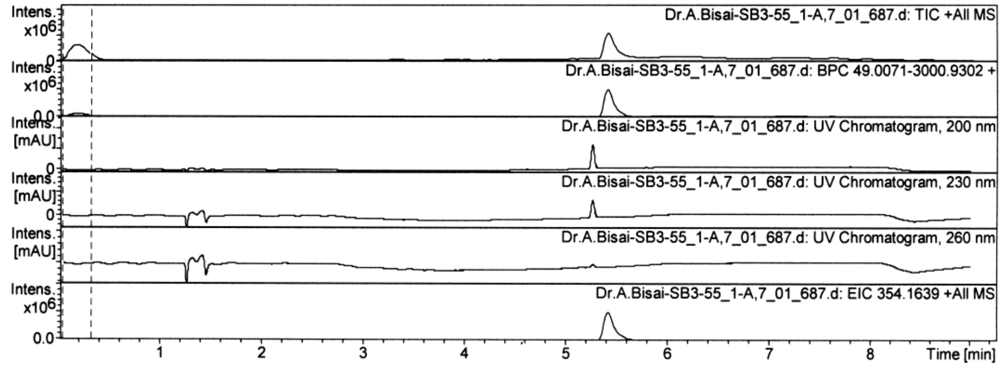

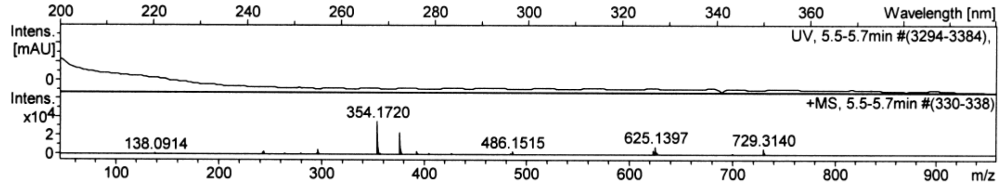

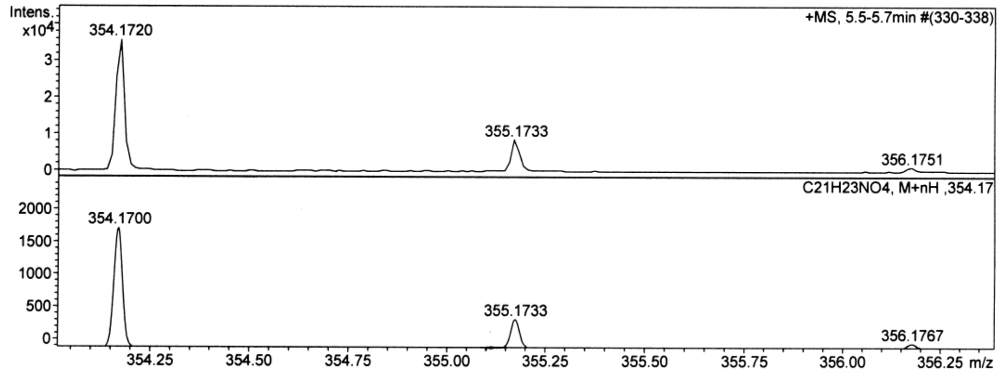

Bruker Compass DataAnalysis 4.0 printed: 3/20/2013 12:23:58 PM Page 1 of 1

Scanned copy of mass spectrum (HRMS) of (±)-**10d**

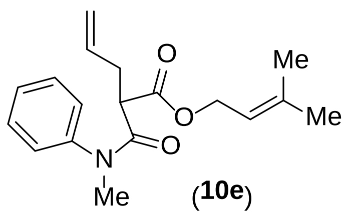

(**10e**)

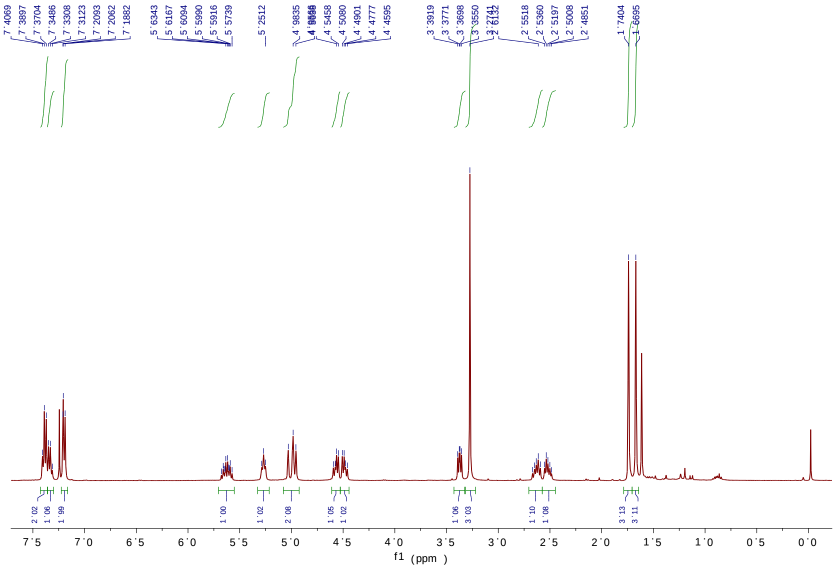

^{1}H NMR (400 MHz, $CDCl_3$) of compound (**10e**)

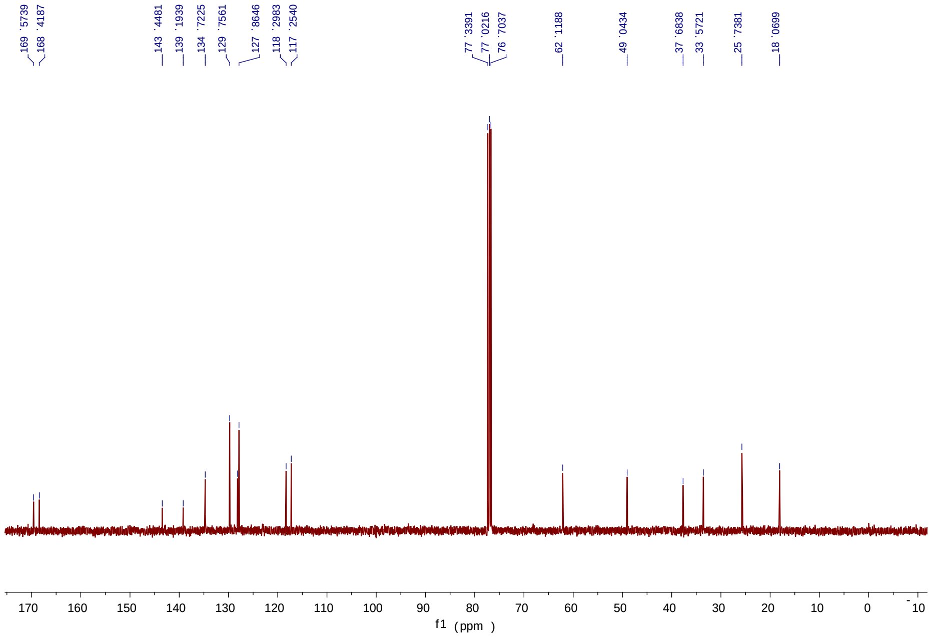

^{13}C NMR (100 MHz, $CDCl_3$) of compound (**10e**)

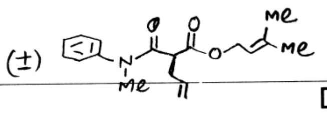

Display Report

Analysis Info		Acquisition Date	3/22/2013 2:59:37 PM
Analysis Name	D:\Data\user data\2013\MARCH\22 MAR\Dr.A.Bisai-SB3-69_1-D,3_01_731.d		
Method	HRLCMS-20 Sept.m	Operator	Amit
Sample Name	Dr.A.Bisai-SB3-69	Instrument	micrOTOF-Q II 10330
Comment			

Acquisition Parameter

Source Type	ESI	Ion Polarity	Positive	Set Nebulizer	1.2 Bar
Focus	Active	Set Capillary	4500 V	Set Dry Heater	200 °C
Scan Begin	50 m/z	Set End Plate Offset	-500 V	Set Dry Gas	7.0 l/min
Scan End	3000 m/z	Set Collision Cell RF	130.0 Vpp	Set Divert Valve	Waste

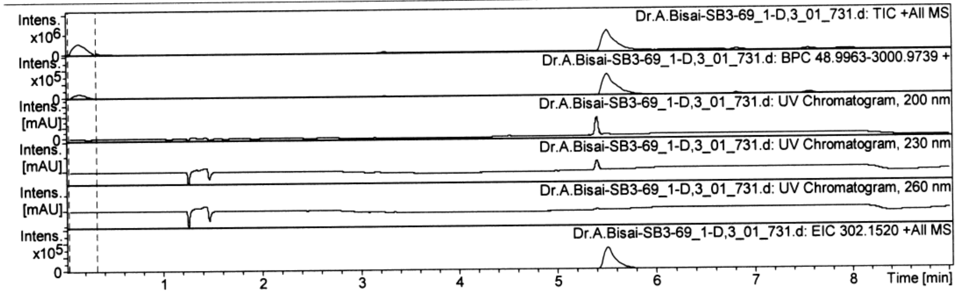

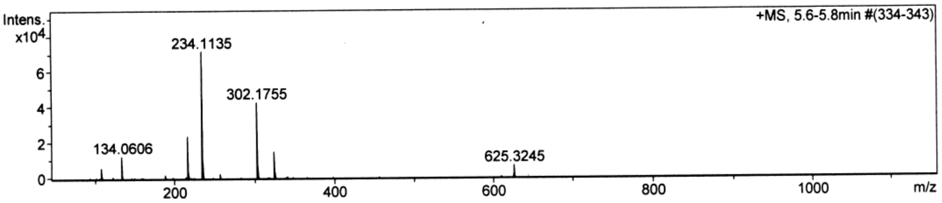

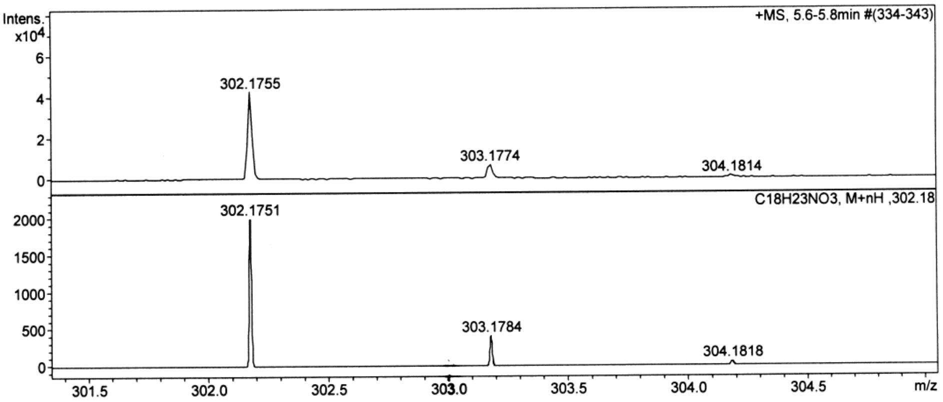

Scanned copy of mass spectrum (HRMS) of (**10e**)

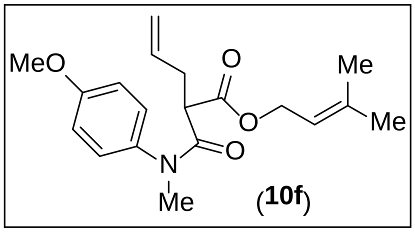

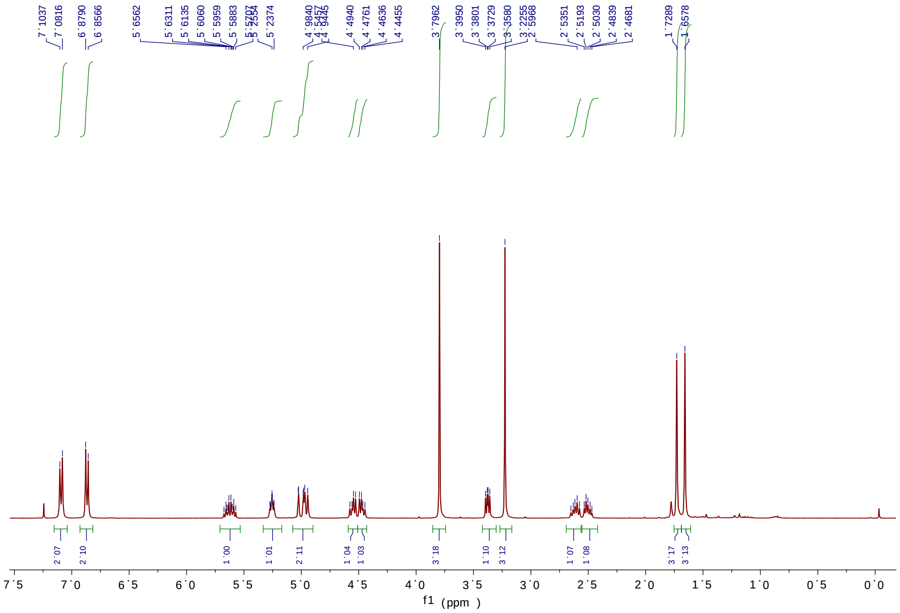

^{1}H NMR (400 MHz, $CDCl_3$) of compound (**10f**)

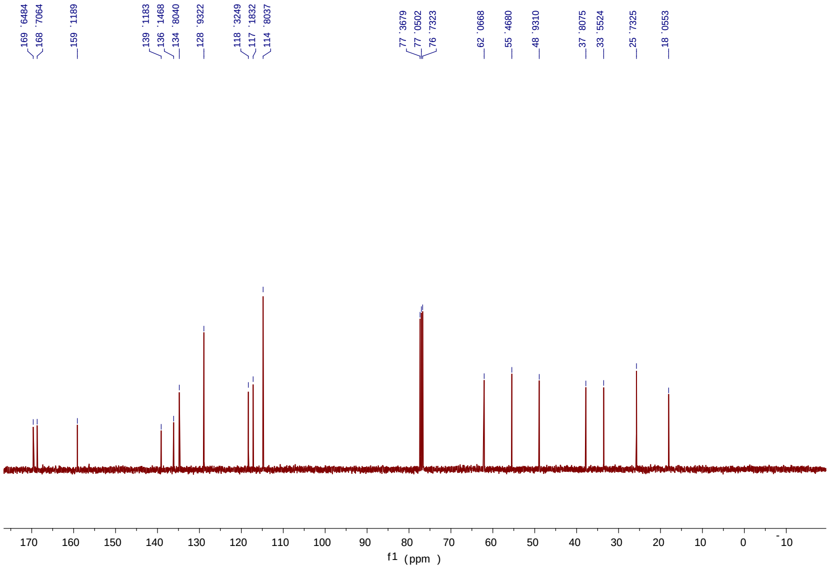

^{13}C NMR (100 MHz, $CDCl_3$) of compound (**10f**)

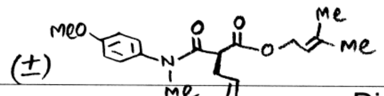

Display Report

Analysis Info

Analysis Name	D:\Data\user data\2013\MARCH\22 MAR\Dr.A.Bisai-SB3-70_1-D,4_01_732.d	Acquisition Date	3/22/2013 3:09:47 PM
Method	HRLCMS-20 Sept.m	Operator	Amit
Sample Name	Dr.A.Bisai-SB3-70	Instrument	micrOTOF-Q II 10330
Comment			

Acquisition Parameter

Source Type	ESI	Ion Polarity	Positive	Set Nebulizer	1.2 Bar
Focus	Active	Set Capillary	4500 V	Set Dry Heater	200 °C
Scan Begin	50 m/z	Set End Plate Offset	-500 V	Set Dry Gas	7.0 l/min
Scan End	3000 m/z	Set Collision Cell RF	130.0 Vpp	Set Divert Valve	Waste

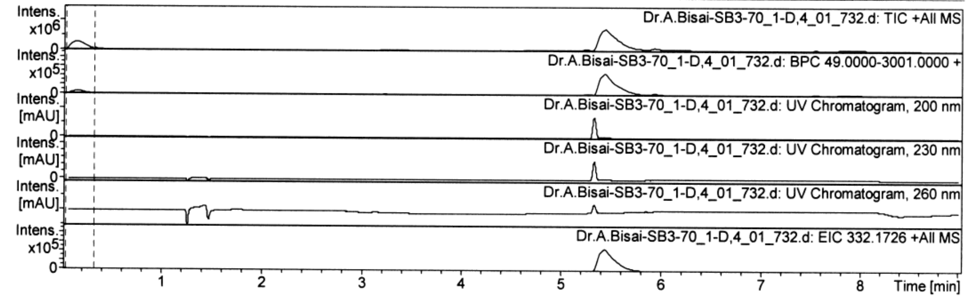

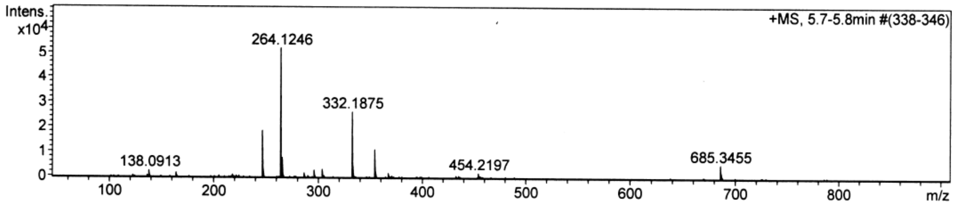

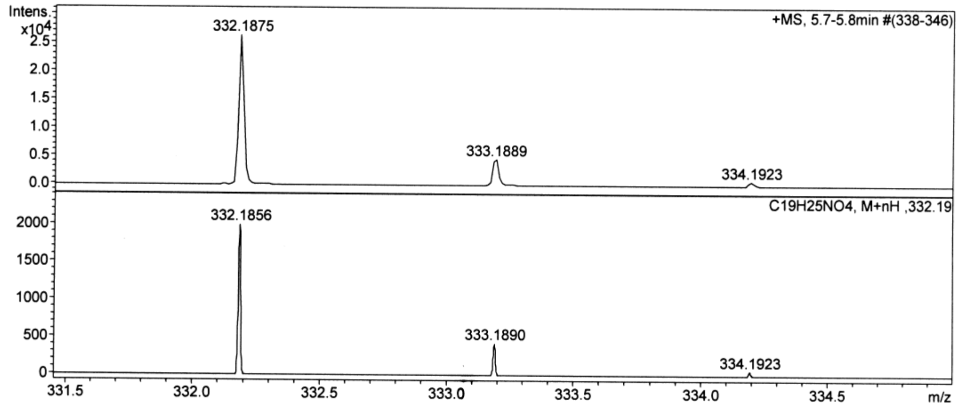

Bruker Compass DataAnalysis 4.0 printed: 3/22/2013 4:28:43 PM Page 1 of 1

Scanned copy of mass spectrum (HRMS) of (**10f**)

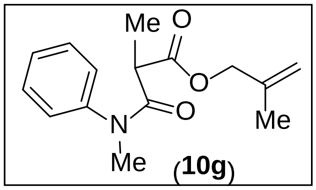

(**10g**)

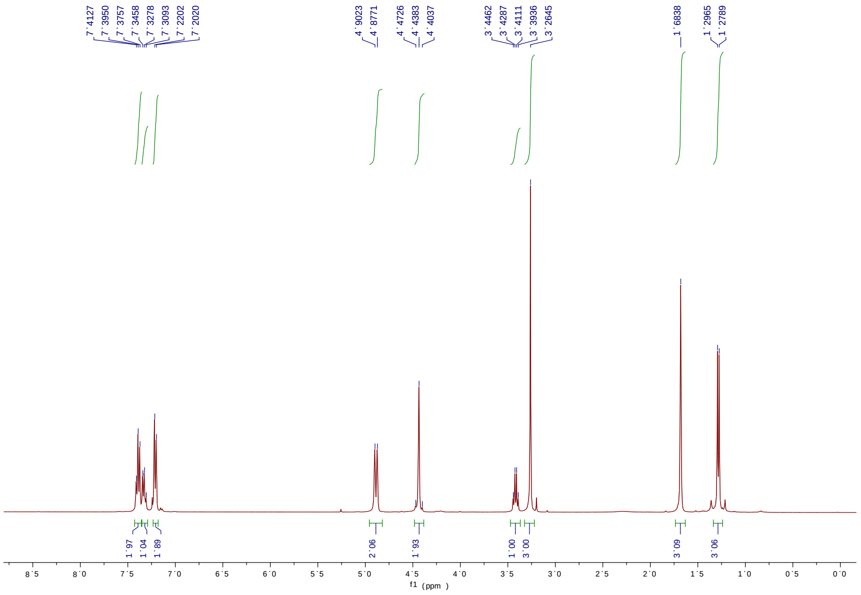

^{1}H NMR (400 MHz, $CDCl_3$) of compound (**10g**)

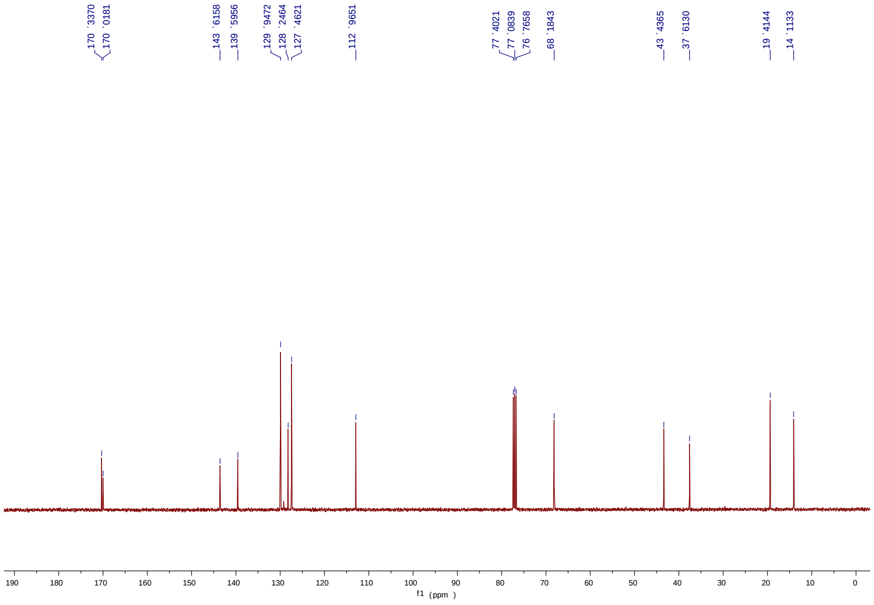

^{13}C NMR (100 MHz, $CDCl_3$) of compound (**10g**)

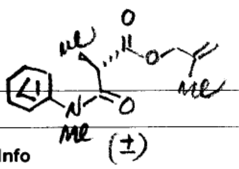

Display Report

Analysis Info

Analysis Name	D:\Data\user data\2013\APRIL\APR 01\Dr.A.Bisai-SG6-163_1-A,5_01_779.d	Acquisition Date	4/1/2013 4:23:09 PM
Method	HRLCMS-20 Sept.m	Operator	Amit
Sample Name	Dr.A.Bisai-SG6-163	Instrument	micrOTOF-Q II 10330
Comment			

Acquisition Parameter

Source Type	ESI	Ion Polarity	Positive	Set Nebulizer	1.2 Bar
Focus	Active	Set Capillary	4500 V	Set Dry Heater	200 °C
Scan Begin	50 m/z	Set End Plate Offset	-500 V	Set Dry Gas	7.0 l/min
Scan End	3000 m/z	Set Collision Cell RF	130.0 Vpp	Set Divert Valve	Waste

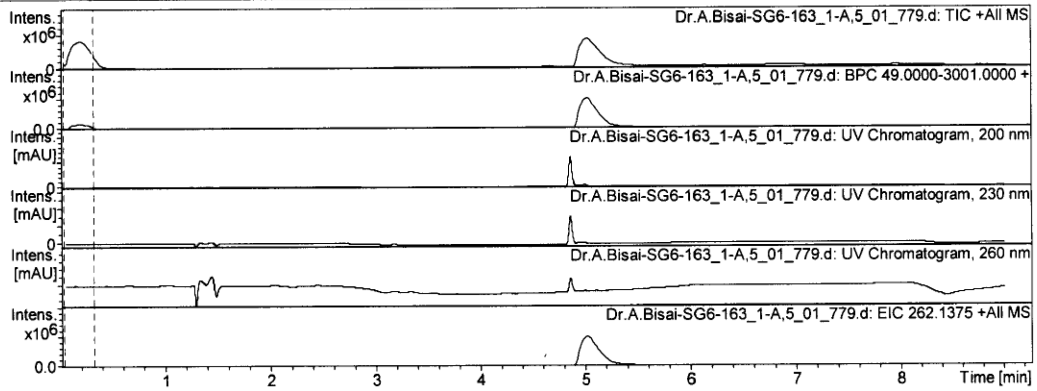

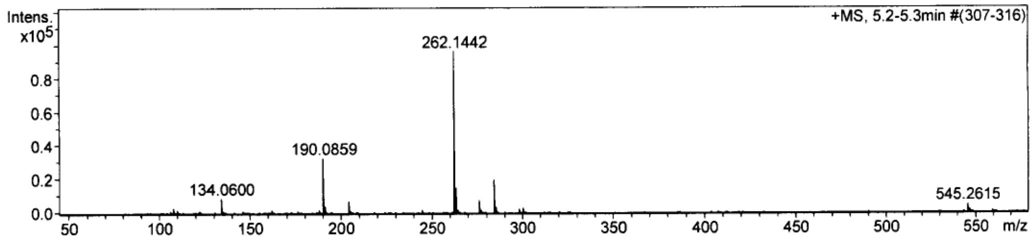

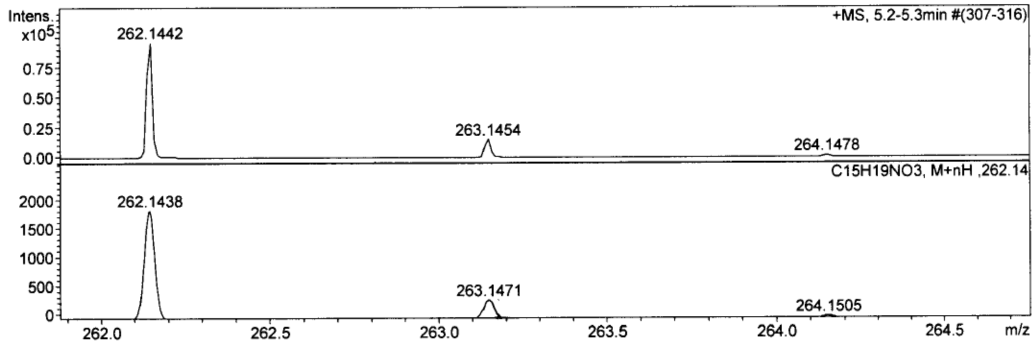

Bruker Compass DataAnalysis 4.0 printed: 4/1/2013 5:36:38 PM Page 1 of 1

Scanned copy of mass spectrum (HRMS) of (**10g**)

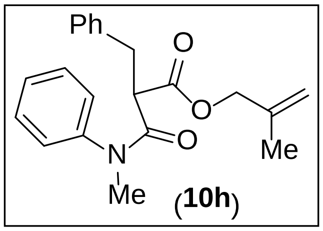

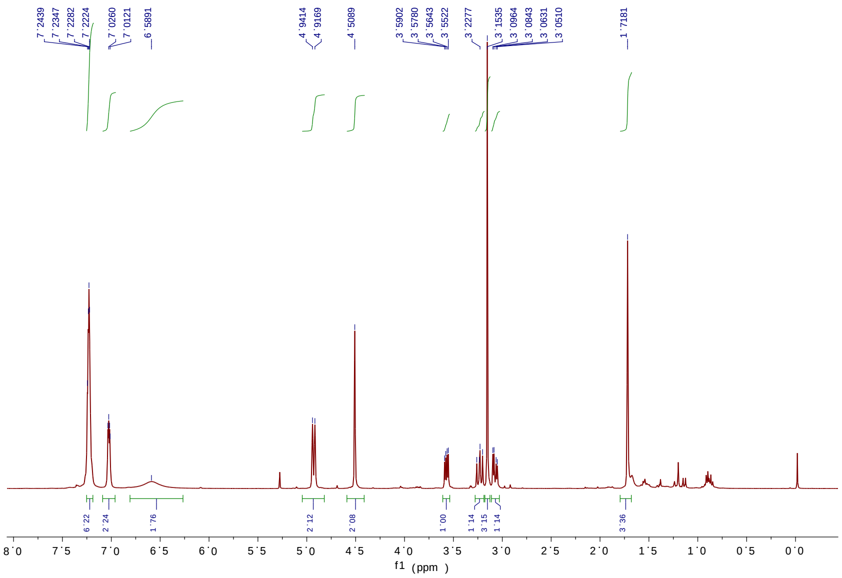

^{1}H NMR (400 MHz, $CDCl_3$) of compound (**10h**)

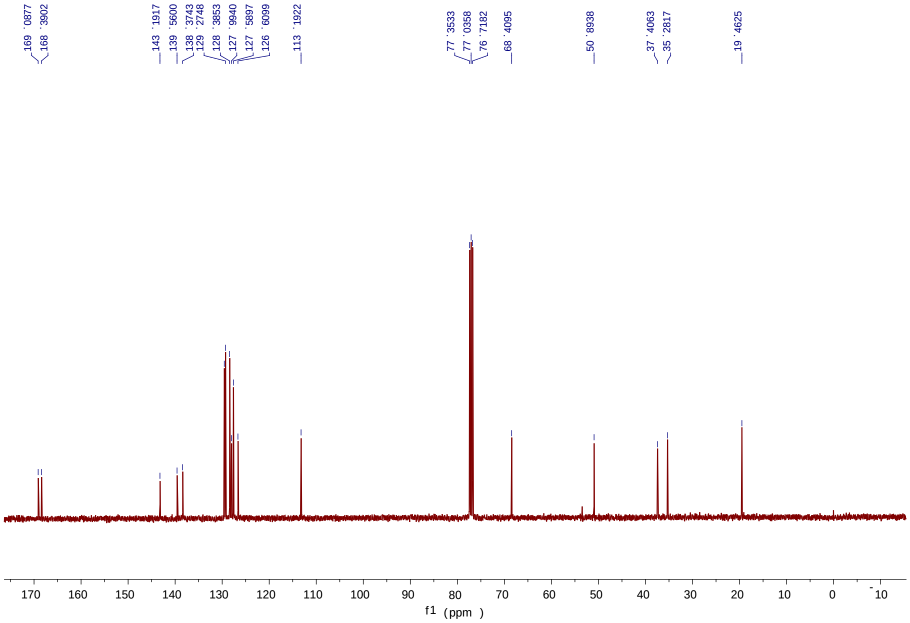

^{13}C NMR (100 MHz, $CDCl_3$) of compound (**10h**)

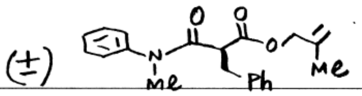

Display Report

Analysis Info

Analysis Name	D:\Data\user data\2013\MARCH\20 MAR\Dr.A.Bisai-SB3-56_1-A,8_01_688.d	Acquisition Date	3/20/2013 12:18:02 PM
Method	HRLCMS-20 Sept.m	Operator	Amit
Sample Name	Dr.A.Bisai-SB3-56	Instrument	micrOTOF-Q II 10330
Comment			

Acquisition Parameter

Source Type	ESI	Ion Polarity	Positive	Set Nebulizer	1.2 Bar
Focus	Active	Set Capillary	4500 V	Set Dry Heater	200 °C
Scan Begin	50 m/z	Set End Plate Offset	-500 V	Set Dry Gas	7.0 l/min
Scan End	3000 m/z	Set Collision Cell RF	130.0 Vpp	Set Divert Valve	Waste

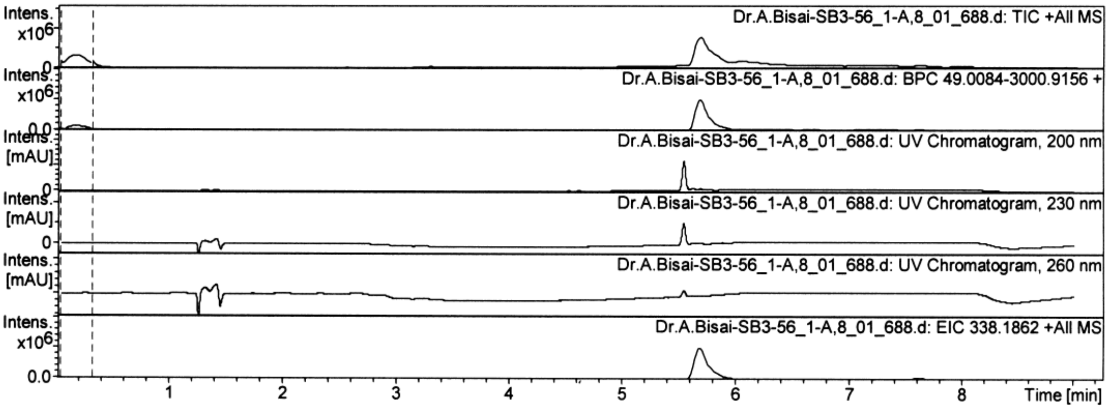

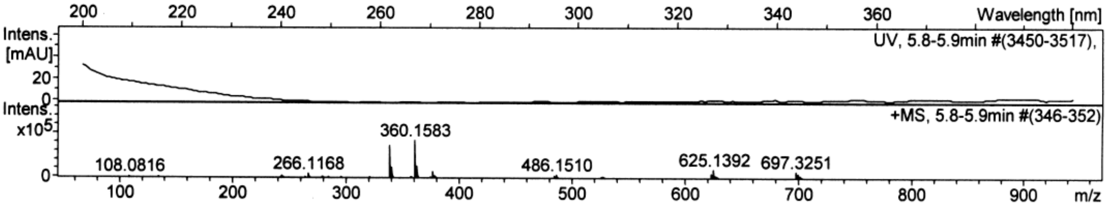

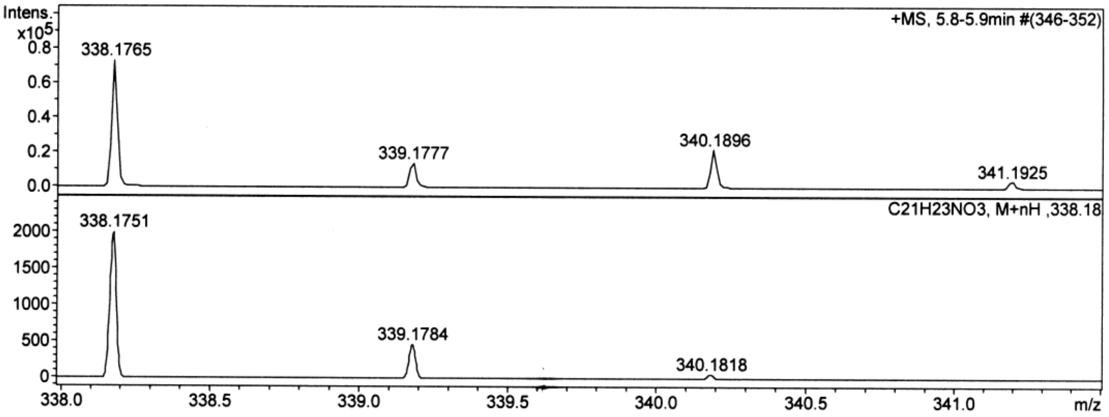

Scanned copy of mass spectrum of (**10h**)

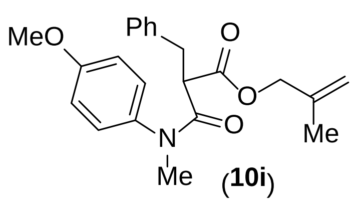

(**10i**)

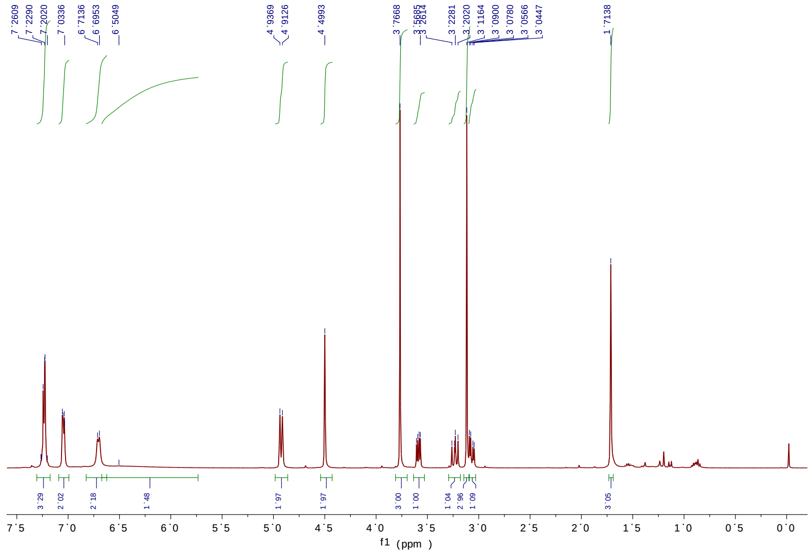

^{1}H NMR (400 MHz, $CDCl_3$) of compound (**10i**)

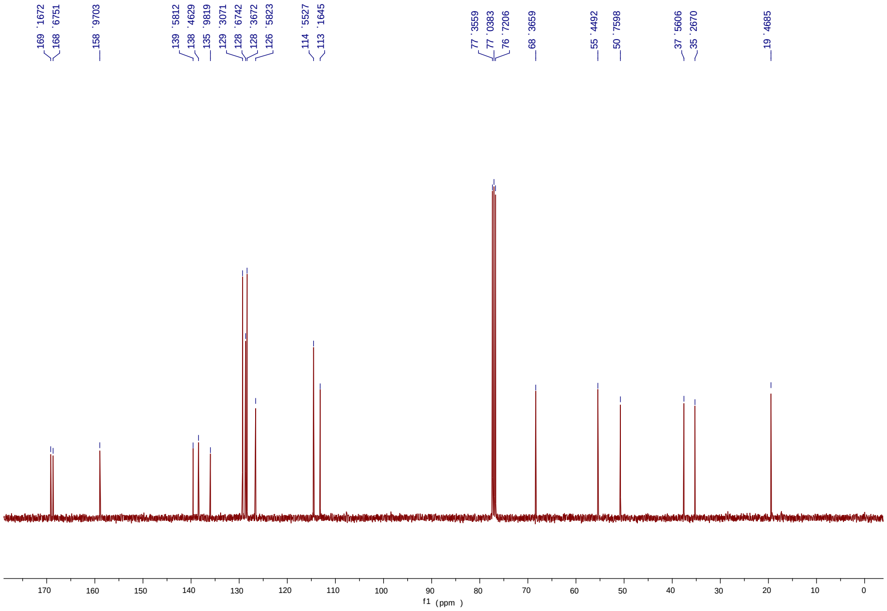

^{13}C NMR (100 MHz, $CDCl_3$) of compound (**10i**)

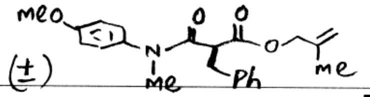

Display Report

Analysis Info

Analysis Name	D:\Data\user data\2013\MARCH\20 MAR\Dr.A.Bisai-SB3-60_1-B,4_01_692.d	Acquisition Date	3/20/2013 12:58:39 PM
Method	HRLCMS-20 Sept.m	Operator	Amit
Sample Name	Dr.A.Bisai-SB3-60	Instrument	micrOTOF-Q II 10330
Comment			

Acquisition Parameter

Source Type	ESI	Ion Polarity	Positive	Set Nebulizer	1.2 Bar
Focus	Active	Set Capillary	4500 V	Set Dry Heater	200 °C
Scan Begin	50 m/z	Set End Plate Offset	-500 V	Set Dry Gas	7.0 l/min
Scan End	3000 m/z	Set Collision Cell RF	130.0 Vpp	Set Divert Valve	Waste

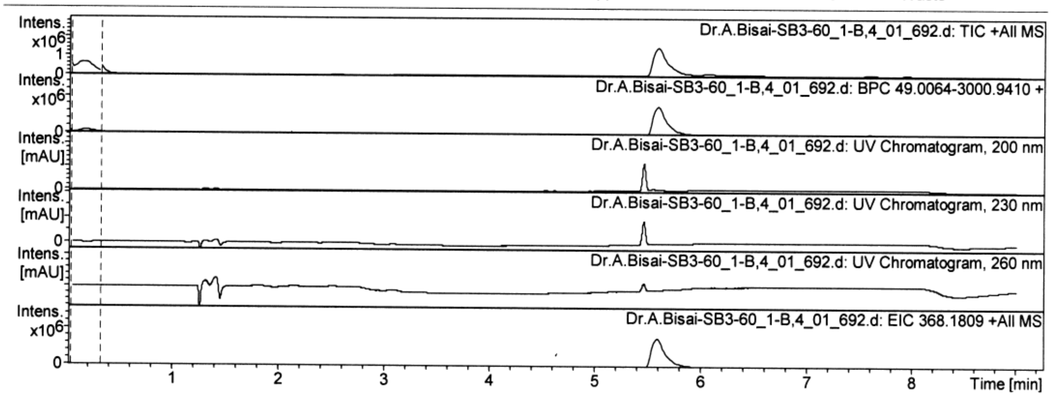

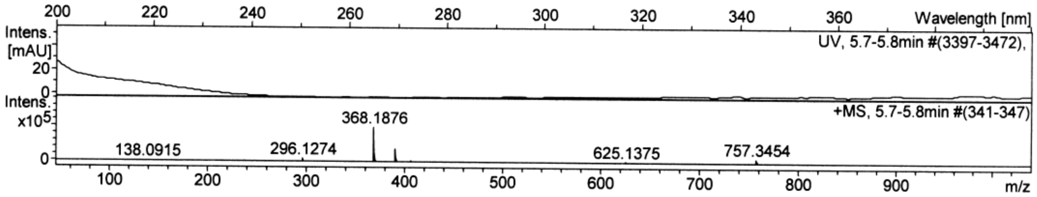

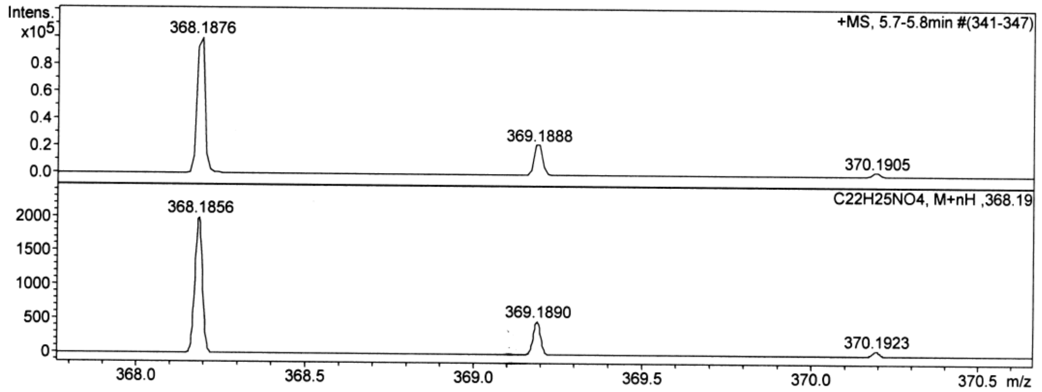

Bruker Compass DataAnalysis 4.0 printed: 3/20/2013 2:12:30 PM Page 1 of 1

Scanned copy of mass spectrum (HRMS) of (**10i**)

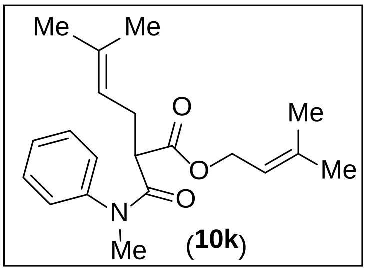

(**10k**)

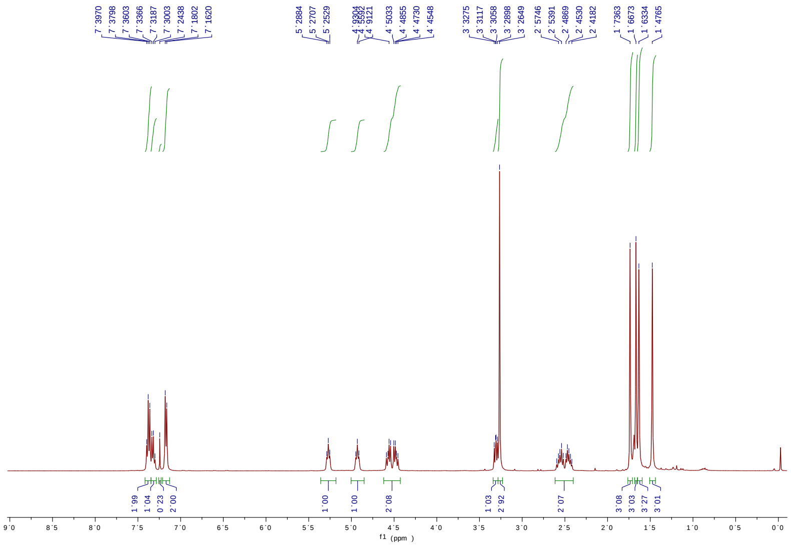

^{1}H NMR (400 MHz, $CDCl_3$) of compound (**10k**)

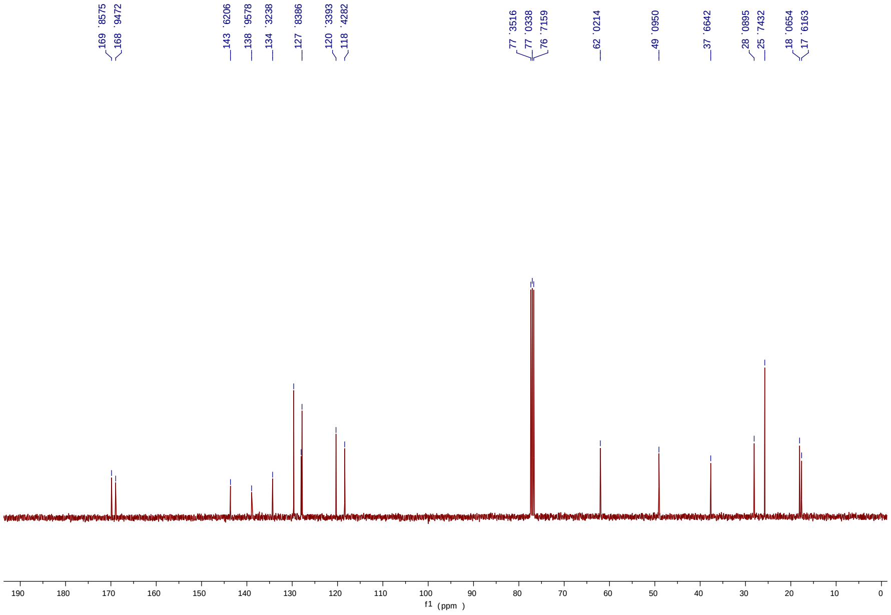

^{13}C NMR (100 MHz, $CDCl_3$) of compound (**10k**)

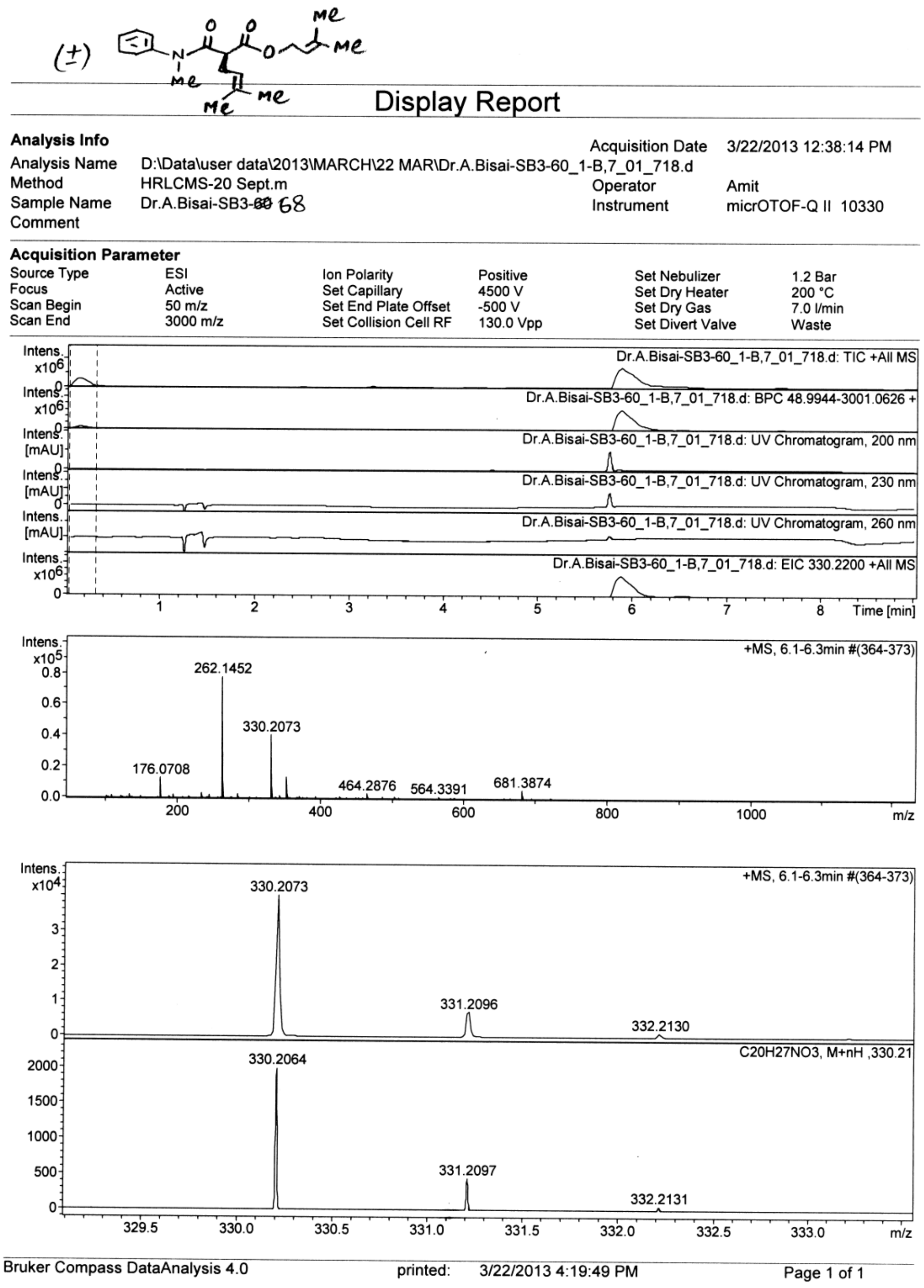

Scanned copy of mass spectrum (HRMS) of (**10k**)

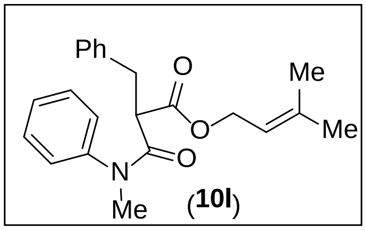

(**10l**)

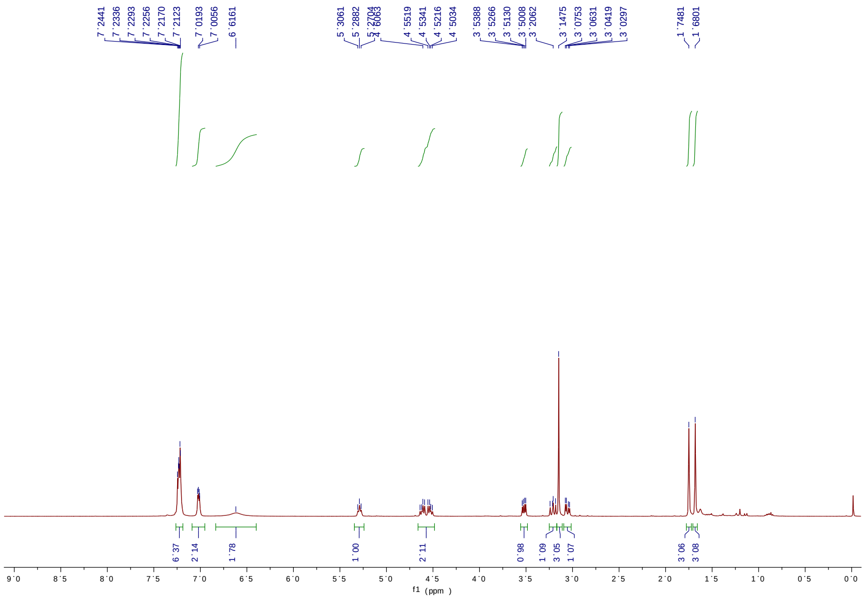

^{1}H NMR (400 MHz, $CDCl_3$) of compound (**10l**)

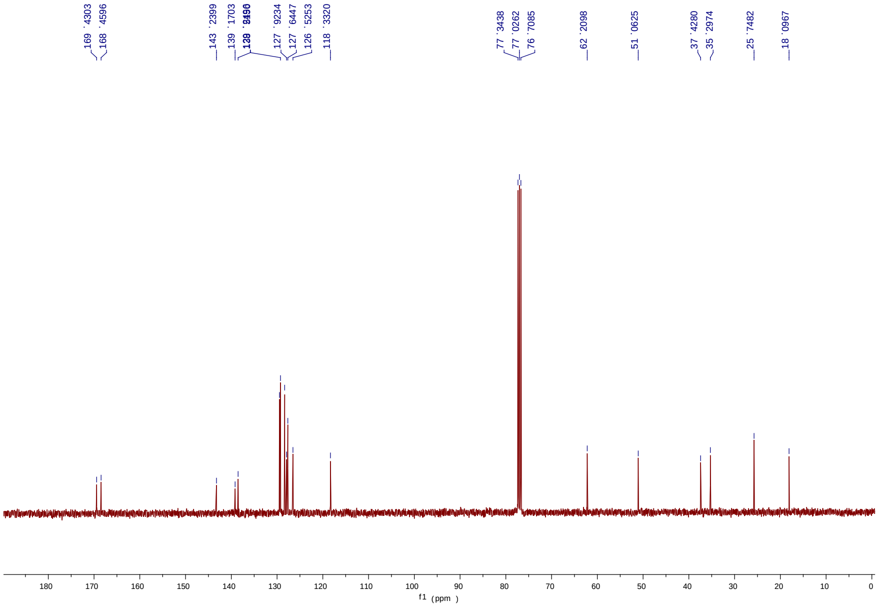

^{13}C NMR (100 MHz, $CDCl_3$) of compound (**10l**)

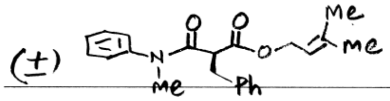

Display Report

Analysis Info

Analysis Name	D:\Data\user data\2013\MARCH\20 MAR\Dr.A.Bisai-SB3-58_1-B,2_01_690.d	Acquisition Date	3/20/2013 12:38:24 PM
Method	HRLCMS-20 Sept.m	Operator	Amit
Sample Name	Dr.A.Bisai-SB3-58	Instrument	micrOTOF-Q II 10330
Comment			

Acquisition Parameter

Source Type	ESI	Ion Polarity	Positive	Set Nebulizer	1.2 Bar
Focus	Active	Set Capillary	4500 V	Set Dry Heater	200 °C
Scan Begin	50 m/z	Set End Plate Offset	-500 V	Set Dry Gas	7.0 l/min
Scan End	3000 m/z	Set Collision Cell RF	130.0 Vpp	Set Divert Valve	Waste

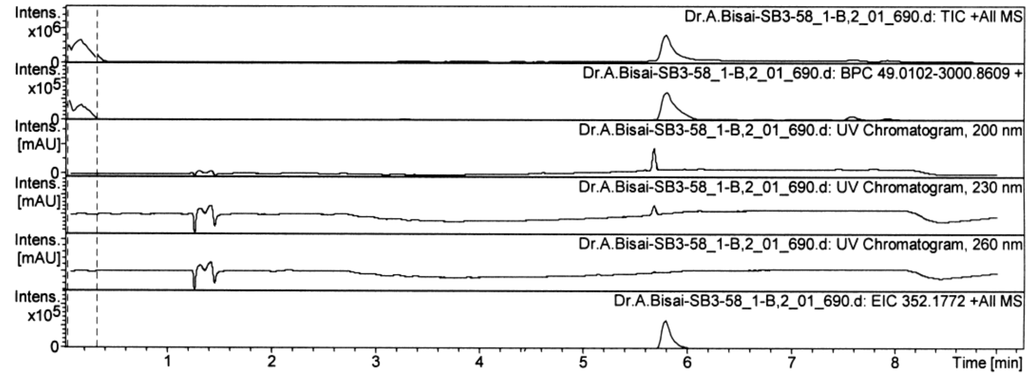

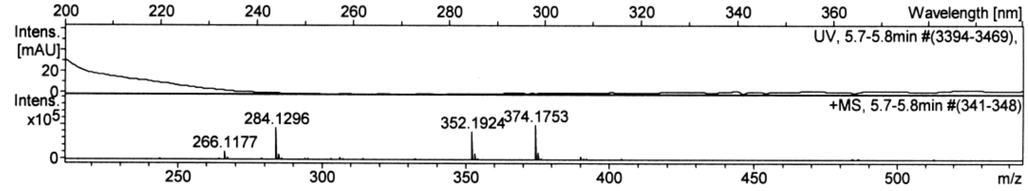

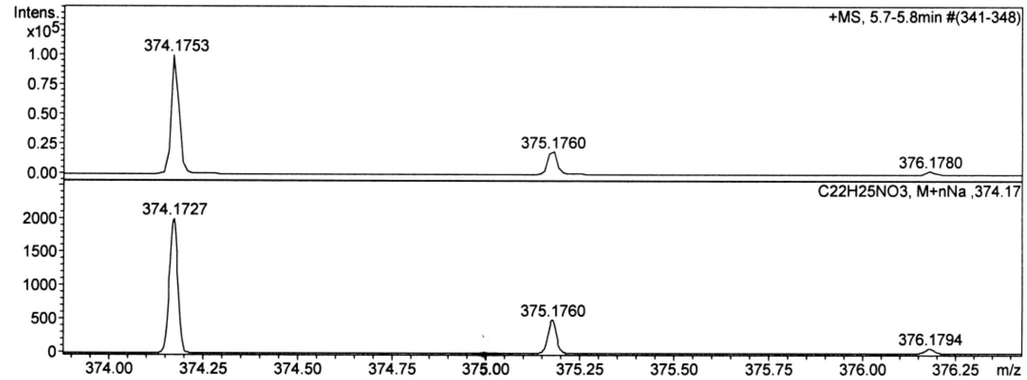

Bruker Compass DataAnalysis 4.0 printed: 3/20/2013 2:06:01 PM Page 1 of 1

Scanned copy of mass spectrum (HRMS) of (**10l**)

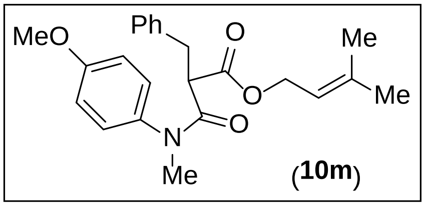

(**10m**)

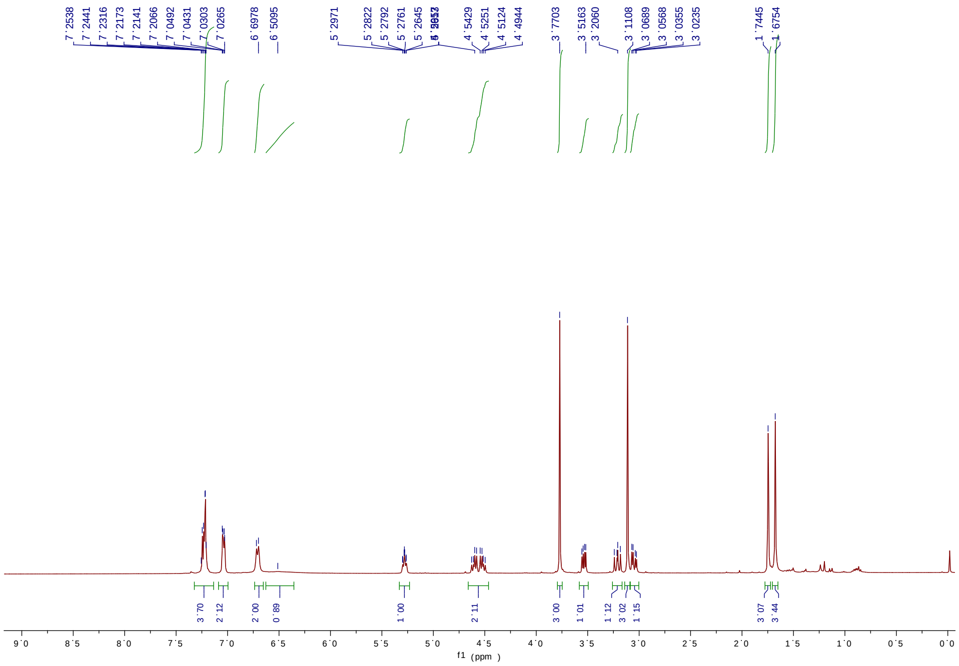

^{1}H NMR (400 MHz, $CDCl_3$) of compound (**10m**)

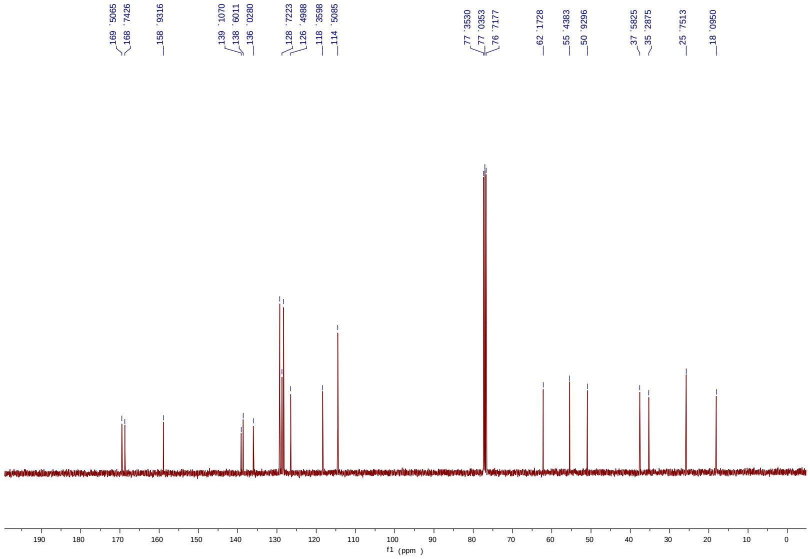

^{13}C NMR (100 MHz, $CDCl_3$) of compound (**10m**)

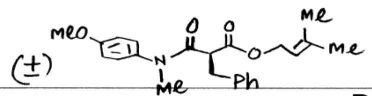

Display Report

Analysis Info

Analysis Name	D:\Data\user data\2013\MARCH\20 MAR\Dr.A.Bisai-SB3-57_1-B,1_01_689.d	Acquisition Date	3/20/2013 12:28:12 PM
Method	HRLCMS-20 Sept.m	Operator	Amit
Sample Name	Dr.A.Bisai-SB3-57	Instrument	micrOTOF-Q II 10330
Comment			

Acquisition Parameter

Source Type	ESI	Ion Polarity	Positive	Set Nebulizer	1.2 Bar
Focus	Active	Set Capillary	4500 V	Set Dry Heater	200 °C
Scan Begin	50 m/z	Set End Plate Offset	-500 V	Set Dry Gas	7.0 l/min
Scan End	3000 m/z	Set Collision Cell RF	130.0 Vpp	Set Divert Valve	Waste

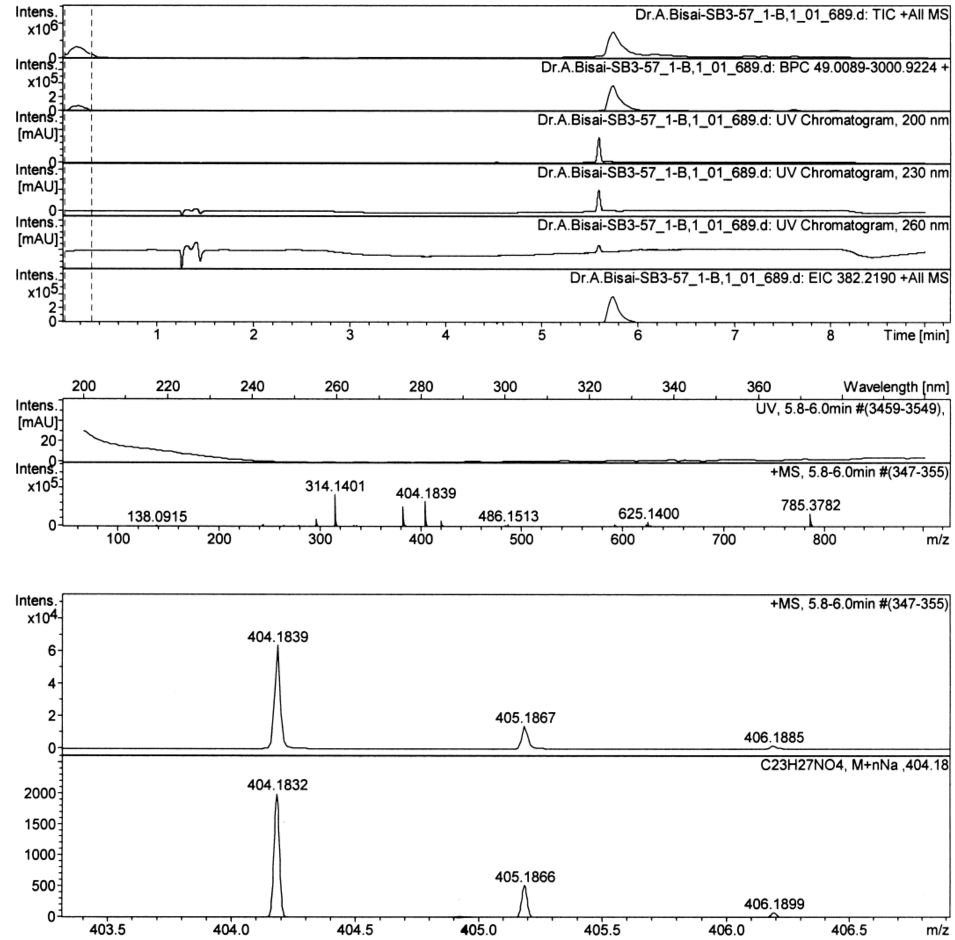

Bruker Compass DataAnalysis 4.0 printed: 3/20/2013 12:53:17 PM Page 1 of 1

Scanned copy of mass spectrum (HRMS) of (**10m**)

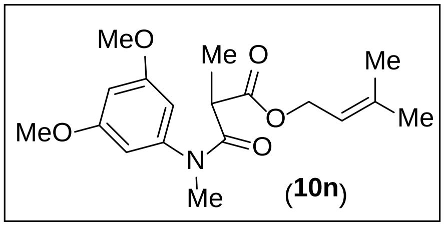

(**10n**)

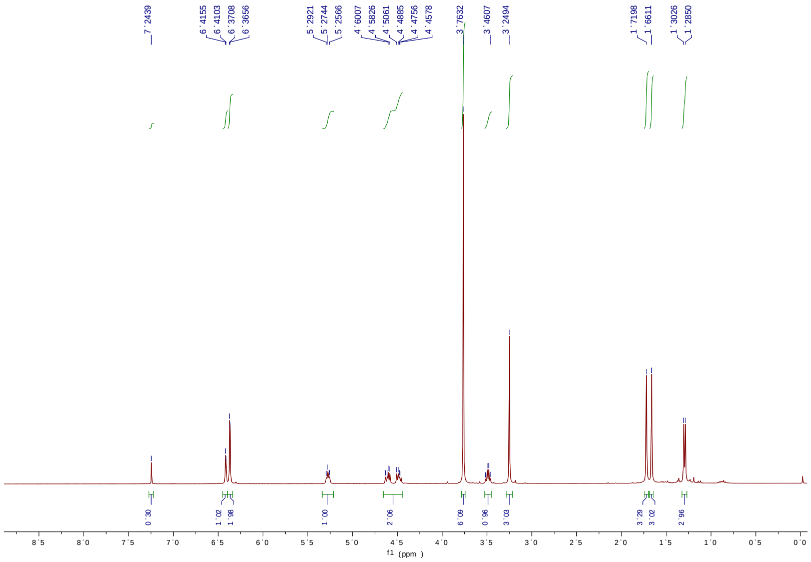

^{1}H NMR (400 MHz, $CDCl_3$) of compound (**10n**)

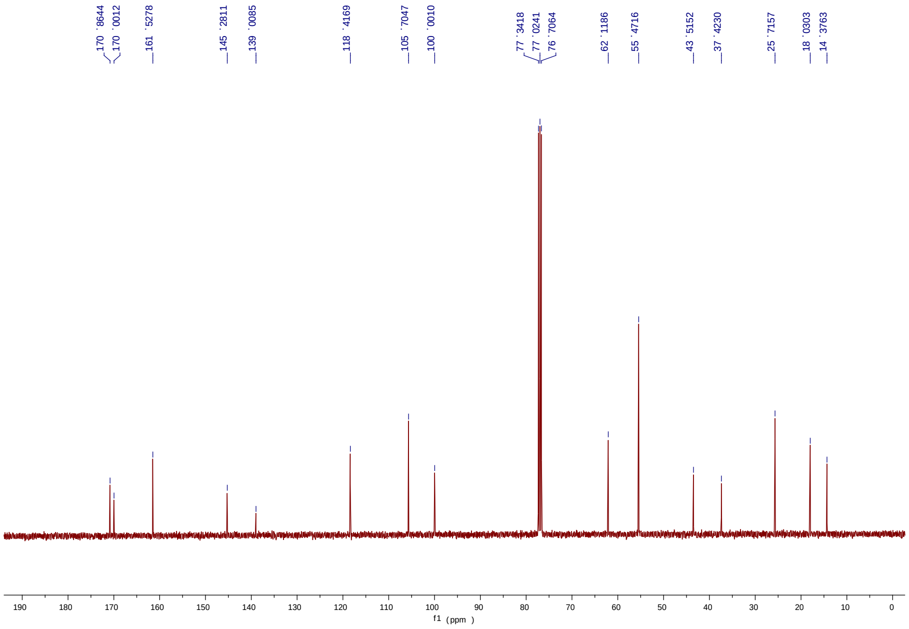

^{13}C NMR (100 MHz, $CDCl_3$) of compound (**10n**)

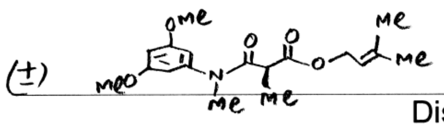

Display Report

Analysis Info

Analysis Name	D:\Data\user data\2013\MARCH\22 MAR\Dr.A.Bisai-SB3-71_1-D,5_01_733.d	Acquisition Date	3/22/2013 3:19:59 PM
Method	HRLCMS-20 Sept.m	Operator	Amit
Sample Name	Dr.A.Bisai-SB3-71	Instrument	micrOTOF-Q II 10330
Comment			

Acquisition Parameter

Source Type	ESI	Ion Polarity	Positive	Set Nebulizer	1.2 Bar
Focus	Active	Set Capillary	4500 V	Set Dry Heater	200 °C
Scan Begin	50 m/z	Set End Plate Offset	-500 V	Set Dry Gas	7.0 l/min
Scan End	3000 m/z	Set Collision Cell RF	130.0 Vpp	Set Divert Valve	Waste

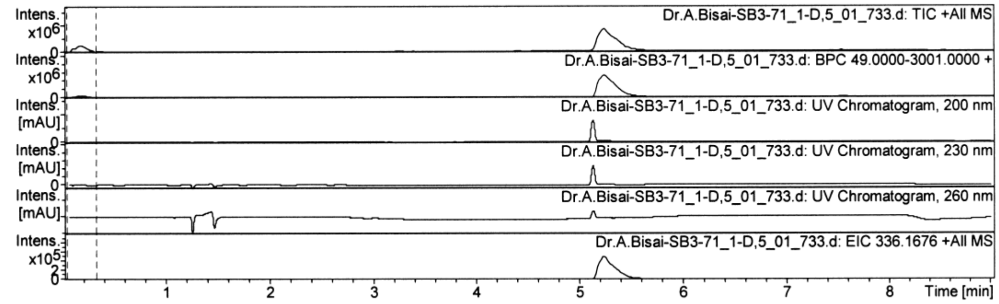

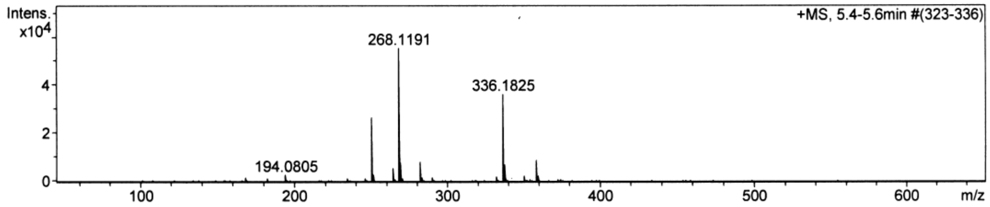

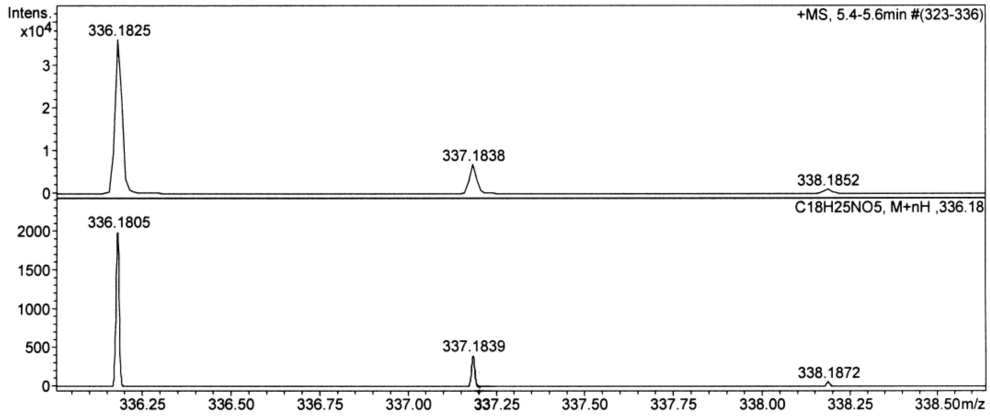

Bruker Compass DataAnalysis 4.0 printed: 3/22/2013 4:30:52 PM Page 1 of 1

Scanned copy of mass spectrum (HRMS) of (**10n**)

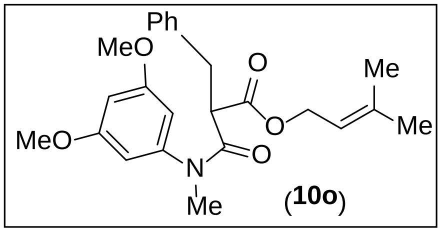

(**10o**)

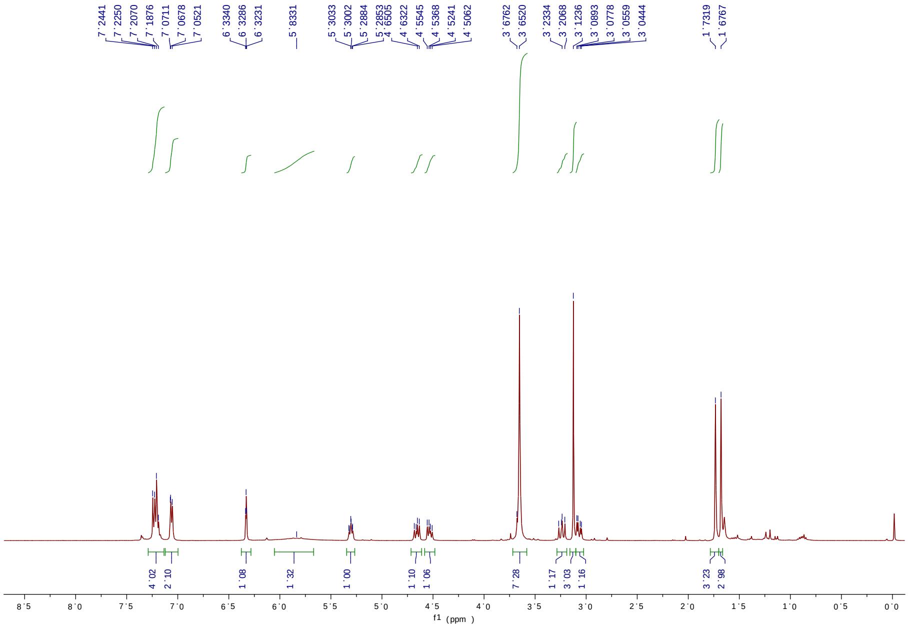

^{1}H NMR (400 MHz, $CDCl_3$) of compound (**10o**)

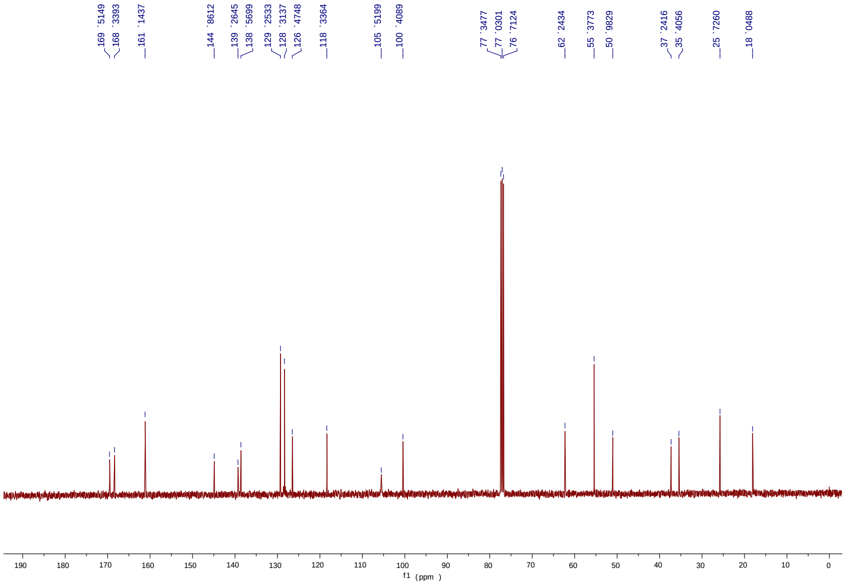

^{13}C NMR (100 MHz, $CDCl_3$) of compound (**10o**)

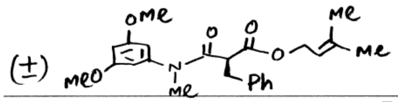

Display Report

Analysis Info

Analysis Name	D:\Data\user data\2013\MARCH\20 MAR\Dr.A.Bisai-SB3-59_1-B,3_01_691.d	Acquisition Date	3/20/2013 12:48:31 PM
Method	HRLCMS-20 Sept.m	Operator	Amit
Sample Name	Dr.A.Bisai-SB3-59	Instrument	micrOTOF-Q II 10330
Comment			

Acquisition Parameter

Source Type	ESI	Ion Polarity	Positive	Set Nebulizer	1.2 Bar
Focus	Active	Set Capillary	4500 V	Set Dry Heater	200 °C
Scan Begin	50 m/z	Set End Plate Offset	-500 V	Set Dry Gas	7.0 l/min
Scan End	3000 m/z	Set Collision Cell RF	130.0 Vpp	Set Divert Valve	Waste

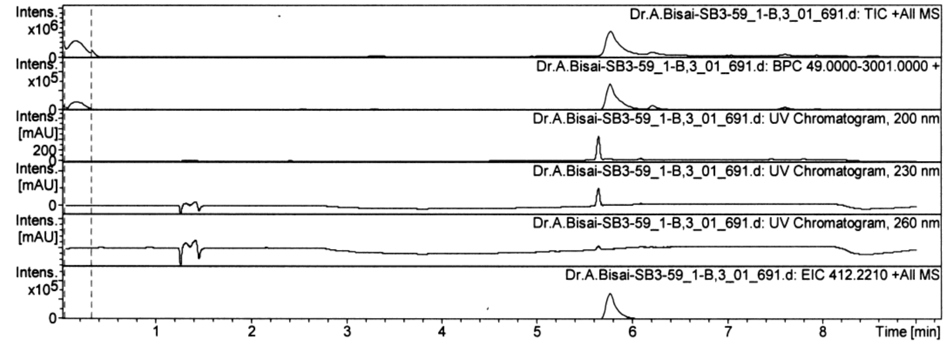

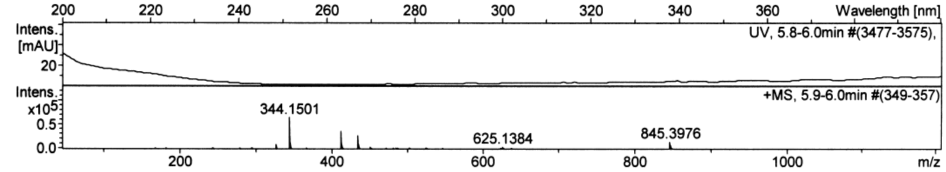

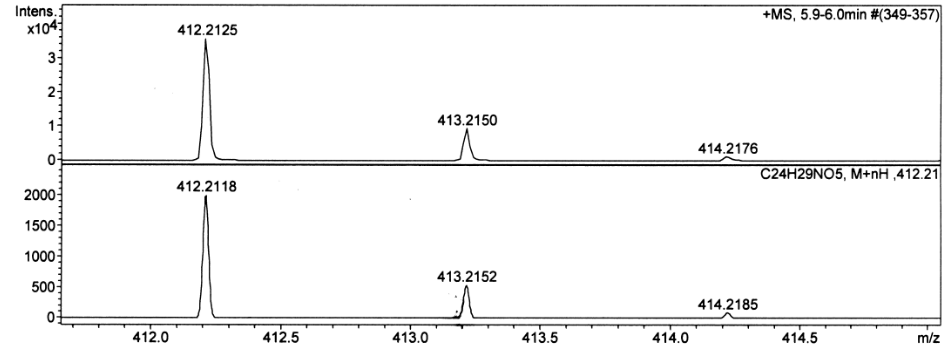

Bruker Compass DataAnalysis 4.0 printed: 3/20/2013 2:08:59 PM Page 1 of 1

Scanned copy of mass spectrum (HRMS) of (**10o**)

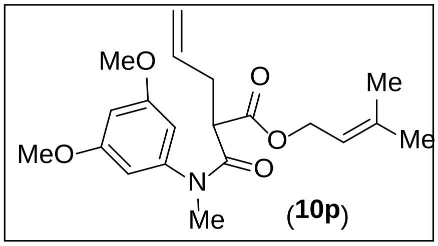

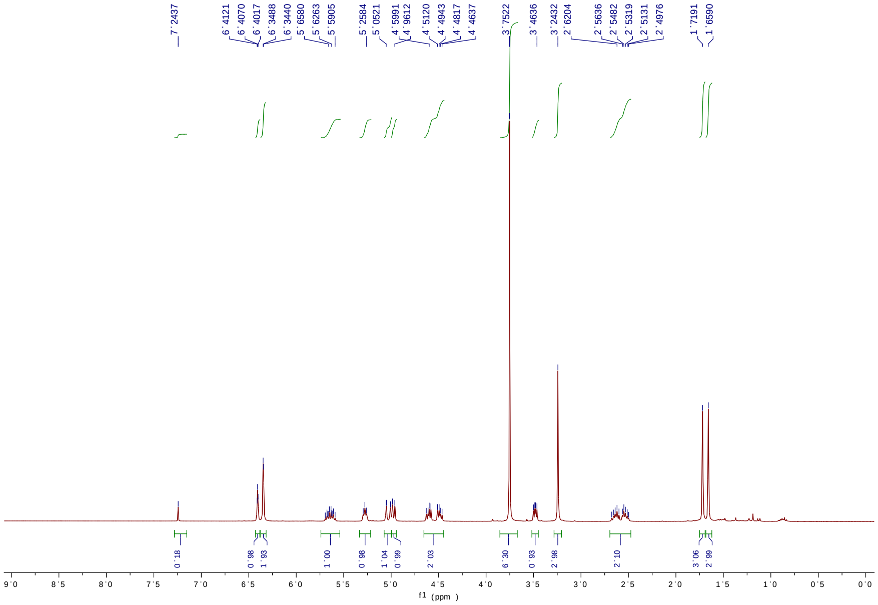

^{1}H NMR (400 MHz, CDCl$_3$) of compound (**10p**)

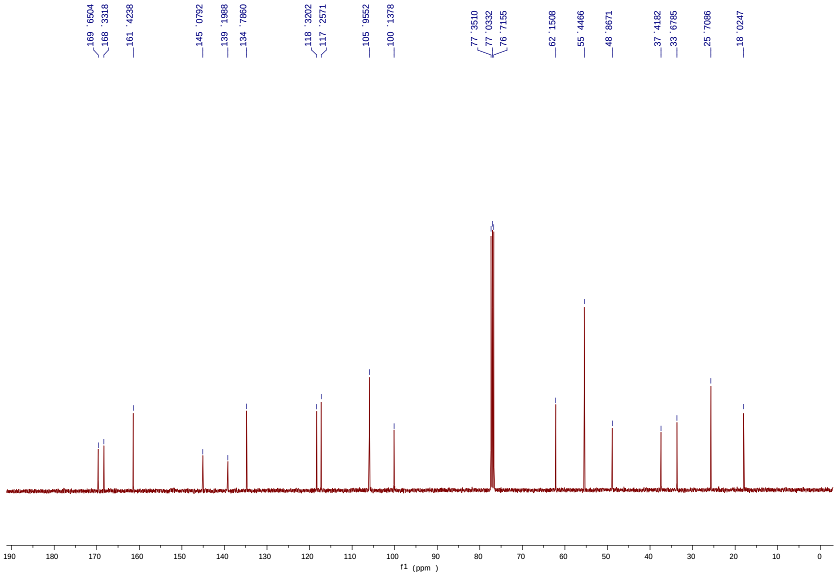

^{13}C NMR (100 MHz, CDCl$_3$) of compound (**10p**)

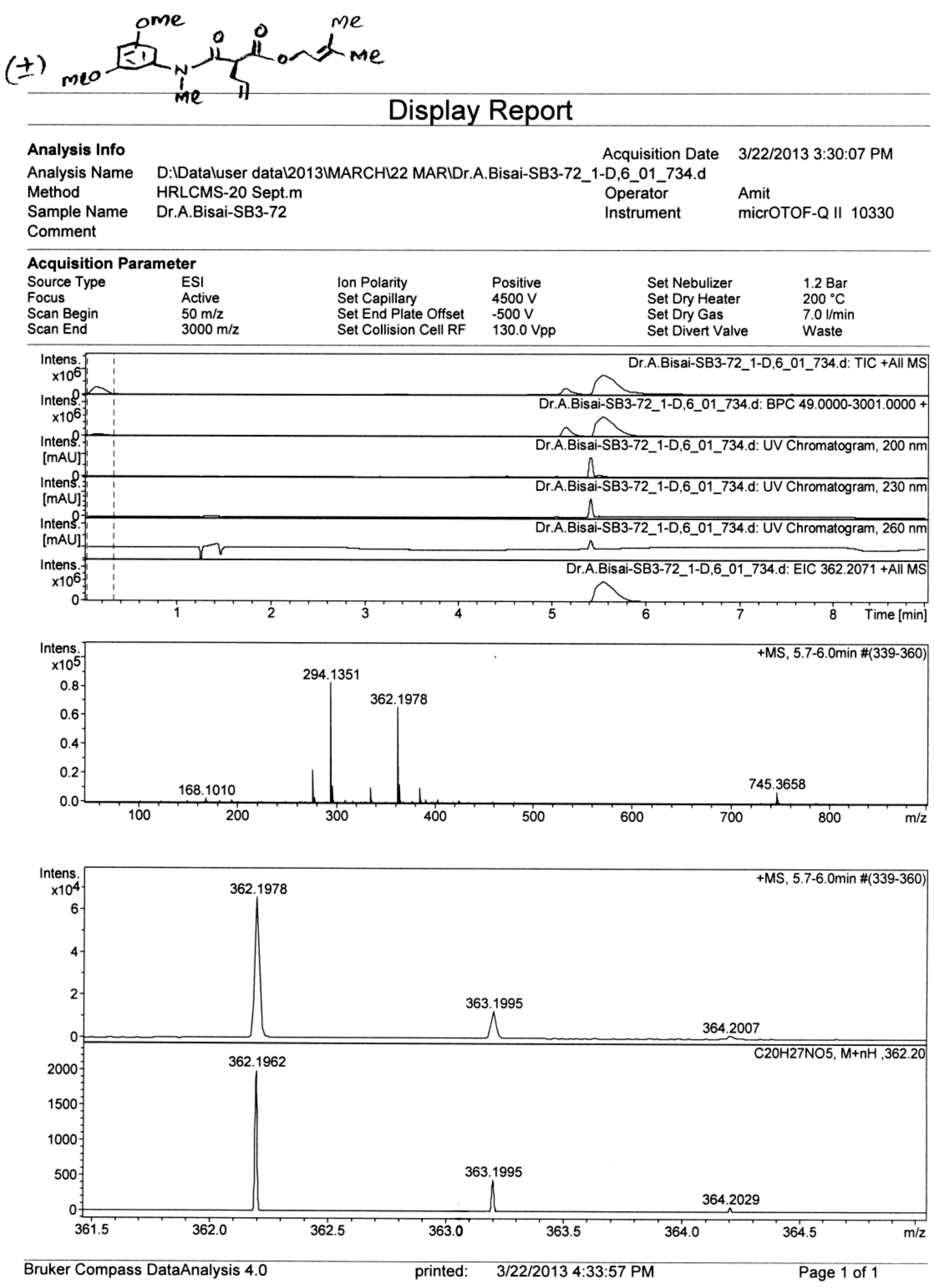

Scanned copy of mass spectrum (HRMS) of (**10p**)

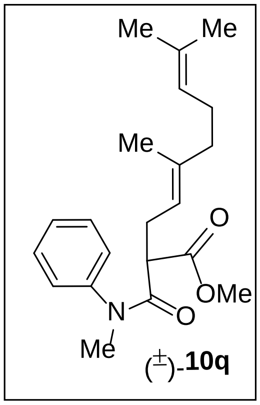

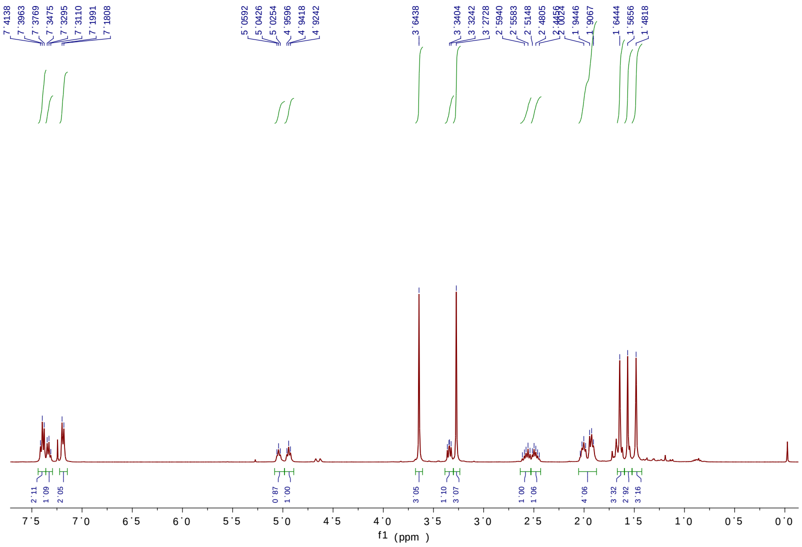

^{1}H NMR (400 MHz, $CDCl_3$) of compound (**10q**)

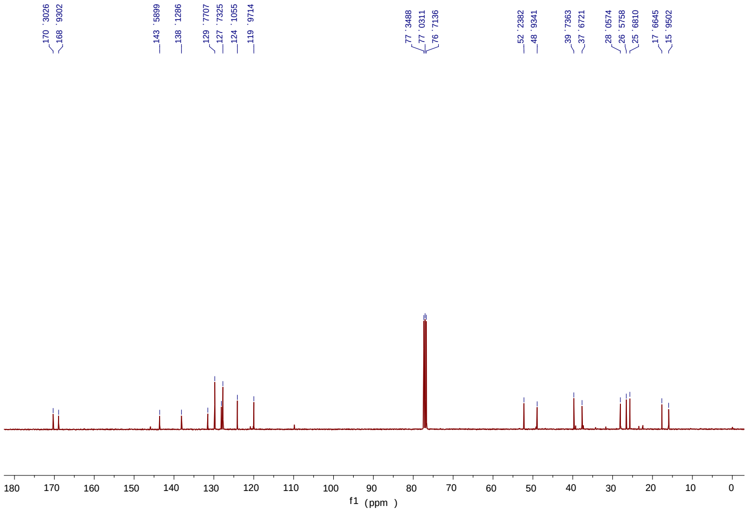

^{13}C NMR (100 MHz, $CDCl_3$) of compound (**10q**)

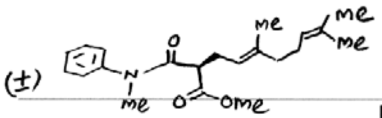

Display Report

Analysis Info

Analysis Name	D:\Data\user data\2013\MARCH\16 MAR\Dr.A.Bisai-SB3-40_1-B,2_01_632.d	Acquisition Date	3/16/2013 5:11:43 PM
Method	HRLCMS-20 Sept.m	Operator	Amit
Sample Name	Dr.A.Bisai-SB3-40	Instrument	micrOTOF-Q II 10330
Comment			

Acquisition Parameter

Source Type	ESI	Ion Polarity	Positive	Set Nebulizer	1.2 Bar
Focus	Active	Set Capillary	4500 V	Set Dry Heater	200 °C
Scan Begin	50 m/z	Set End Plate Offset	-500 V	Set Dry Gas	7.0 l/min
Scan End	3000 m/z	Set Collision Cell RF	130.0 Vpp	Set Divert Valve	Waste

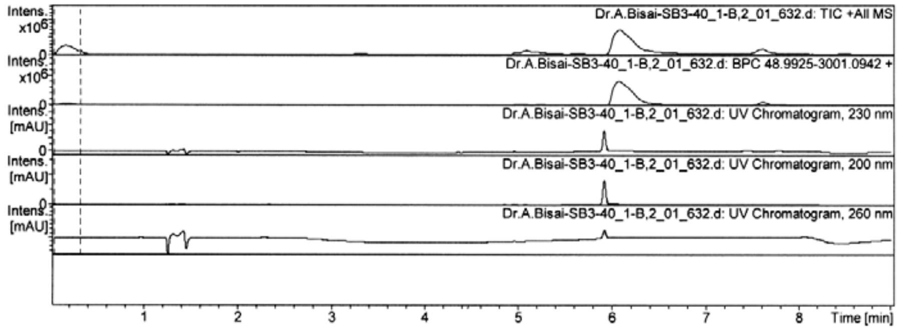

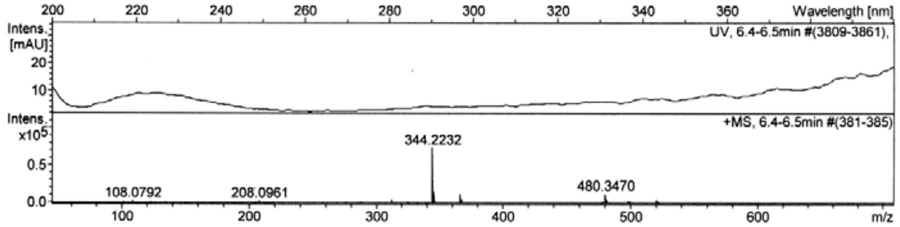

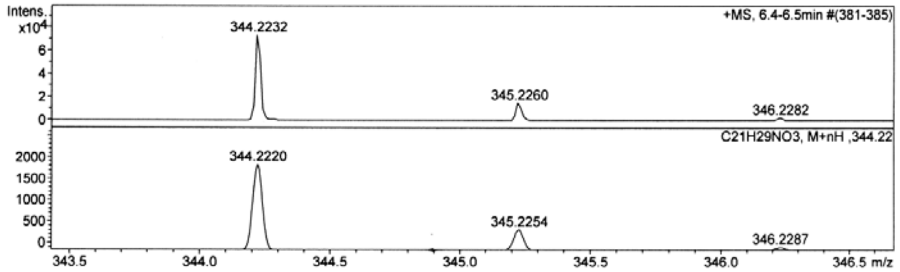

Bruker Compass DataAnalysis 4.0 printed: 3/16/2013 7:16:06 PM Page 1 of 1

Scanned copy of mass spectrum (HRMS) of (**10q**)

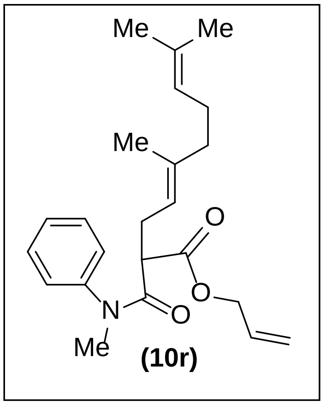

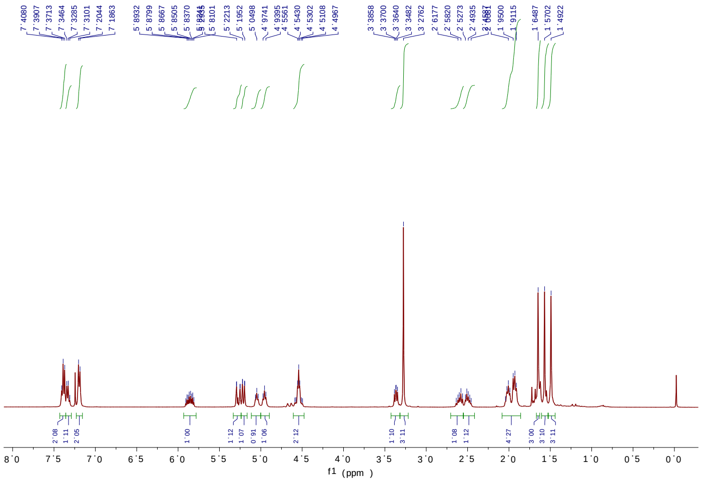

^{1}H NMR (400 MHz, $CDCl_3$) of compound (**10r**)

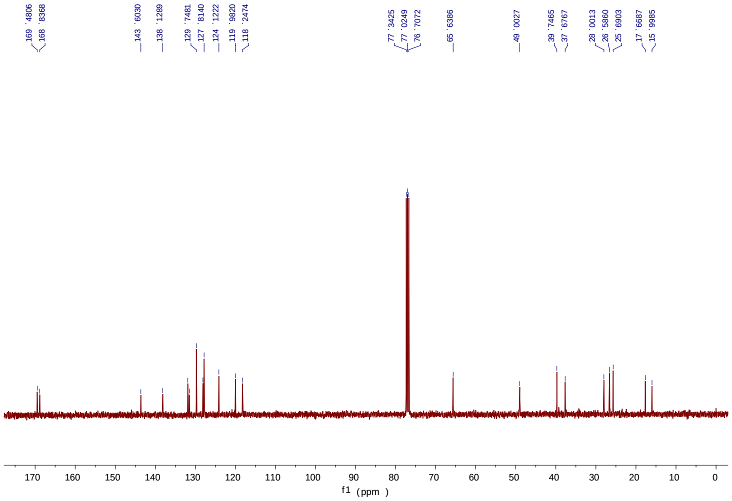

^{13}C NMR (100 MHz, $CDCl_3$) of compound (**10r**)

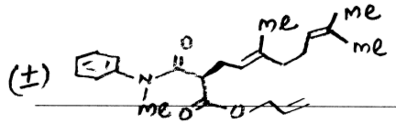

Display Report

Analysis Info

Analysis Name	D:\Data\user data\2013\MARCH\16 MAR\Dr.A.Bisai-SB3-42_1-B,4_01_634.d	Acquisition Date	3/16/2013 5:32:01 PM
Method	HRLCMS-20 Sept.m	Operator	Amit
Sample Name	Dr.A.Bisai-SB3-42	Instrument	micrOTOF-Q II 10330
Comment			

Acquisition Parameter

Source Type	ESI	Ion Polarity	Positive	Set Nebulizer	1.2 Bar
Focus	Active	Set Capillary	4500 V	Set Dry Heater	200 °C
Scan Begin	50 m/z	Set End Plate Offset	-500 V	Set Dry Gas	7.0 l/min
Scan End	3000 m/z	Set Collision Cell RF	130.0 Vpp	Set Divert Valve	Waste

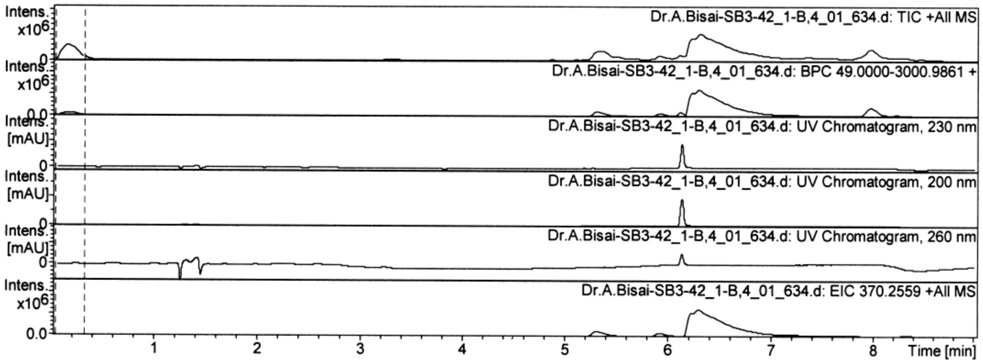

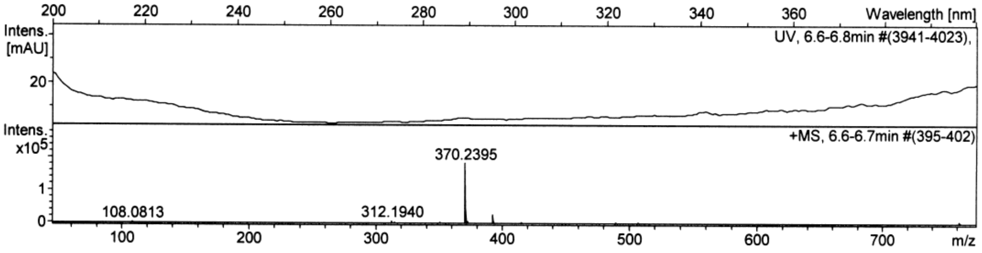

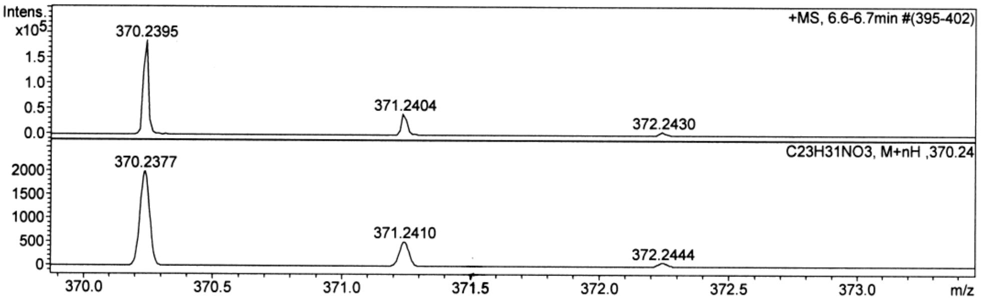

Bruker Compass DataAnalysis 4.0 printed: 3/16/2013 7:30:33 PM Page 1 of 1

Scanned copy of mass spectrum (HRMS) of (**10r**)

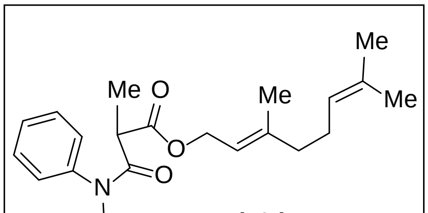

(10t)

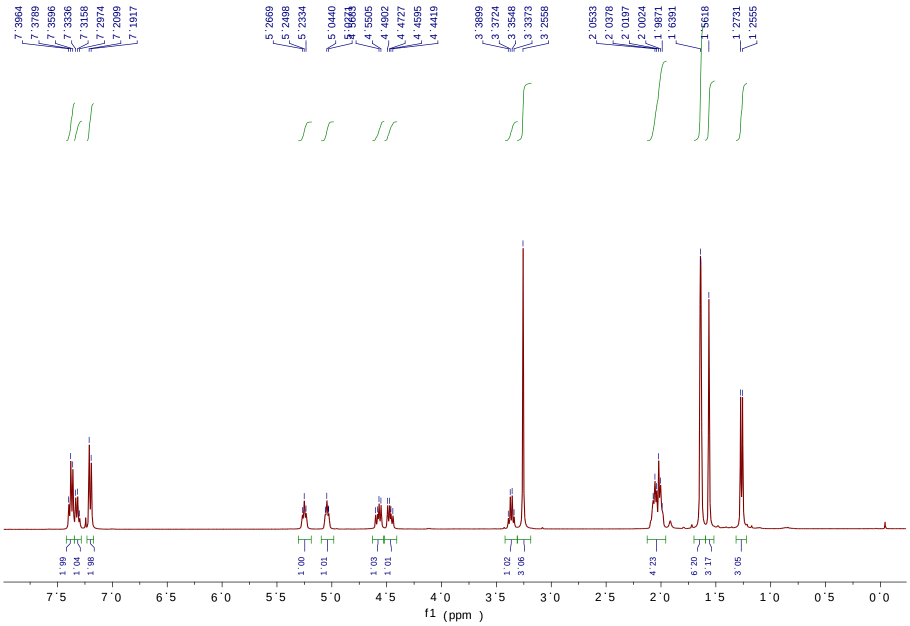

^{1}H NMR (400 MHz, $CDCl_3$) of compound (**10t**)

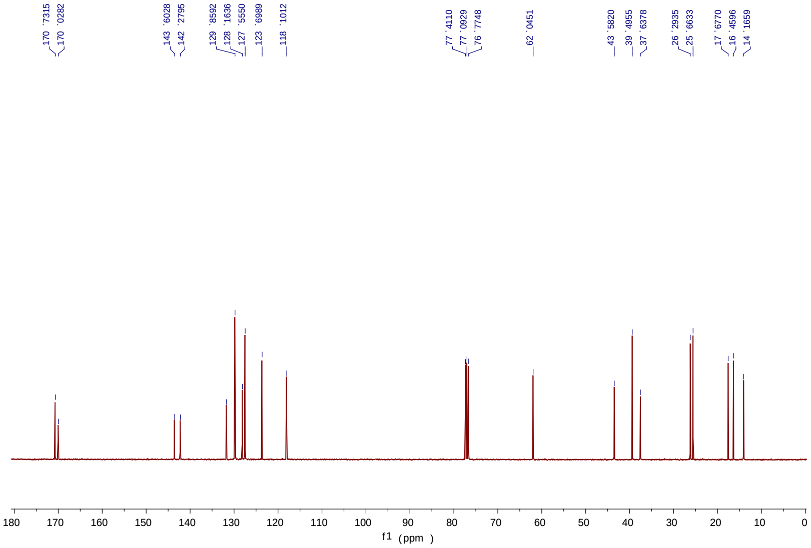

^{13}C NMR (100 MHz, $CDCl_3$) of compound (**10t**)

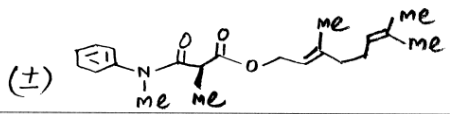

Display Report

Analysis Info

Analysis Name	D:\Data\user data\2013\MARCH\22 MAR\Dr.A.Bisai-SB3-46_1-B,4_01_715.d	Acquisition Date	3/22/2013 12:07:43 PM
Method	HRLCMS-20 Sept.m	Operator	Amit
Sample Name	Dr.A.Bisai-SB3-46	Instrument	micrOTOF-Q II 10330
Comment			

Acquisition Parameter

Source Type	ESI	Ion Polarity	Positive	Set Nebulizer	1.2 Bar
Focus	Active	Set Capillary	4500 V	Set Dry Heater	200 °C
Scan Begin	50 m/z	Set End Plate Offset	-500 V	Set Dry Gas	7.0 l/min
Scan End	3000 m/z	Set Collision Cell RF	130.0 Vpp	Set Divert Valve	Waste

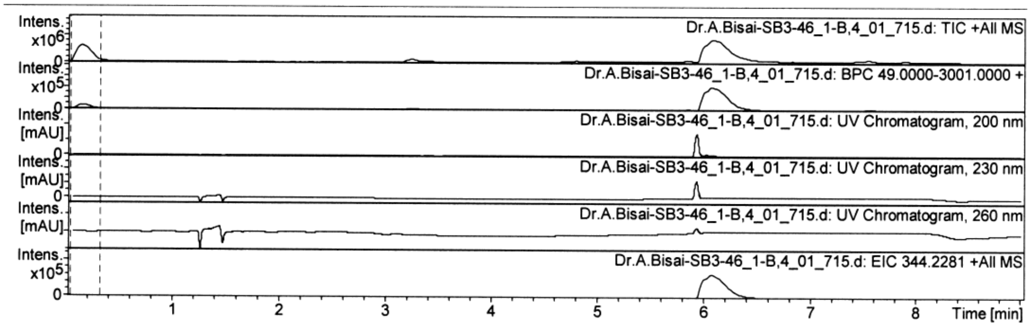

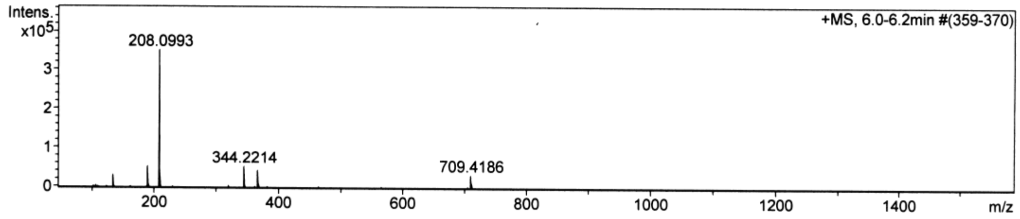

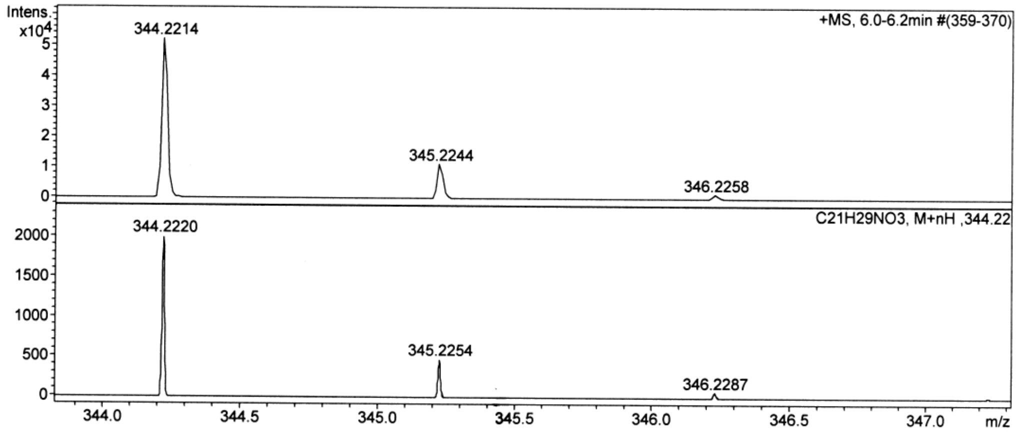

Bruker Compass DataAnalysis 4.0 printed: 3/22/2013 4:12:55 PM Page 1 of 1

Scanned copy of mass spectrum (HRMS) of (**10t**)

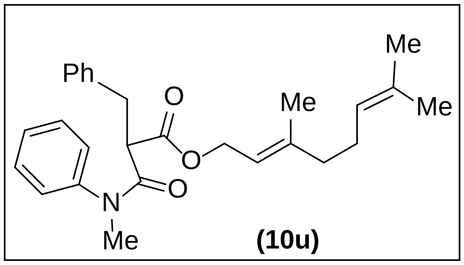

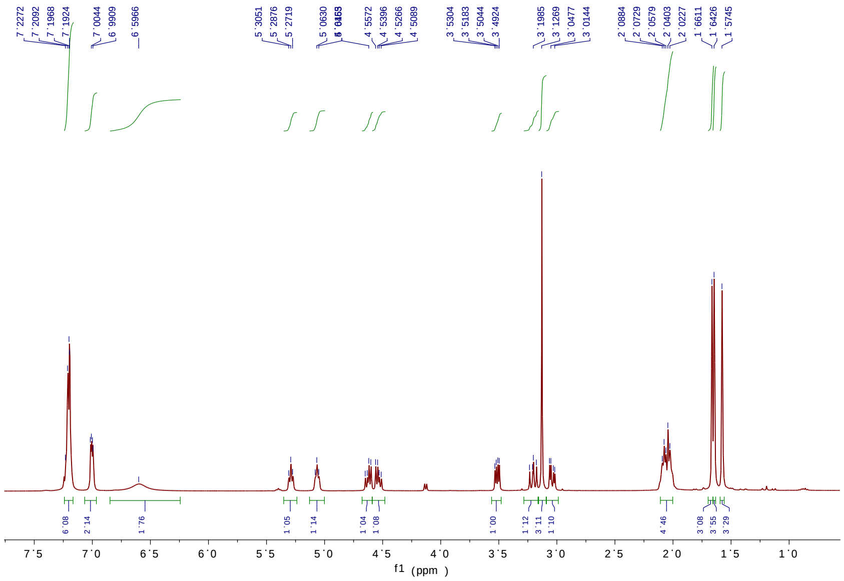

^{1}H NMR (400 MHz, $CDCl_3$) of compound (**10u**)

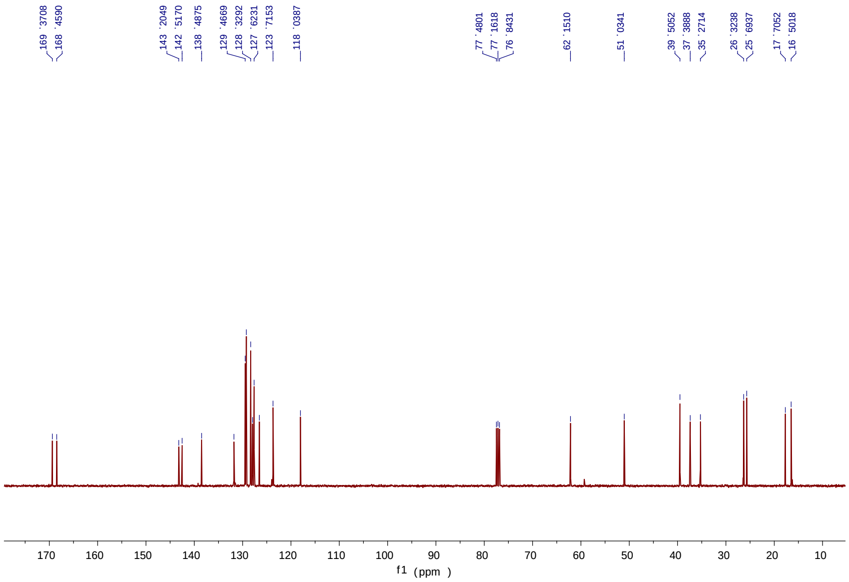

^{13}C NMR (100 MHz, $CDCl_3$) of compound (**10u**)

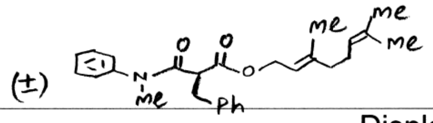

Display Report

Analysis Info

Analysis Name	D:\Data\user data\2013\MARCH\22 MAR\Dr.A.Bisai-SB3-47_1-B,5_01_716.d	Acquisition Date	3/22/2013 12:17:52 PM
Method	HRLCMS-20 Sept.m	Operator	Amit
Sample Name	Dr.A.Bisai-SB3-47	Instrument	micrOTOF-Q II 10330
Comment			

Acquisition Parameter

Source Type	ESI	Ion Polarity	Positive	Set Nebulizer	1.2 Bar
Focus	Active	Set Capillary	4500 V	Set Dry Heater	200 °C
Scan Begin	50 m/z	Set End Plate Offset	-500 V	Set Dry Gas	7.0 l/min
Scan End	3000 m/z	Set Collision Cell RF	130.0 Vpp	Set Divert Valve	Waste

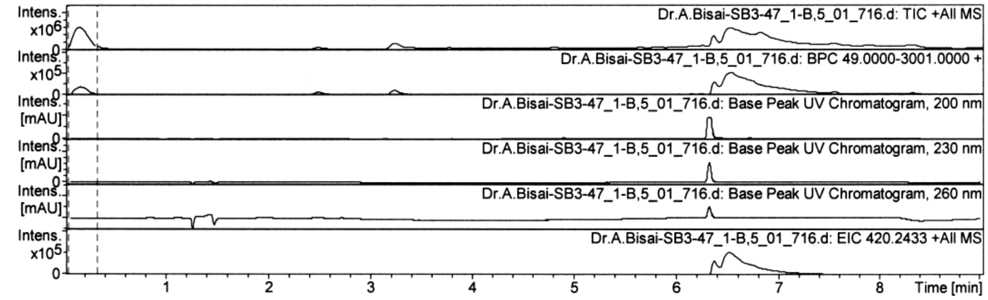

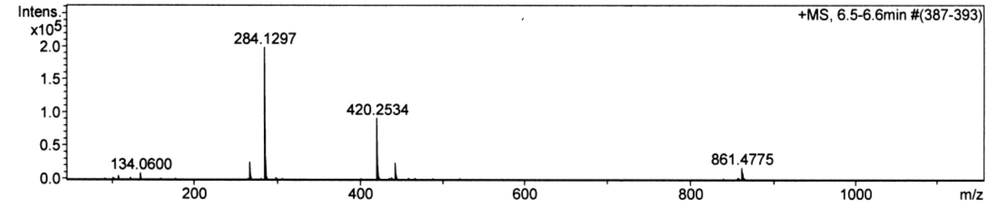

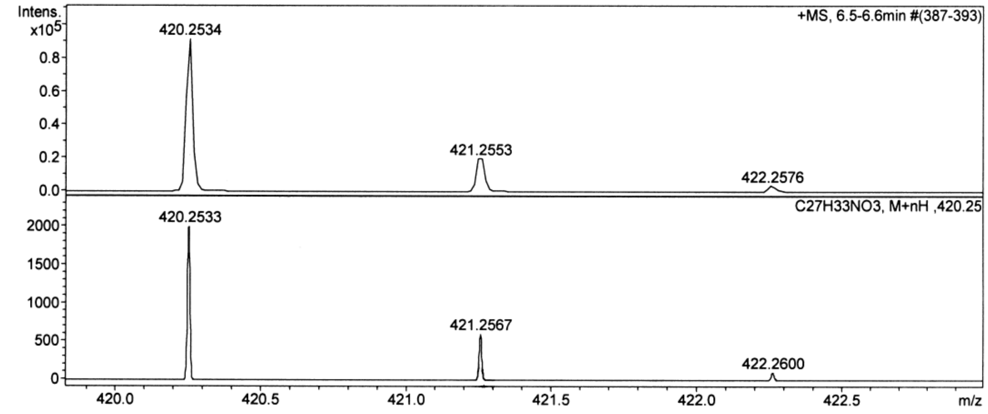

Bruker Compass DataAnalysis 4.0 printed: 3/22/2013 4:15:20 PM Page 1 of 1

Scanned copy of mass spectrum (HRMS) of (**10u**)

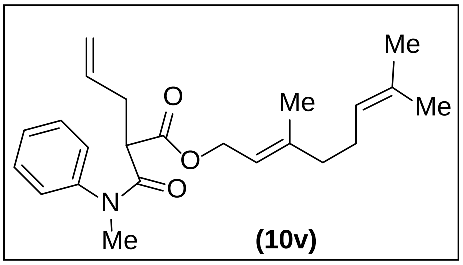

(10v)

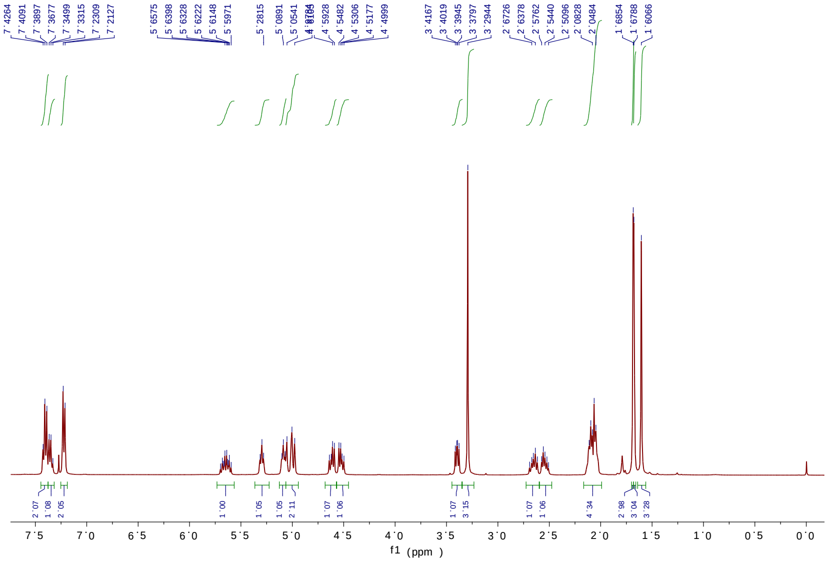

^{1}H NMR (400 MHz, $CDCl_3$) of compound (**10v**)

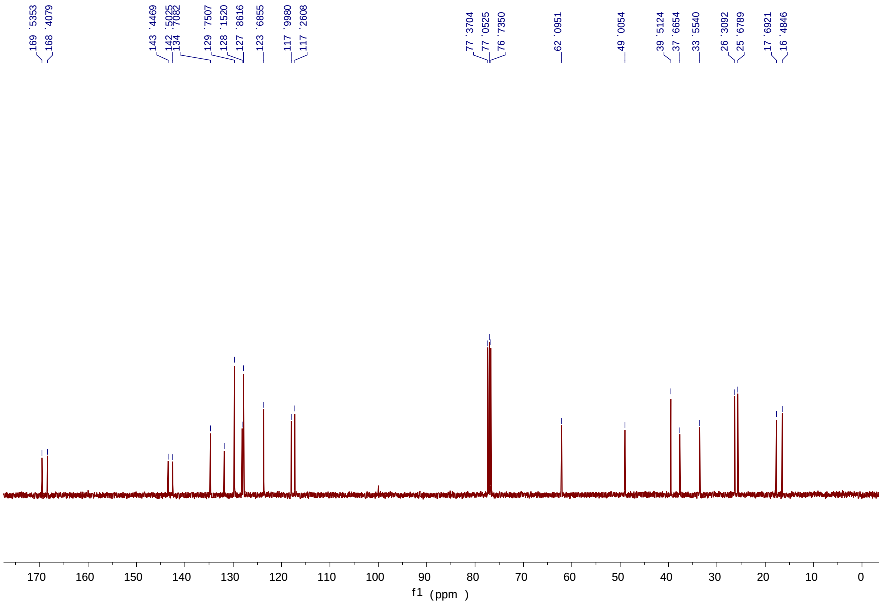

^{13}C NMR (100 MHz, $CDCl_3$) of compound (**10v**)

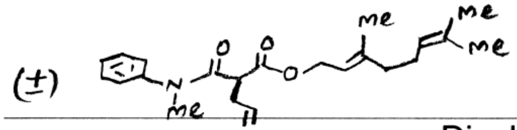

Display Report

Analysis Info

Analysis Name	D:\Data\user data\2013\MARCH\22 MAR\Dr.A.Bisai-SB3-48_1-B,6_01_717.d	Acquisition Date	3/22/2013 12:28:03 PM
Method	HRLCMS-20 Sept.m	Operator	Amit
Sample Name	Dr.A.Bisai-SB3-48	Instrument	micrOTOF-Q II 10330
Comment			

Acquisition Parameter

Source Type	ESI	Ion Polarity	Positive	Set Nebulizer	1.2 Bar
Focus	Active	Set Capillary	4500 V	Set Dry Heater	200 °C
Scan Begin	50 m/z	Set End Plate Offset	-500 V	Set Dry Gas	7.0 l/min
Scan End	3000 m/z	Set Collision Cell RF	130.0 Vpp	Set Divert Valve	Waste

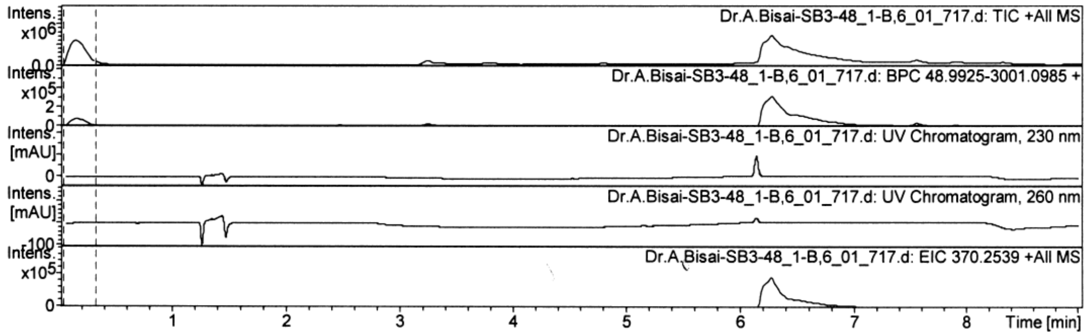

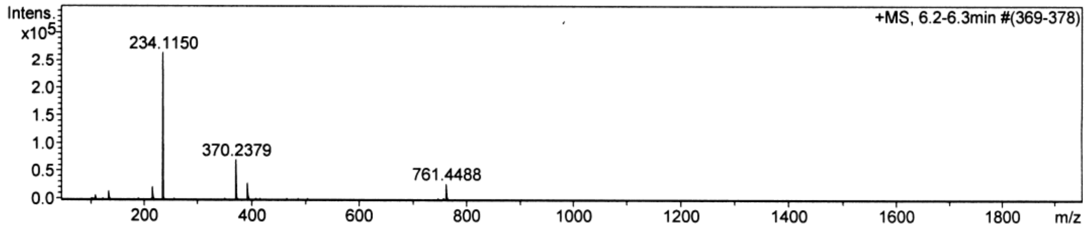

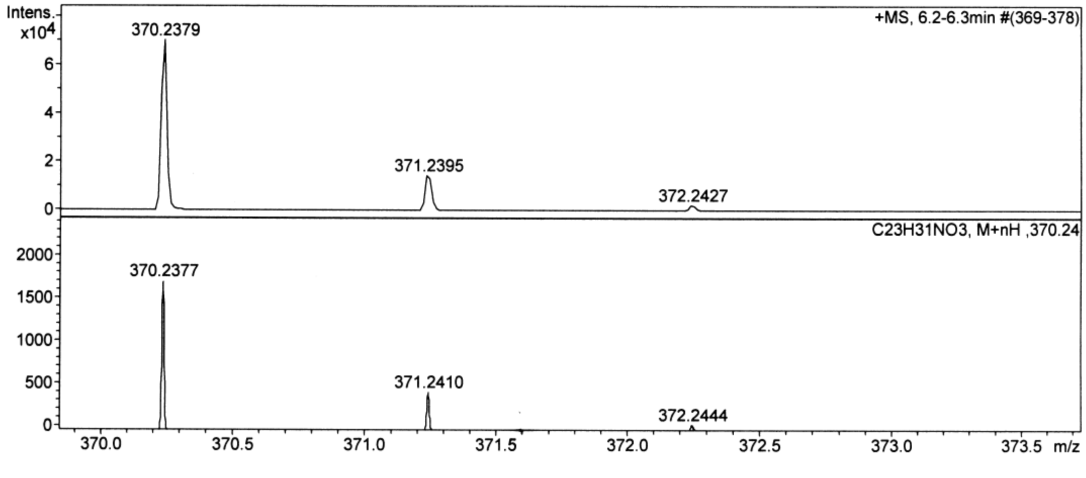

Scanned copy of mass spectrum (HRMS) of (**10v**)

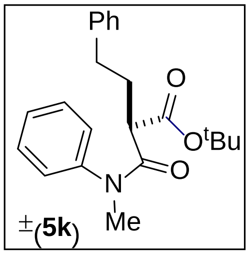

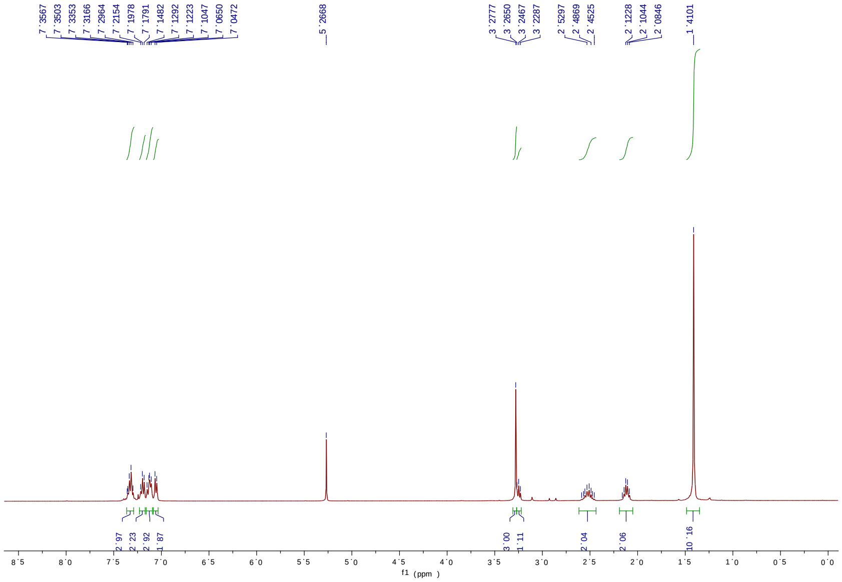

^{1}H NMR (400 MHz, $CDCl_3$) of compound (**5k**)

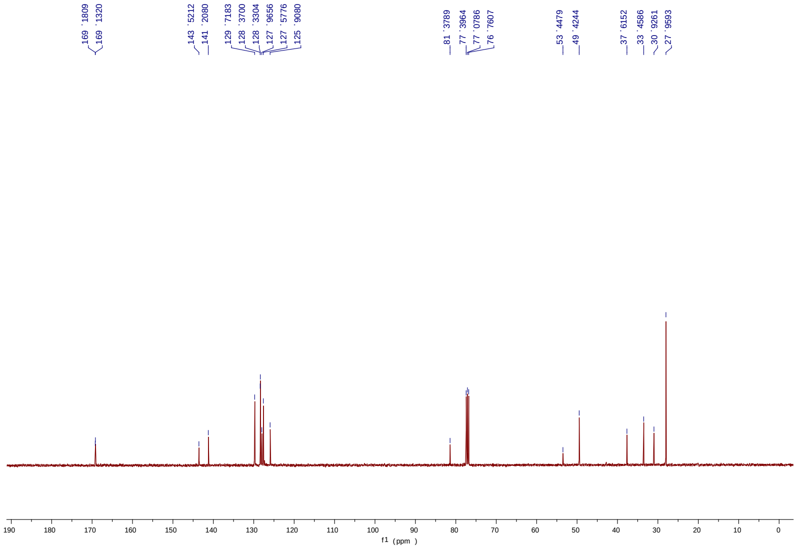

^{13}C NMR (100 MHz, $CDCl_3$) of compound (**5k**)

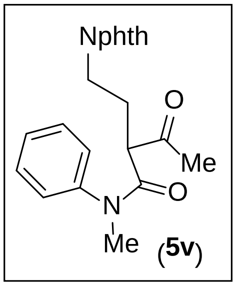

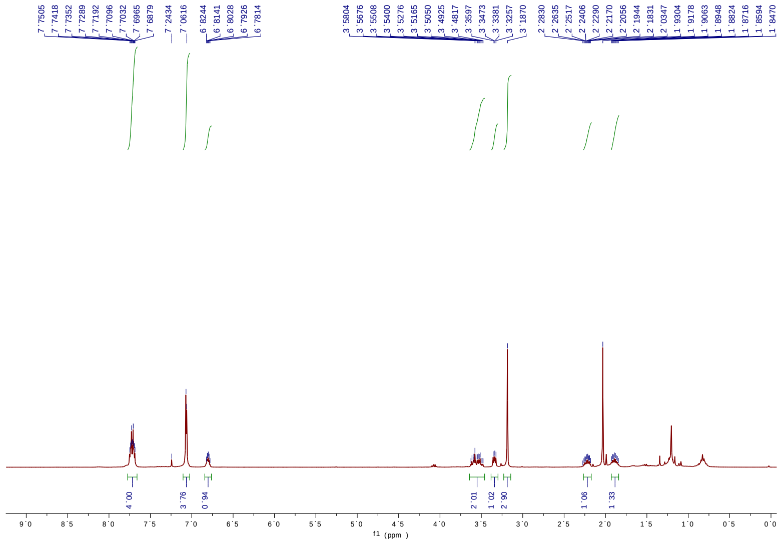

^{1}H NMR (400 MHz, $CDCl_3$) of compound (**5v**)

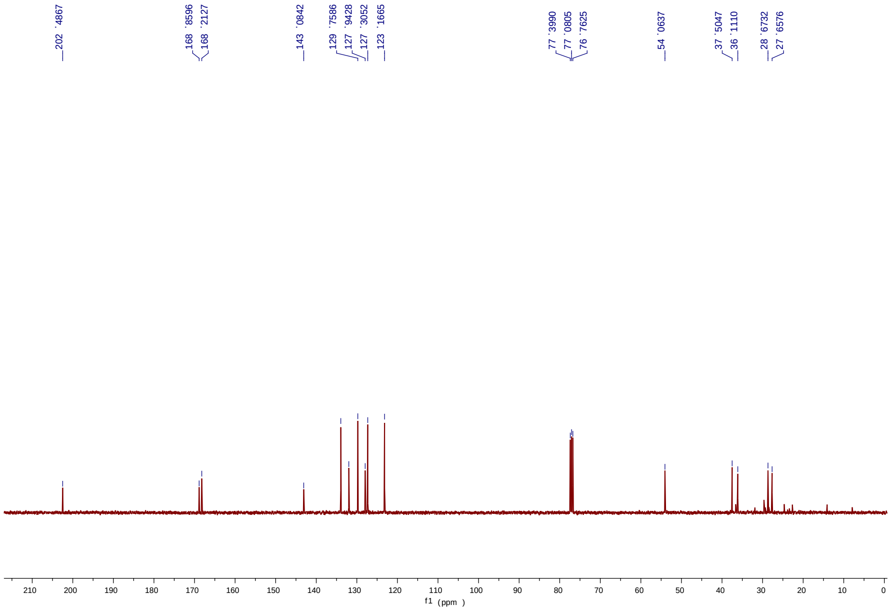

^{13}C NMR (100 MHz, $CDCl_3$) of compound (**5v**)

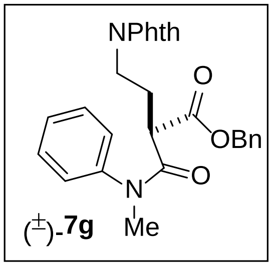

(±)-**7g**

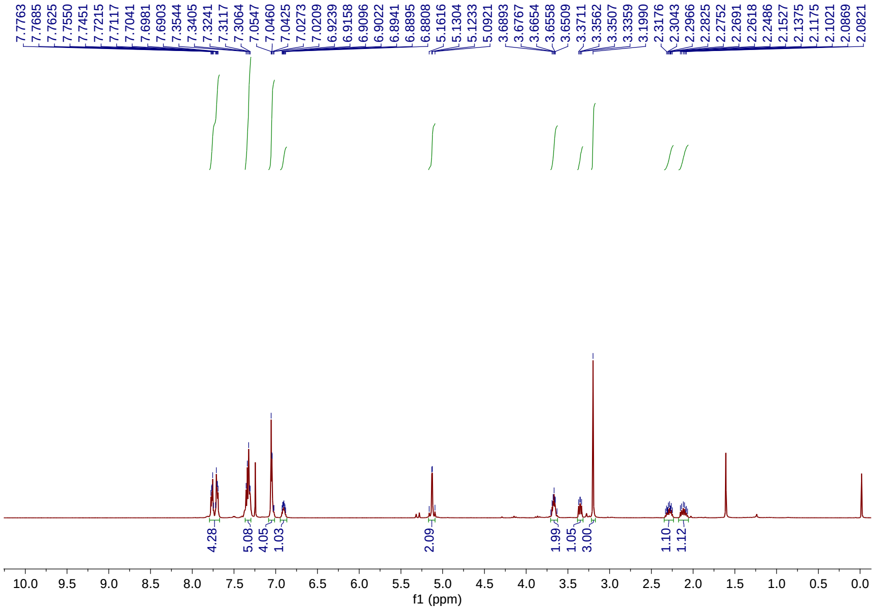

^{1}H NMR (400 MHz, CDCl$_3$) of compound (**7g**)

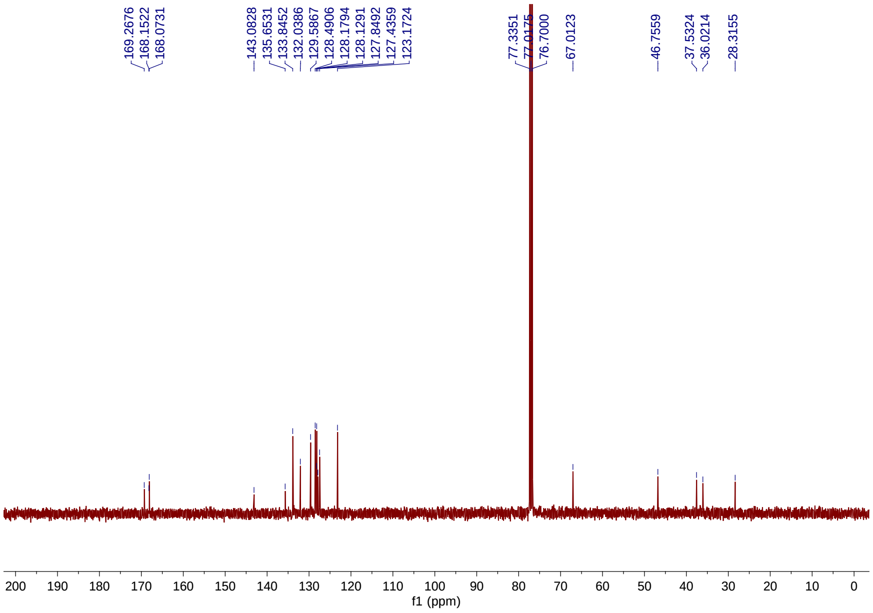

^{13}C NMR (100 MHz, CDCl$_3$) of compound (**7g**)

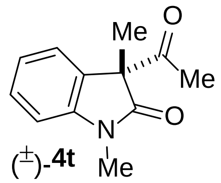

(±)-**4t**

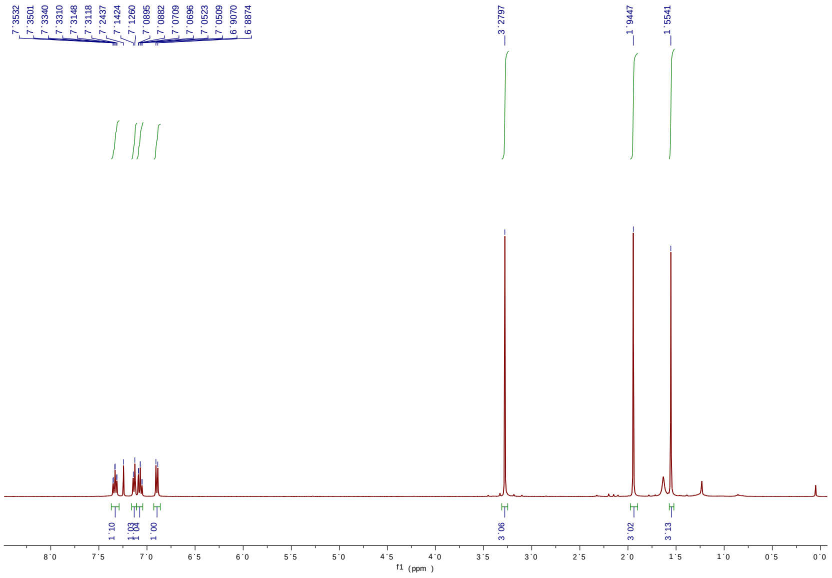

^{1}H NMR (400 MHz, $CDCl_3$) of compound (±)-**4t**

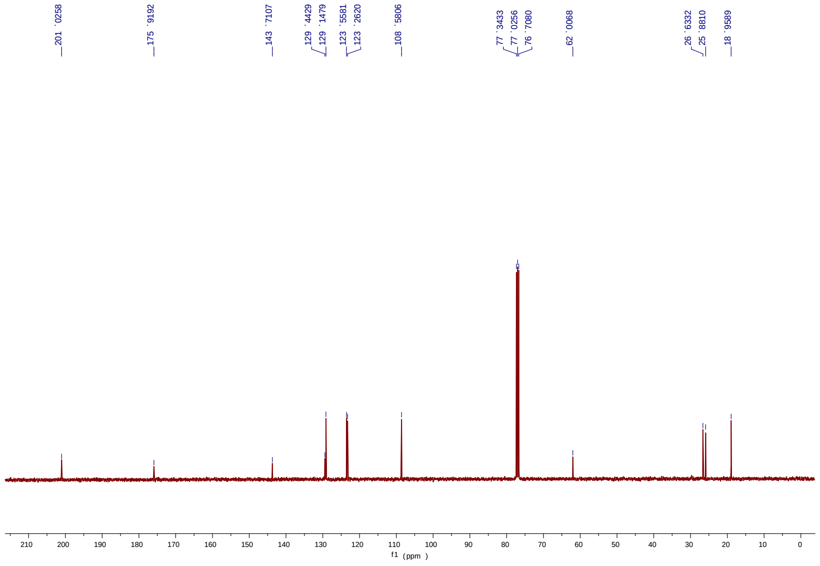

^{13}C NMR (100 MHz, $CDCl_3$) of compound (±)-**4t**

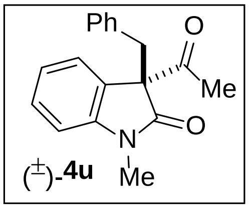

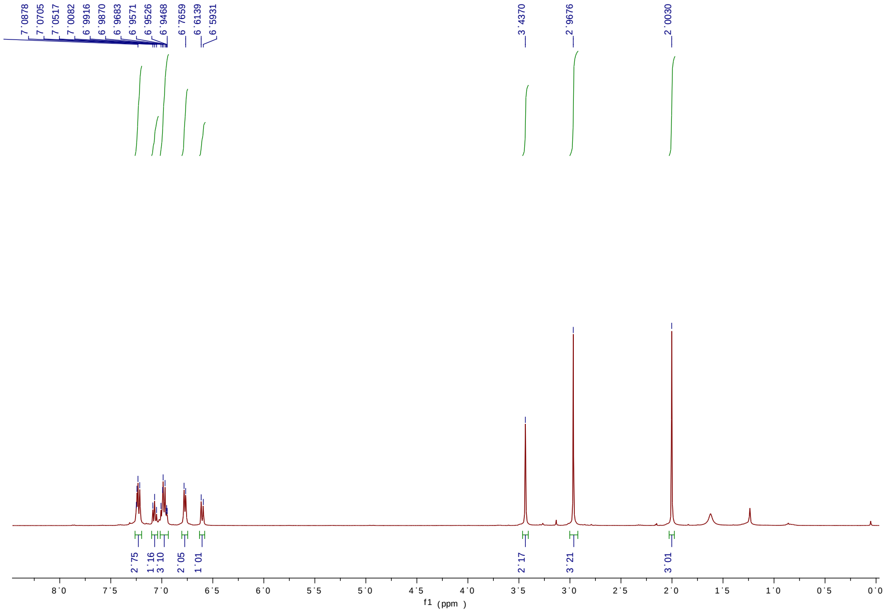

^{1}H NMR (400 MHz, $CDCl_3$) of compound (±)-**4u**

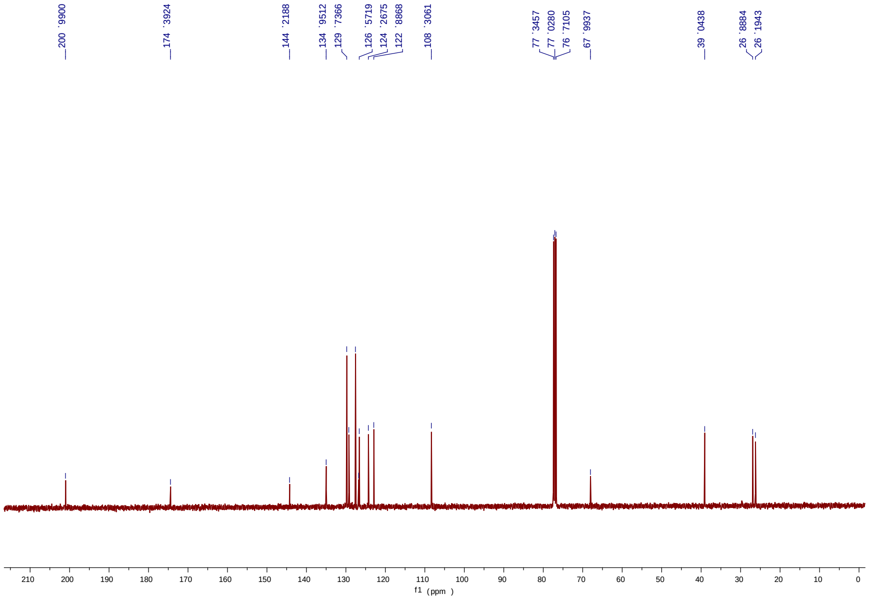

^{13}C NMR (100 MHz, $CDCl_3$) of compound (±)-**4u**

Display Report

Analysis Info

Analysis Name	D:\Data\user data\2013\AUG\22 AUG\Dr.A.Bisai-SG6-213_1-A,5_01_1113.d	Acquisition Date	8/22/2013 1:18:31 PM
Method	HRLCMS-20 Sept.m	Operator	Amit
Sample Name	Dr.A.Bisai-SG6-213	Instrument	micrOTOF-Q II 10330
Comment			

Acquisition Parameter

Source Type	ESI	Ion Polarity	Positive	Set Nebulizer	1.2 Bar
Focus	Active	Set Capillary	4500 V	Set Dry Heater	200 °C
Scan Begin	50 m/z	Set End Plate Offset	-500 V	Set Dry Gas	7.0 l/min
Scan End	3000 m/z	Set Collision Cell RF	130.0 Vpp	Set Divert Valve	Waste

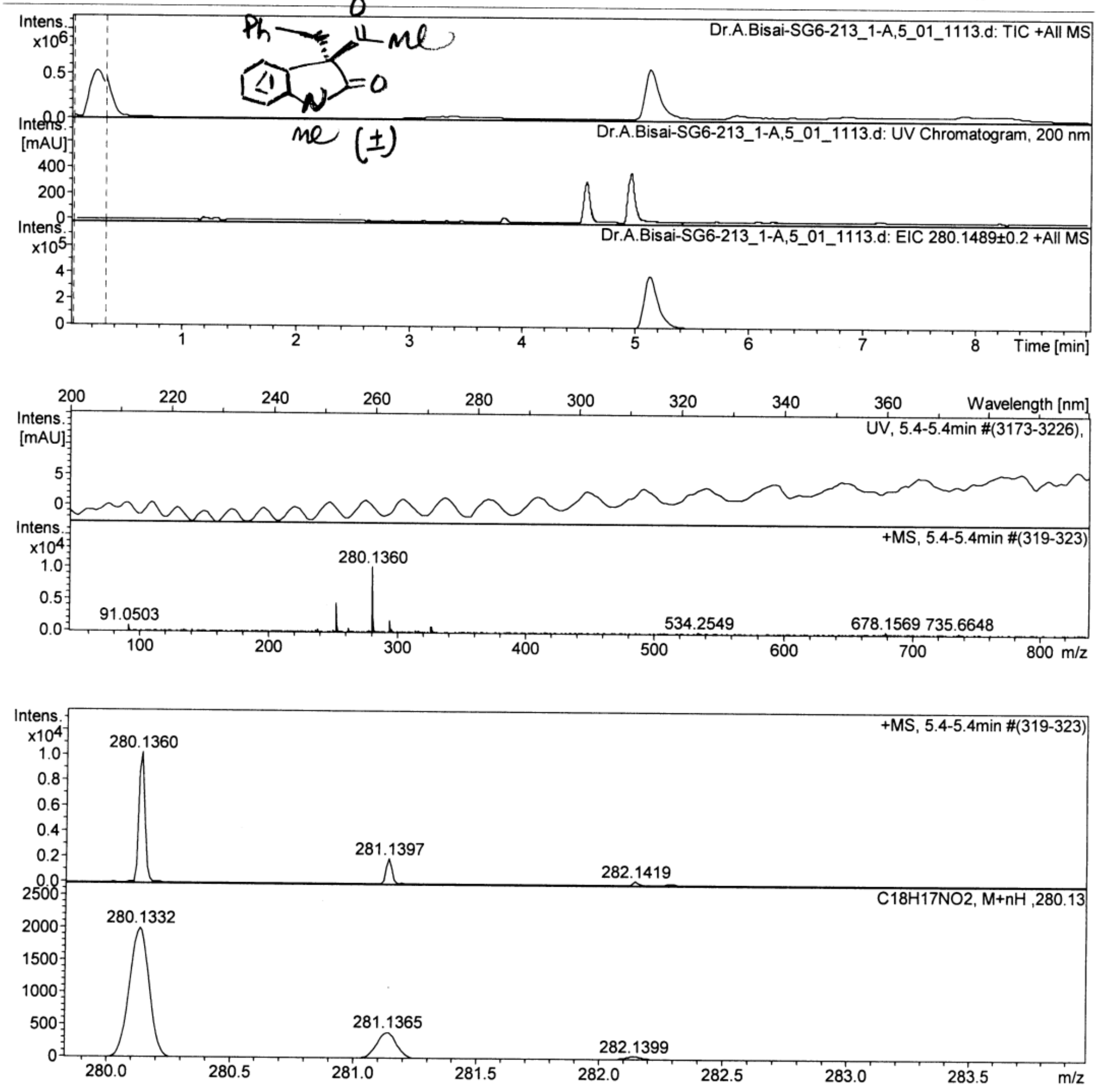

Bruker Compass DataAnalysis 4.0 printed: 8/22/2013 3:24:21 PM Page 1 of 1

Scanned copy of mass spectrum (HRMS) of (±)-**4u**

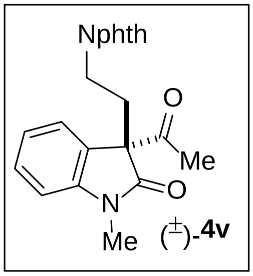

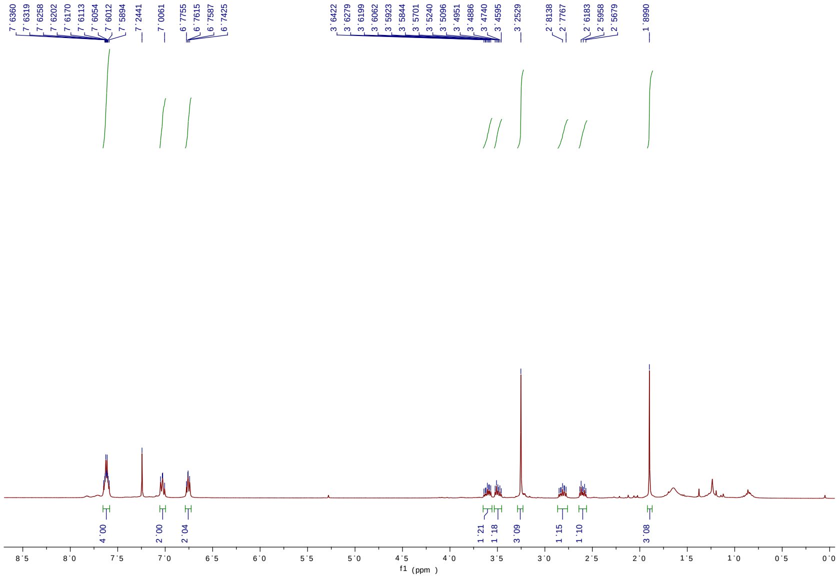

^{1}H NMR (400 MHz, $CDCl_3$) of compound (±)-**4v**

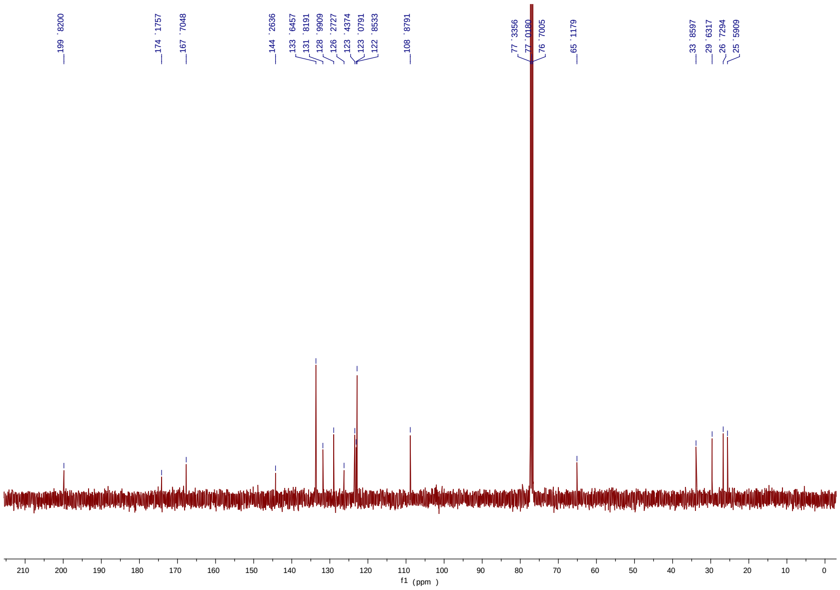

^{13}C NMR (100 MHz, $CDCl_3$) of compound (±)-**4v**

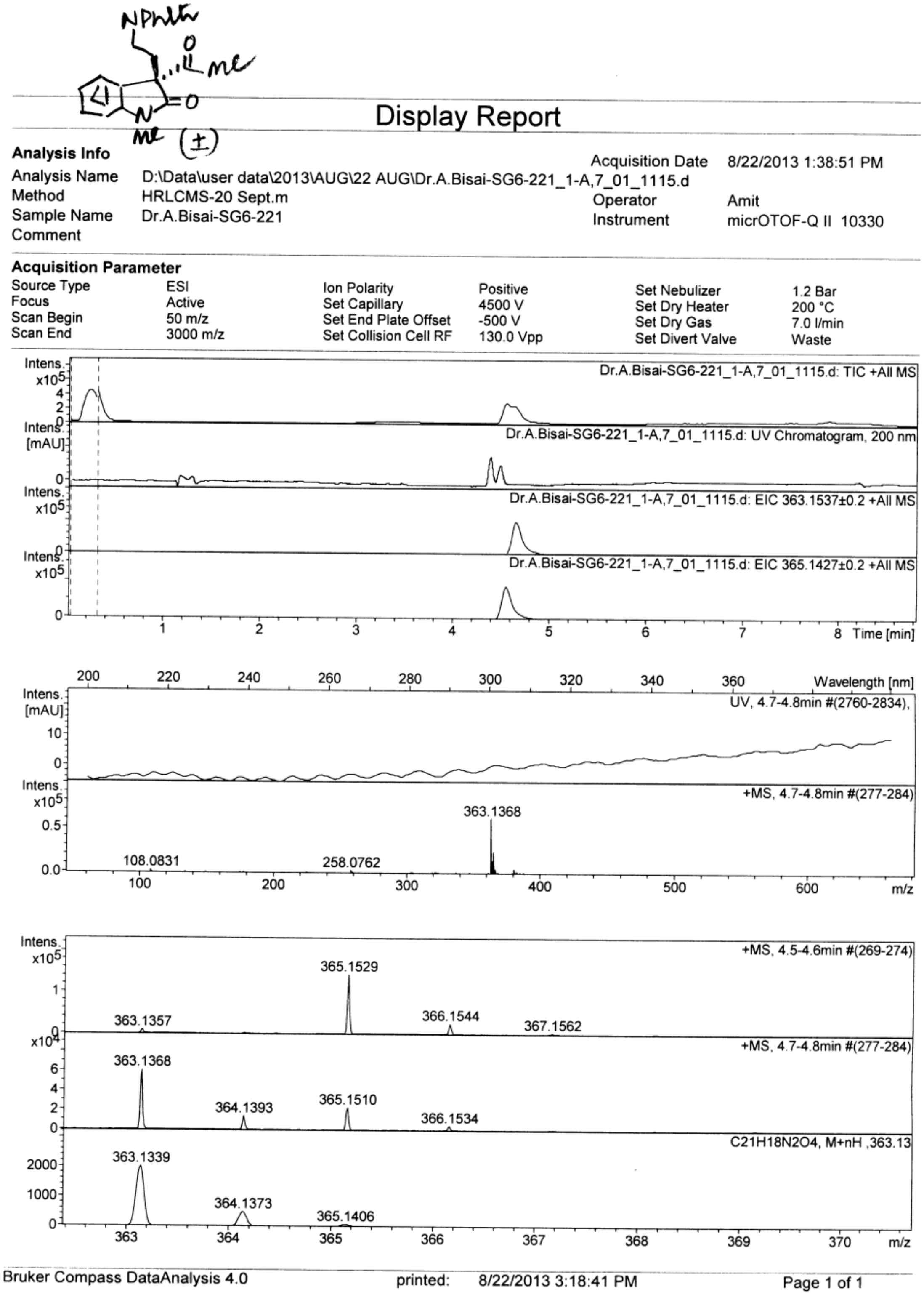

Scanned copy of mass spectrum (HRMS) of (±)-**4v**

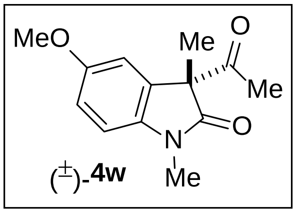

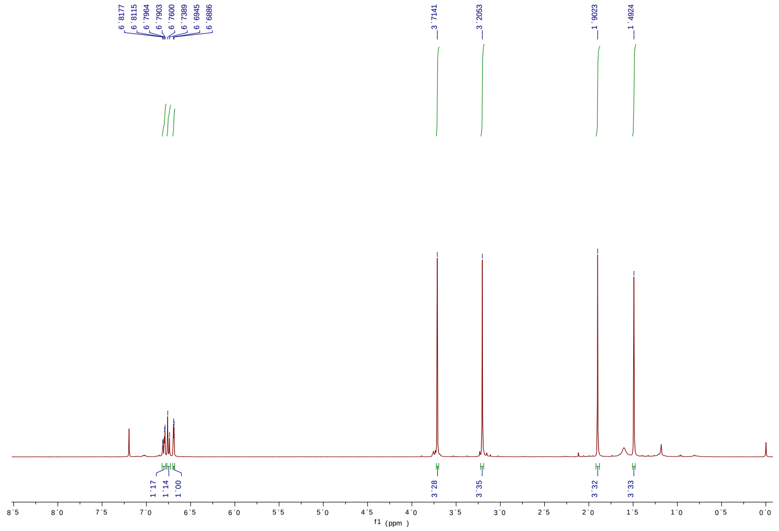

^{1}H NMR (400 MHz, $CDCl_3$) of compound (±)-**4w**

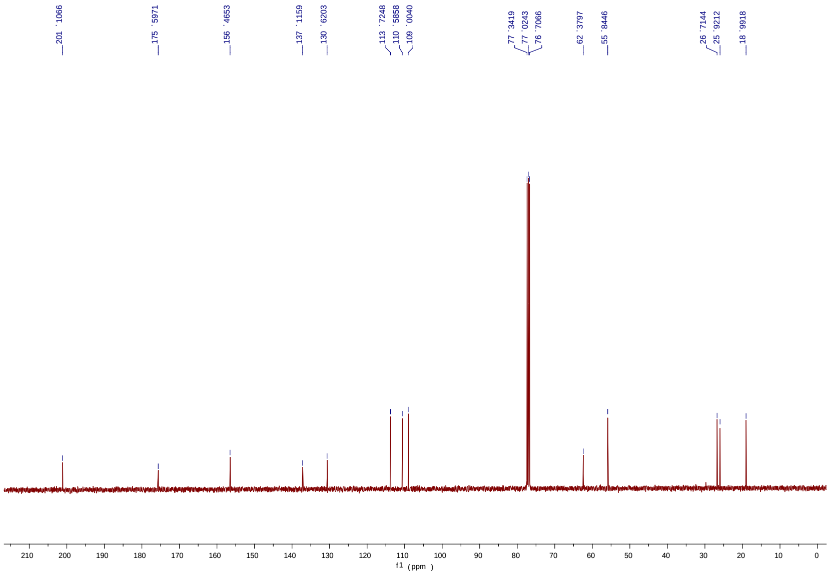

^{13}C NMR (100 MHz, $CDCl_3$) of compound (±)-**4w**

Display Report

Analysis Info

Analysis Name	D:\Data\user data\2013\AUG\23 AUG\Dr.A.Bisai-SG6-226_1-A,5_01_1120.d	Acquisition Date	8/23/2013 2:06:03 PM
Method	HRLCMS-20 Sept.m	Operator	Amit
Sample Name	Dr.A.Bisai-SG6-226	Instrument	micrOTOF-Q II 10330
Comment			

Acquisition Parameter

Source Type	ESI	Ion Polarity	Positive	Set Nebulizer	1.2 Bar
Focus	Active	Set Capillary	4500 V	Set Dry Heater	200 °C
Scan Begin	50 m/z	Set End Plate Offset	-500 V	Set Dry Gas	7.0 l/min
Scan End	3000 m/z	Set Collision Cell RF	130.0 Vpp	Set Divert Valve	Waste

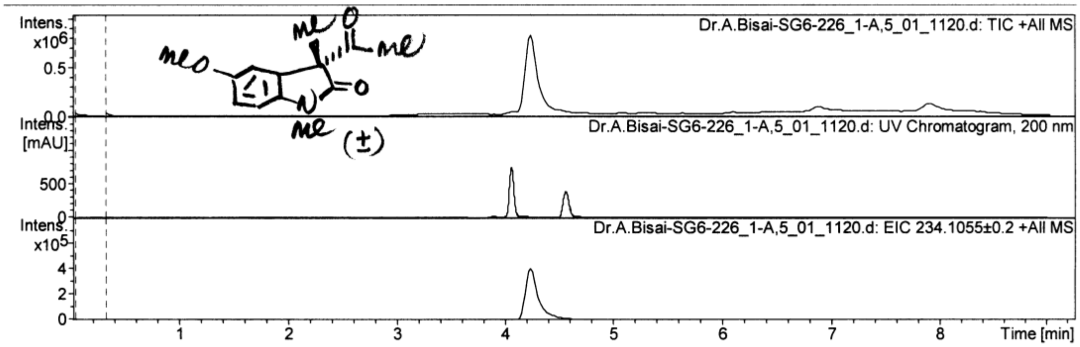

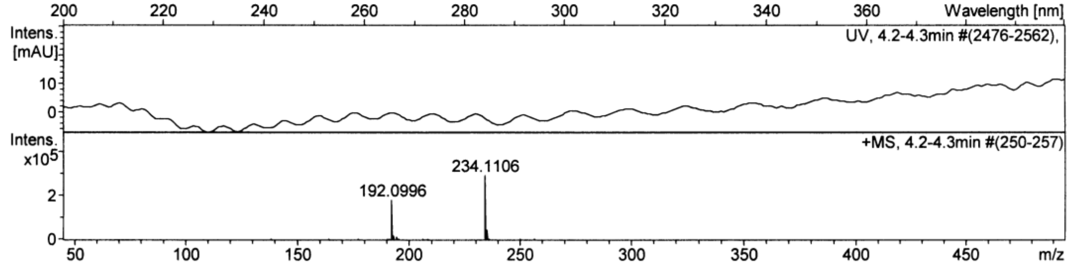

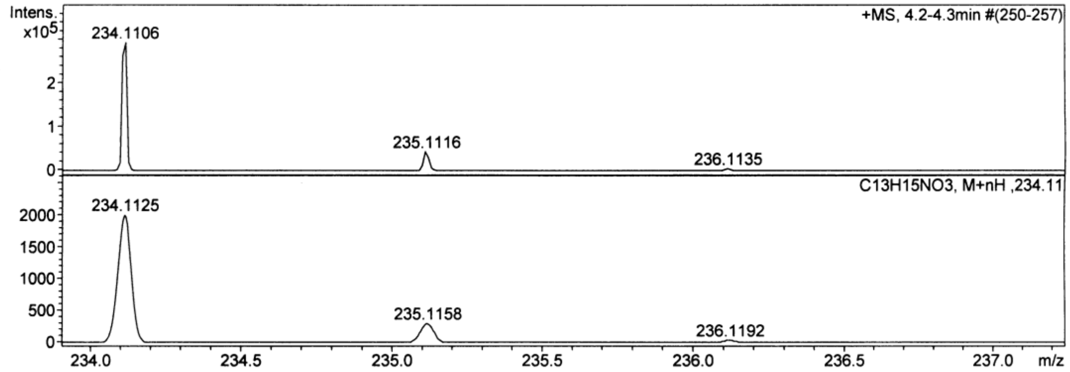

Bruker Compass DataAnalysis 4.0 printed: 8/23/2013 3:45:29 PM Page 1 of 1

Scanned copy of mass spectrum (HRMS) of (±)-**4w**

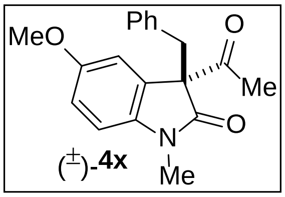

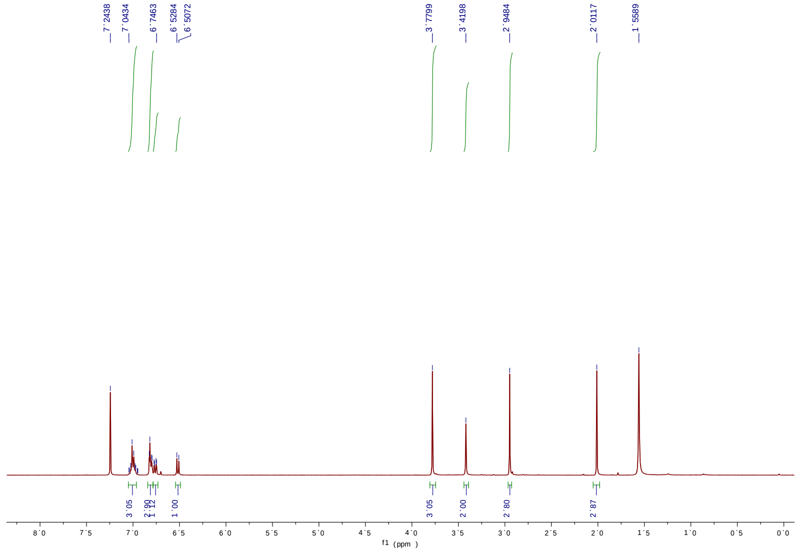

^{1}H NMR (400 MHz, $CDCl_3$) of compound (±)-**4x**

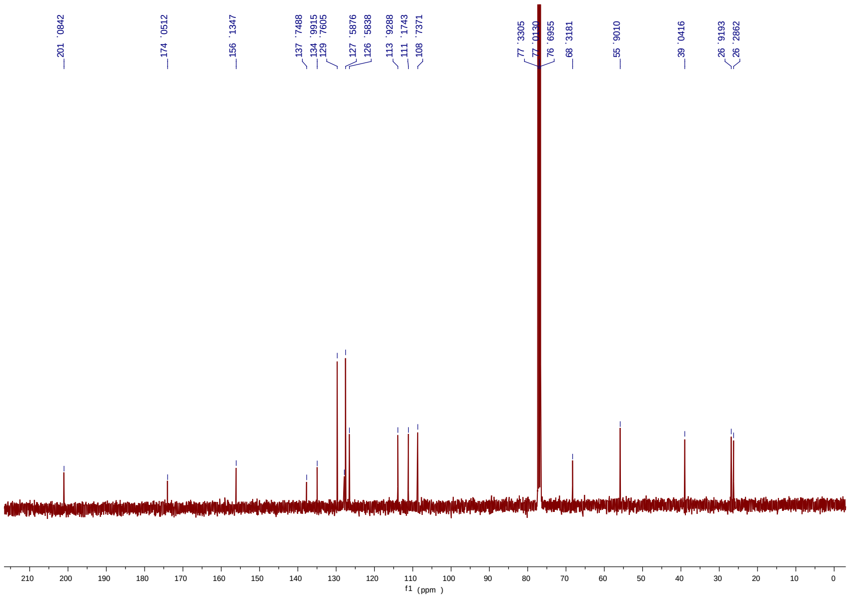

^{13}C NMR (100 MHz, $CDCl_3$) of compound (±)-**4x**

Display Report

Analysis Info

Analysis Name	D:\Data\user data\2013\AUG\23 AUG\Dr.A.Bisai-SG6-217_1-A,4_01_1119.d	Acquisition Date	8/23/2013 1:55:55 PM
Method	HRLCMS-20 Sept.m	Operator	Amit
Sample Name	Dr.A.Bisai-SG6-217	Instrument	micrOTOF-Q II 10330
Comment			

Acquisition Parameter

Source Type	ESI	Ion Polarity	Positive	Set Nebulizer	1.2 Bar
Focus	Active	Set Capillary	4500 V	Set Dry Heater	200 °C
Scan Begin	50 m/z	Set End Plate Offset	-500 V	Set Dry Gas	7.0 l/min
Scan End	3000 m/z	Set Collision Cell RF	130.0 Vpp	Set Divert Valve	Waste

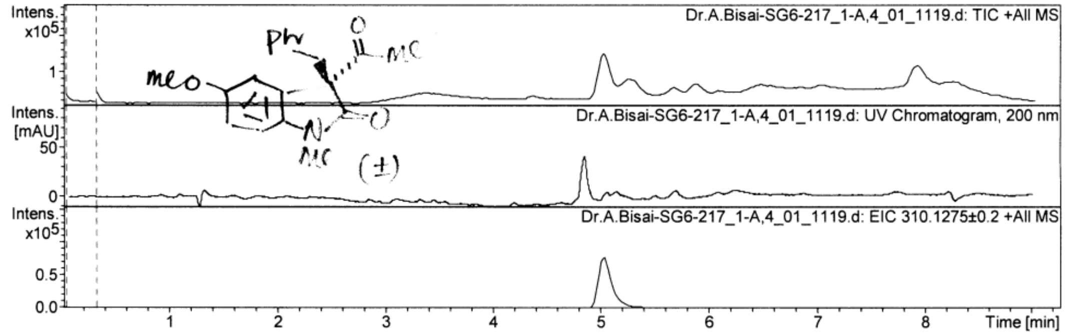

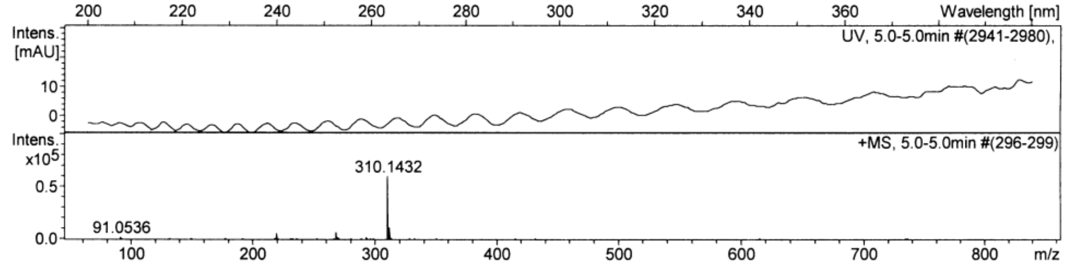

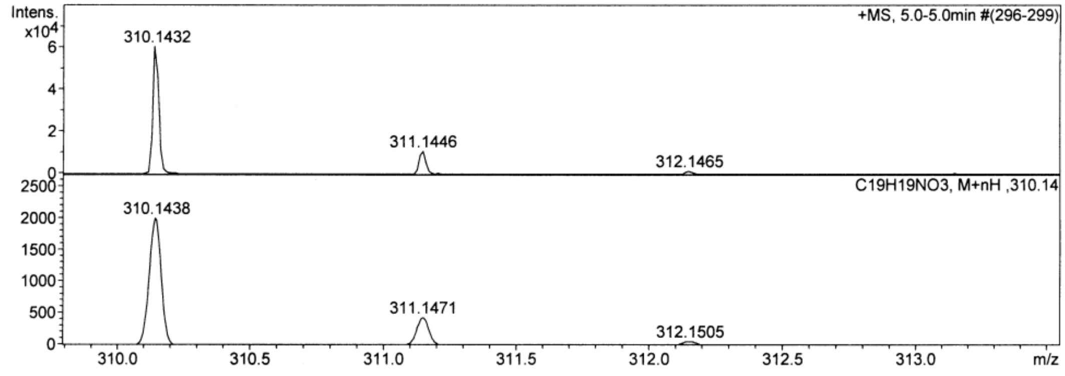

Bruker Compass DataAnalysis 4.0 printed: 8/23/2013 3:43:30 PM Page 1 of 1

Scanned copy of mass spectrum (HRMS) of (±)-**4x**

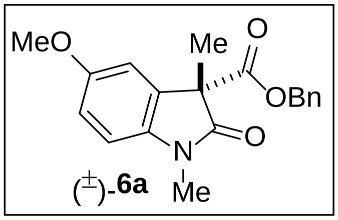

(±)-**6a**

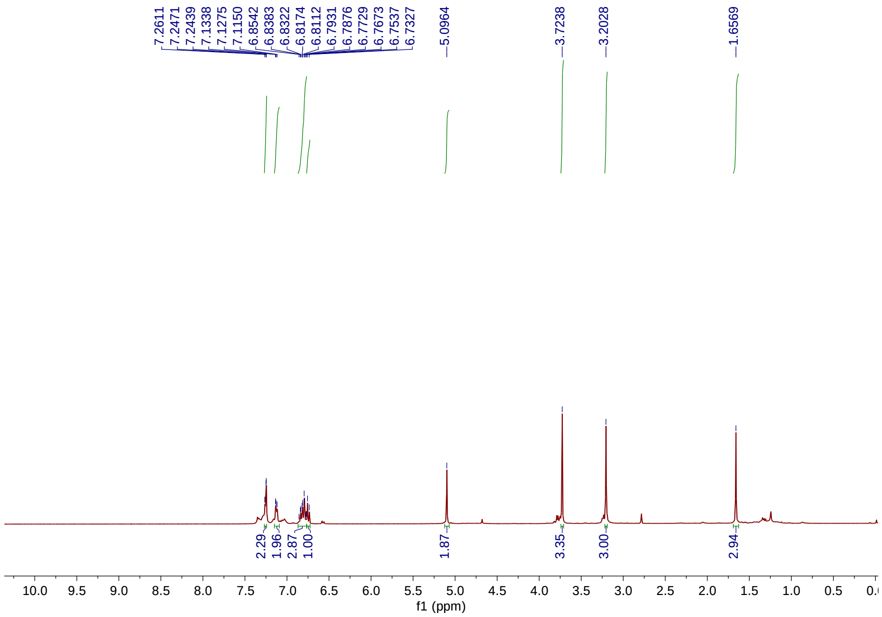

^{1}H NMR (400 MHz, $CDCl_3$) of compound (**6a**)

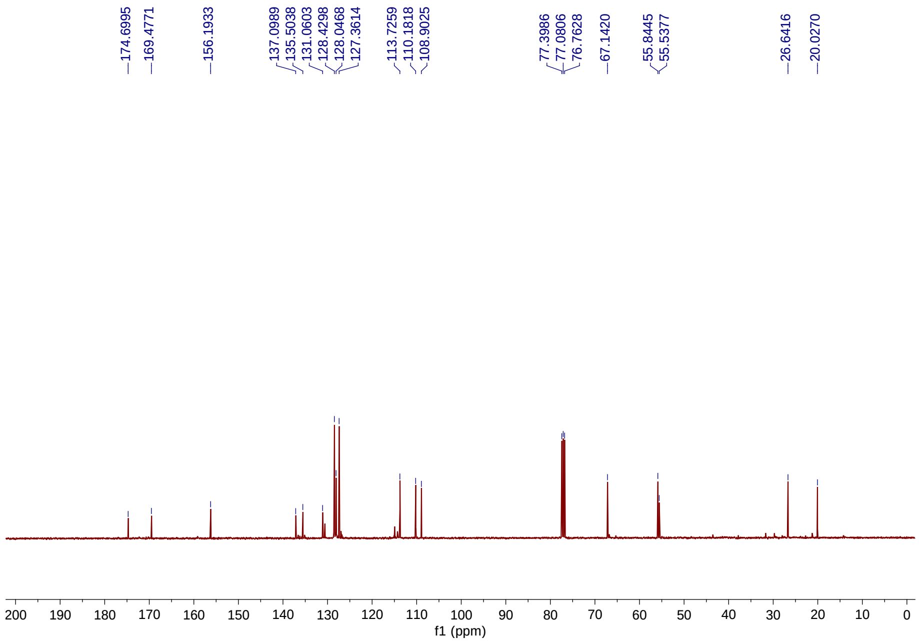

^{13}C NMR (100 MHz, $CDCl_3$) of compound (**6a**)

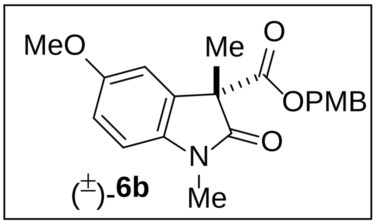

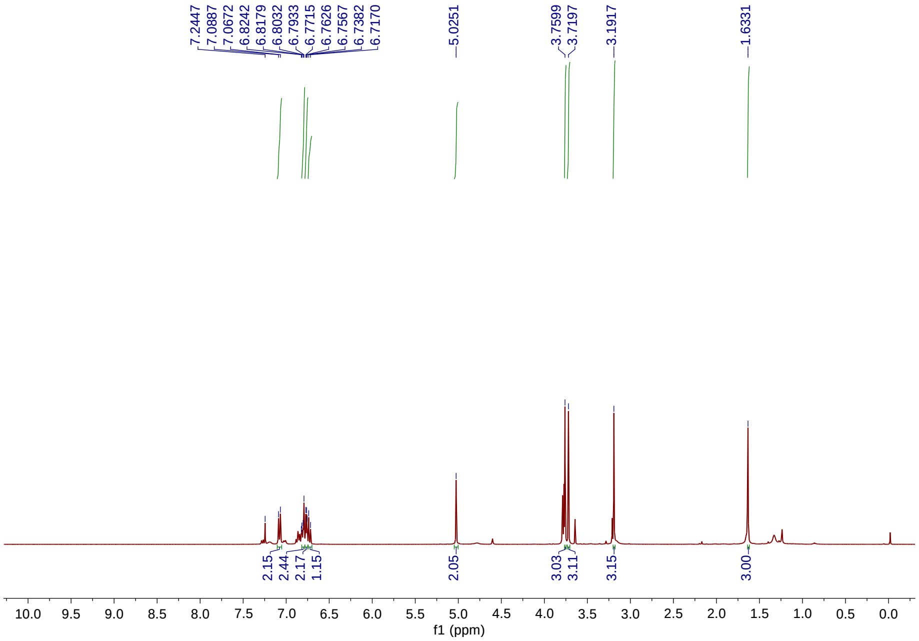

^{1}H NMR (400 MHz, $CDCl_3$) of compound (**6b**)

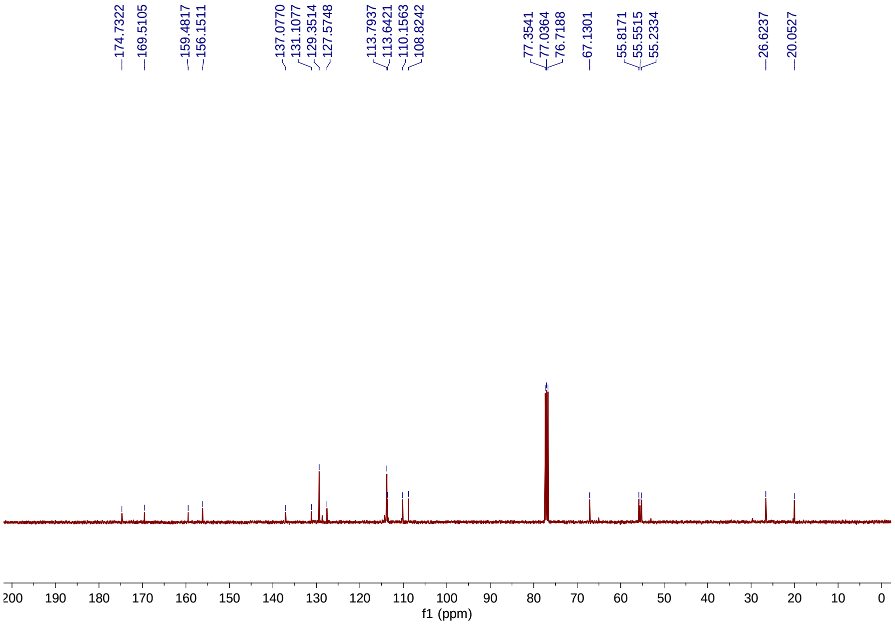

^{13}C NMR (100 MHz, $CDCl_3$) of compound (**6b**)

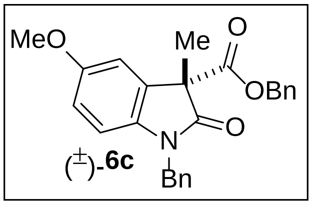

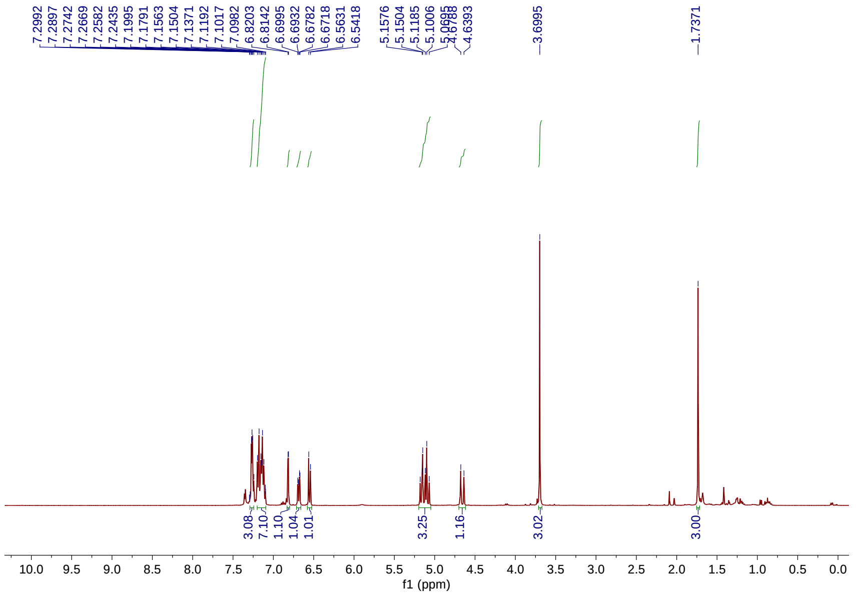

^{1}H NMR (400 MHz, $CDCl_3$) of compound (**6c**)

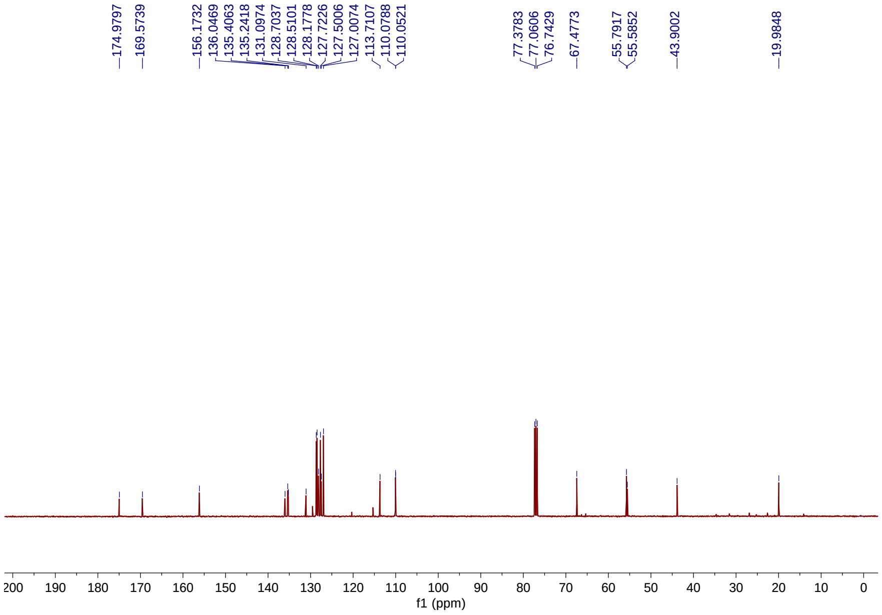

^{13}C NMR (100 MHz, $CDCl_3$) of compound (**6c**)

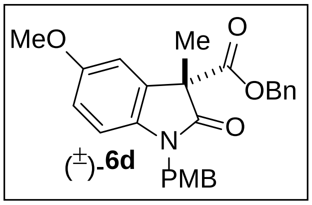

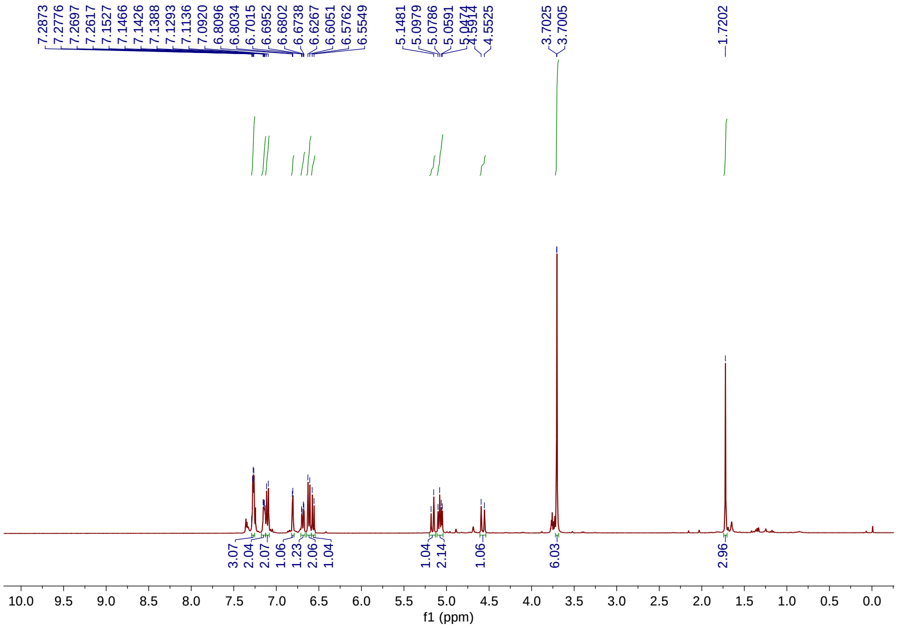

^{1}H NMR (400 MHz, $CDCl_3$) of compound (**6d**)

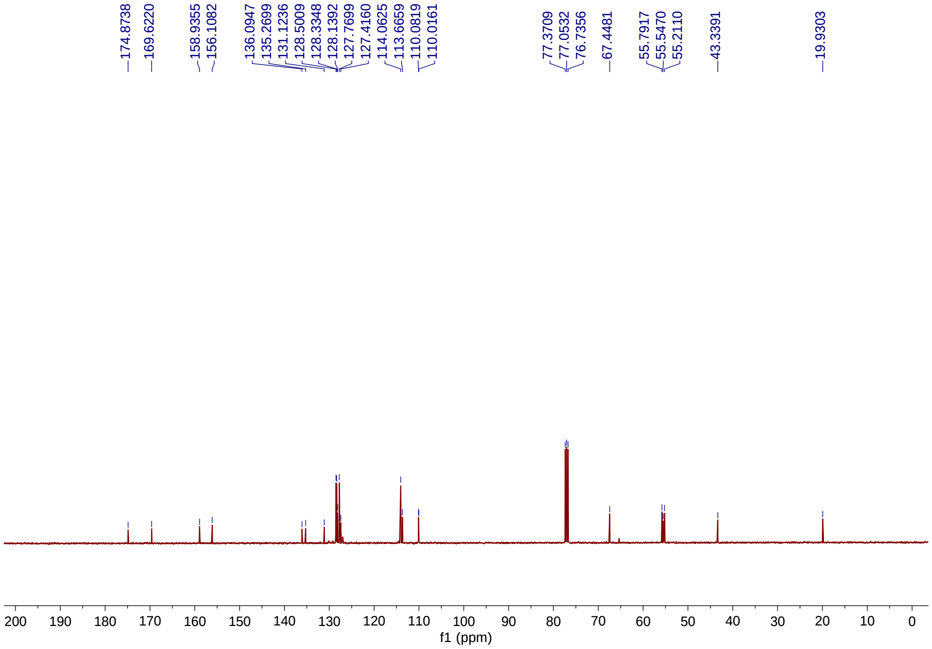

^{13}C NMR (100 MHz, $CDCl_3$) of compound (**6d**)

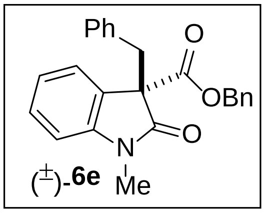

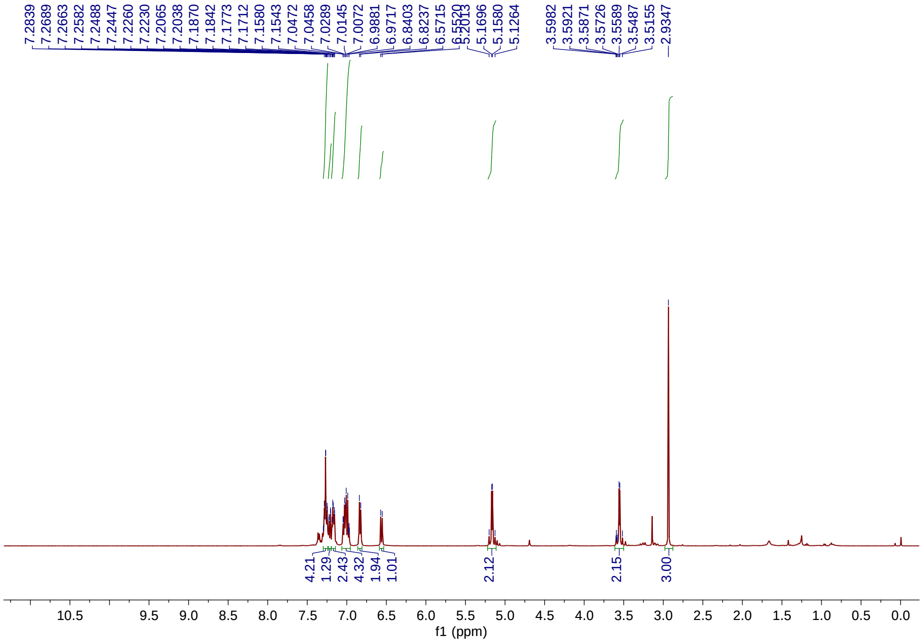

^{1}H NMR (400 MHz, $CDCl_3$) of compound (**6e**)

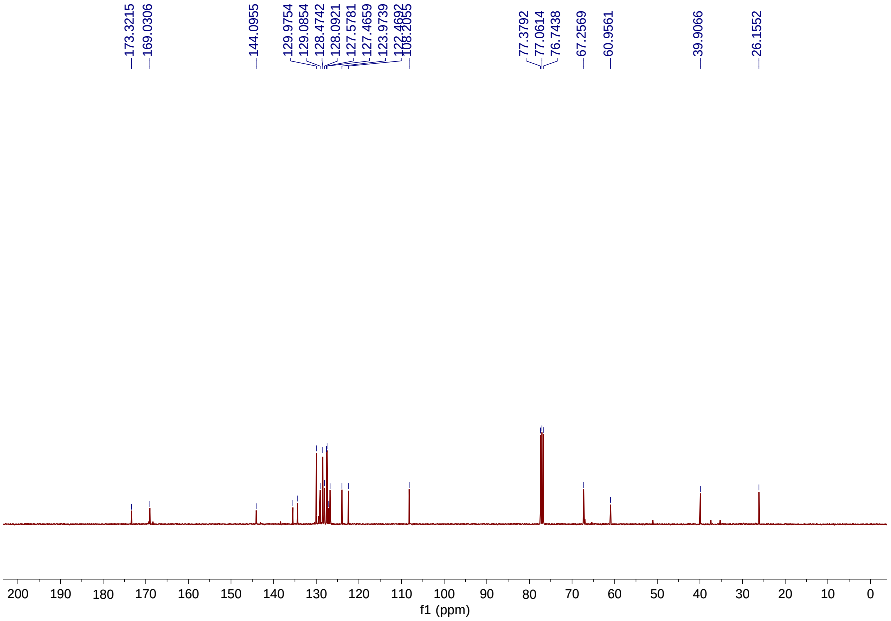

^{13}C NMR (100 MHz, $CDCl_3$) of compound (**6e**)

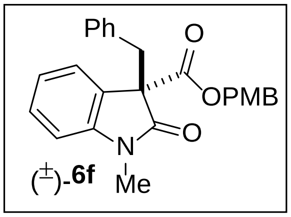

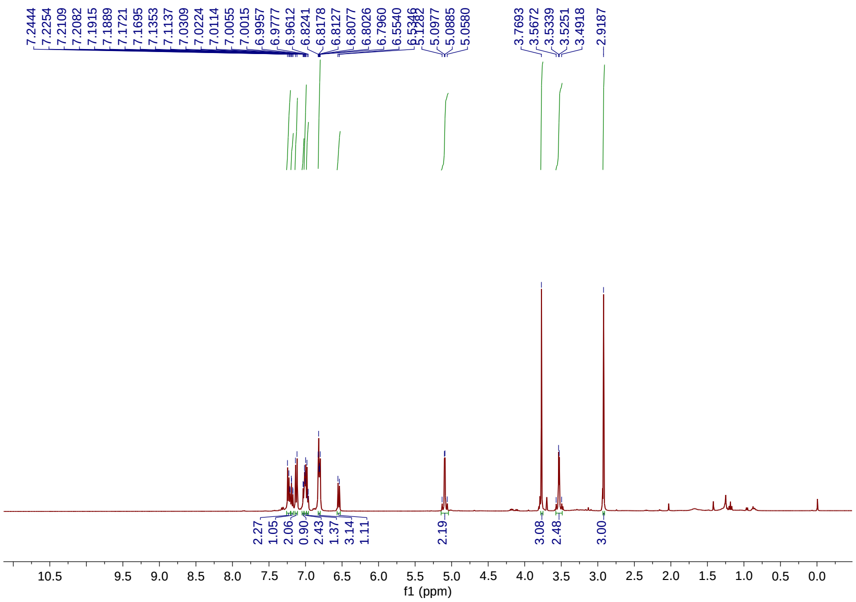

^{1}H NMR (400 MHz, $CDCl_3$) of compound (**6f**)

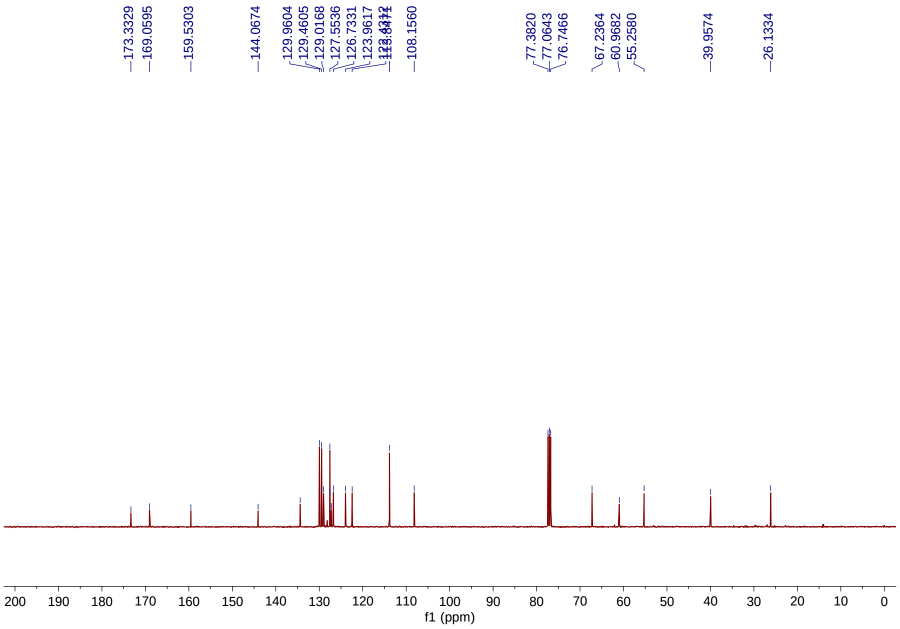

^{13}C NMR (100 MHz, $CDCl_3$) of compound (**6f**)

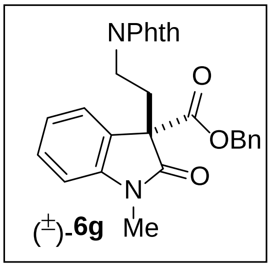

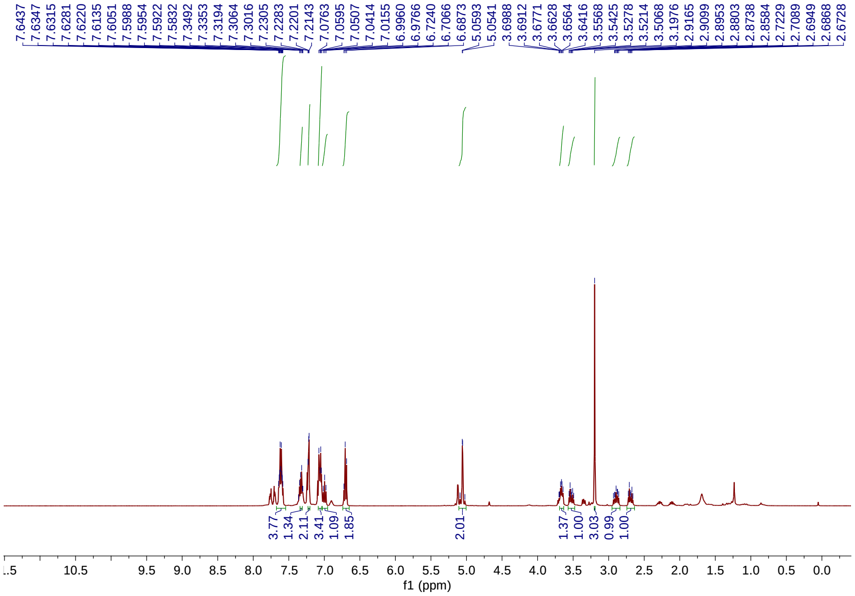

^{1}H NMR (400 MHz, $CDCl_3$) of compound (**6g**)

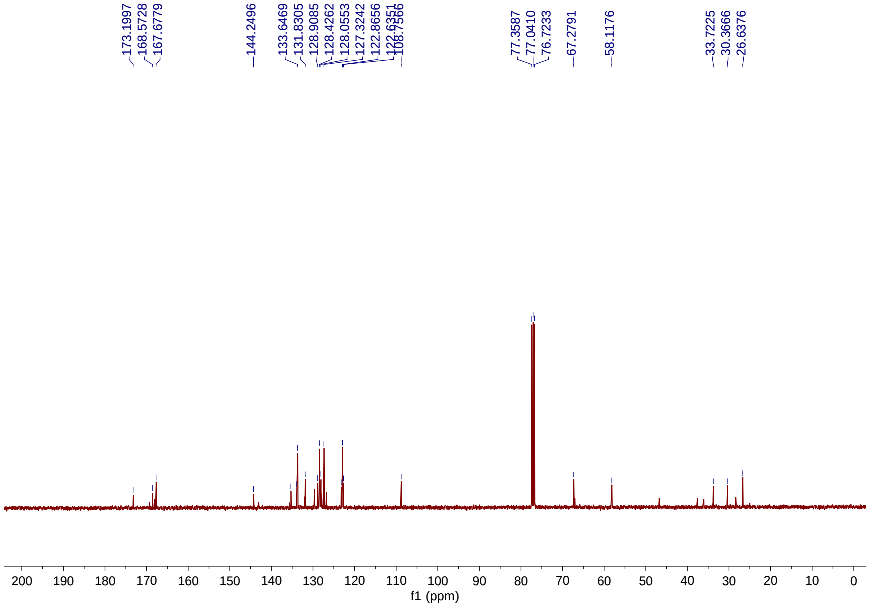

^{13}C NMR (100 MHz, $CDCl_3$) of compound (**6g**)

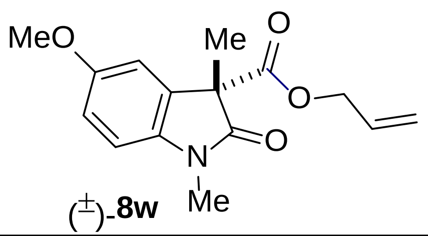

(±)-**8w**

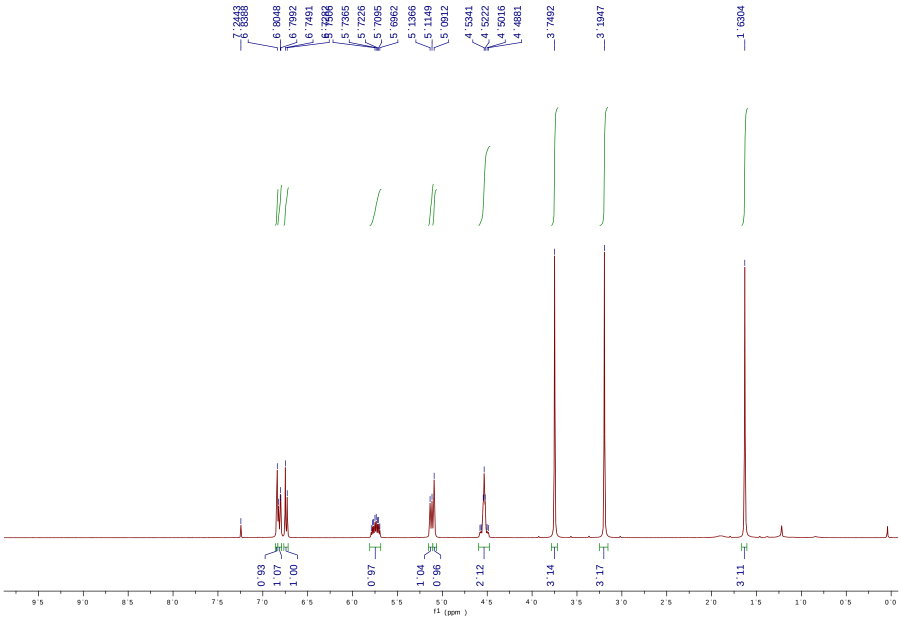

^{1}H NMR (400 MHz, $CDCl_3$) of compound (±)-**8w**

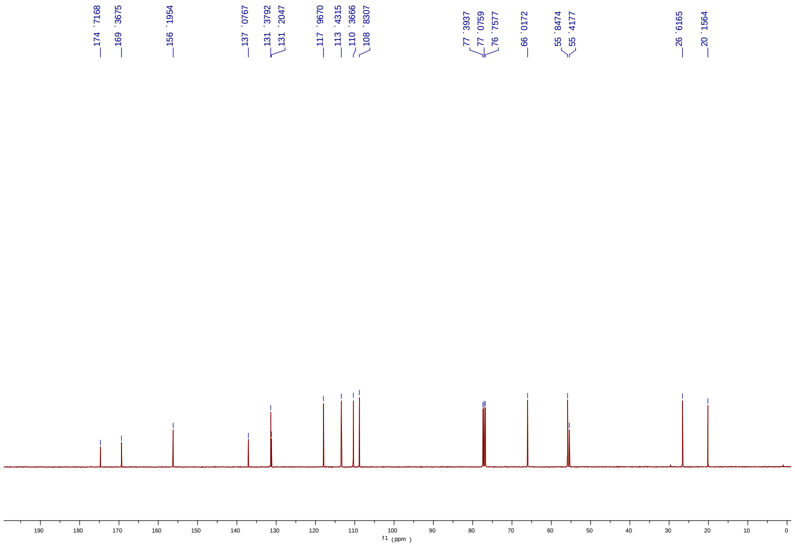

^{13}C NMR (100 MHz, $CDCl_3$) of compound (±)-**8w**

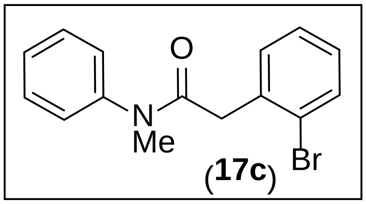

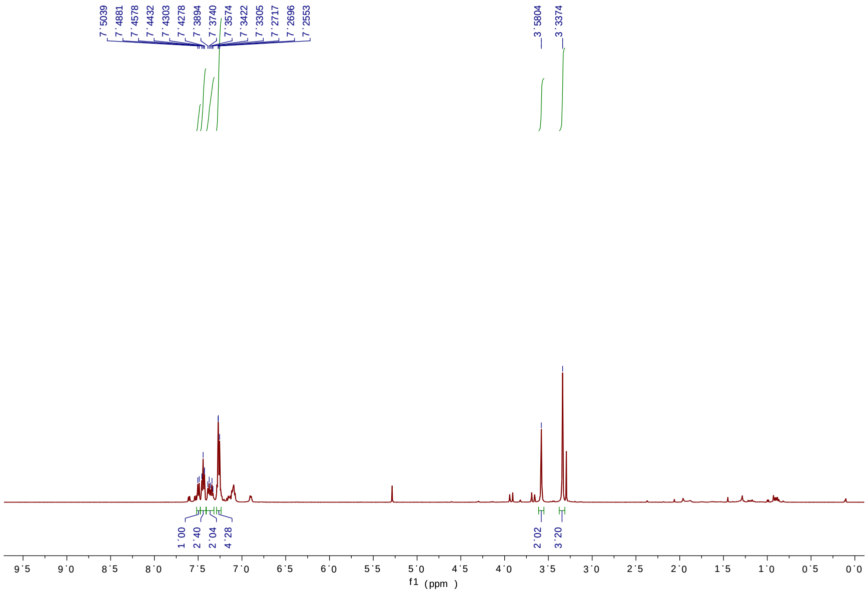

^{1}H NMR (500 MHz, $CDCl_3$) of compound (**17c**)

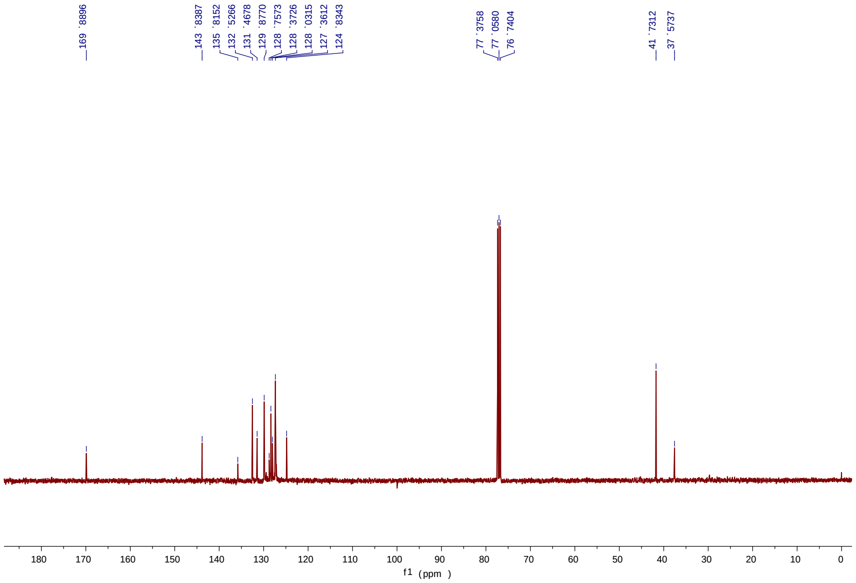

^{13}C NMR (100 MHz, $CDCl_3$) of compound (**17c**)

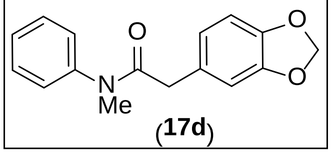

(**17d**)

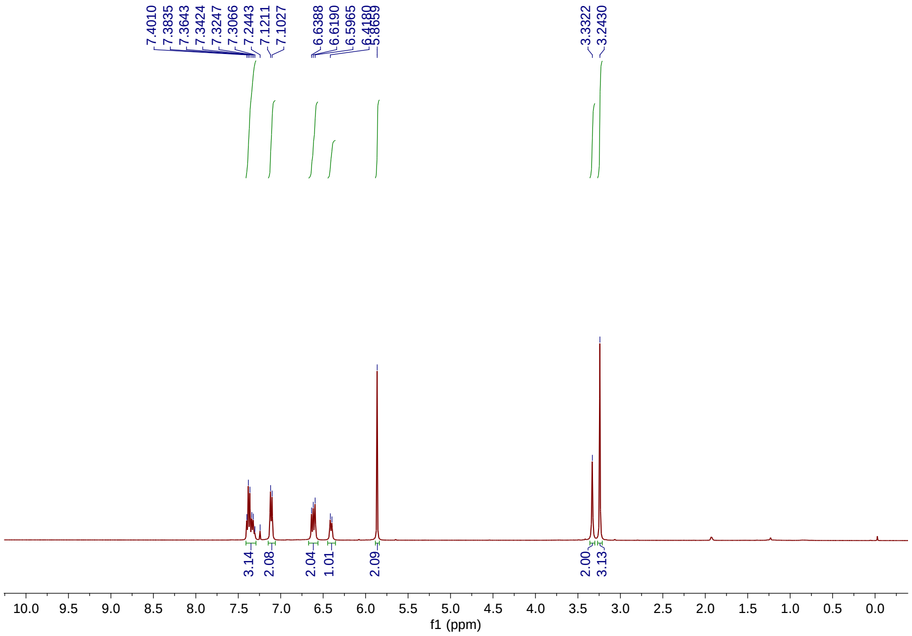

^{1}H NMR (400 MHz, $CDCl_3$) of compound (**17d**)

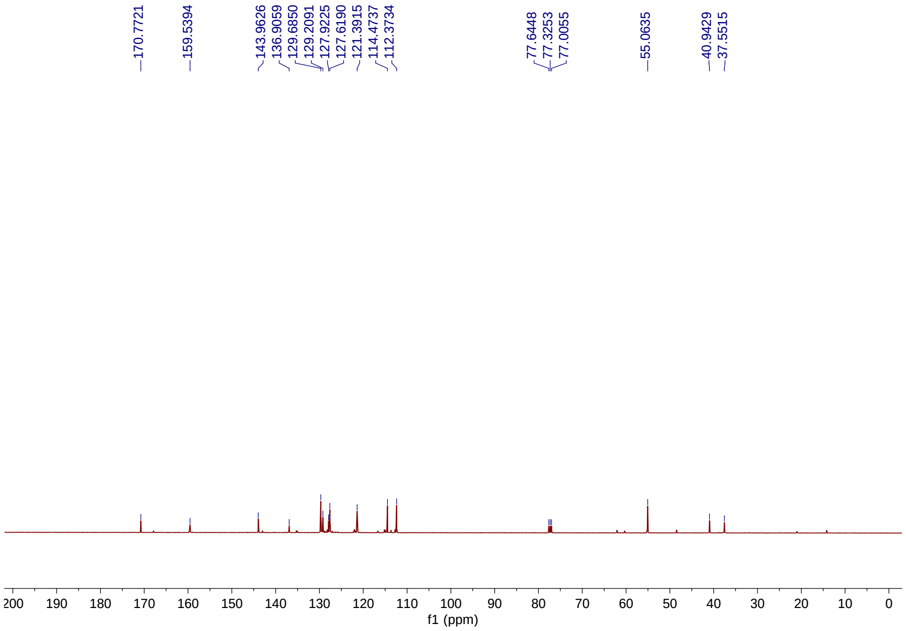

^{13}C NMR (100 MHz, $CDCl_3$) of compound (**17d**)

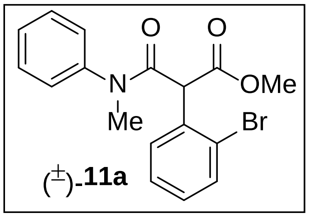

(±)-**11a**

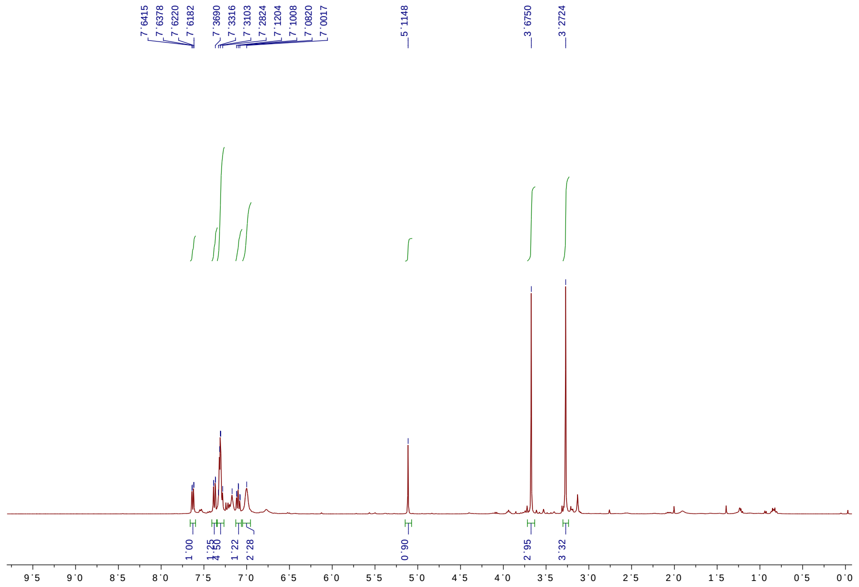

^{1}H NMR (400 MHz, $CDCl_3$) of compound (**11a**)

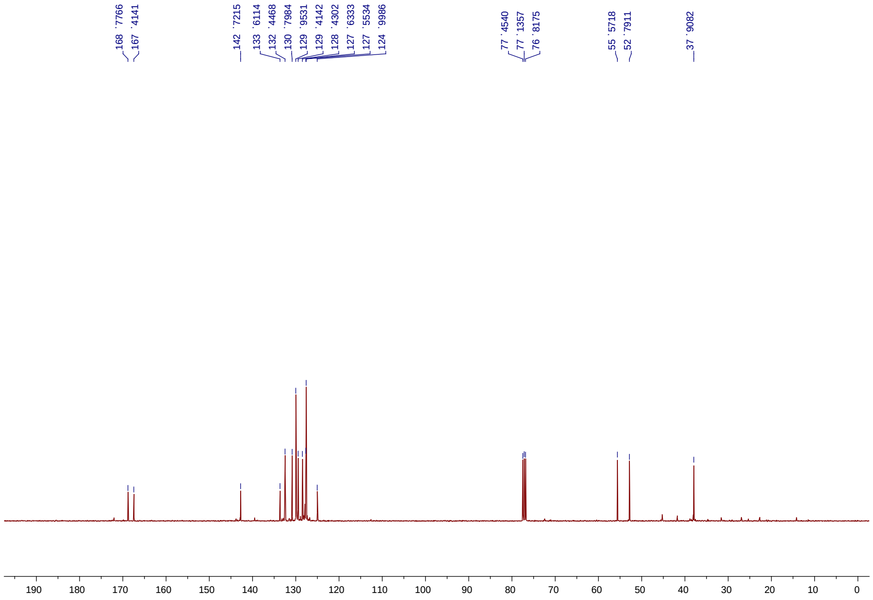

^{13}C NMR (100 MHz, $CDCl_3$) of compound (**11a)**

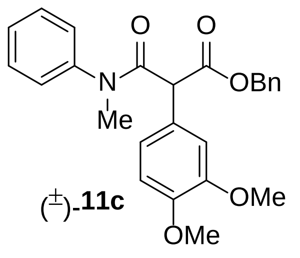

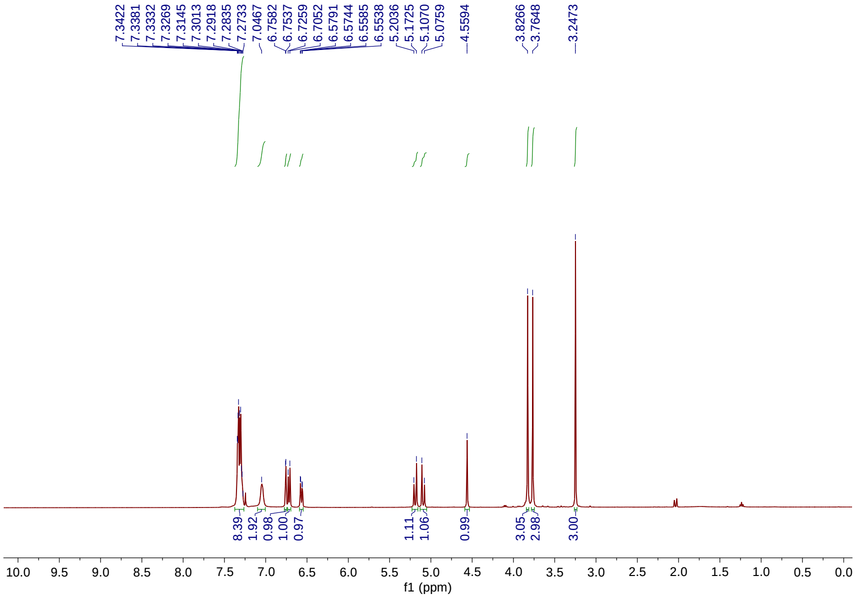

^{1}H NMR (400 MHz, $CDCl_3$) of compound (**11c**)

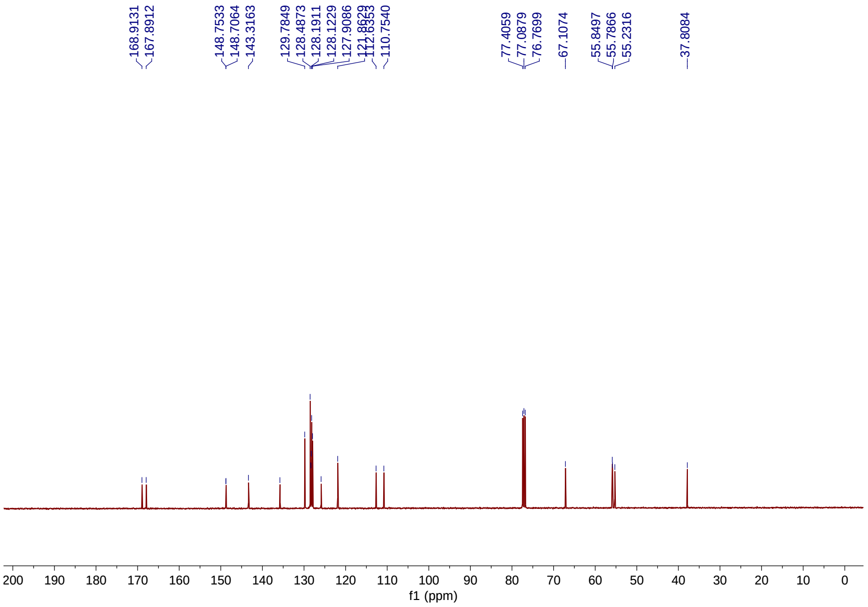

^{13}C NMR (100 MHz, $CDCl_3$) of compound (**11c**)

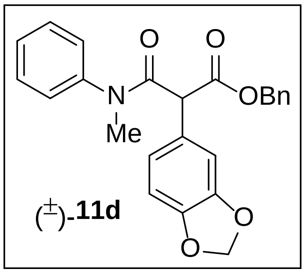

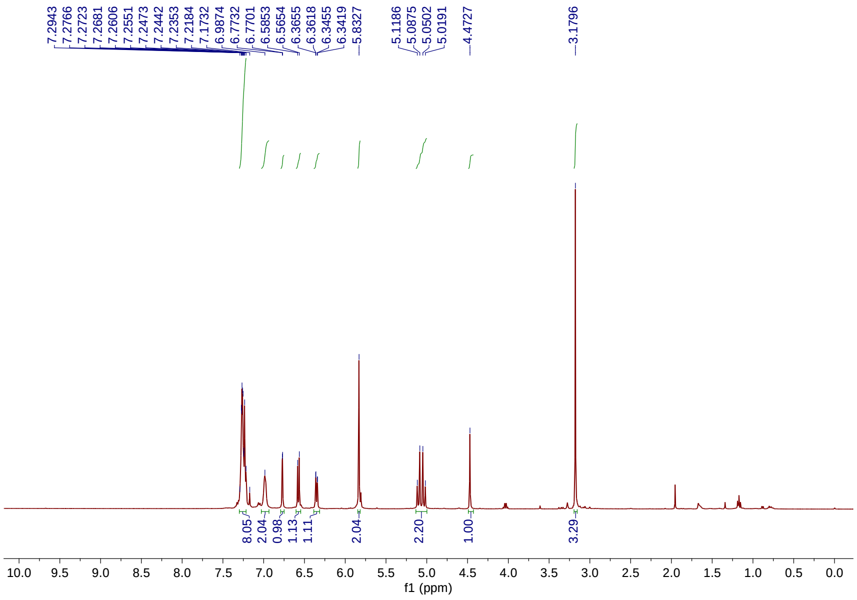

^{1}H NMR (400 MHz, $CDCl_3$) of compound (**11d**)

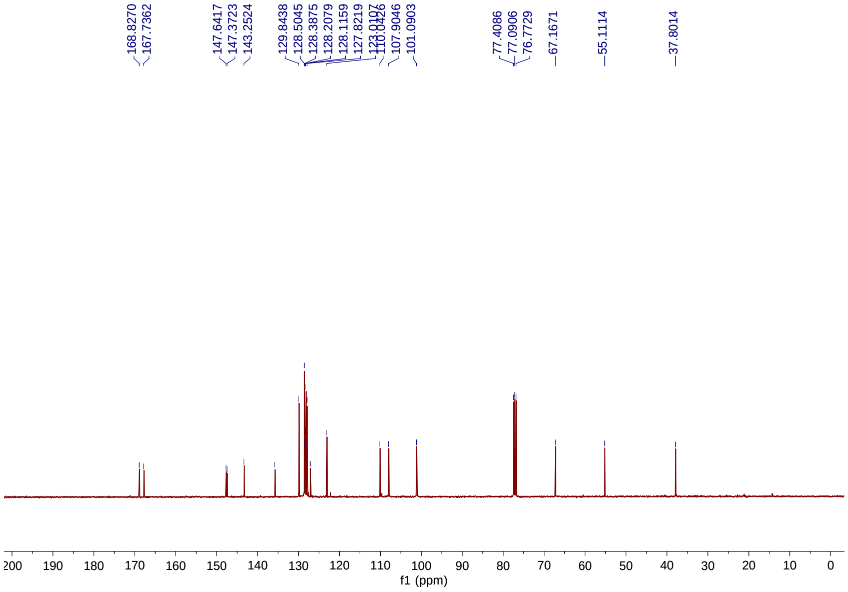

^{13}C NMR (100 MHz, $CDCl_3$) of compound (**11d**)

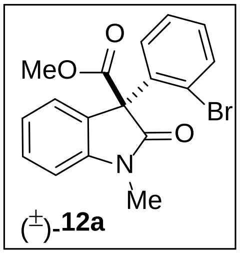

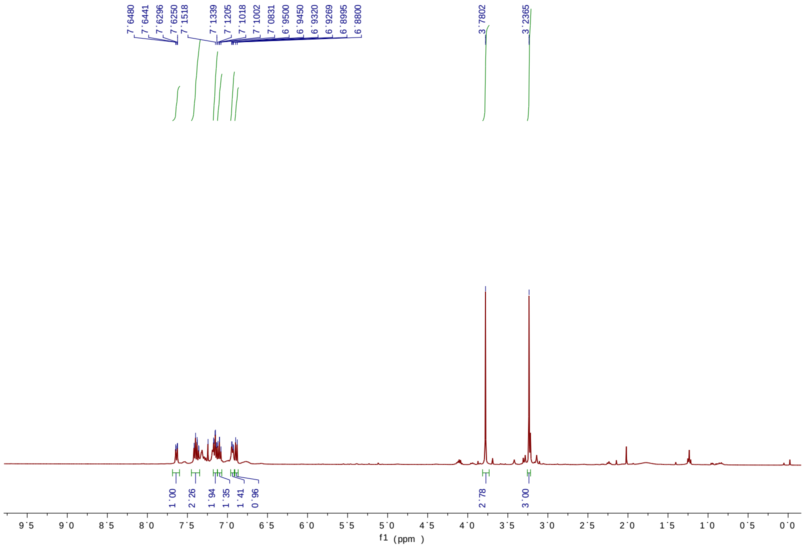

^{1}H NMR (400 MHz, $CDCl_3$) of compound (±)-**12a**

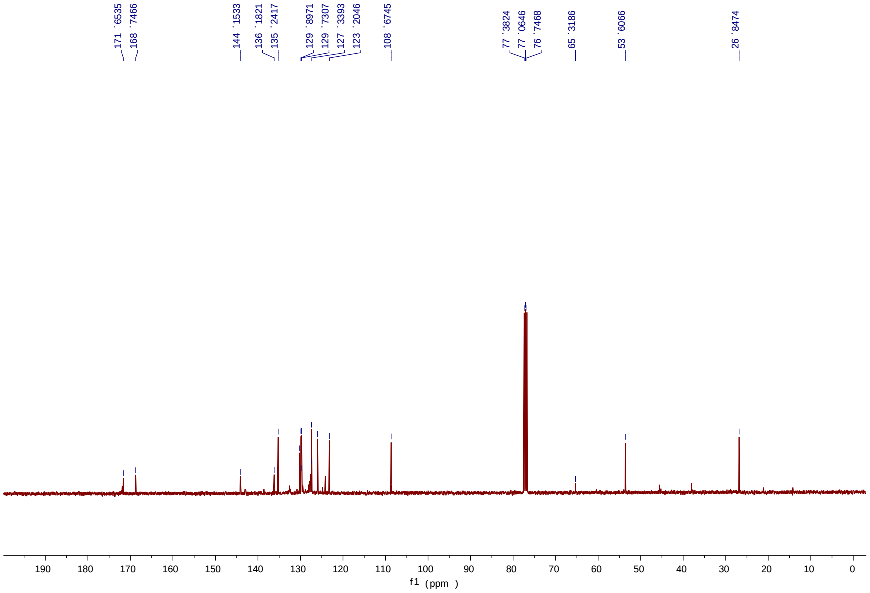

^{13}C NMR (100 MHz, $CDCl_3$) of compound (±)-**12a**

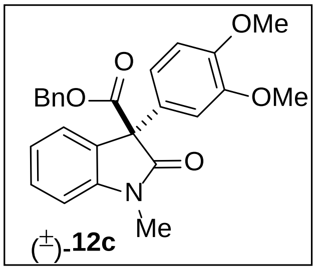

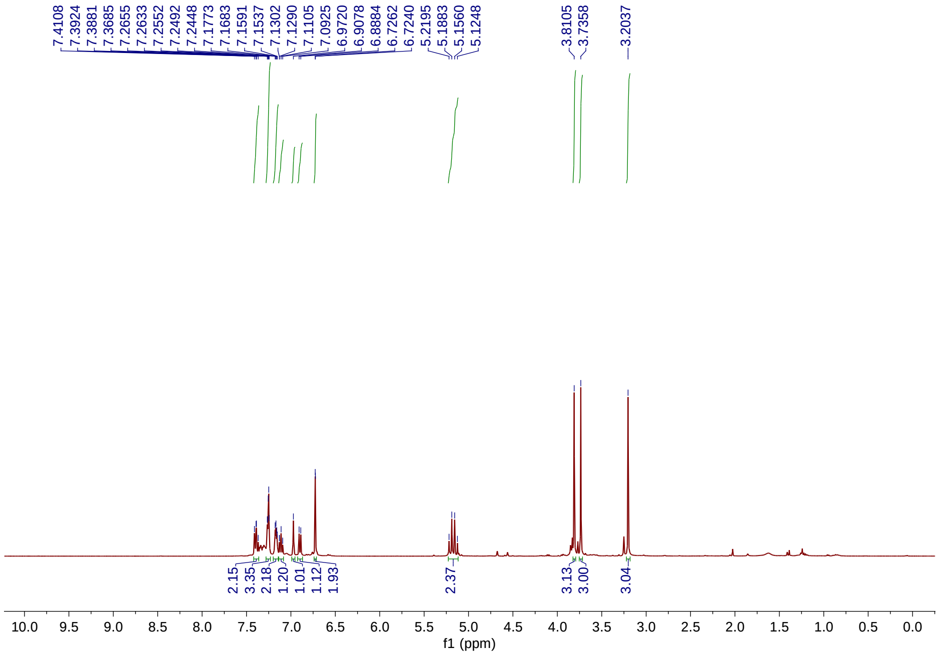

^{1}H NMR (400 MHz, $CDCl_3$) of compound (**12c**)

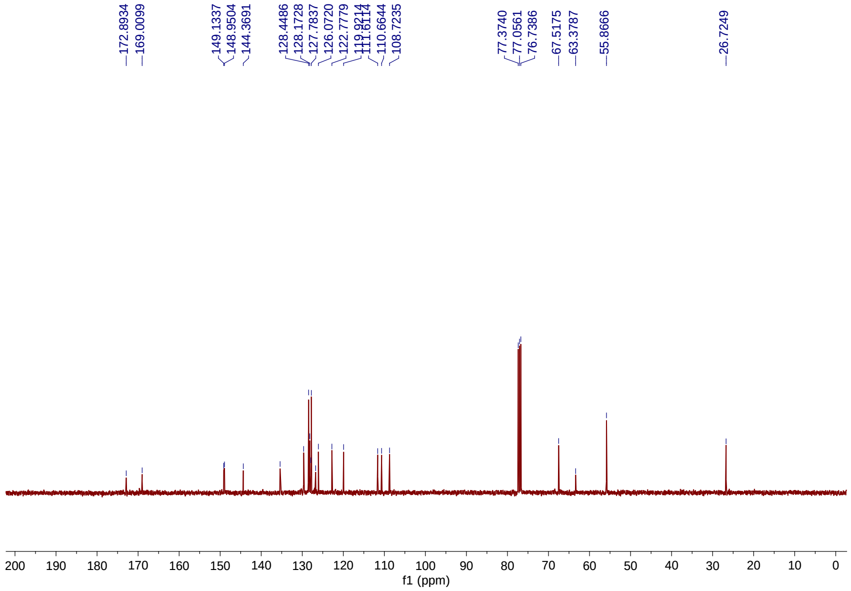

^{13}C NMR (100 MHz, $CDCl_3$) of compound (**12c**)

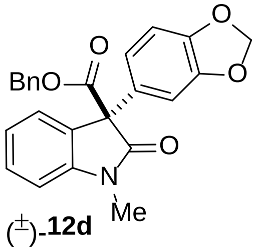

(±)-**12d**

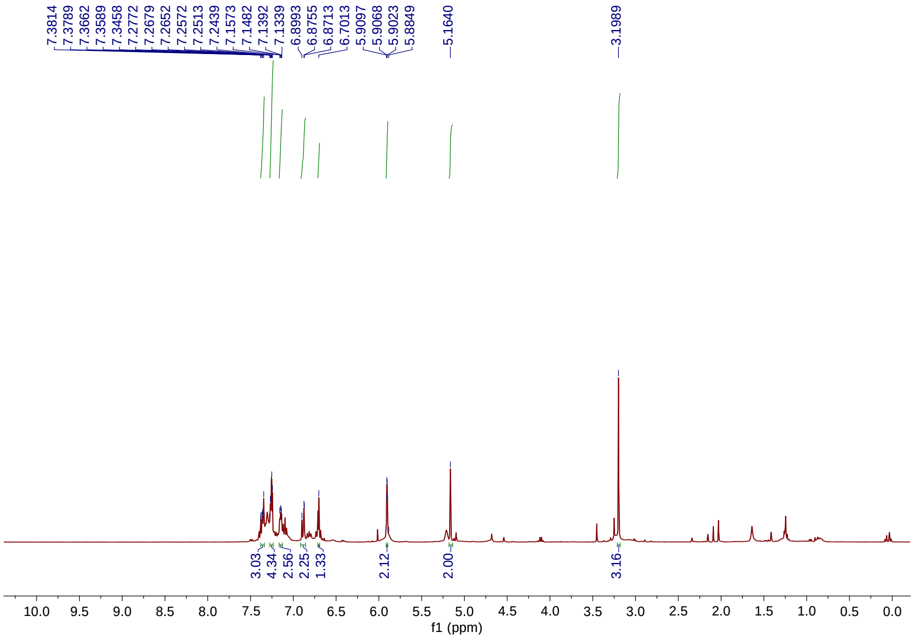

^{1}H NMR (400 MHz, $CDCl_3$) of compound (**12d**)

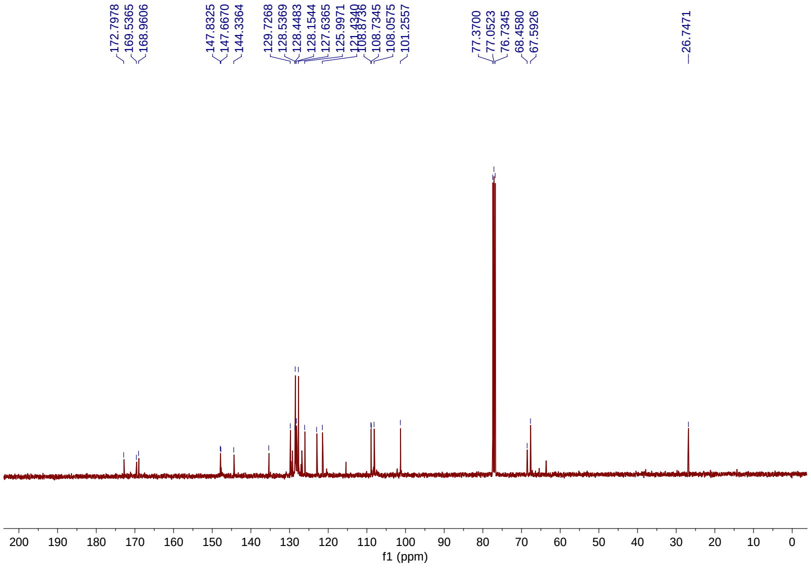

^{13}C NMR (100 MHz, $CDCl_3$) of compound (**12d**)

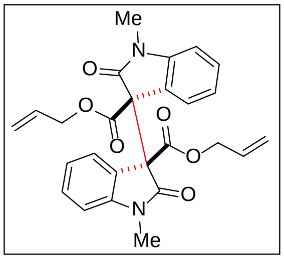

trans (±)-**19c**
Major diastereomer

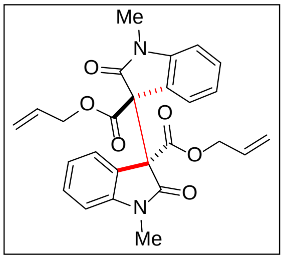

meso-(**19c**)
Minor diastereomer

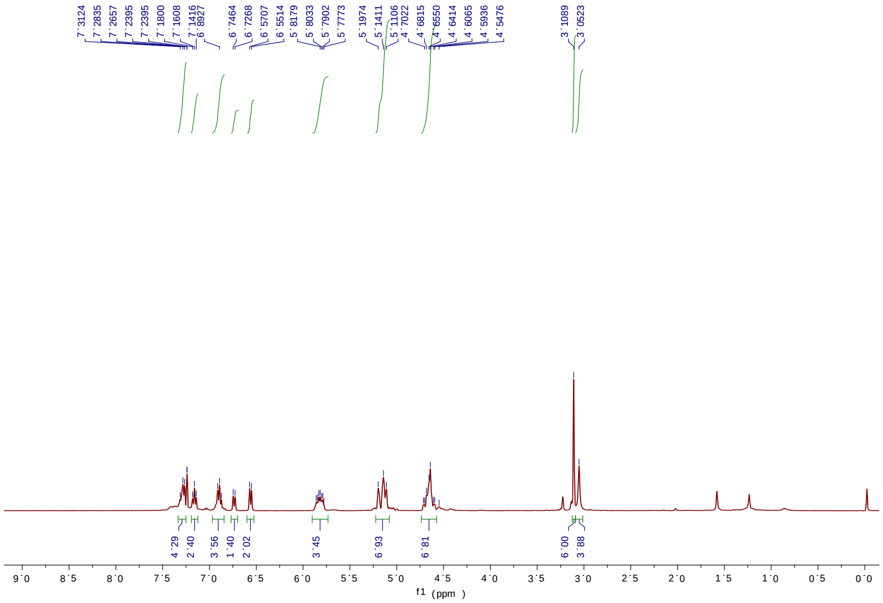

^{1}H NMR(400 MHz, CDCl$_3$) of compound (±)-**19c**

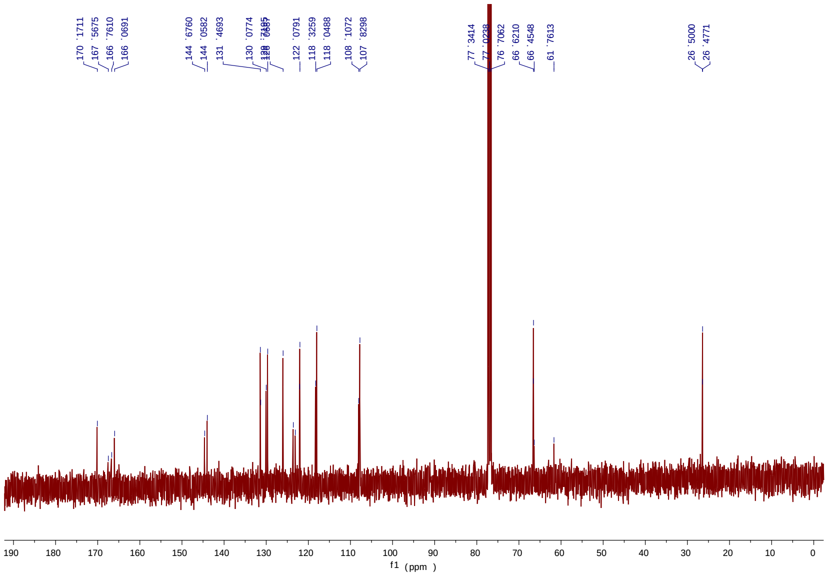

^{13}C NMR(100 MHz, CDCl$_3$) of compound (±)-**19c**

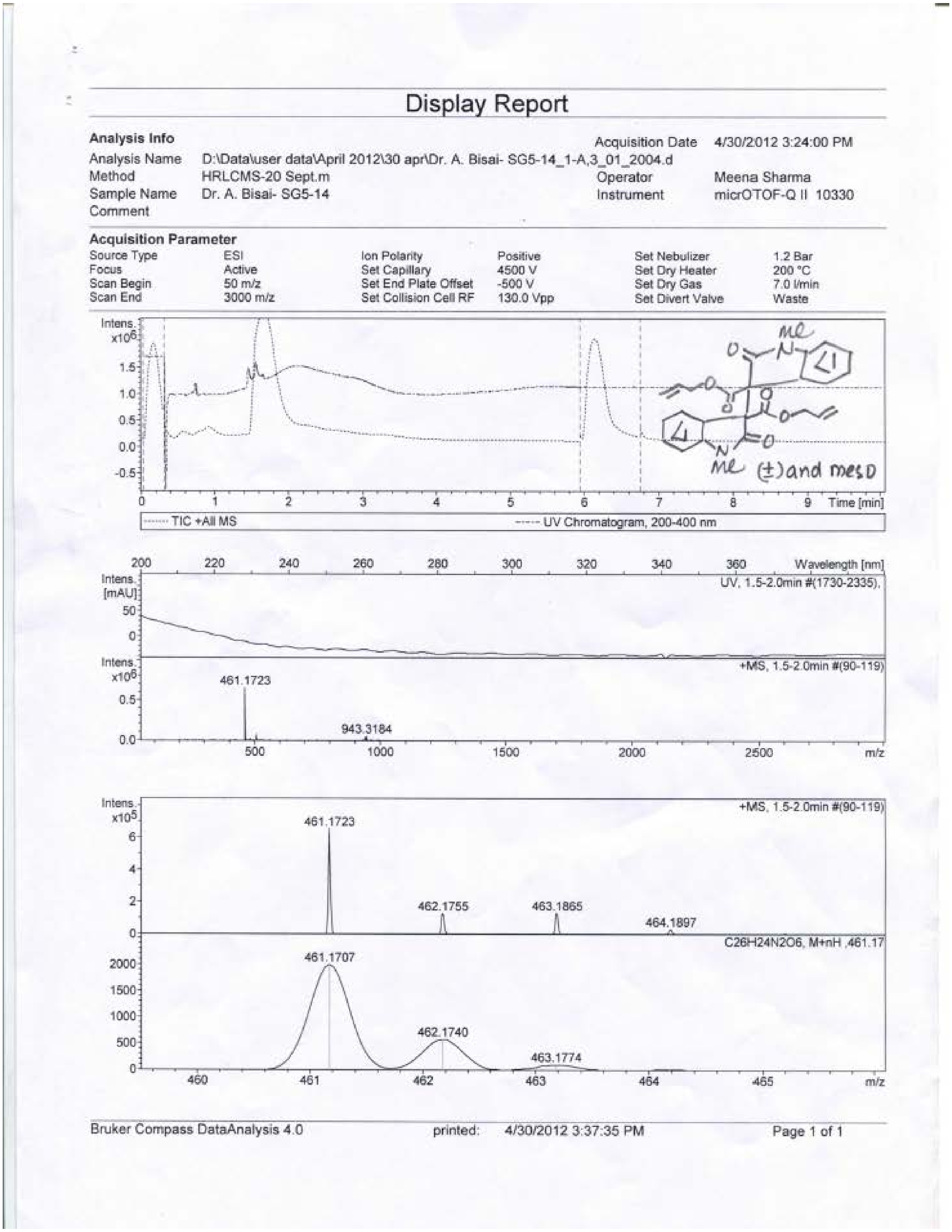
Display Report

Analysis Info

Analysis Name	D:\Data\user data\April 2012\30 apr\Dr. A. Bisai- SG5-14_1-A,3_01_2004.d	Acquisition Date	4/30/2012 3:24:00 PM
Method	HRLCMS-20 Sept.m	Operator	Meena Sharma
Sample Name	Dr. A. Bisai- SG5-14	Instrument	micrOTOF-Q II 10330
Comment			

Acquisition Parameter

Source Type	ESI	Ion Polarity	Positive	Set Nebulizer	1.2 Bar
Focus	Active	Set Capillary	4500 V	Set Dry Heater	200 °C
Scan Begin	50 m/z	Set End Plate Offset	-500 V	Set Dry Gas	7.0 l/min
Scan End	3000 m/z	Set Collision Cell RF	130.0 Vpp	Set Divert Valve	Waste

Bruker Compass DataAnalysis 4.0 printed: 4/30/2012 3:37:35 PM Page 1 of 1

Scanned copy of mass spectrum of compound (±)-**19c**

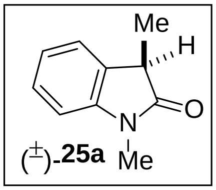

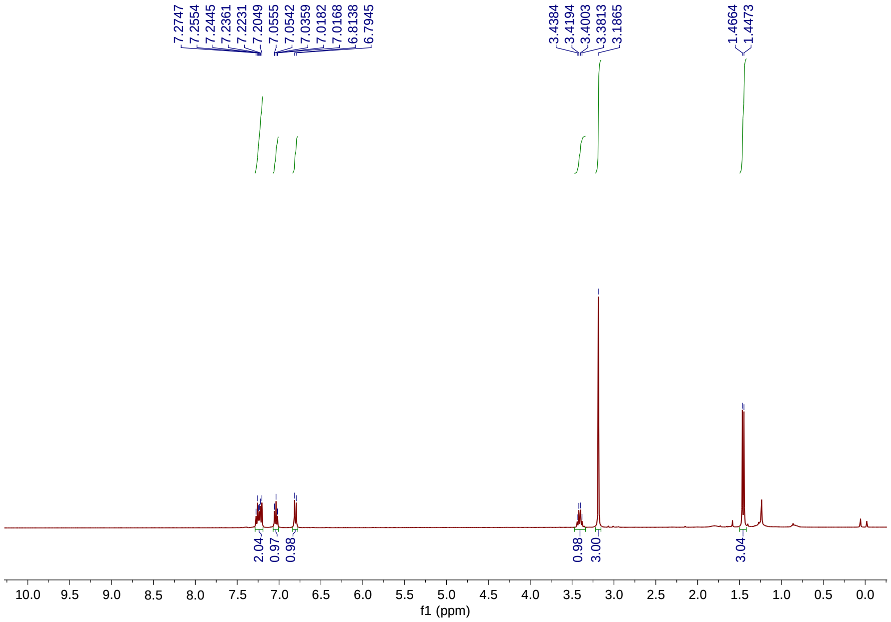

^{1}H NMR (400 MHz, $CDCl_3$) of compound (**25a**)

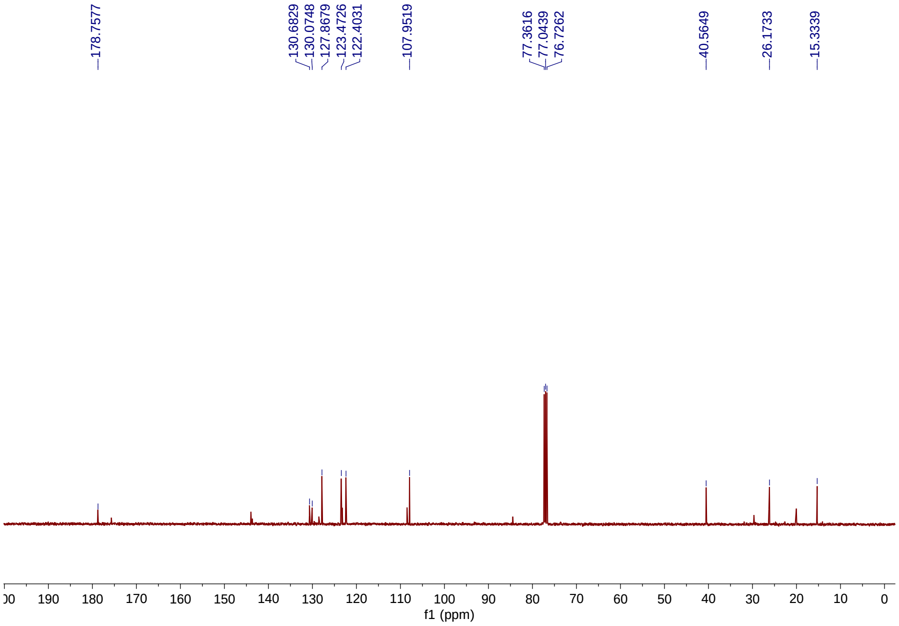

^{13}C NMR (100 MHz, $CDCl_3$) of compound (**25a**)

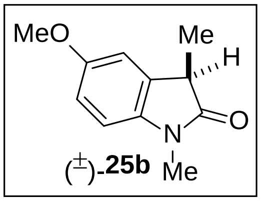

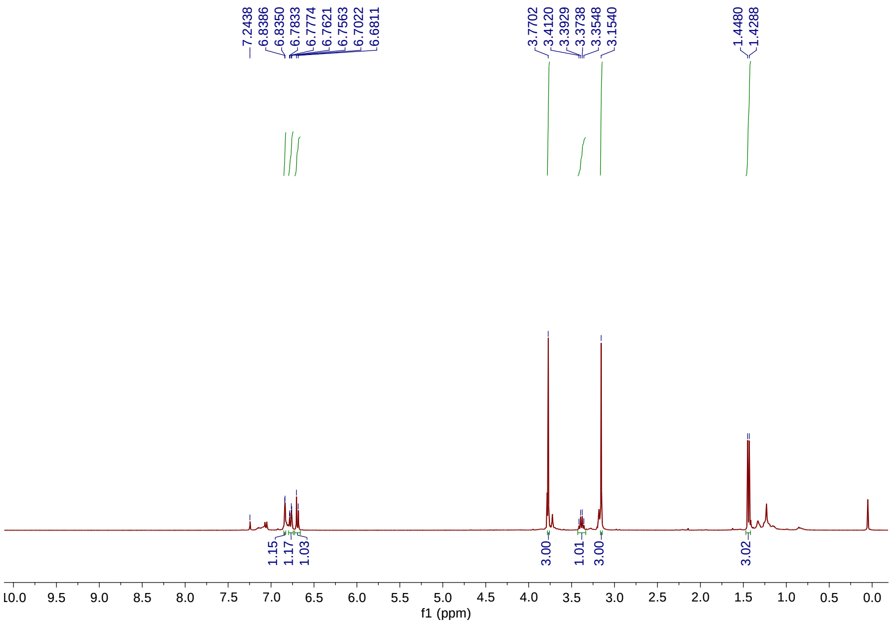

^{1}H NMR (400 MHz, $CDCl_3$) of compound (**25b**)

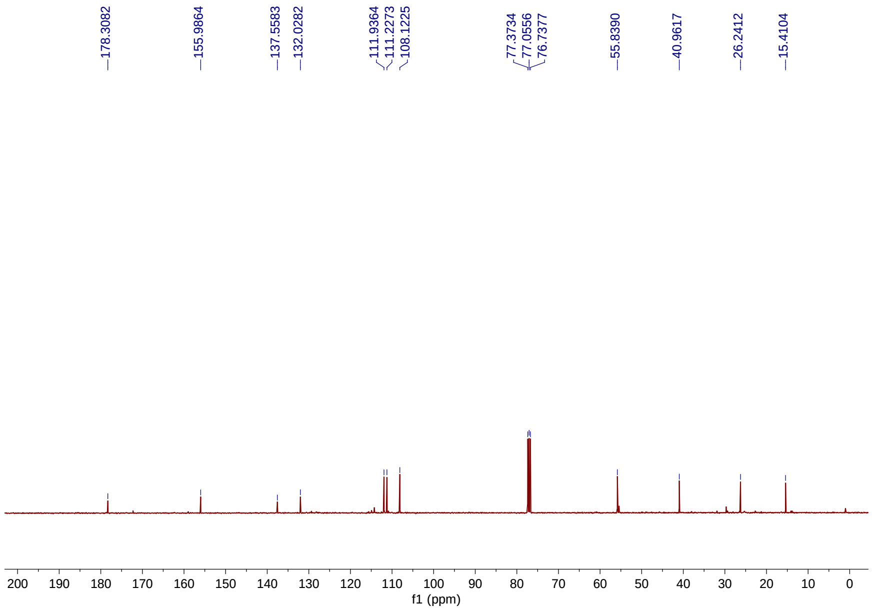

^{13}C NMR (100 MHz, $CDCl_3$) of compound (**25b**)

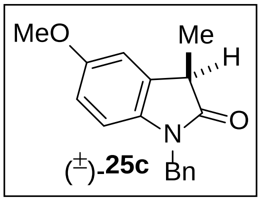

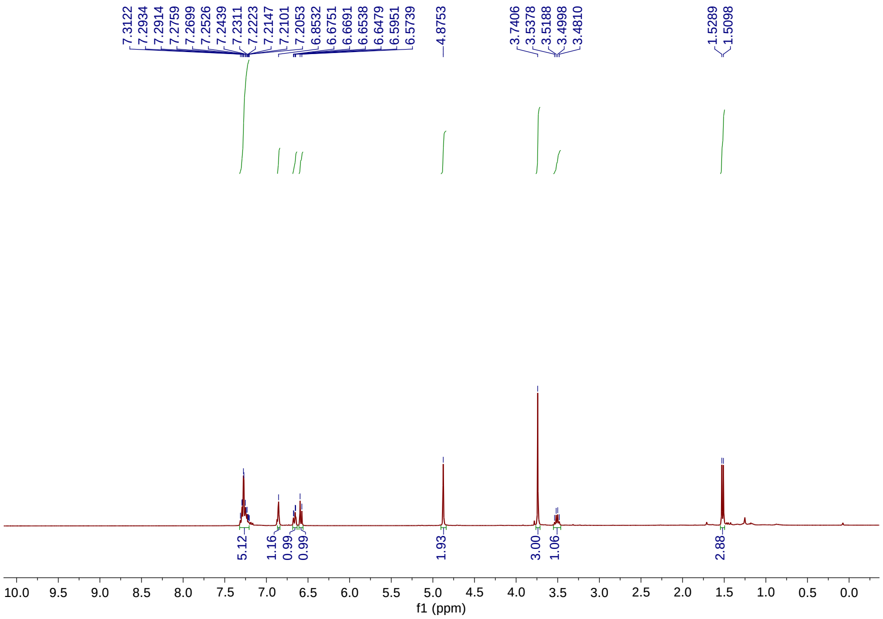

^{1}H NMR (400 MHz, $CDCl_3$) of compound (**25c**)

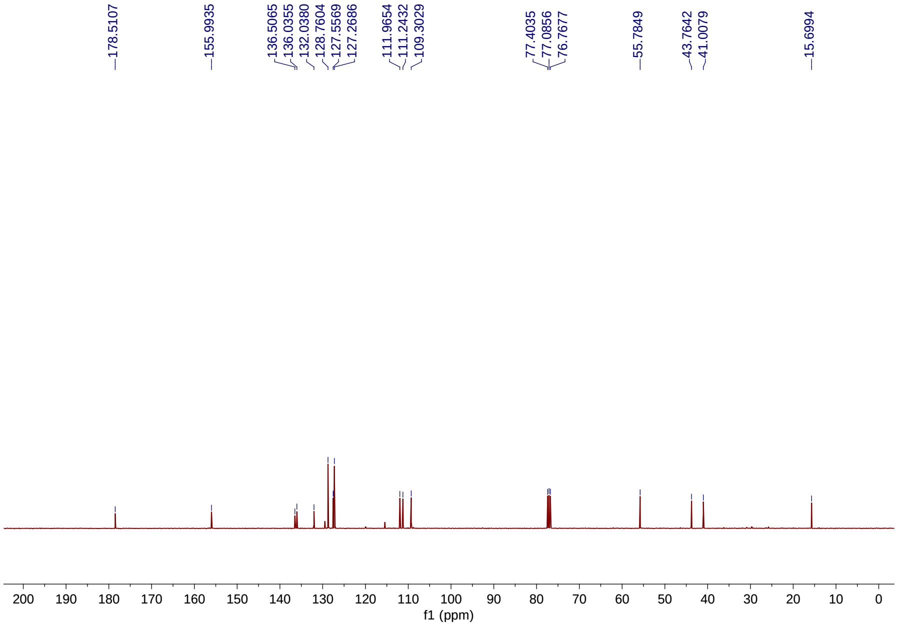

^{13}C NMR (100 MHz, $CDCl_3$) of compound (**25c**)

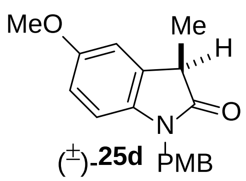

(±)-**25d**

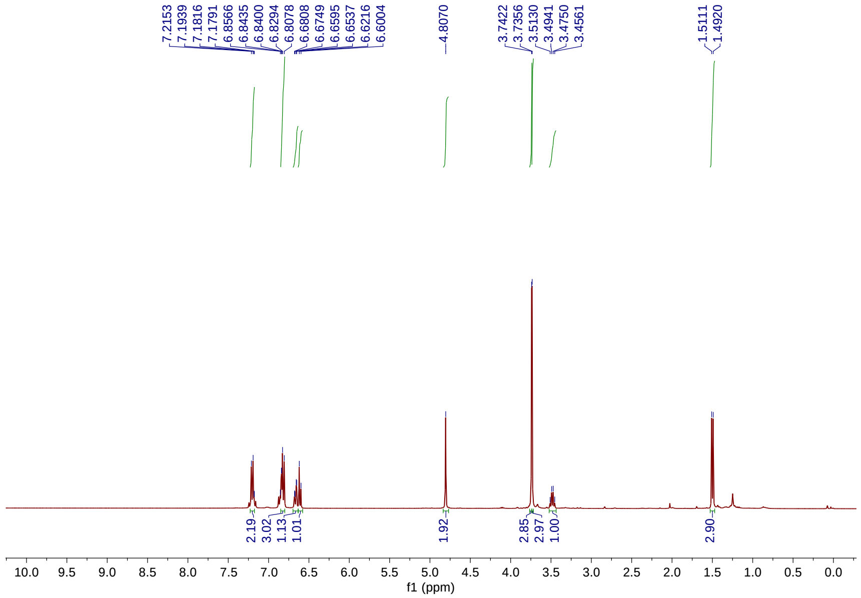

^{1}H NMR (400 MHz, $CDCl_3$) of compound (**25d**)

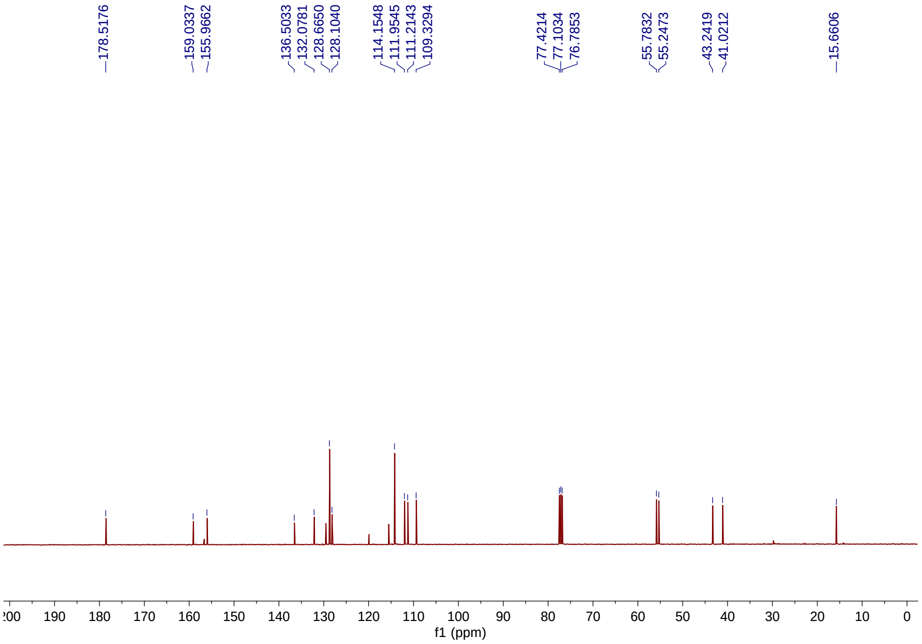

^{13}C NMR (100 MHz, $CDCl_3$) of compound (**25d**)

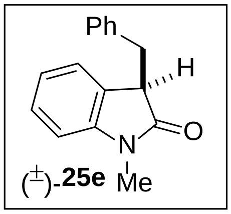

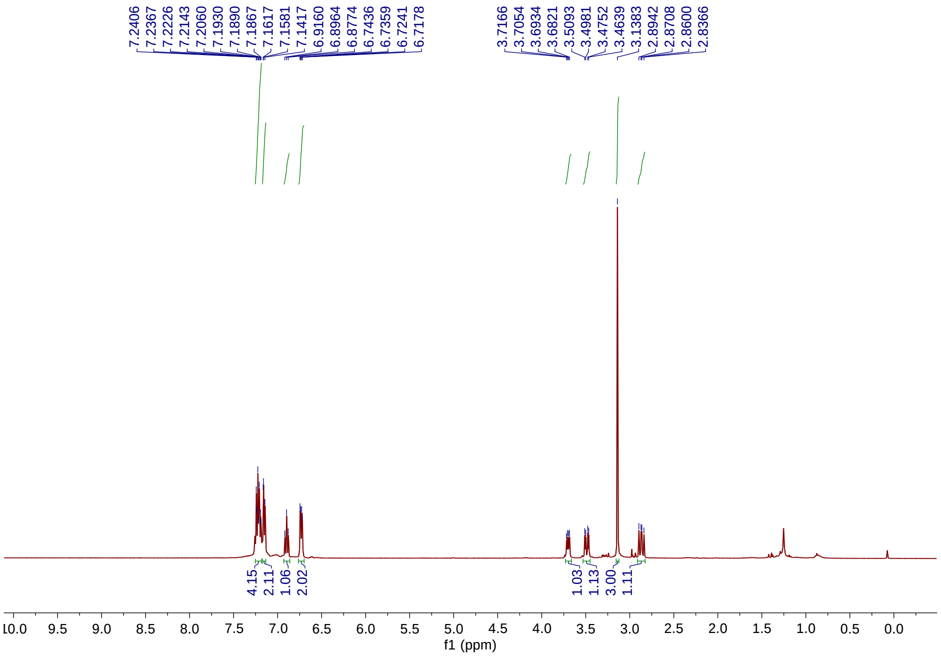

^{1}H NMR (400 MHz, $CDCl_3$) of compound (**25e**)

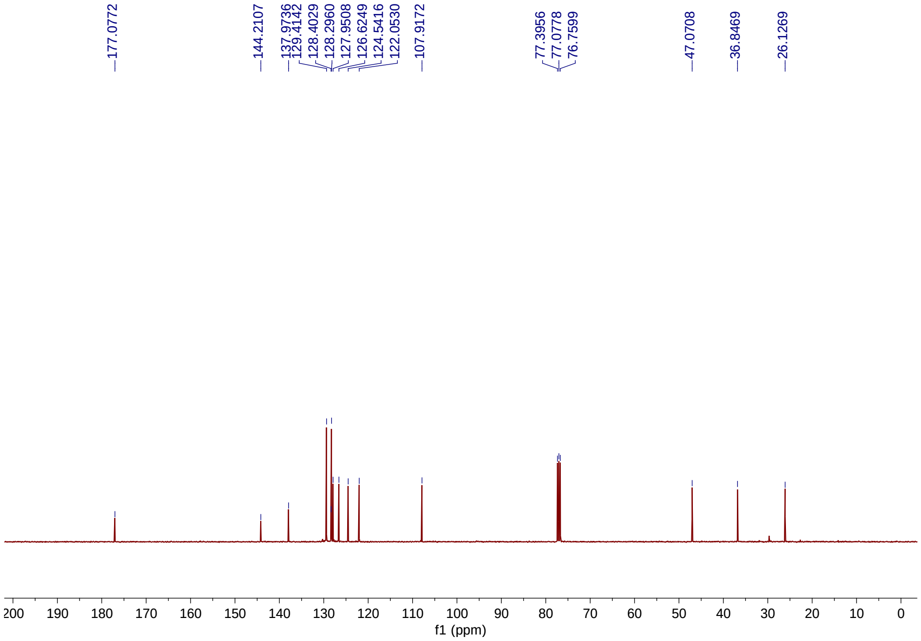

^{13}C NMR (100 MHz, $CDCl_3$) of compound (**25e**)

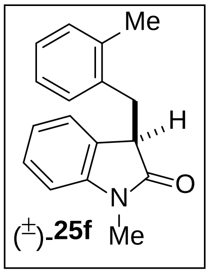

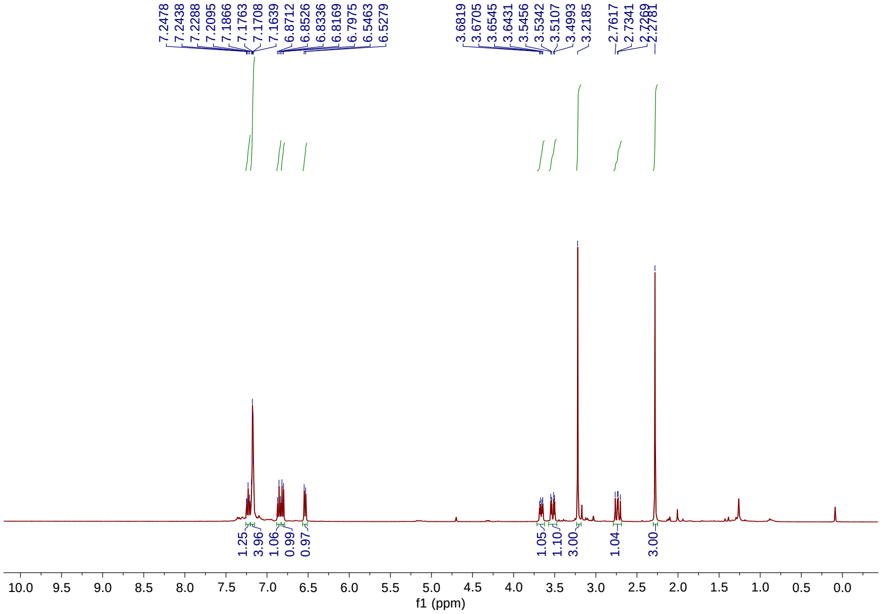

^{1}H NMR (400 MHz, CDCl$_3$) of compound (**25f**)

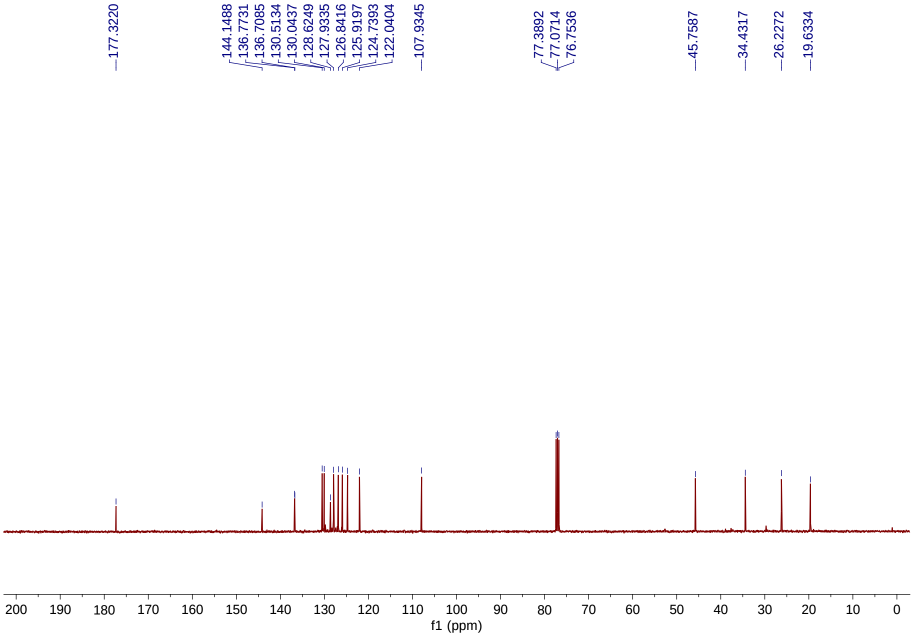

^{13}C NMR (100 MHz, CDCl$_3$) of compound (**25f**)

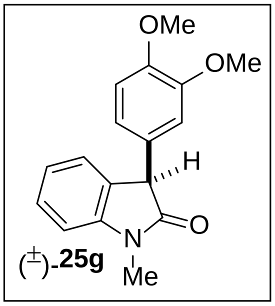

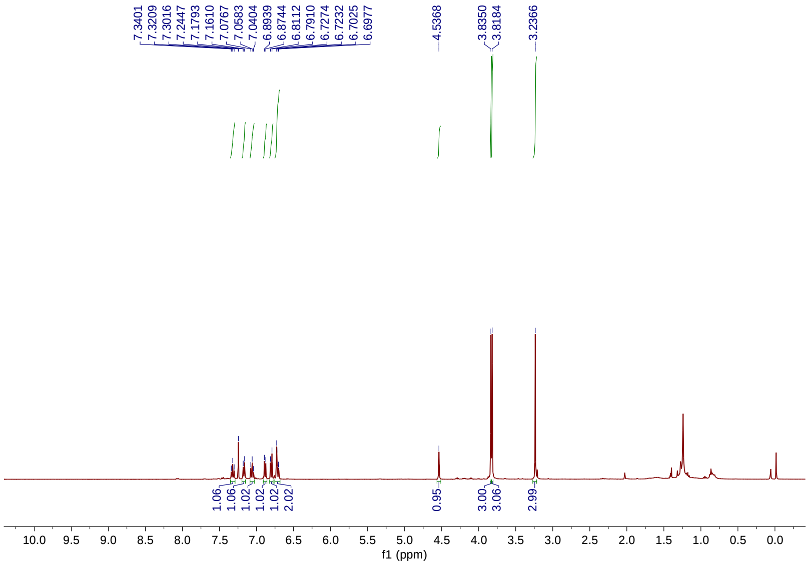

^{1}H NMR (400 MHz, $CDCl_3$) of compound (**25g**)

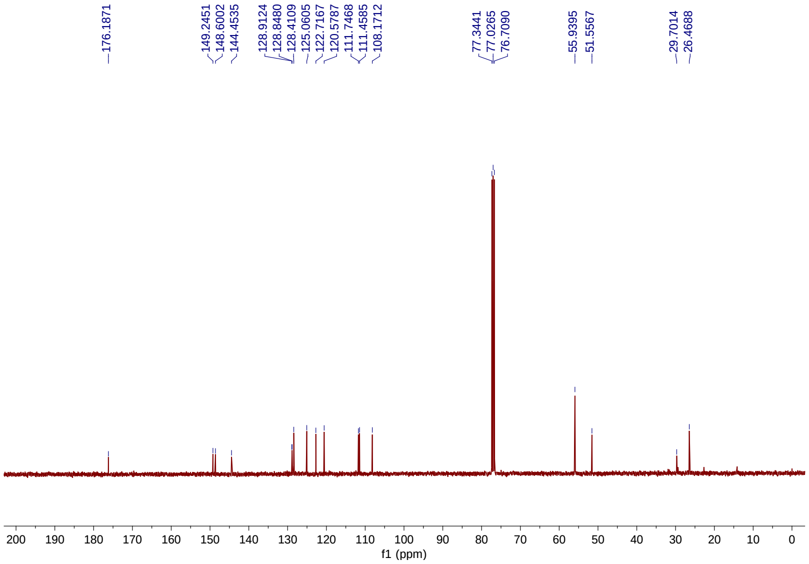

^{13}C NMR (100 MHz, $CDCl_3$) of compound (**25g**)

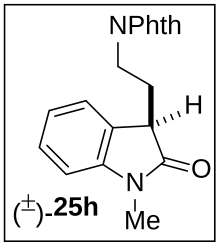

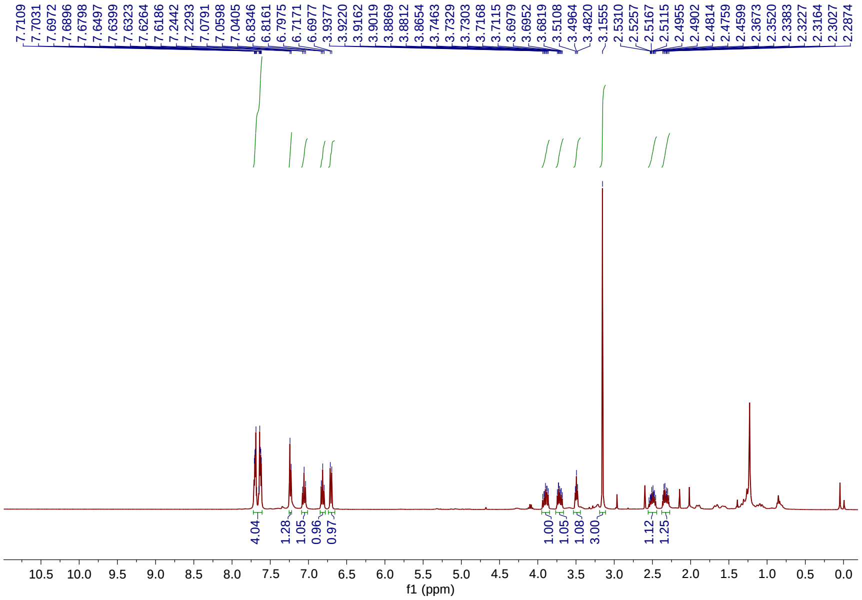

^{1}H NMR (400 MHz, $CDCl_3$) of compound (**25h**)

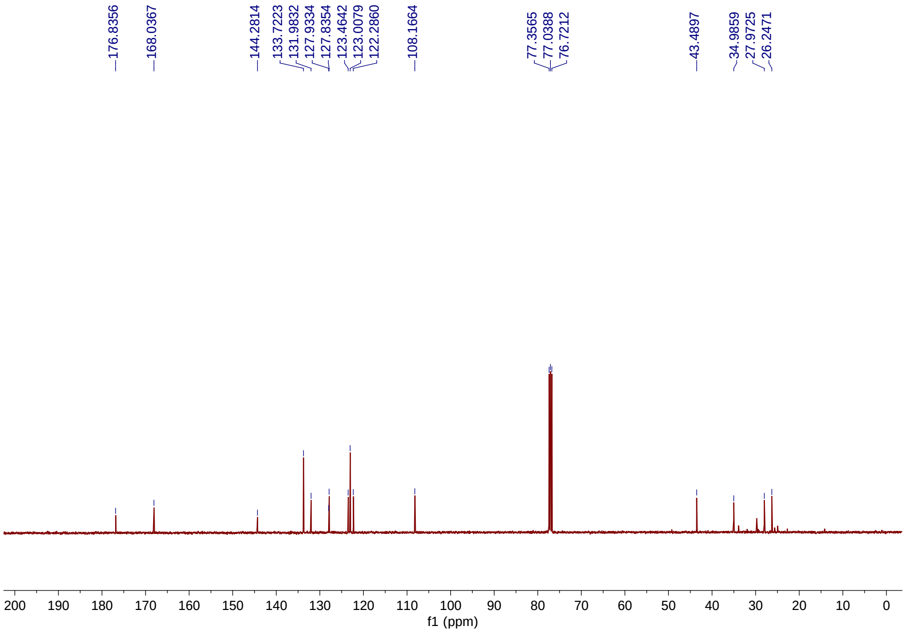

^{13}C NMR (100 MHz, $CDCl_3$) of compound (**25h**)
